# Supplementary material for: Global gut microbiome atlas identifies epidemiologic-stage-specific signatures in inflammatory bowel disease
Source: Cell Rep Med. 2026 Aug 10;7(9):102974. doi: 10.1016/j.xcrm.2026.102974 (PMC13589510; doi:10.1016/j.xcrm.2026.102974)
Supplement: Supplementary file 1 [file mmc1.pdf]

Cell Reports Medicine, Volume *7*

Supplemental information

Global gut microbiome atlas identifies epidemiologic-stage-specific signatures in inflammatory bowel disease

Jinxia Zhai (翟锦霞), Yingjie Li (李迎杰), Jiameng Liu (刘佳梦), Xinyi Su (苏心怡), Runze Cui (崔润泽), Dianyu Zheng (郑殿宇), Yuhan Sun (孙羽晗), Jingsheng Yu (于景盛), and Cong Dai (戴聪)

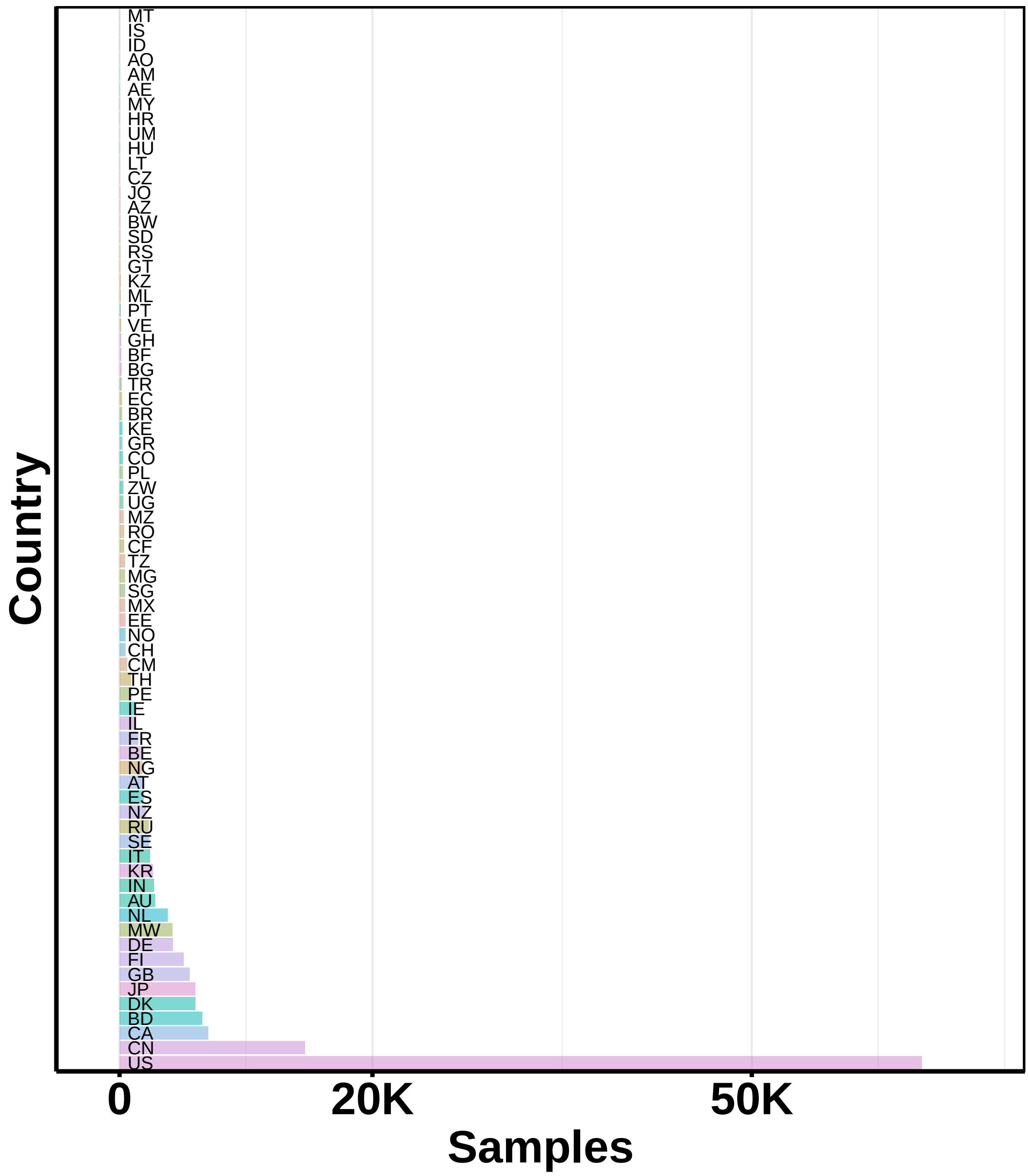

Figure S1. Regional structure. Related to Figure 1

A bar plot illustrating the number of samples contributed by each of the 70 countries analyzed. The x-axis indicates the total number of samples, and the y-axis lists all countries evaluated.

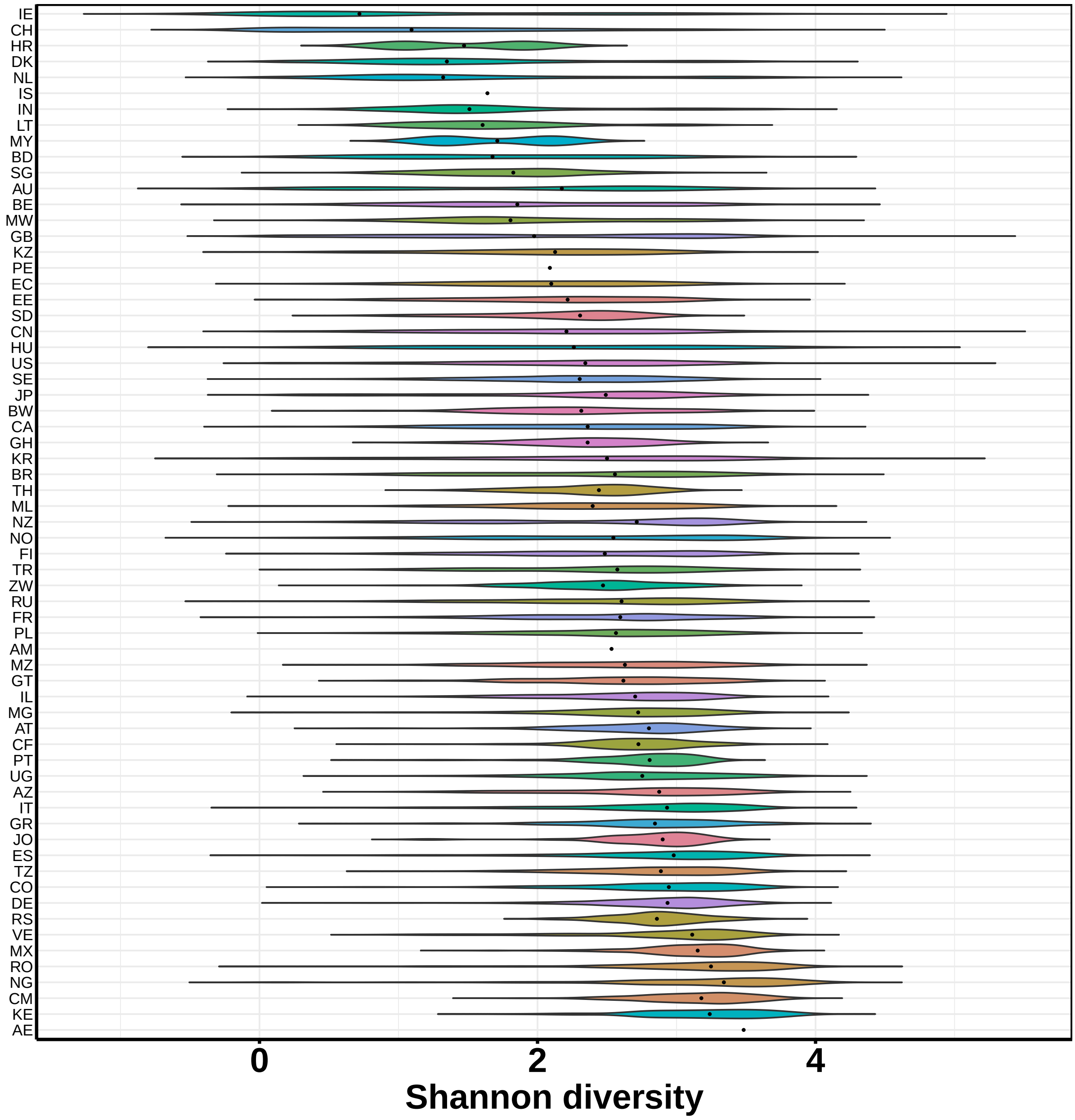

Figure S2. Regional structure. Related to Figure 1

Violin plots showing the distributions of observed Shannon index across the 70 countries. Vertical lines within each violin indicate the median values.

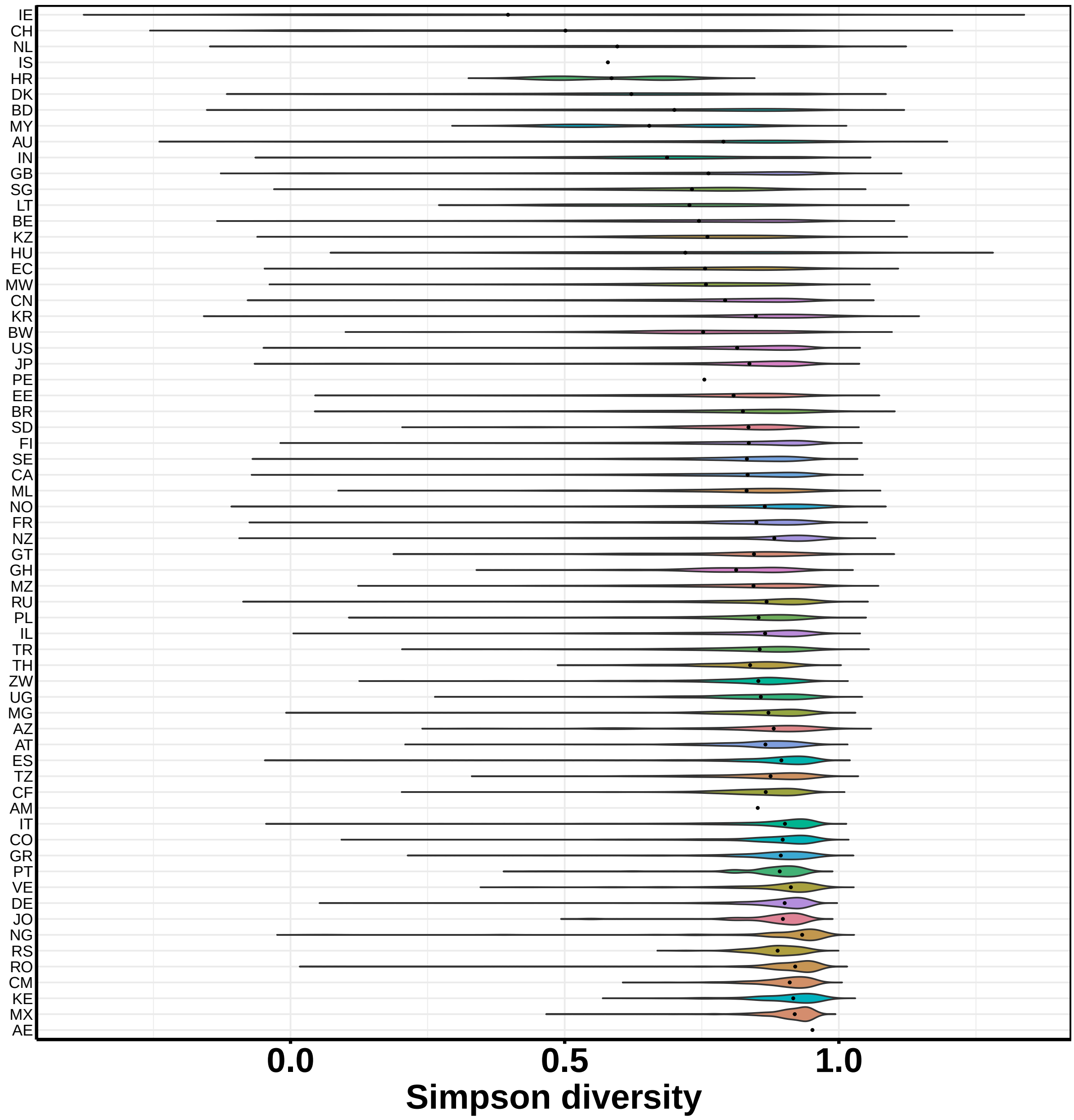

Figure S3. Regional structure. Related to Figure 1

Violin plots showing the distributions of observed Simpson index across the 70 countries. Vertical lines within each violin indicate the median values.

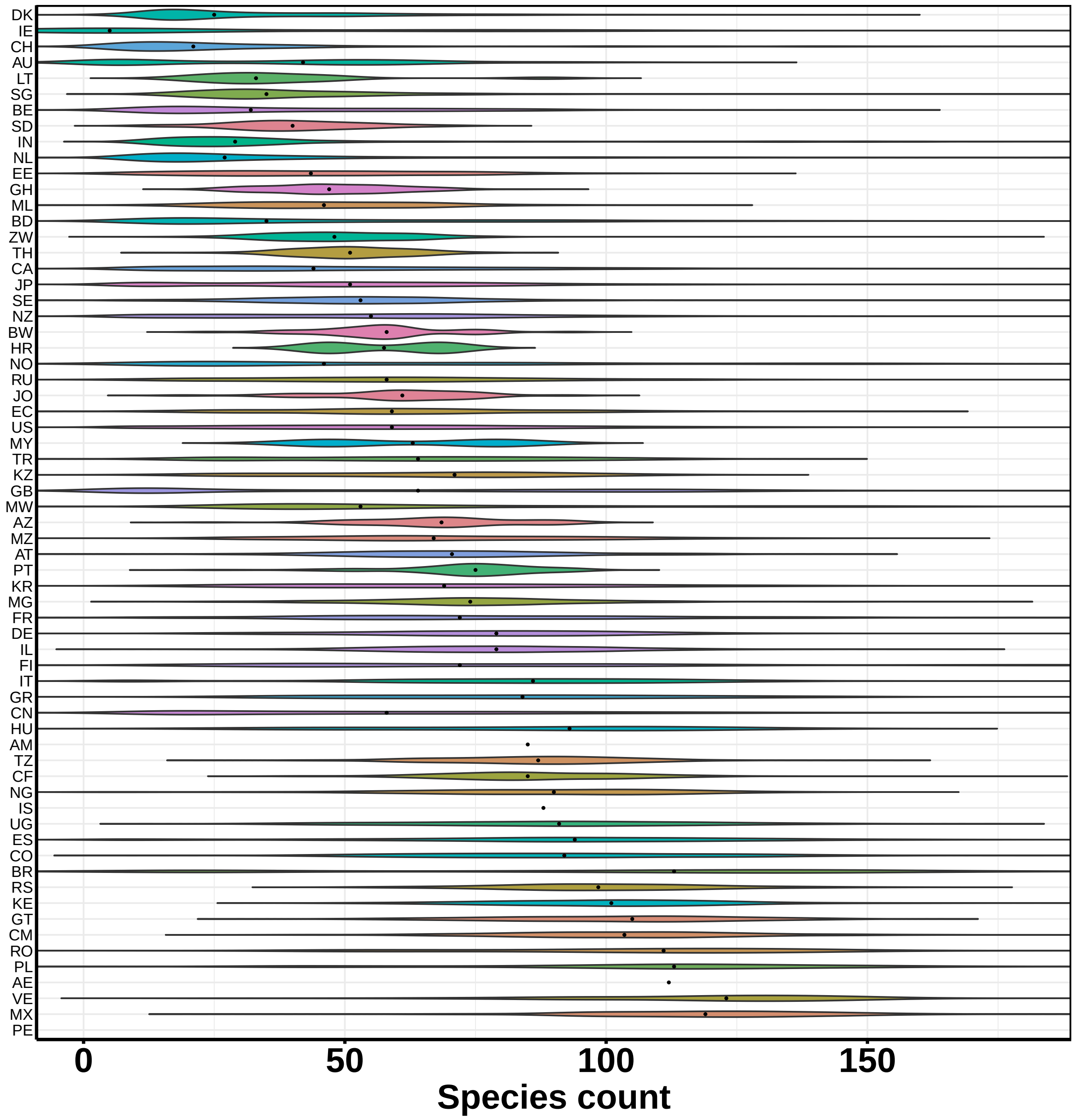

Figure S4. Regional structure. Related to Figure 1

Violinplots showing the distributions of observed species count values across the 70 countries . Vertical lines within each violin indicate the median values.

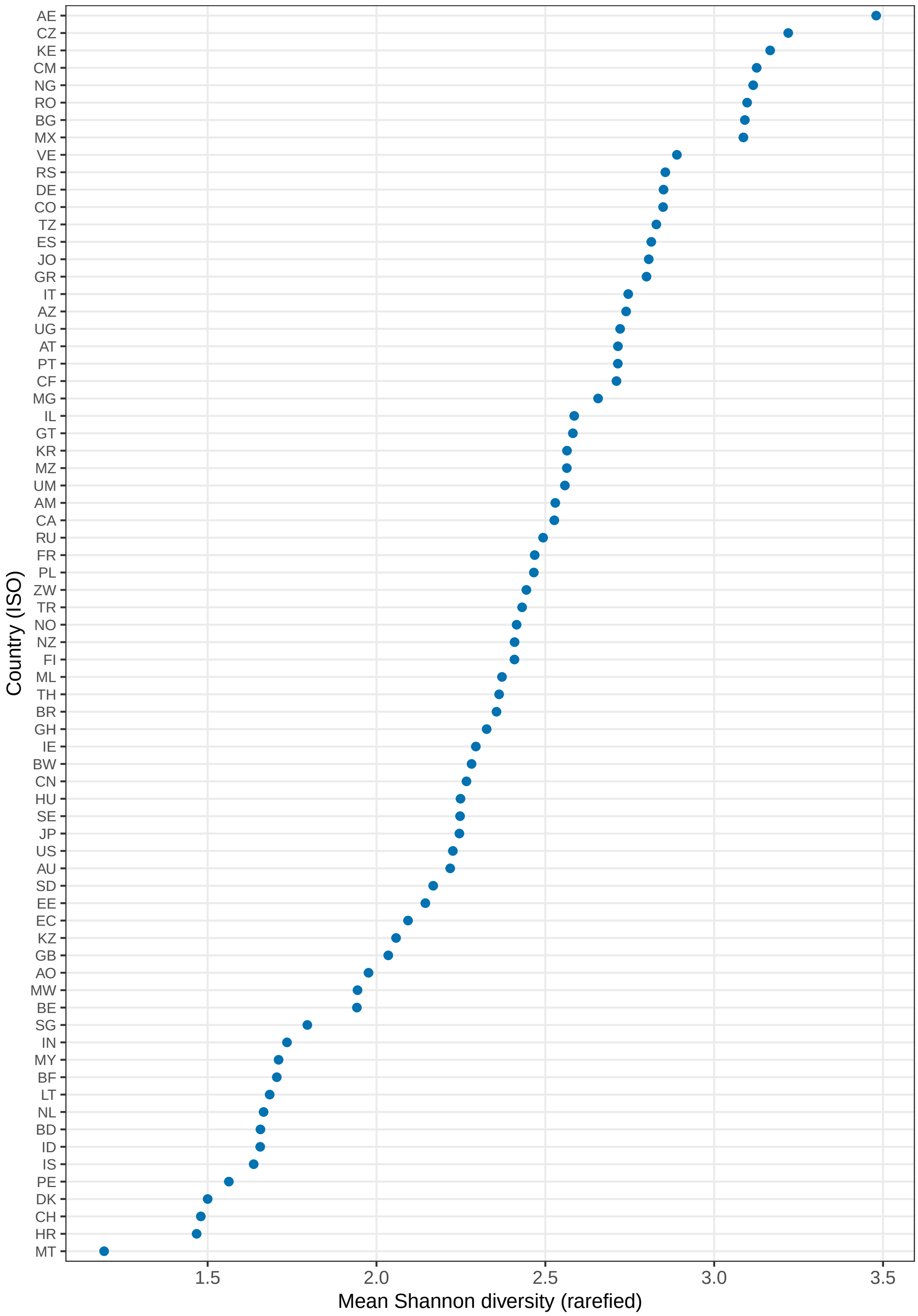

Figure S5. Rarefaction diversity estimates. Related to Figure 1

The x-axis denotes Shannon diversity, and the y-axis lists all countries evaluated in the rarefaction analysis. Each point represents the mean α diversity calculated across 1,000 iterations (see Methods). Statistical estimates were derived using the R t.test function.

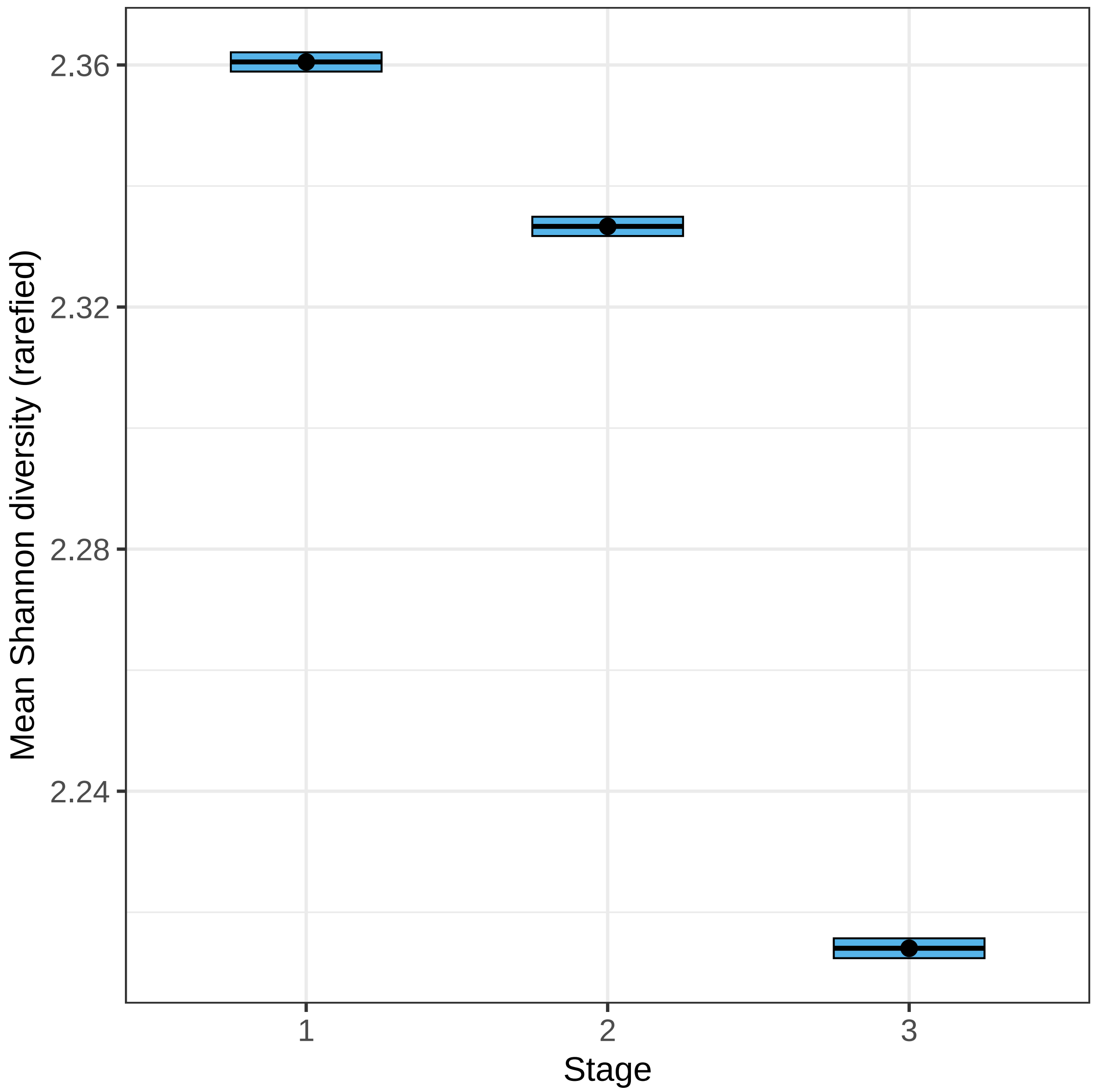

Figure S6. Rarefaction diversity estimates. Related to Figure 1

The y-axis denotes Shannon diversity, and the x-axis lists regions stratified by the three IBD epidemiologic stages evaluated in the rarefaction analysis. Each point represents the mean α diversity calculated across 1,000 iterations (see Methods). Statistical estimates were computed using the R t.test function.

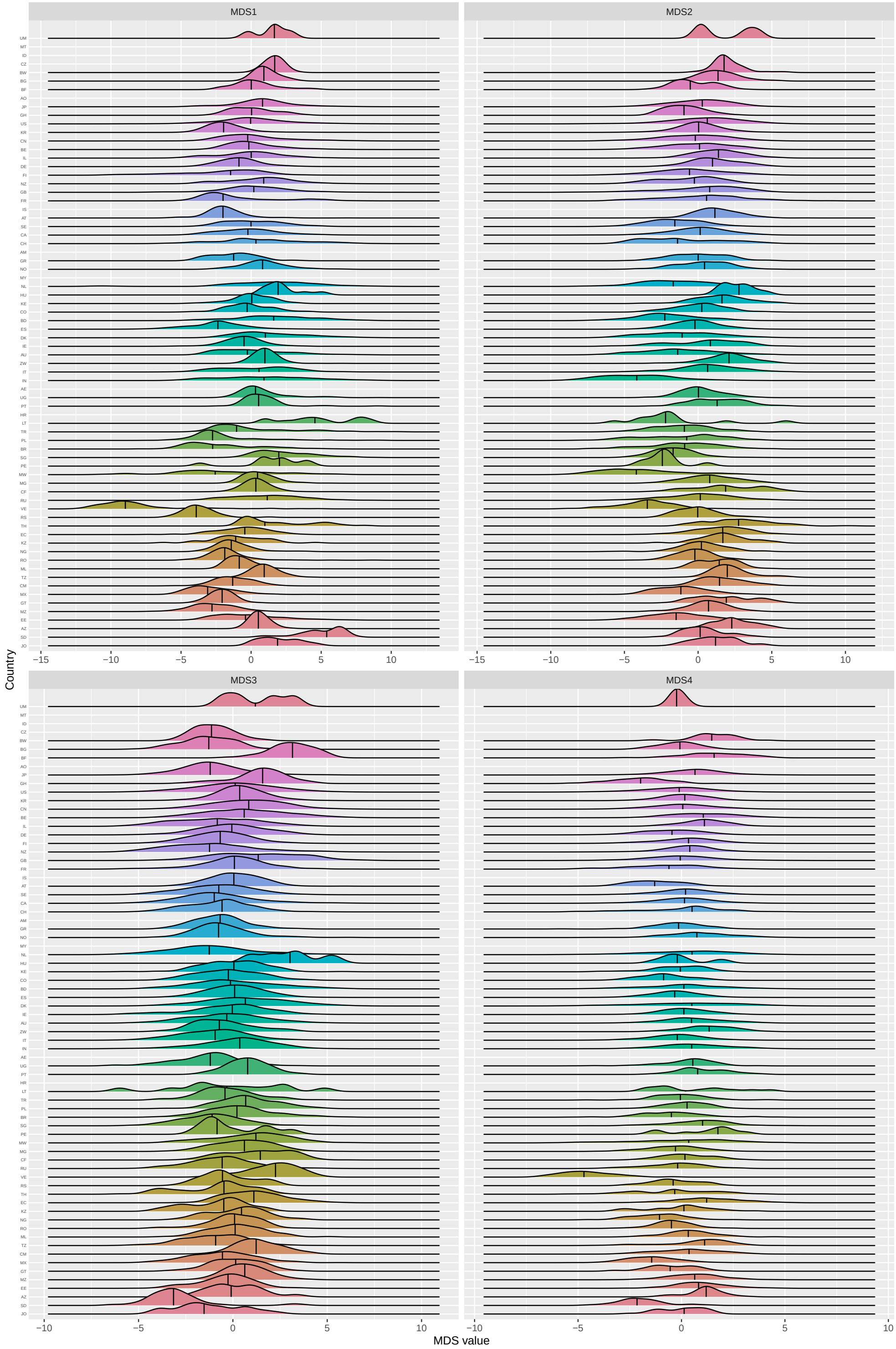

Figure S7. Ordination results. Related to Figure 1

This figure comprises a series of panels illustrating the results of a principal coordinates analysis (PCoA) of samples from 70 countries. Factors MDS1 through MDS4 are shown. Each panel depicts a single factor; the x-axis indicates the value of that factor, and the y-axis represents the relative frequency of that value within each country.

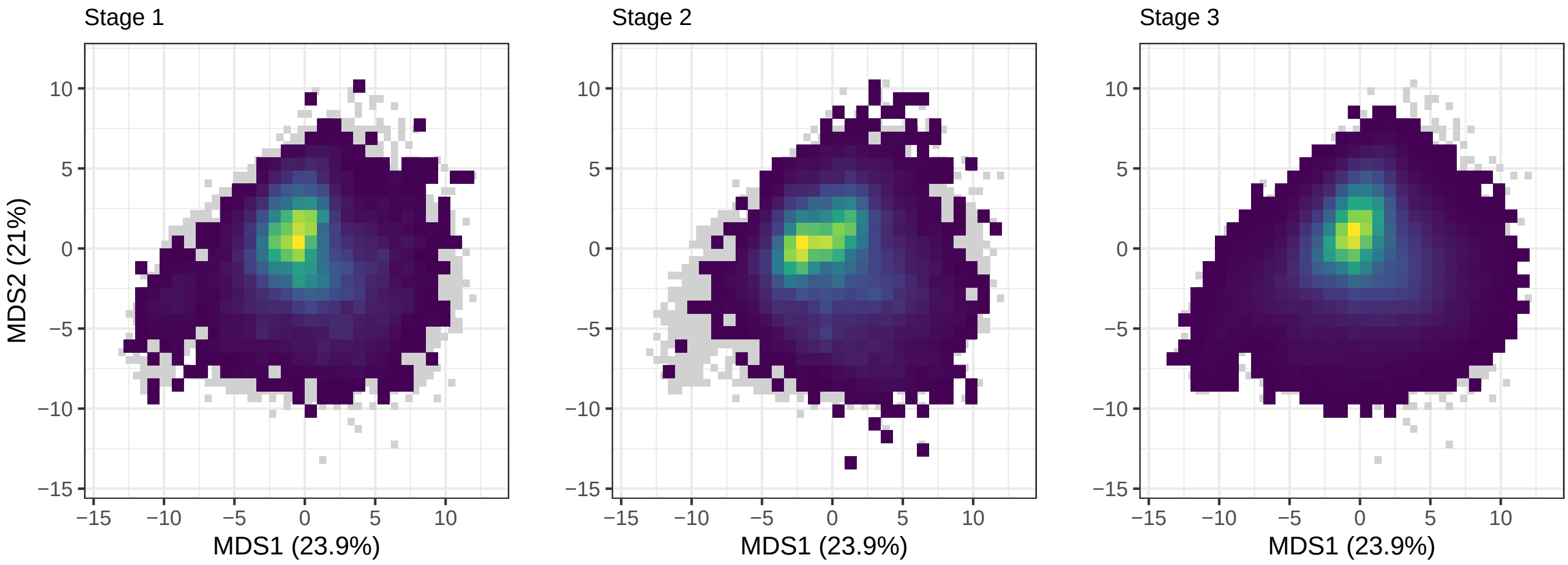

Figure S8. Alternate ordination plot. Related to Figure 1

This figure presents the results of a principal coordinates analysis of samples stratified by the three IBD epidemiologic stages. The layout is analogous to that of Figure 1I but is rendered at higher resolution (a 50-by-50 grid rather than a 30-by-30 grid). Patterns along the first and second MDS axes are shown. Each point represents an individual sample, and each panel includes only samples from a single stage. To facilitate evaluation of regions with substantial point overlap, a heatmap representation is used instead of a scatter plot: yellow areas indicate regions of higher sample density, whereas deep purple areas denote regions with fewer (but non-zero) samples. The gray shading overlaid on each panel indicates the area occupied by all samples across all stages.

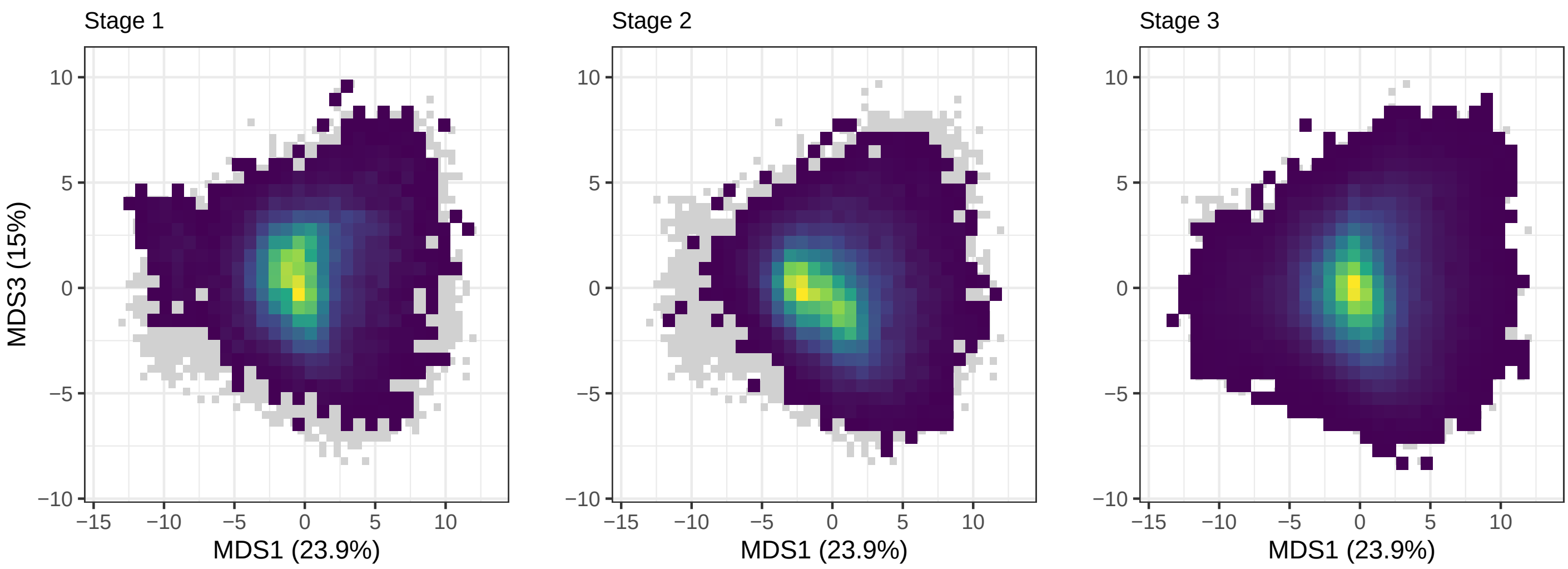

Figure S9. Alternate ordination plot. Related to Figure 1

Similar to Supplementary Figure 8,but showing axes MDS 1vs MDS3.

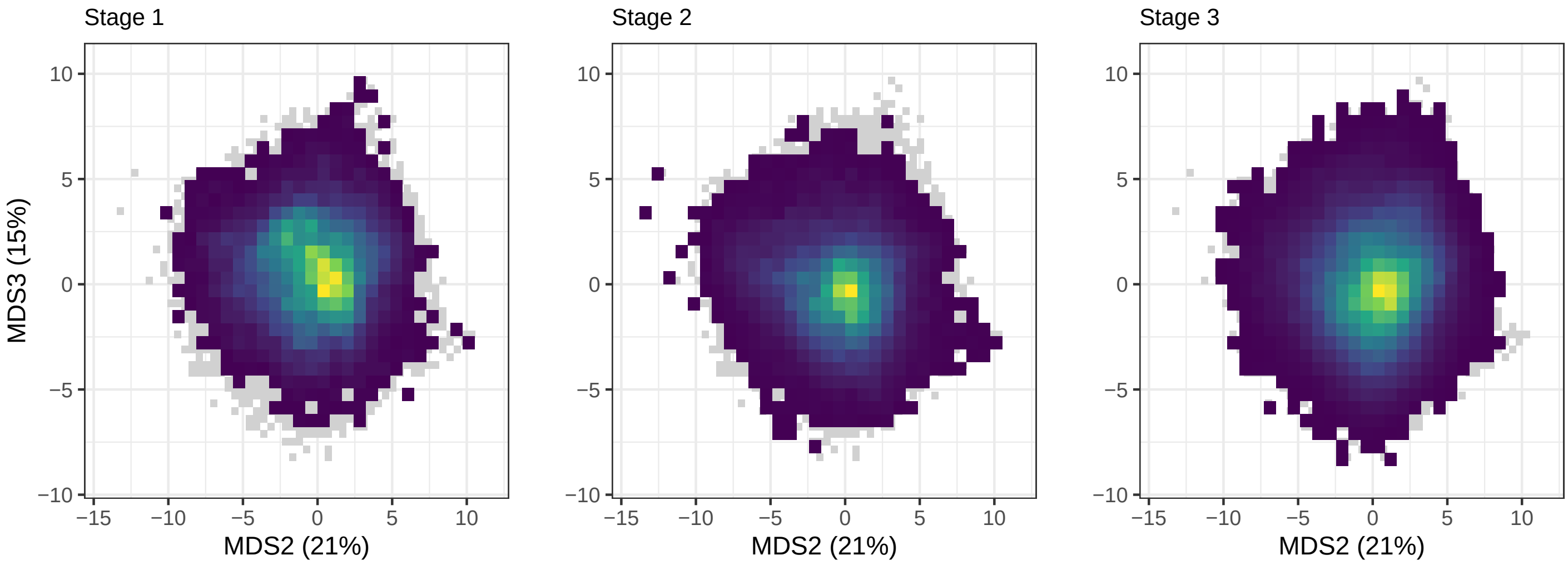

Figure S10. Alternate ordination plot. Related to Figure 1

Similar to Supplementary Figure 8,but showing axes MDS 2vs MDS3.

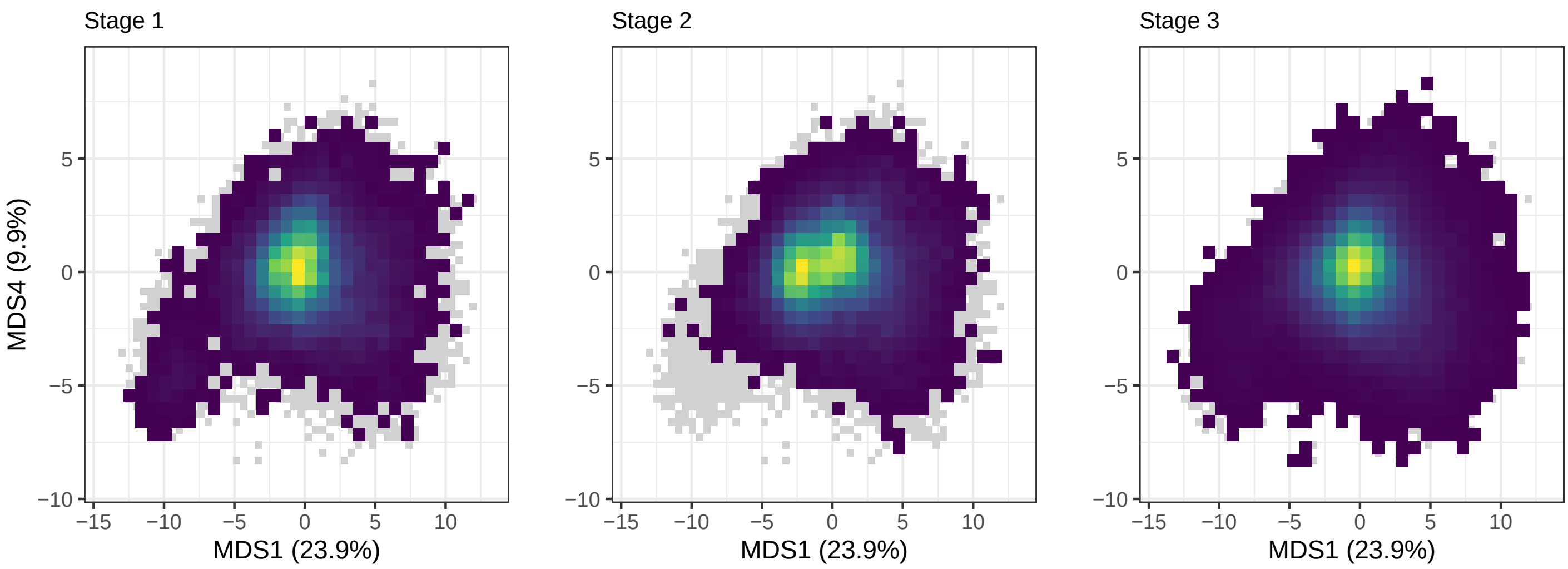

Figure S11. Alternate ordination plot. Related to Figure 1

Similar to Supplementary Figure 8,but showing axes MDS 1vs MDS4.

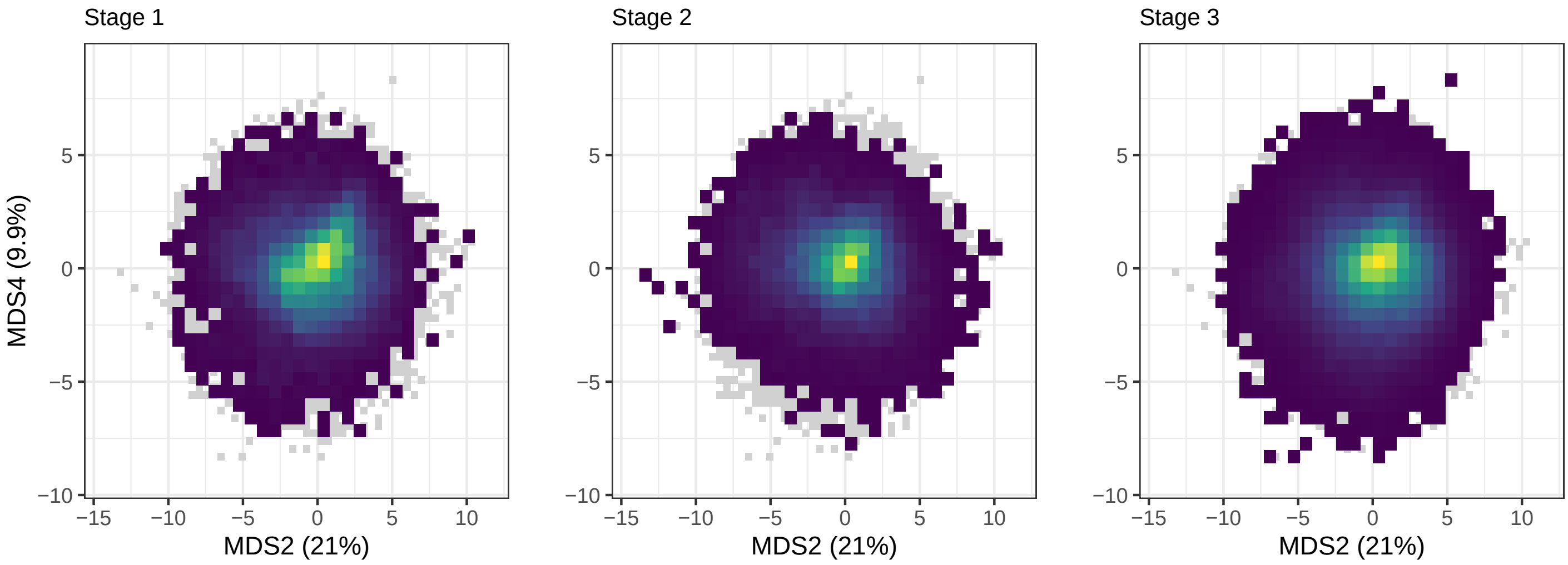

Figure S12. Alternate ordination plot. Related to Figure 1

Similar to Supplementary Figure 8,but showing axes MDS 2vs MDS4.

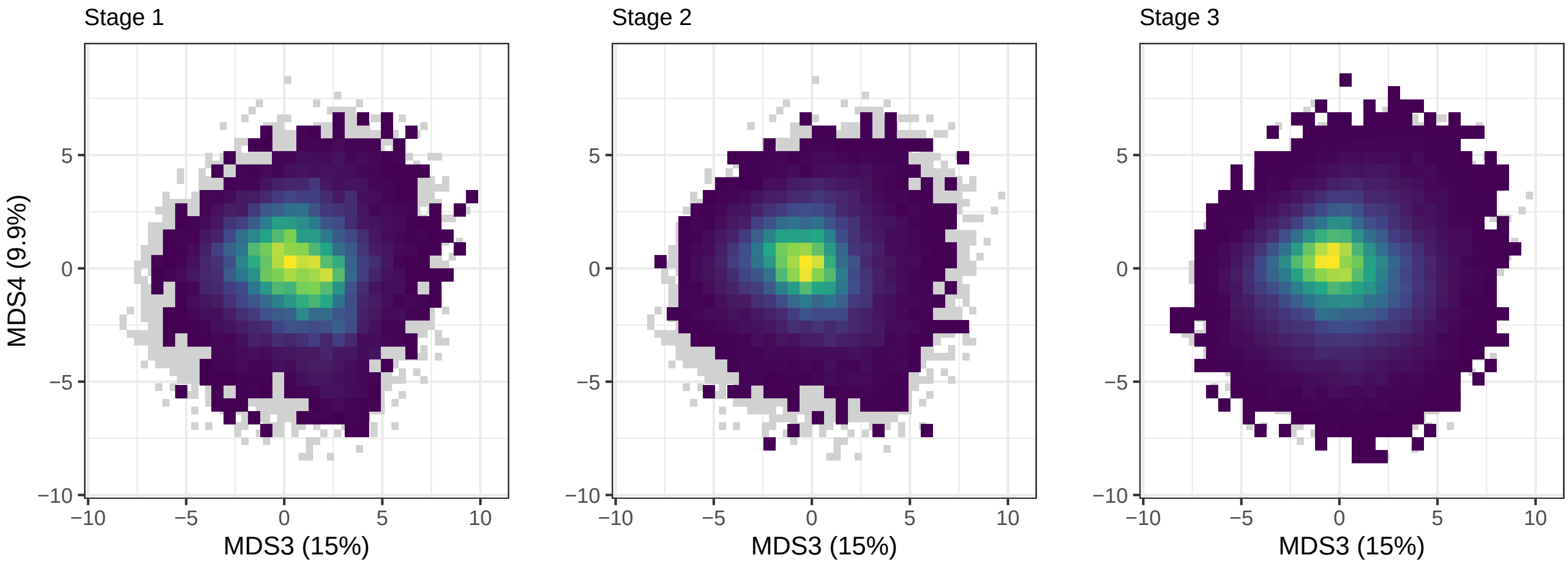

Figure S13. Alternate ordination plot. Related to Figure 1

Similar to Supplementary Figure 8,but showing axes MDS 3vs MDS4.

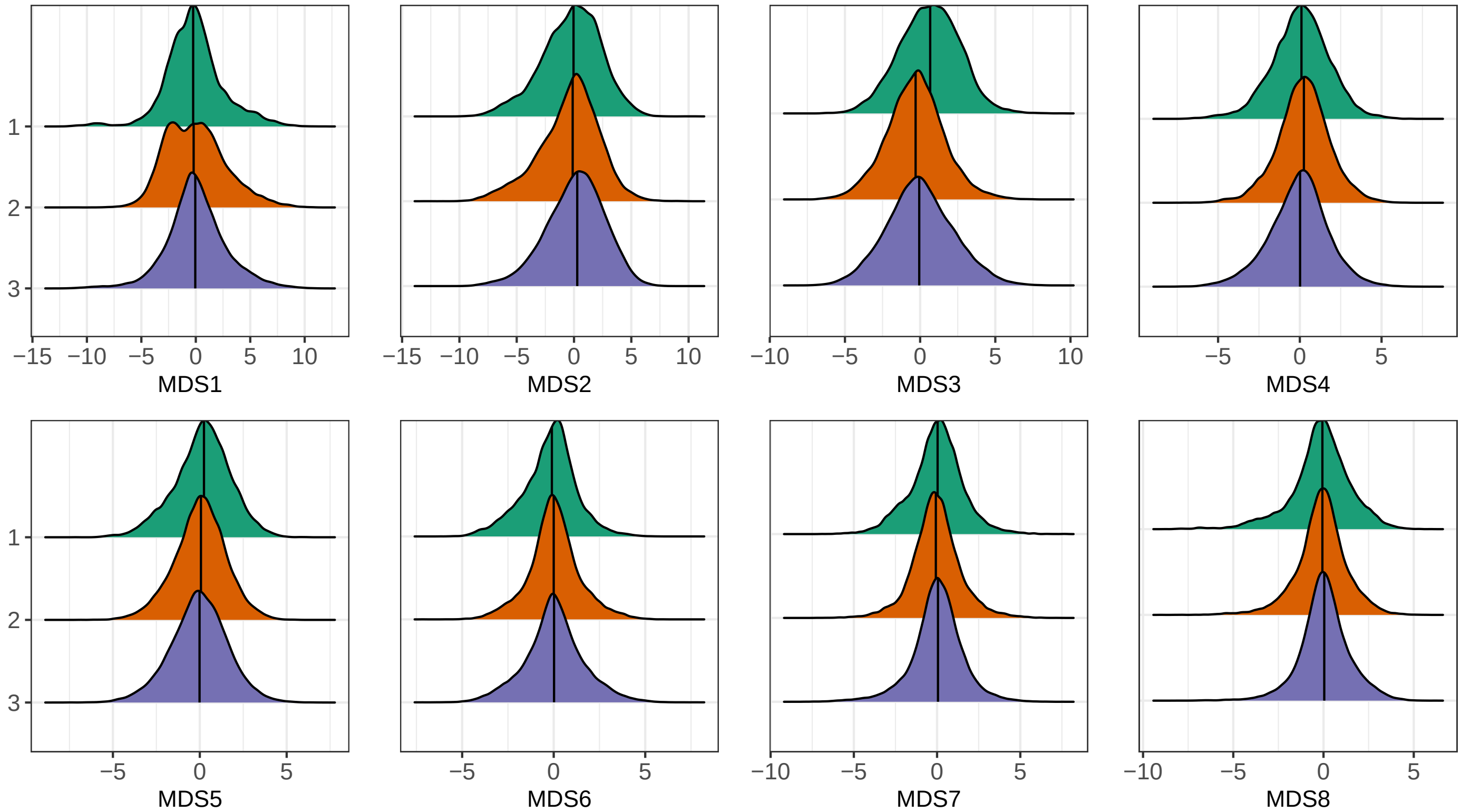

Figure S14. Ordination results. Related to Figure 1

This figure comprises a series of panels illustrating the results of a principal coordinates analysis of samples stratified by the three IBD epidemiologic stages. This analysis extends the data shown in Figure 1J by displaying factors MDS1 through MDS8. Each panel depicts a single factor; the x-axis indicates the value of that factor, and the y-axis represents the relative frequency of that value within the corresponding region.

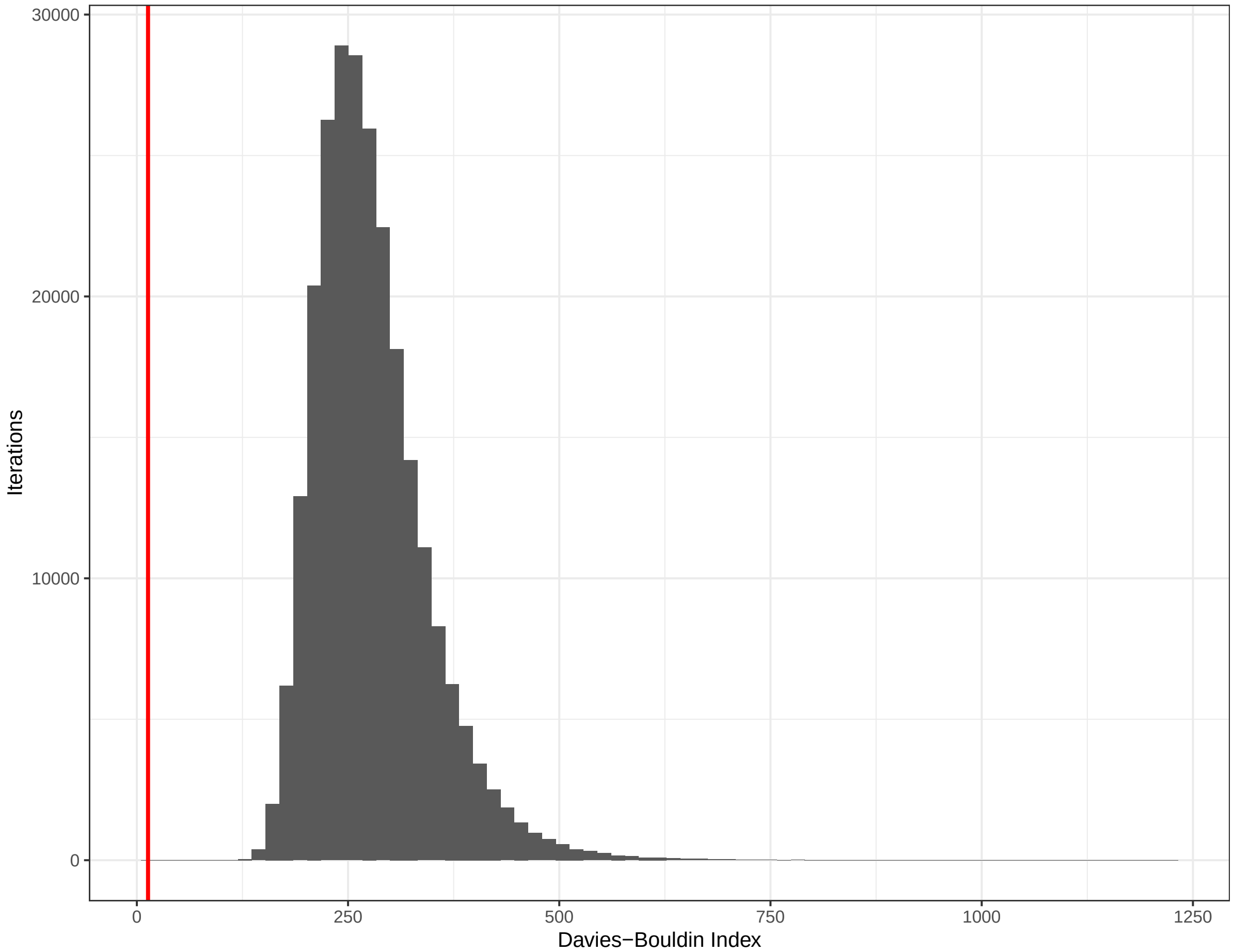

Figure S15. Cluster strength analysis. Related to Figure 1

This histogram presents the results of a bootstrap analysis assessing the strength of clustering by the three IBD epidemiologic stages in ordination space. In each iteration (250,000 in total), the regional labels corresponding to stages 1, 2, and 3 were randomly permuted across all samples, and a single score (the DaviesCBouldin index) was calculated for that iteration. The x-axis indicates the score, and the y-axis denotes the number of iterations falling within each bin. The red vertical line indicates the clustering score (13.26) obtained using the true stage assignments.

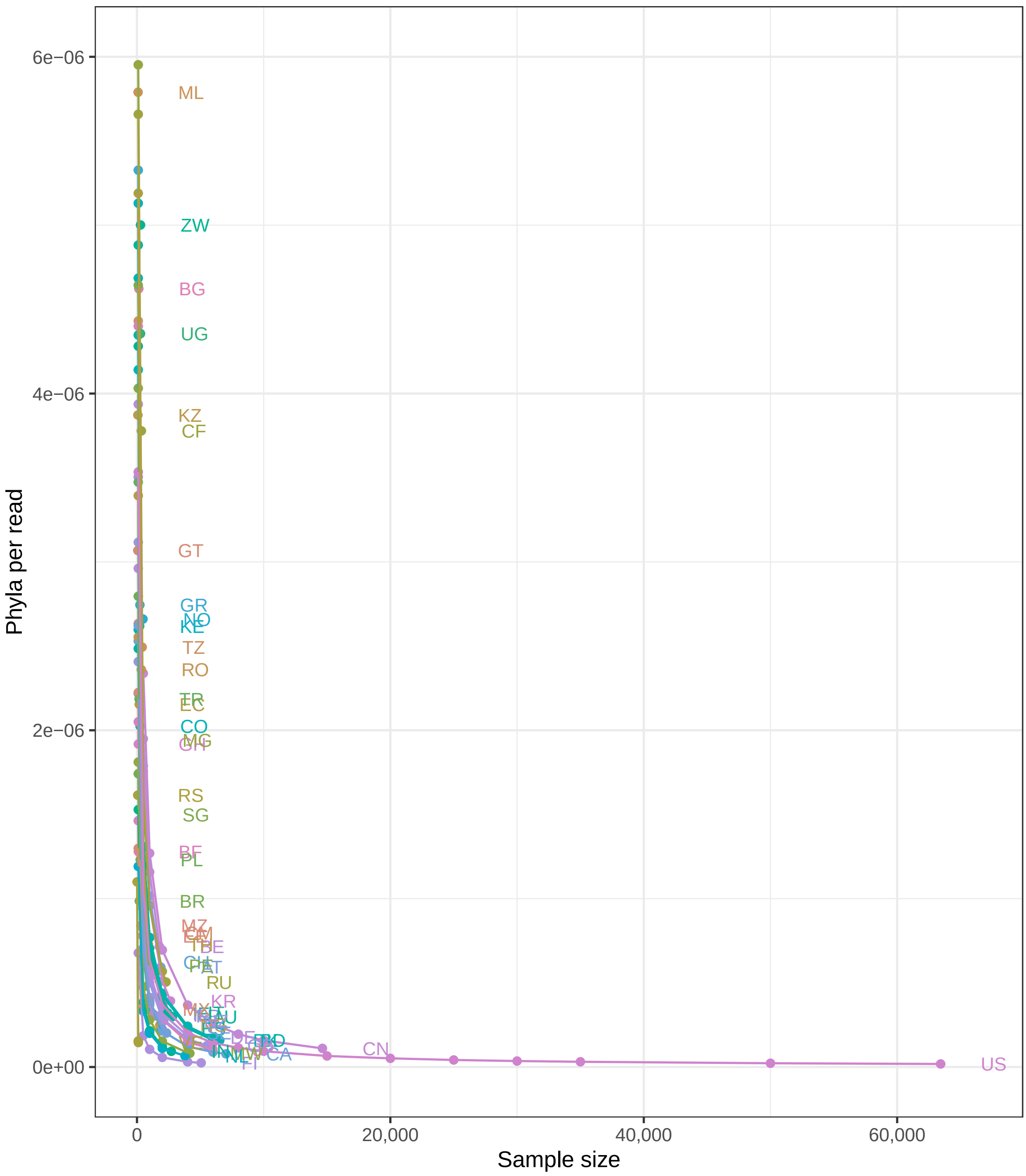

Figure S16. Discovery curves at the country level across 70 countries. Related to Figure 2

The x-axis indicates the number of samples, and the y-axis denotes the number of phyla per read (phylum per read). Similar to the inset in Figure 2A, increasing numbers of microbiome samples were progressively subsampled from each country. For each sample size (shown on the x-axis), we calculated the number of unique phyla observed and the number of sequencing reads per sample. We then computed the mean phyla per read (y-axis) by dividing the mean number of unique phyla by the mean read count for each sample size.

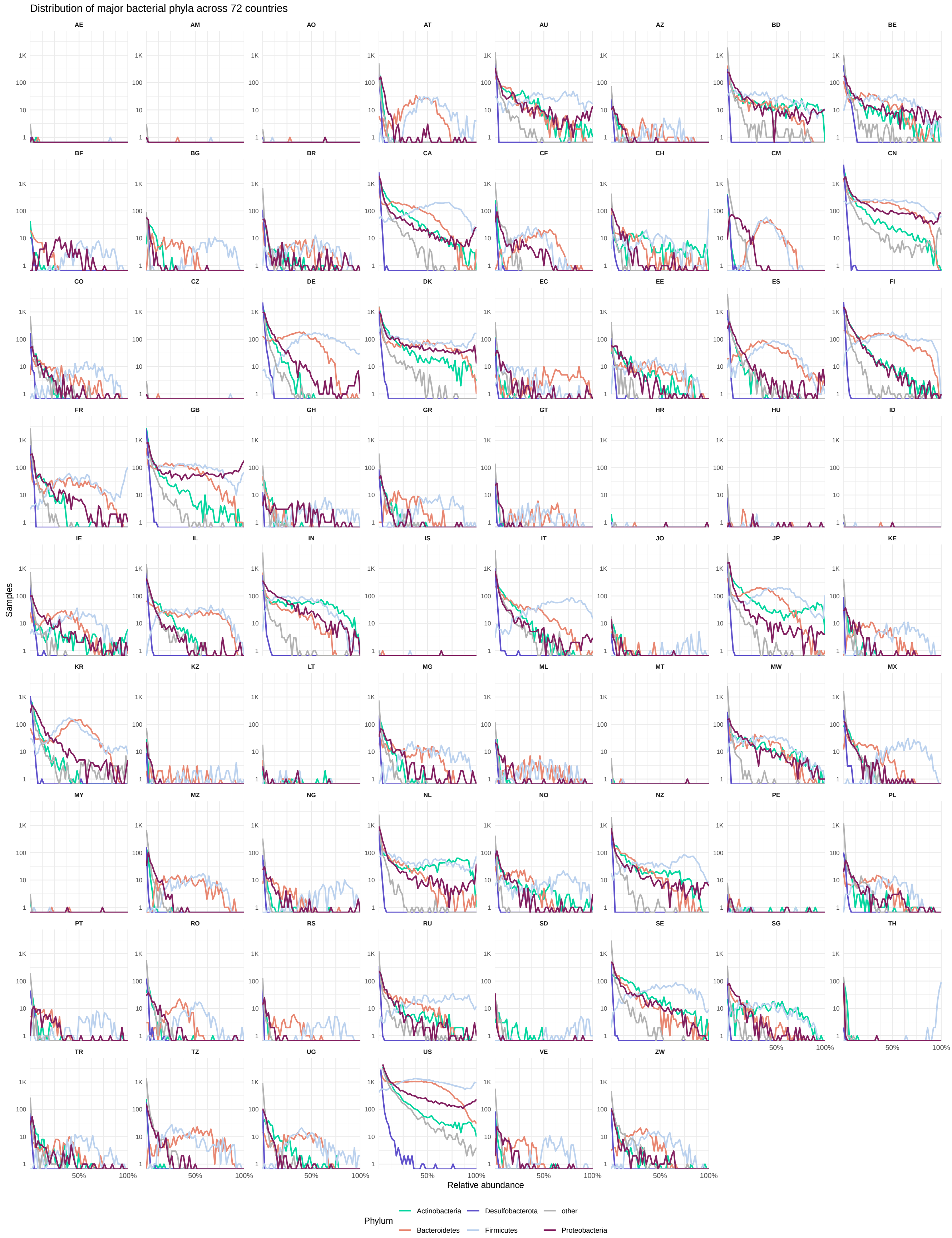

Figure S17. Relative abundance of dominant phyla. Related to Figure 2

This figure illustrates the distribution of relative abundances of the most prevalent phyla in the compendium. Each panel visualizes all samples from a single country. The x-axis indicates the relative abundance of each phylum, and the y-axis denotes the number of samples exhibiting the corresponding relative abundance. Each line represents a single phylum and is distinguished by color.

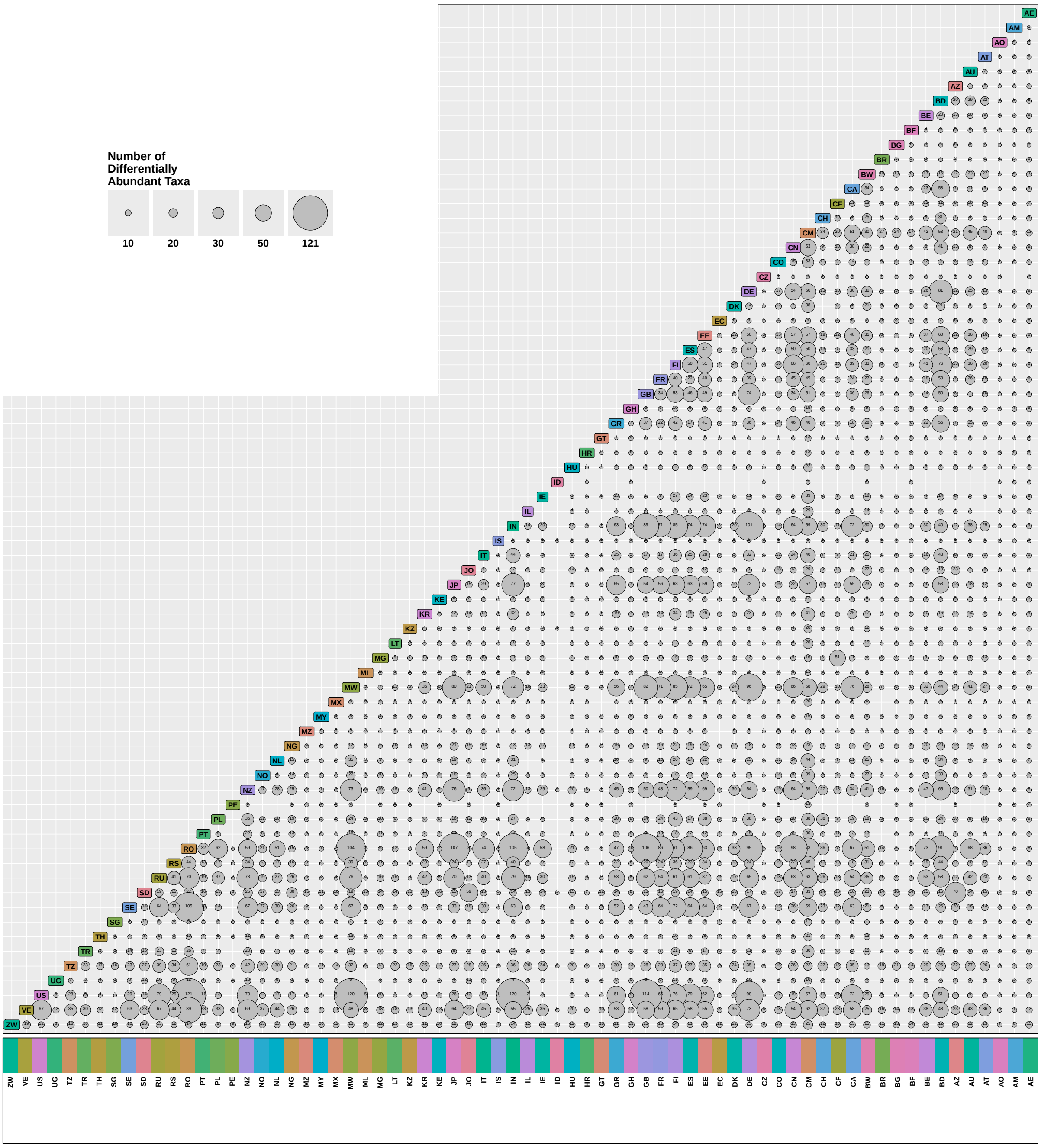

Figure S18. Taxa are differentially abundant between countries. Related to Figure 3

203 taxa were selected to test for differential abundance between countries. The x-and y- axes are each colored by country; at each intersection, the size of the circle and the number below it indicate the number of taxa that were significantly different between the two countries.

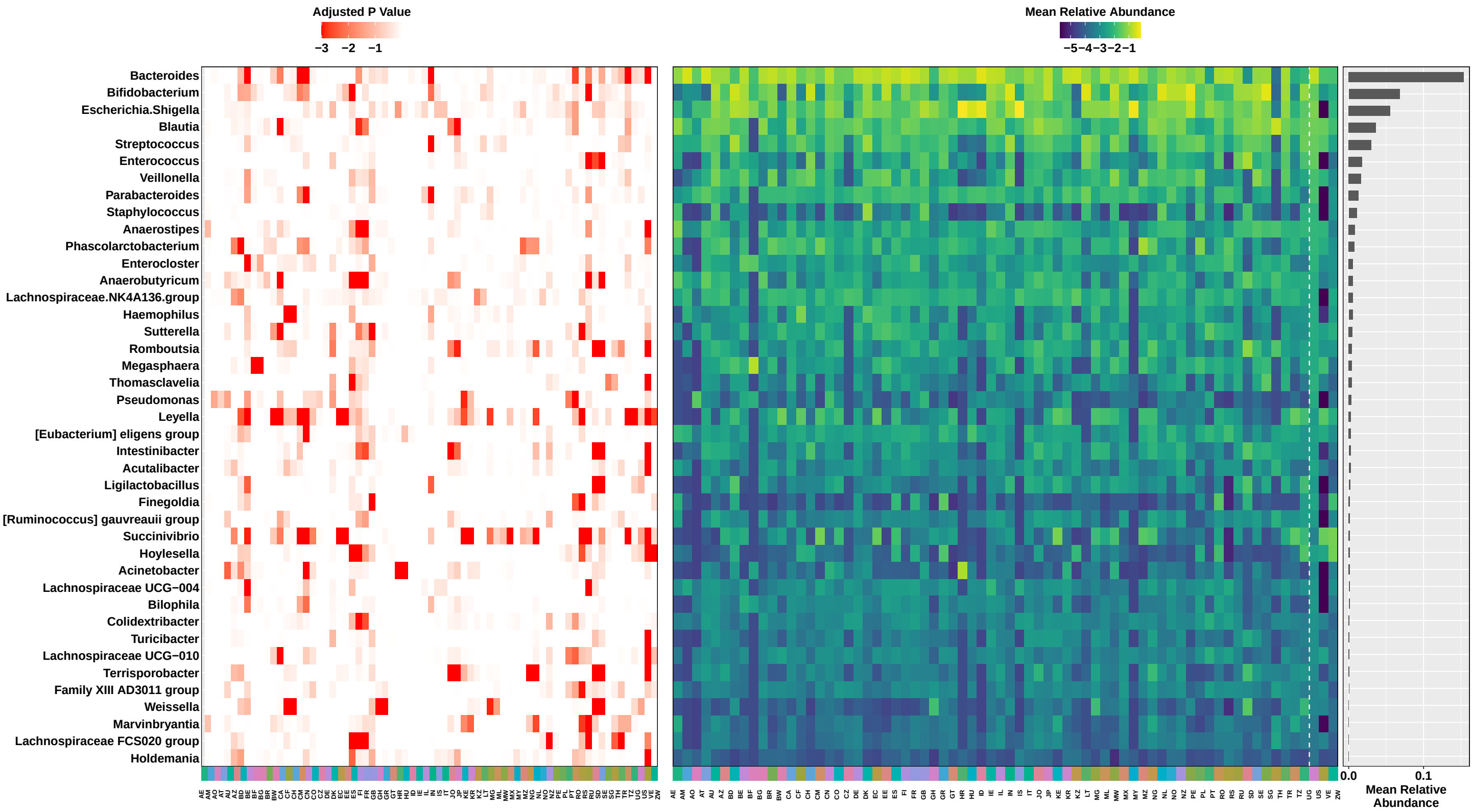

Figure S19. Taxa are differentially abundant between countries. Related to Figure 3

The red-white heat map illustrates adjusted p-values for country-wise differences when each country is compared to the United States. The y-axis lists all evaluated genera, and the x-axis lists all countries (using the same color scale as in Supplementary Figure 18). Each cell represents the strength of the differential abundance result for that taxon. The blue-green heat map depicts the mean relative abundance (log10) of each taxon in each country, as indicated on the x-axis. The accompanying bar chart shows the mean relative abundance of each taxon averaged across all countries.

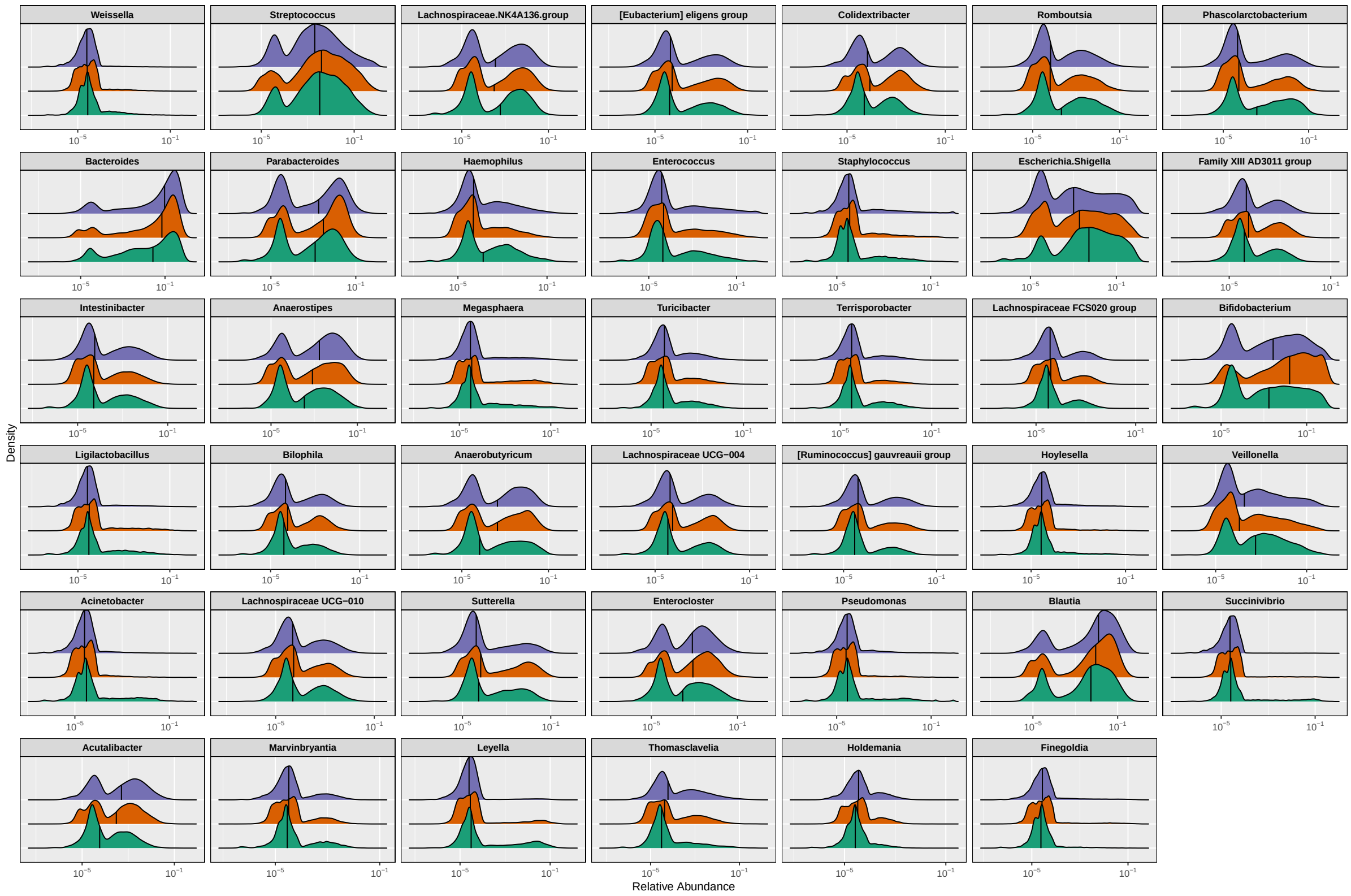

Figure S20. Relative abundance of differentially abundant taxa across the three IBD stages.Related to Figure 3

Similar to Figure 3C, each panel illustrates the relative abundance of a single taxon. Shown here are all taxa ranke 1-41 by average relative abundance. Within each panel, colored areas represent the distributions of samples from a single IBD stage. Thex-axis indicates the relative abundance of the specified taxon (log10-transformed), and the y-axis denotes the relative frequency with which that abundance is observed within the corresponding stage. Black vertical lines indicate the median.

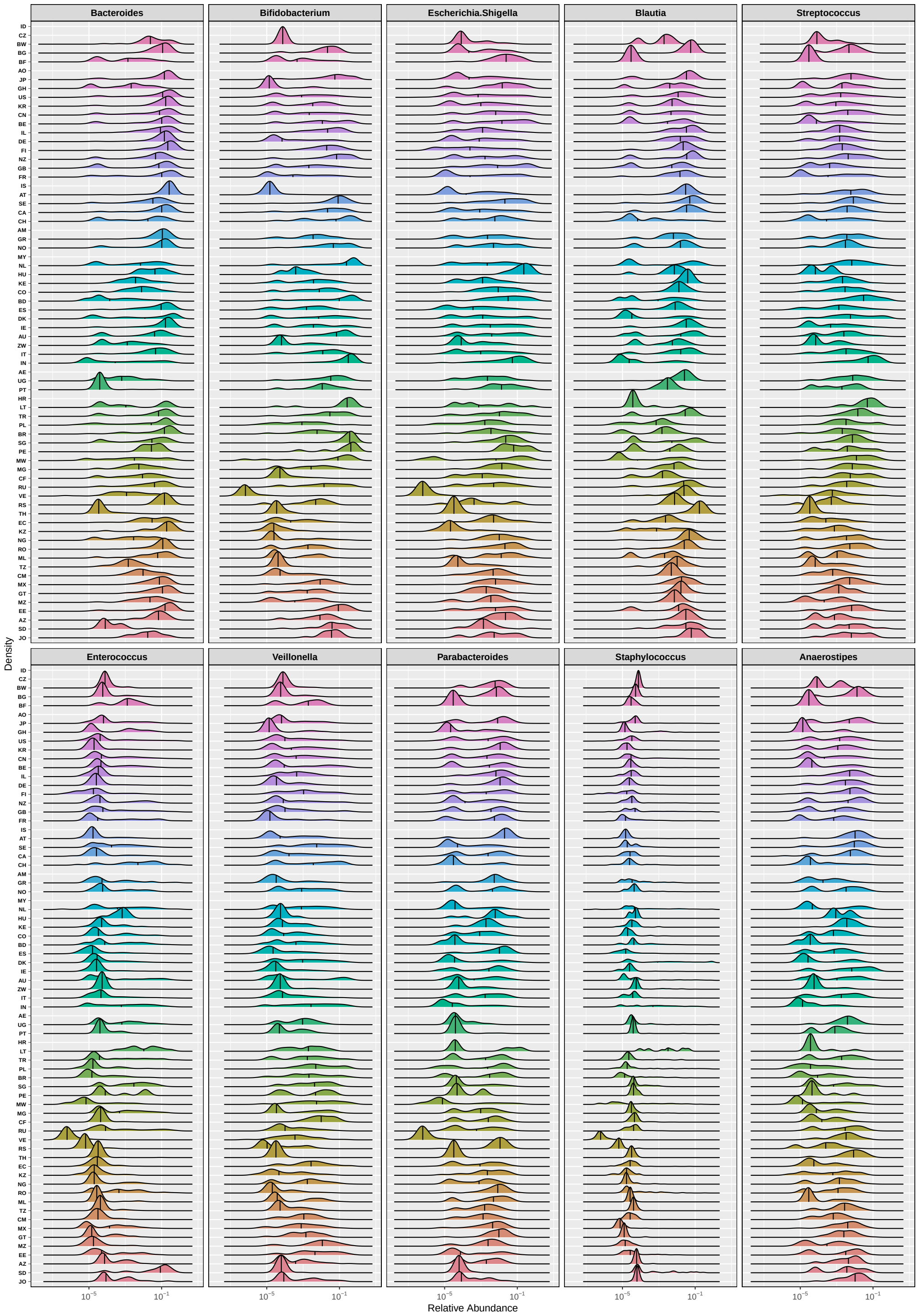

Figure S21. Taxa are differentially abundant between countries. Related to Figure 3

Each panel illustrates the relative abundance (log10) of one of the ten most abundant taxa. Each colored area indicates the distribution of samples from an individual country, using the same color scheme as SF18. The x-axis represents the (log10) relative abundance of the specified genus, and the y-axis shows the relative frequency with which that abundance is observed in the specified country. Black vertical lines indicate the median values.

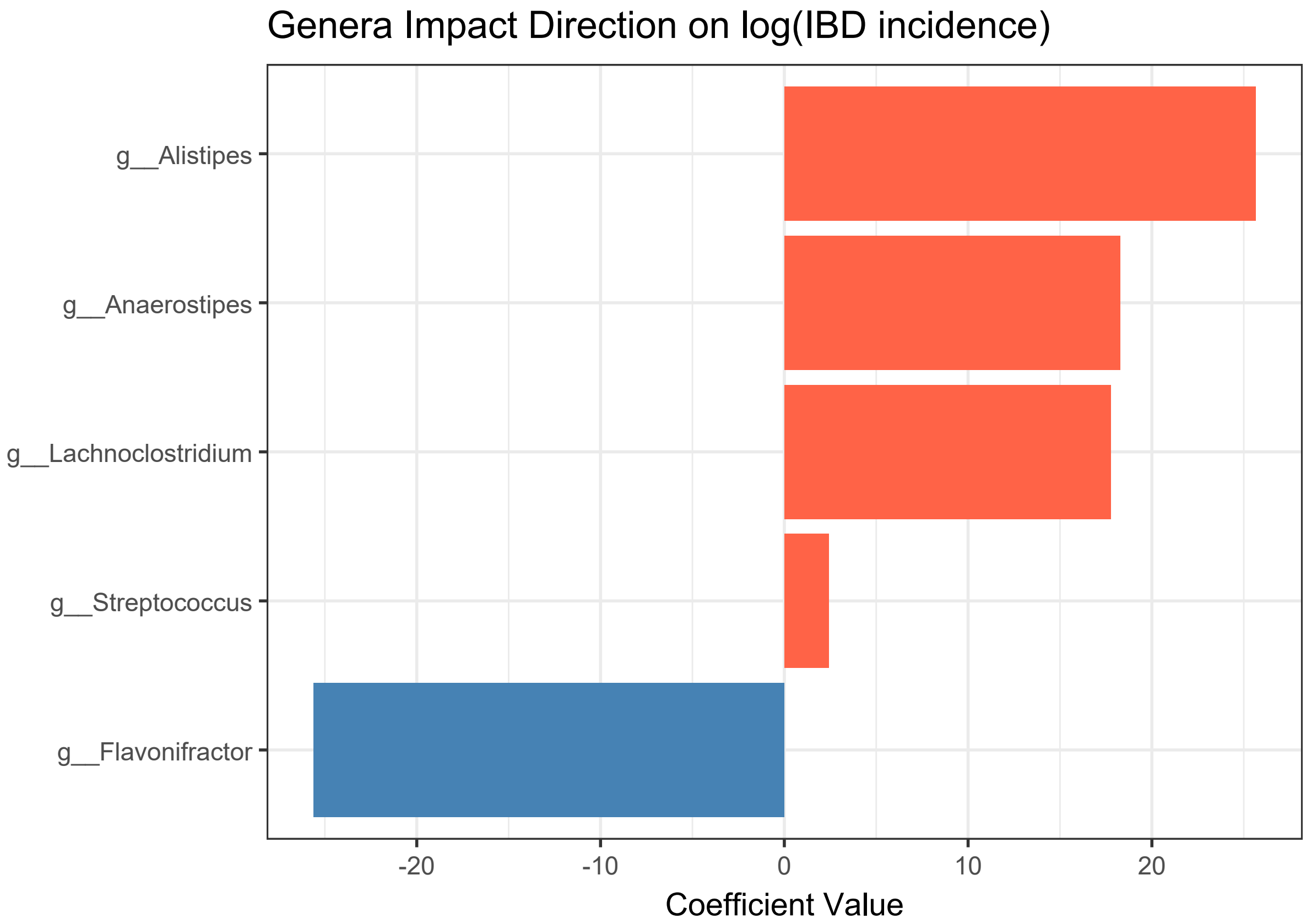

Figure S22. Direction and magnitude of genus-level effects on IBD incidence. Related to Figure 6

This figure shows the direction and magnitude of effects of core microbial genera on national IBD incidence, as estimated by a multivariable linear regression (MLR) model following LASSO-based feature selection. The analysis was performed using country-level mean relative abundances of genera from 70 countries, with the outcome modeled as log-transformed IBD incidence. The x-axis indicates the regression coefficient value, and the y-axis lists the genera retained in the final model. Positive coefficients (red bars) indicate genera whose higher relative abundance is associated with increased IBD incidence, whereas negative coefficients (blue bars) indicate genera associated with lower incidence. The absolute value of each coefficient reflects the strength of its contribution to the model.

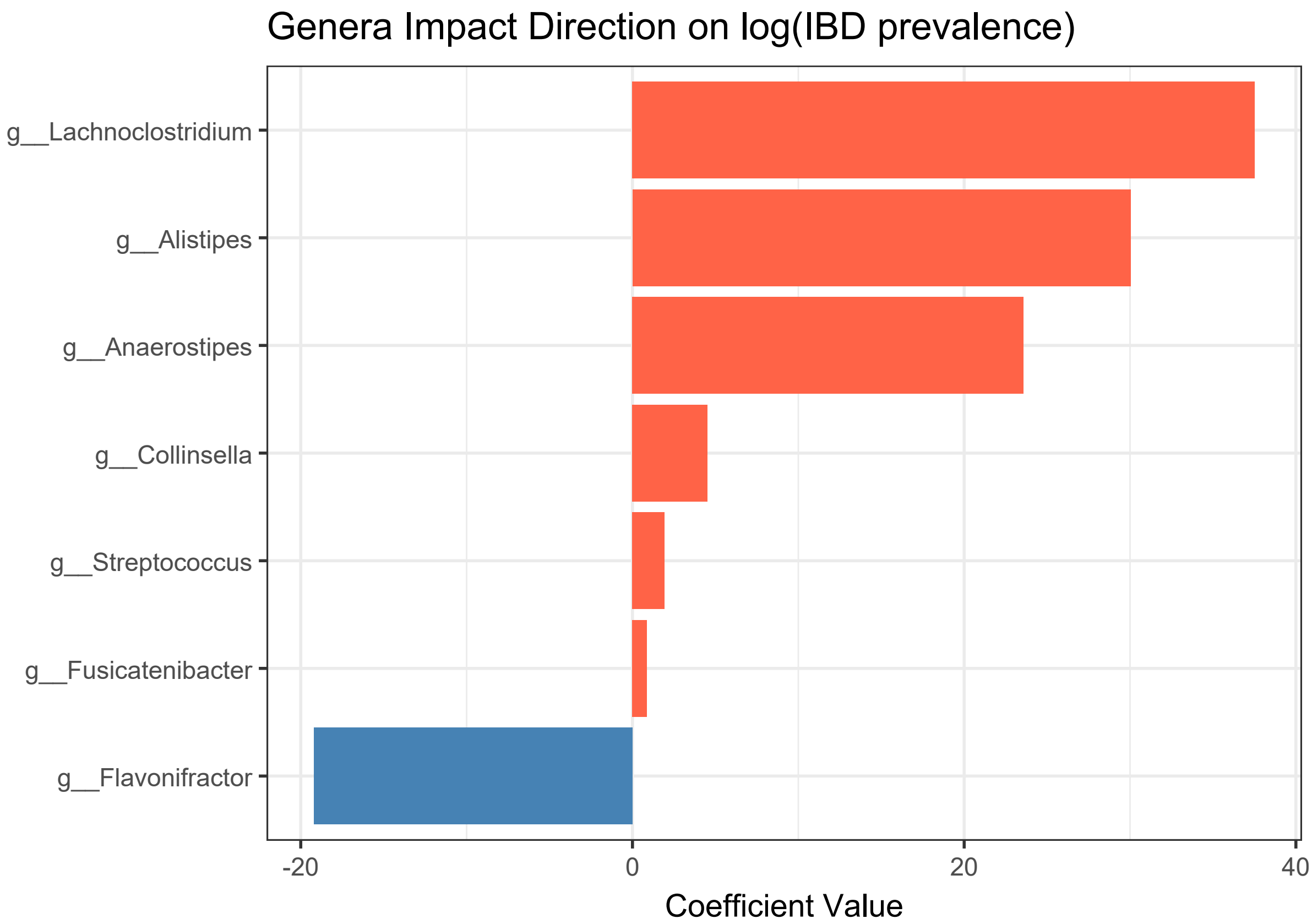

Figure S23. Direction and magnitude of genus-level effects on IBD prevalence. Related to Figure 6

Using the same LASSO-MLR framework as in Supplementary Figure 22, this figure illustrates the effects of selected microbial genera on national IBD prevalence (log-transformed). Country-level mean relative abundances were used as predictors. The x-axis denotes regression coefficient values, and the y-axis lists genera included in the final model. Positive coefficients (red bars) represent genera positively associated with higher IBD prevalence, while negative coefficients (blue bars) represent genera inversely associated with prevalence. Larger absolute coefficient values indicate stronger predictive contributions to the prevalence model.

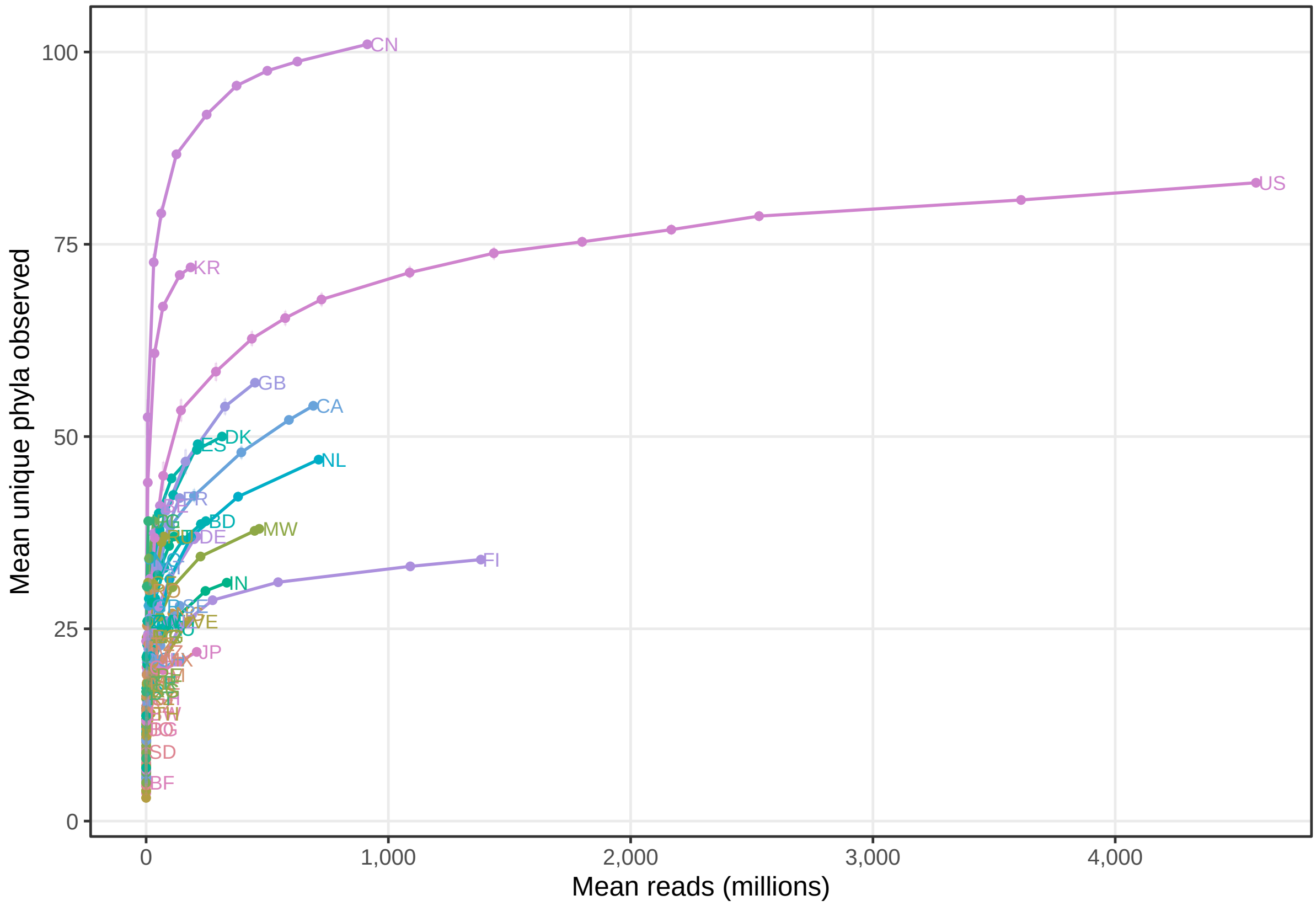

Figure S24. Country-level discovery curves. Related to Figure 2

This figure illustrates the number of phyla discovered as the number of sequencing reads increases. Using the same sampling scheme as in Figure 2A, increasing numbers of microbiome samples were progressively subsampled from each country. For each sample size, we calculated the mean number of sequencing reads in the selected samples (x-axis) and the mean number of unique phyla observed in the selected samples (y-axis).

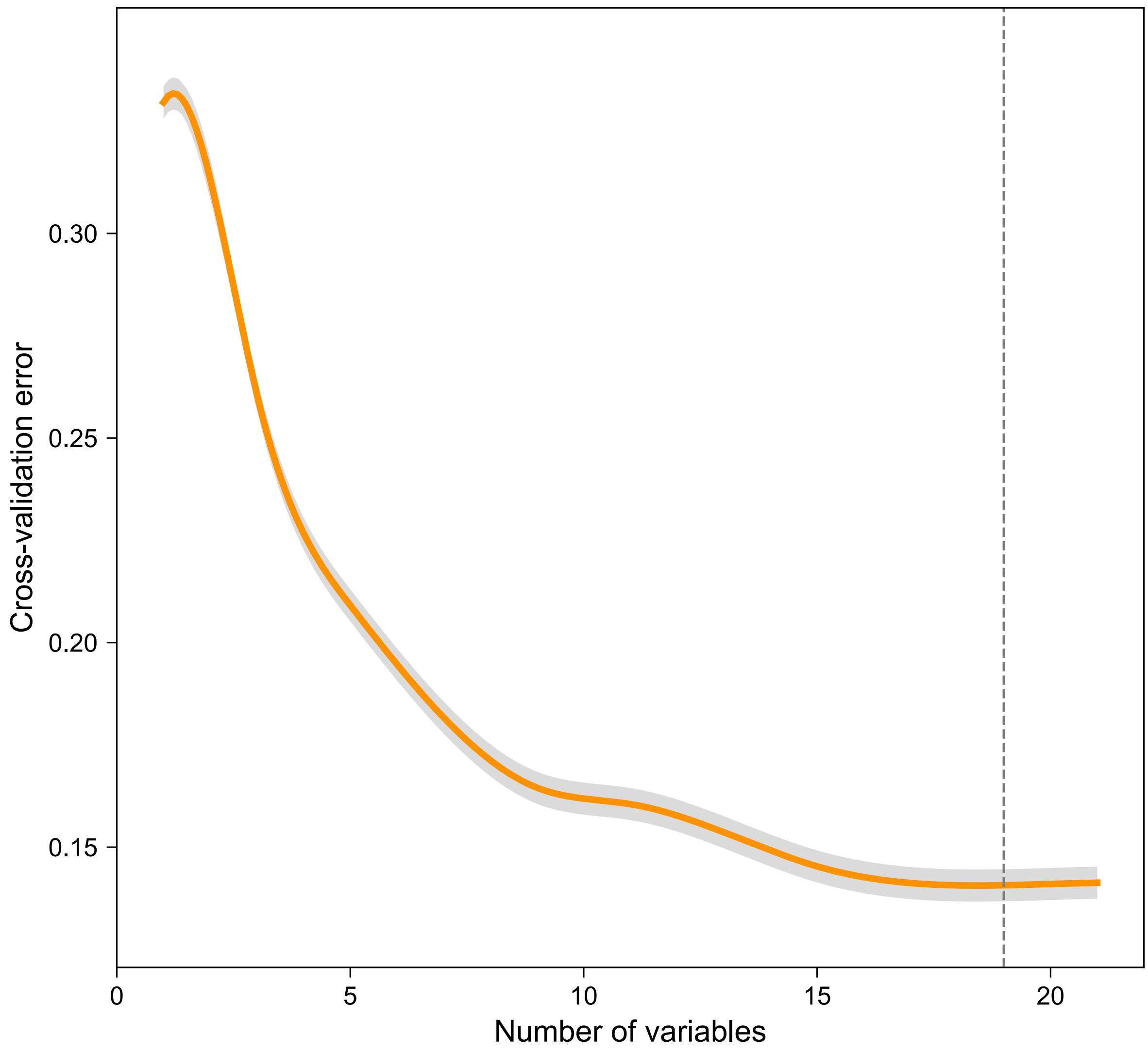

Figure S25. Feature selection and cross-validation of the random forest model for MIRS construction. Related to Figure 6

This plot shows the relationship between the number of microbial genera included in the random forest model and the corresponding cross-validation error. The x-axis indicates the number of variables (genera) ranked by importance based on mean decrease in accuracy, and the y-axis represents the tenfold cross-validation error. The orange line denotes the mean cross-validation error across folds, with the shaded area indicating the associated variability. The dashed vertical line marks the selected model incorporating the top 19 genera, which achieved a near-minimal cross-validation error and was subsequently used to construct the Microbial Inflammatory Risk Score (MIRS).

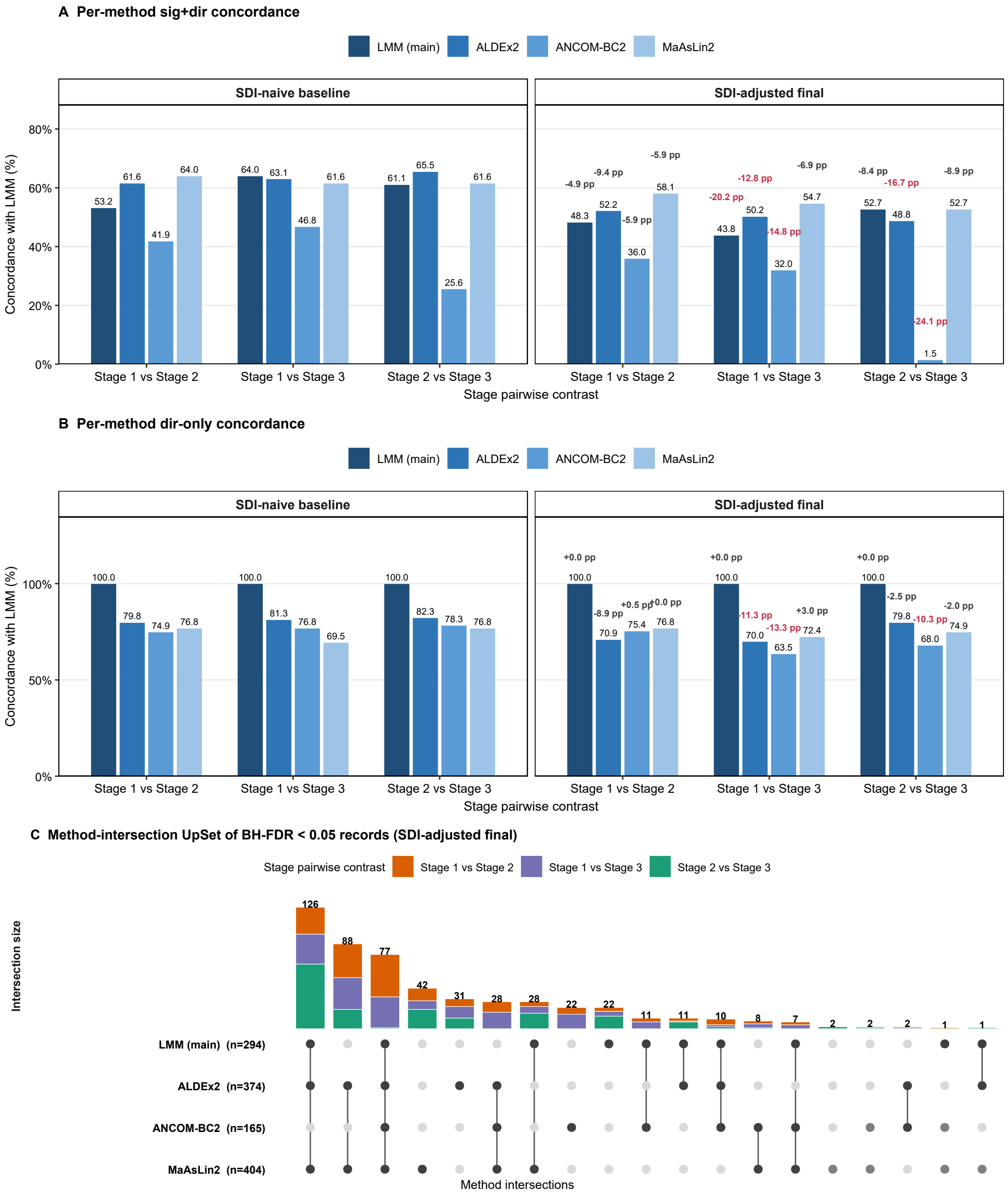

Figure S26.Sig+diranddir-onlyconcordancecomparisonsusing16Samplicondata. Related to Figure 3

The sig+dir % and dir-only % of four differential abundance (DA) analyses (two-sided) are shown in A and B, respectively. The reference direction is set to be the LMM main analysis. The Y-axis denotes patterns of percentage of LMM-concordant taxa. Numbers above bars are per-cell percentage values. By default, all four DA methods implement the BH procedure to adjust for multiple comparisons. Color and the name of the corresponding DA method are shown at the top within the graph. Two cohort-confounding scenarios are considered: SDI-naive baseline (no SDI covariate), as well as SDI-adjusted final (SDI as continuous covariate); number of modelled taxa = 203. Panel C shows the four-method UpSet of BH-FDR < 0.05 records under SDI-adjusted, stacked by stage pairwise contrast; the largest combination is LMM + ALDEx2 + MaAsLin2 (126 records) and the four- method full consensus is 77 records.

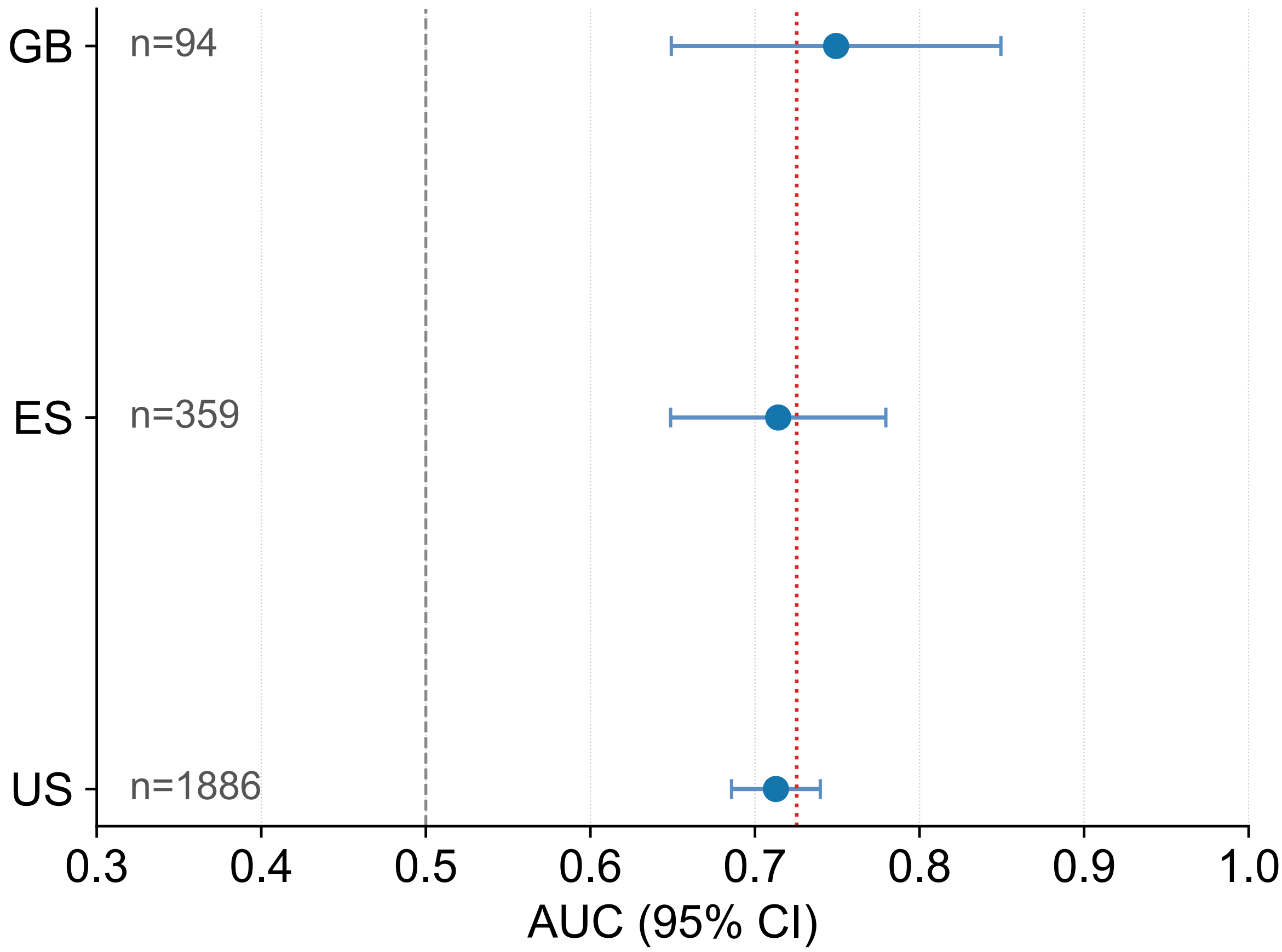

Figure S27. Leave-one-country-out cross-cohort generalisation of the IBD random forest classifier. Related to Figure 6

Forest plot showing per-country AUC of the 19-genera random forest classifier when one country at a time is held out from the metagenomic training pool. The x-axis denotes AUC with 95% confidence intervals, and the y-axis lists evaluable held-out countries by ISO alpha-2 code. The dashed vertical line marks AUC = 0.5; the dotted vertical line marks the mean AUC across evaluable countries.

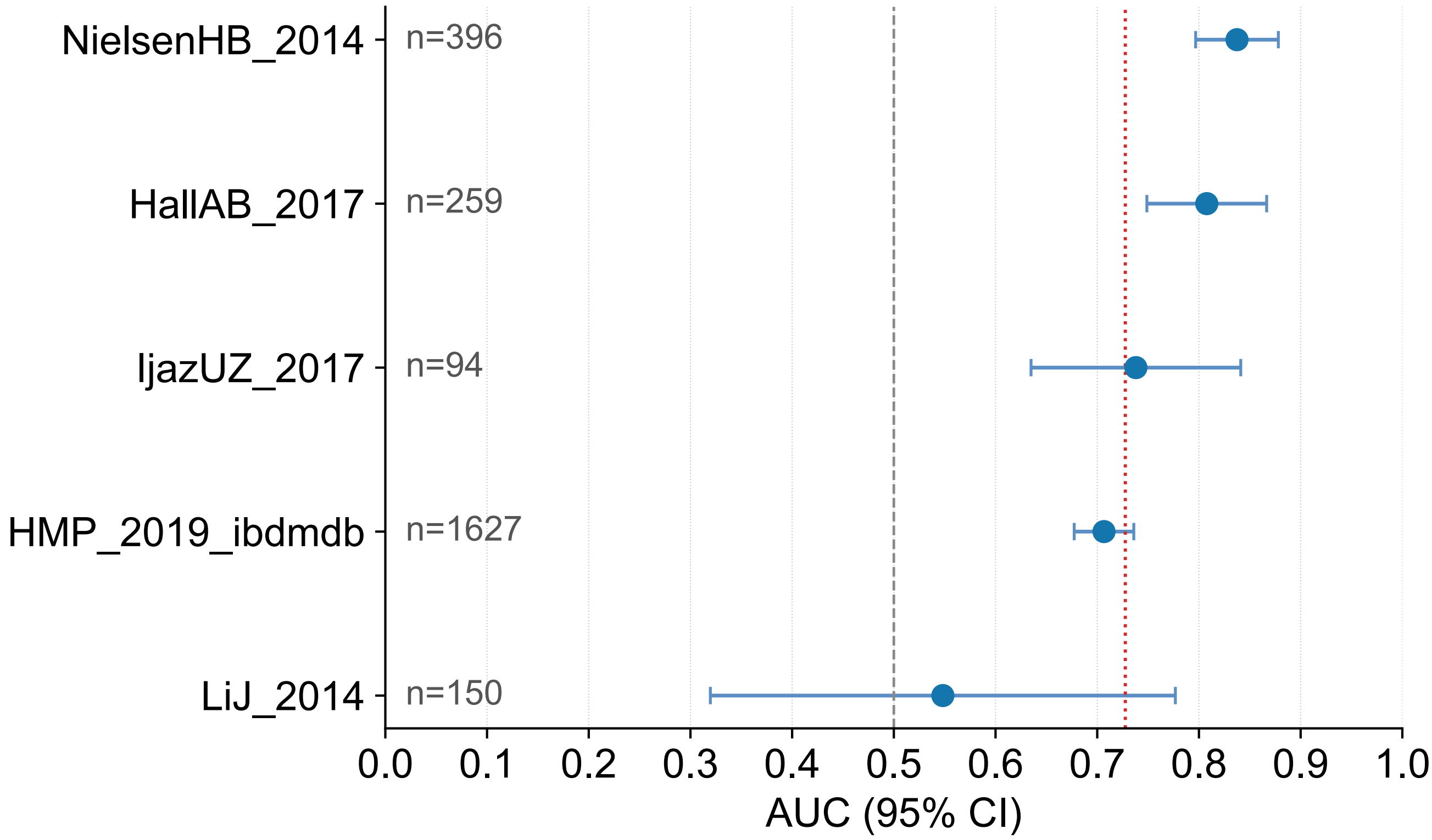

Figure S28. Leave-one-BioProject-out cross-cohort generalisation of the IBD random forest classifier. Related to Figure 6

Forest plot showing per-BioProject AUC of the 19-genera random forest classifier when one BioProject at a time is held out from the metagenomic training pool. The x-axis denotes AUC with 95% confidence intervals, and the y-axis lists evaluable held-out BioProjects. The dashed vertical line marks AUC = 0.5; the dotted vertical line marks the mean AUC across evaluable BioProjects.

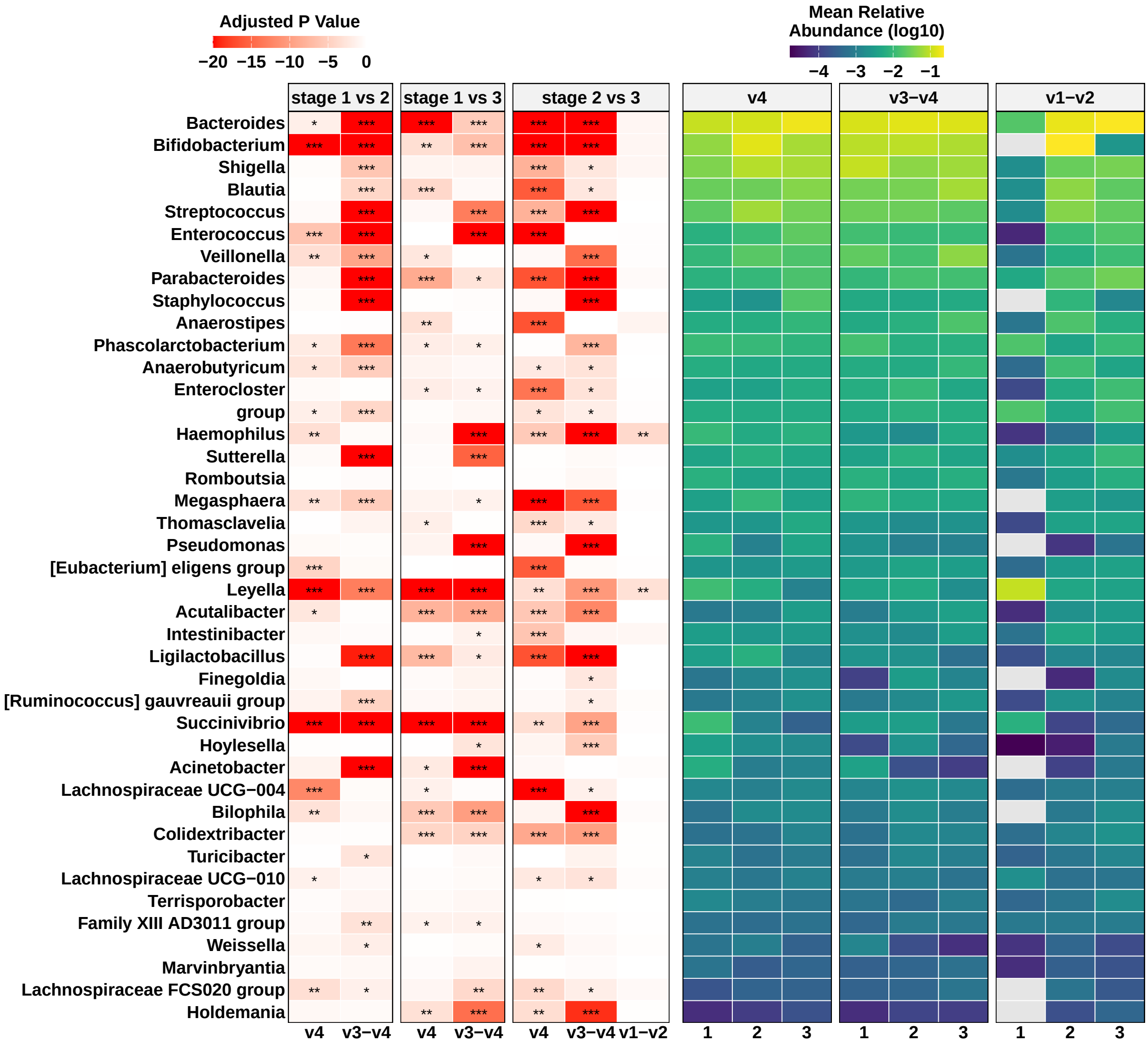

Figure S29. Taxa are differentially abundant between IBD stage-associated regions across V-region subsets. Related to Figure 3

The red-white heat map illustrates adjusted p-values for stage-wise differences within three V-region subsets. The y-axis lists the 41 taxa flagged by all four methods (LMM, ALDEx2, ANCOM-BC2, MaAsLin2; 0-4) as significant (BH-FDR < 0.05) and direction-concordant with LMM, and the x-axis lists V-region subsets within three stage-pairwise- contrast panels (Stage 1 vs. Stage 2, Stage 1 vs. Stage 3, Stage 2 vs. Stage 3). Each cell represents the strength of the differential abundance result for that taxon, with stars (*, **, ***) overlaying cells at BH-FDR < 0.05, 0.001, and 10^{-4}. The blue-green heatmap depicts the mean relative abundance (log10) of each taxon across the three V-region subsets, each stratified by stage (1, 2, 3) on the x-axis.

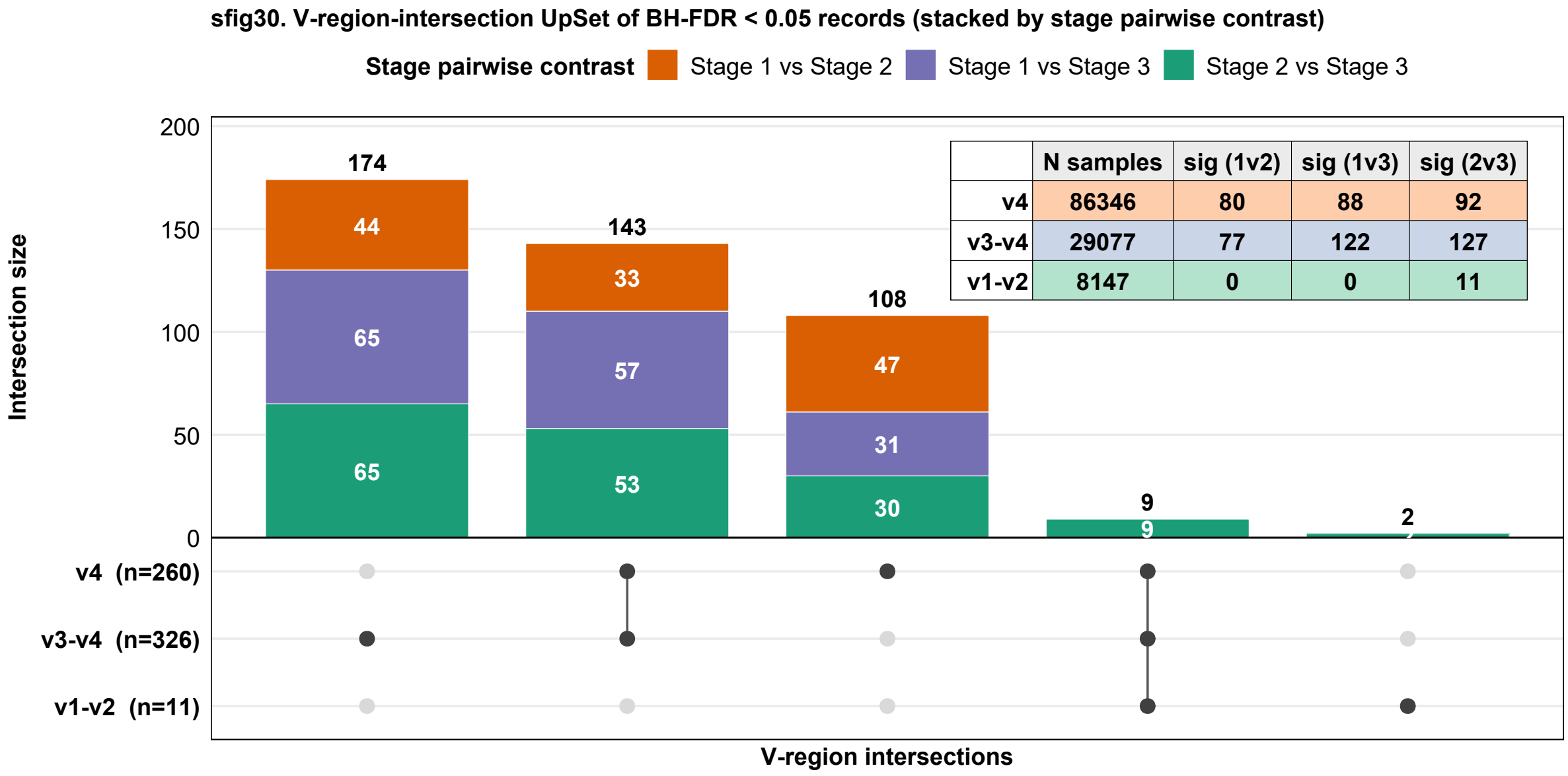

Figure S30. Cross-V-region consistency of BH-FDR- significant taxa across stage pairwise contrasts. Related to Figure 3

Overview of 436 BH-FDR < 0.05 records detected across three V-region subsets and three stage pairwise contrasts (Stage 1 vs. Stage 2, Stage 1 vs. Stage 3, Stage 2 vs. Stage 3), based on the per-subset LMM. Records show substantial cross-V-region consistency, with 58% (152/260) of v4 records also detected in v3-v4 and 82% (9/11) of v1-v2 records confirmed in both v4 and v3-v4.

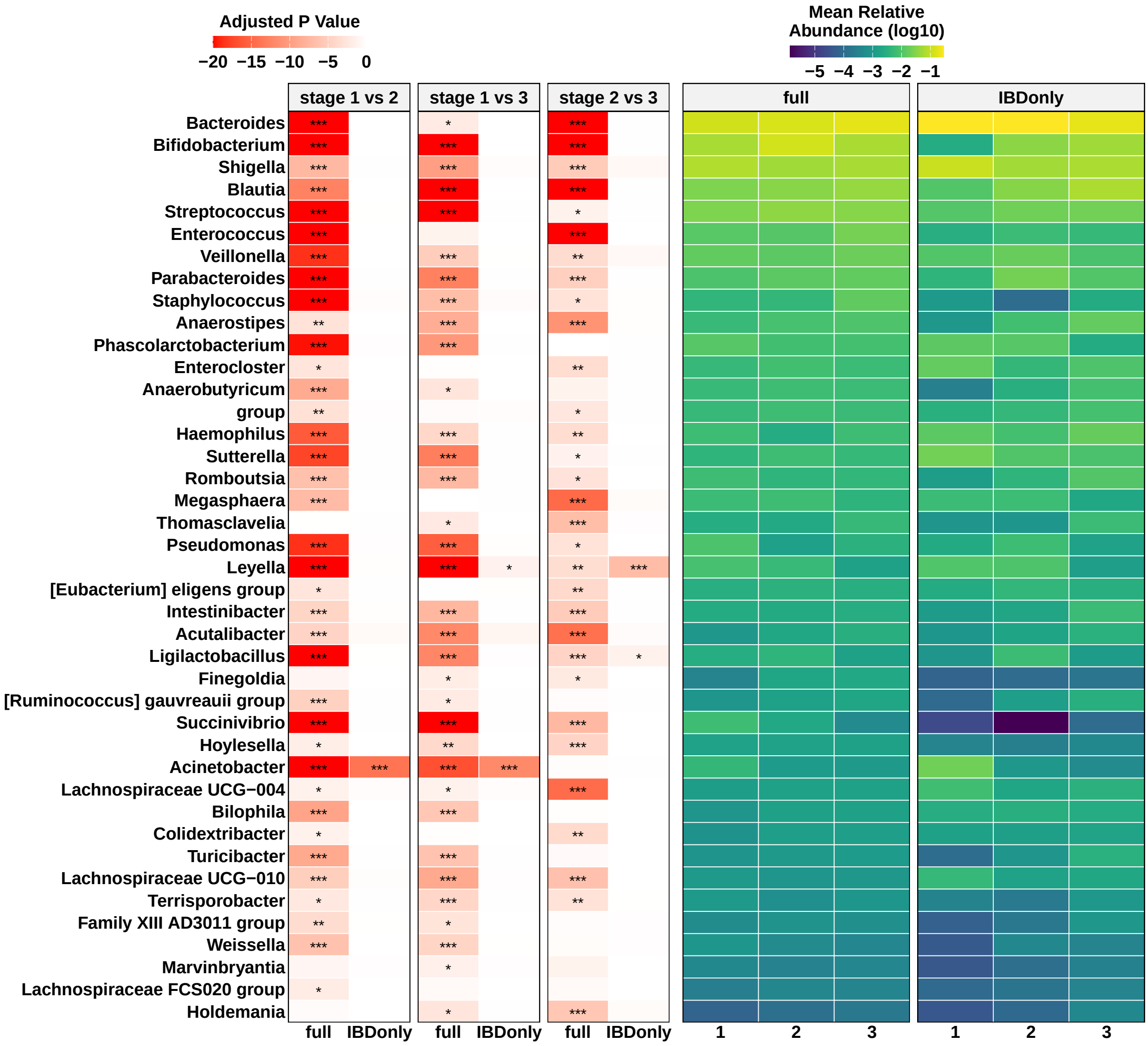

Figure S31. Taxa are differentially abundant between IBD stage-associated regions across both the full cohort and the single-disease IBD-only subcohort. Related to Figure 3

The red-white heat map illustrates adjusted p-values for stage-wise differences within two cohorts. The y-axis lists the 41 taxa flagged by all four methods (LMM, ALDEx2, ANCOM-BC2, MaAsLin2; 0-4) as significant (BH-FDR < 0.05) and direction-concordant with LMM, and the x-axis lists cohorts (full, n = 117,671; IBD-only, n = 4,171) within three stage-pairwise-contrast panels (Stage 1 vs. Stage 2, Stage 1 vs. Stage 3, Stage 2 vs. Stage 3). Each cell represents the strength of the differential abundance result for that taxon, with stars (*, **, ***) overlaying cells at BH-FDR < 0.05, 0.001, and 10^{-4}. The blue- green heatmap depicts the mean relative abundance (log10) of each taxon across the two cohorts, each stratified by stage (1, 2, 3) on the x-axis.

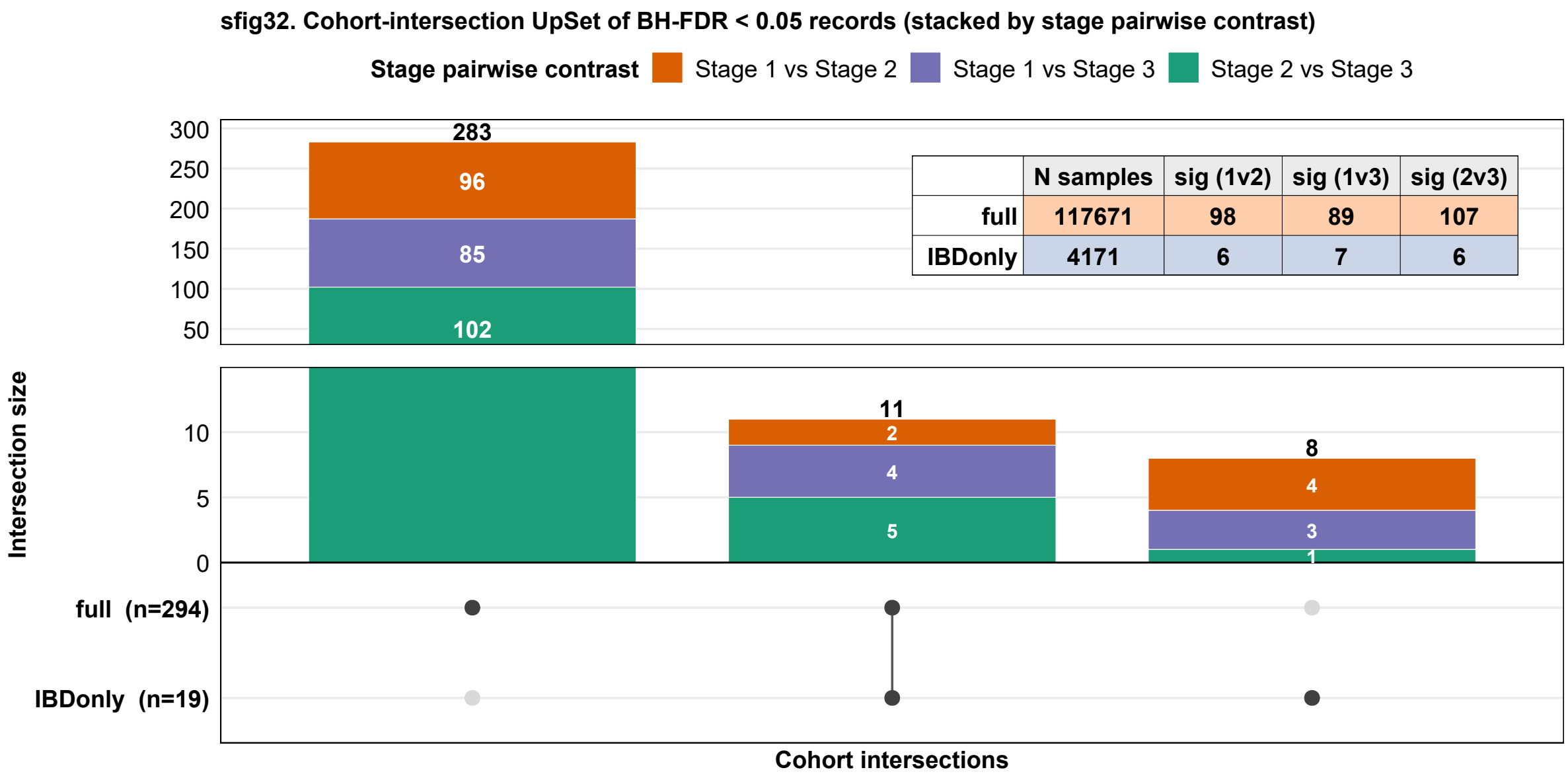

Figure S32. Cross-cohort consistency of BH-FDR- significant taxa between the full cohort and the IBD-only subcohort across stage pairwise contrasts. Related to Figure 3

The UpSet plot summarises the 302 unique taxon × stage-pair records reaching BH-FDR < 0.05 in at least one cohort, distributed across three stage pairwise contrasts (Stage 1 vs. Stage 2, Stage 1 vs. Stage 3, Stage 2 vs. Stage 3), based on the per-cohort LMM. Records show substantial cross-cohort consistency, with 58% (11/19) of IBD-only records also detected in the full cohort and 100% (11/11) of these doubly-significant records being direction-concordant between cohorts.

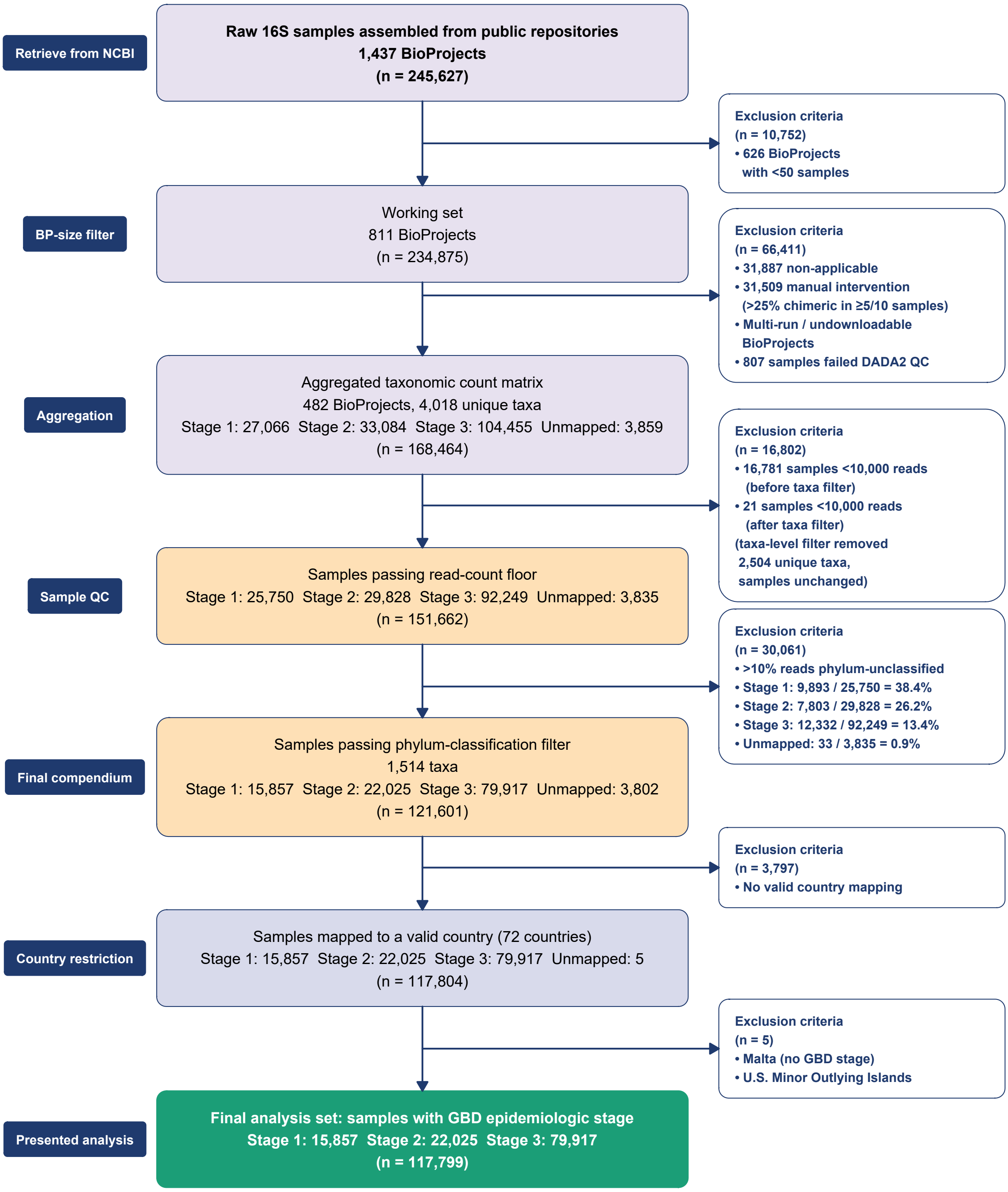

Figure S33. Overview of the sample-retention flow for the 16S compendium. Related to Figure 1

A list of the general steps in the data pipeline and how many samples completed each step. See Methods for more details about each process. From the aggregation step onwards, the number of samples in each GBD epidemiologic stage (Stage 1, Stage 2, Stage 3, and Unmapped) is shown within each step.

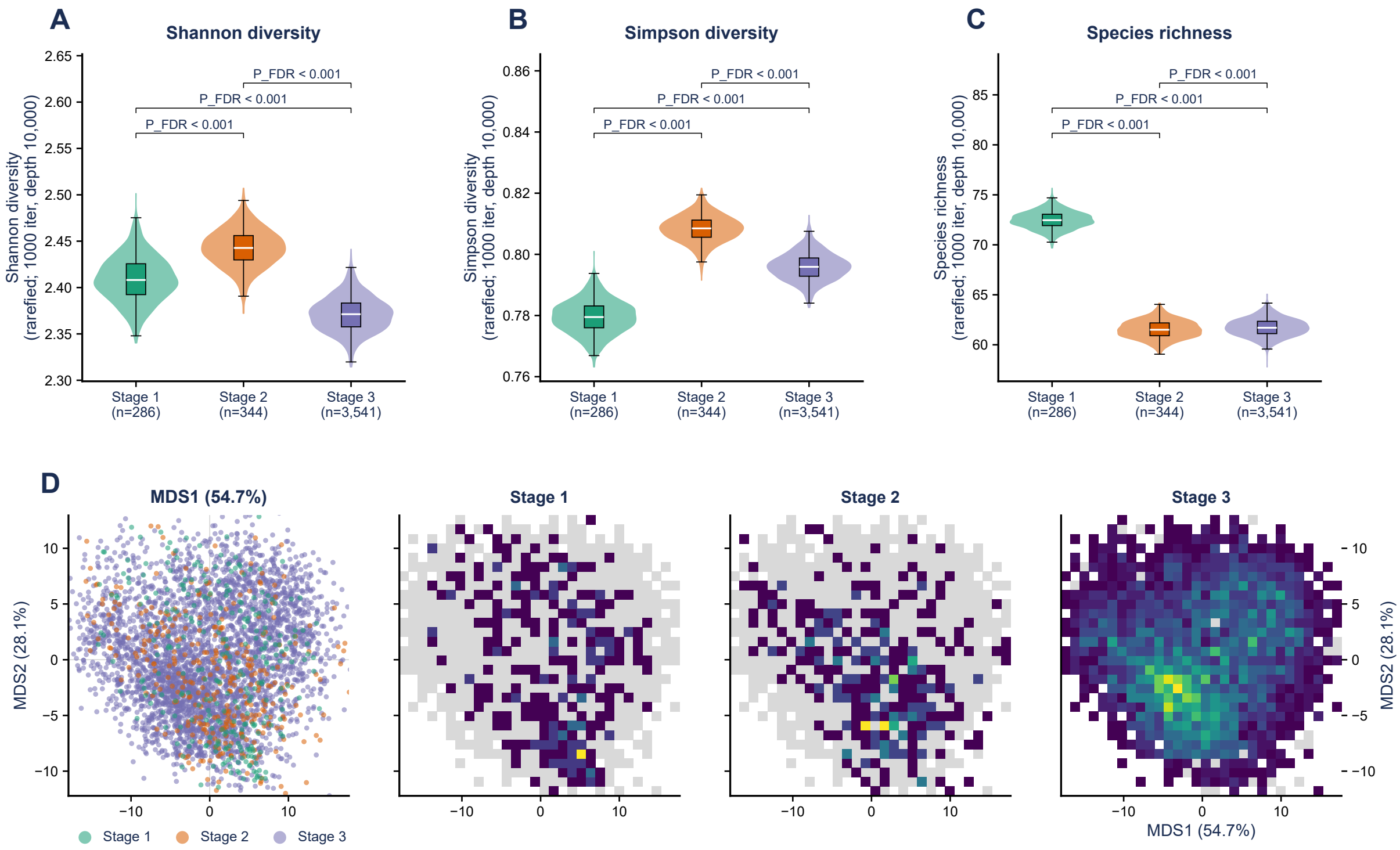

Figure S34. Alpha-diversity and PCoA analyses on an IBD-only subset. Related to Figure 1

(A–C) Violin plots showing the distributions of rarefied Shannon index, Simpson index, and species richness values for samples from the IBD-only subset assigned to the three IBD stages. (D) A series of plots illustrating the results of a PCoA of samples from all IBD stages. In the leftmost scatter plot, each point represents a single sample; the x-axis corresponds to the first PCoA axis, which explains the greatest proportion of variation in the dataset, and the y-axis corresponds to the second PCoA axis, which explains the second-greatest proportion of variation. The other three plots use the same axes but include only samples from a single IBD stage. These plots employ a heatmap design rather than a scatter plot to better visualize areas with high point overlap— yellow areas indicate regions of the ordination space with a higher density of samples, while dark blue areas represent regions with few (but not zero) samples. The gray shading indicates the area occupied by all samples across all stages.

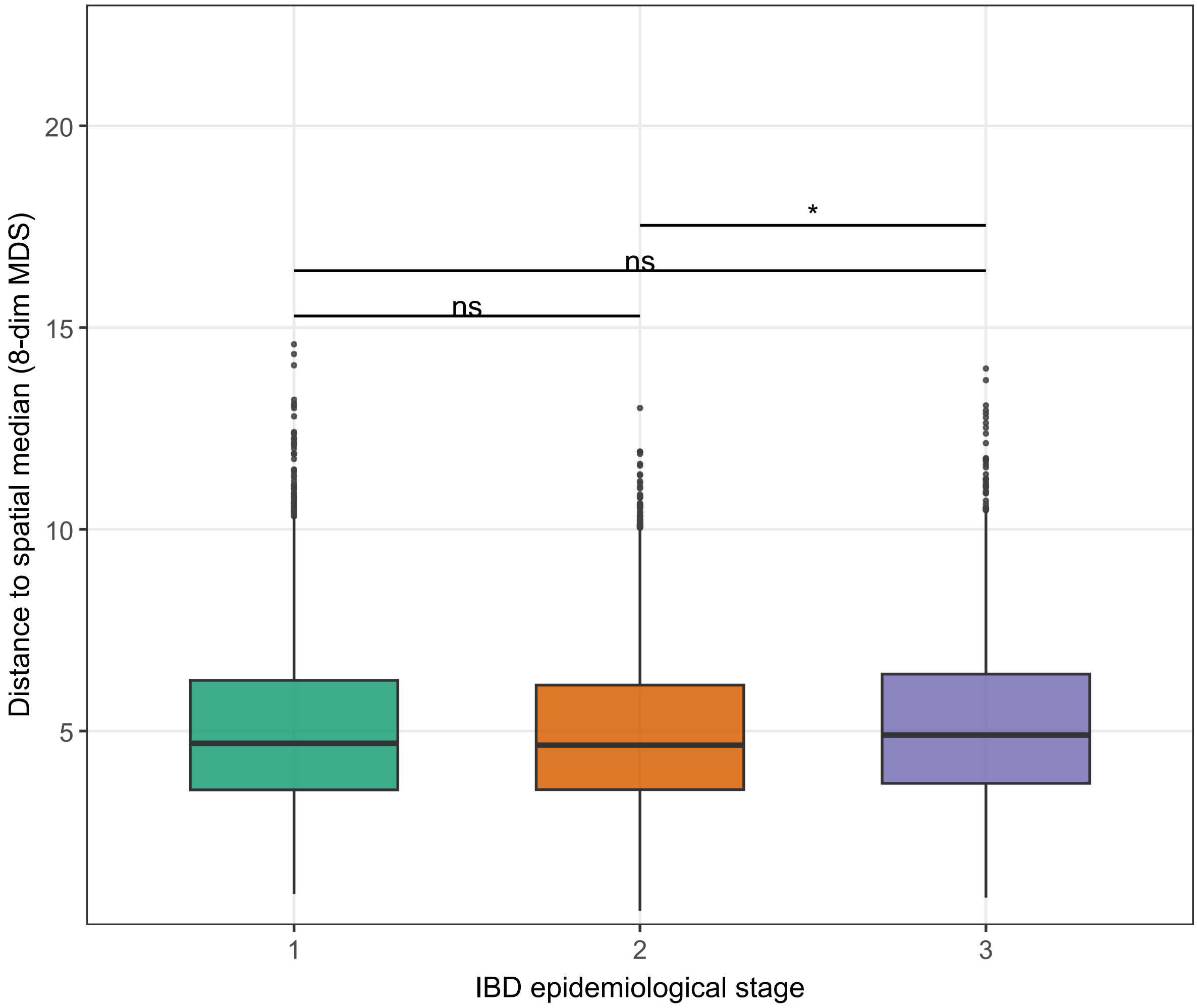

Figure S35. Multivariate dispersion across the three IBD epidemiological stages. Related to Figure 1

Boxplots showing the per-sample distance to the spatial median in the eight-dimensional MDS embedding for samples from countries assigned to the three IBD stages. The colors used here match those shown in Figure 1H. Statistical comparisons were performed using PERMDISP2.

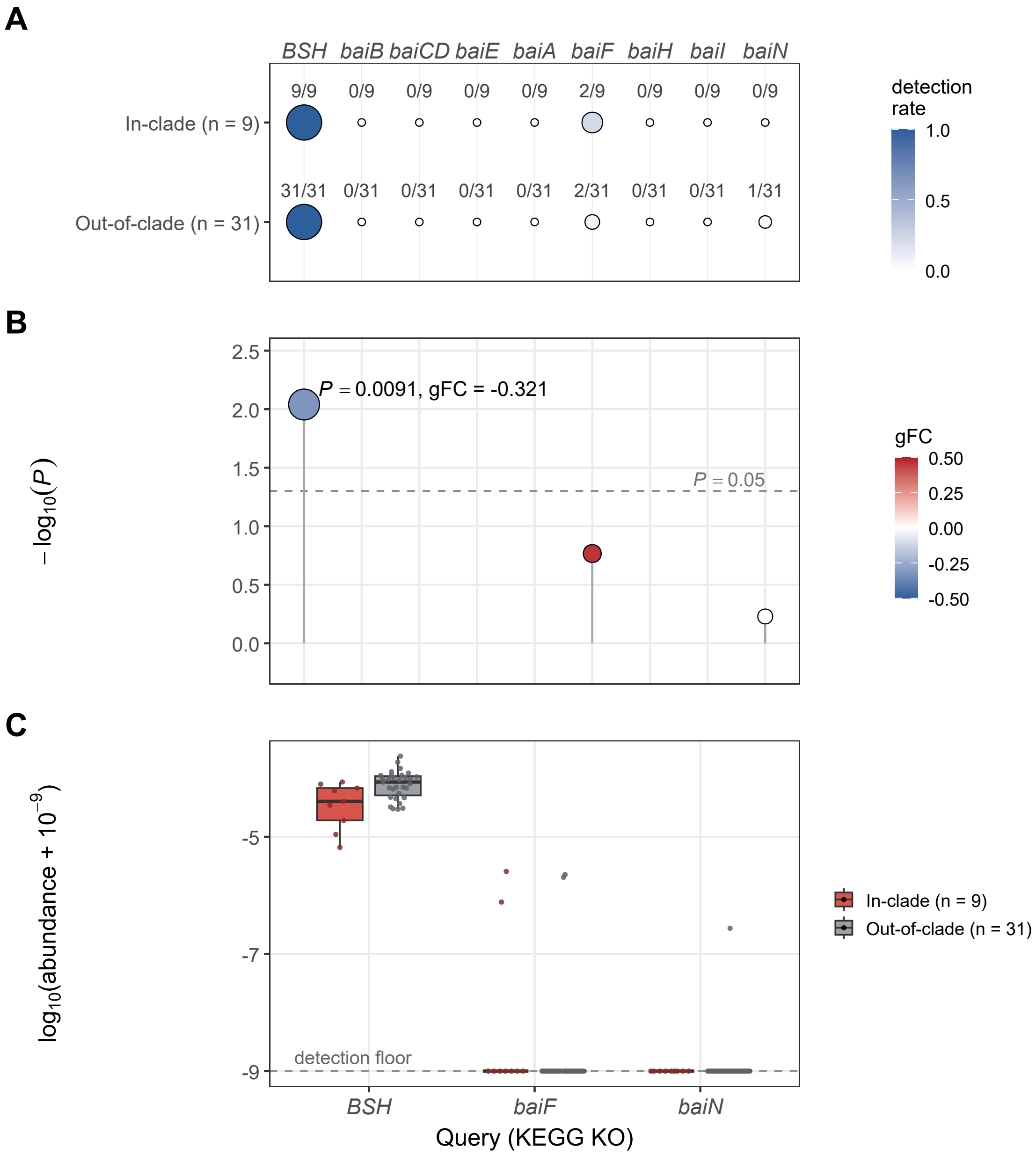

Figure S36. Subclade-level functional validation of bile acid metabolism genes in the IBD-enriched SGB4993 clade. Related to Figure 7

(A) Dot plot showing per-group detection rate of each query. (B) Lollipop plot showing $-\log_{10}$(blocked Wilcoxon *P*) per query; point fill encodes the generalized fold change (gFC), and the dashed line marks $P = 0.05$. (C) Boxplot showing per-sample $\log_{10}$ UniRef90 abundance for the three testable queries (BSH, baiF, baiN); the dashed line marks the detection floor.

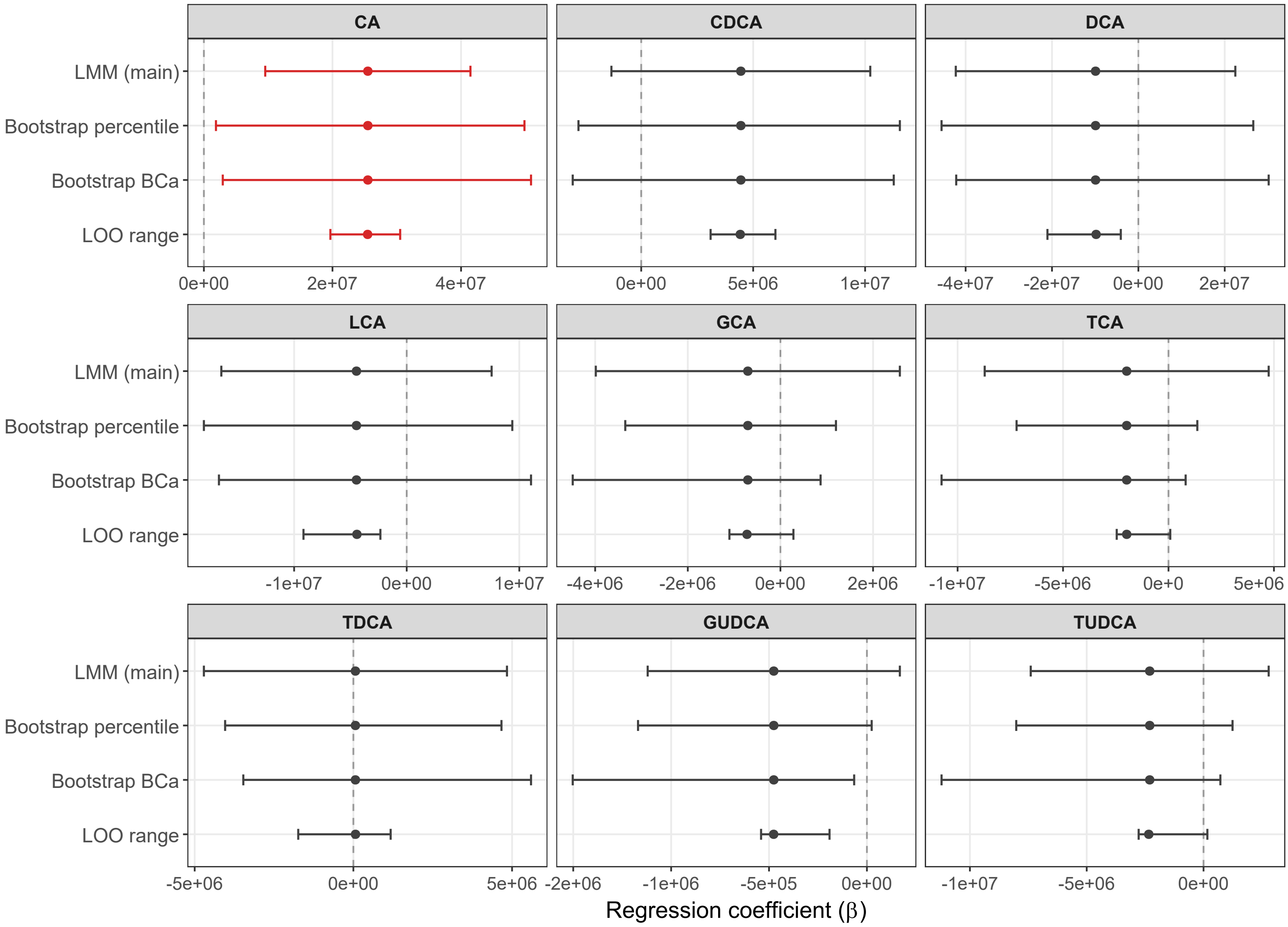

Figure S37. Robustness of the SGB4993 × bile-acid association across four estimate views per bile acid. Related to Figure 7

Forest plot showing the regression coefficient (β) (in-clade vs. out-of-clade) for each of the nine measured bile acids on the HMP2 IBDMDB n = 40 cohort (9 in-clade vs. 31 out-of-clade). The x-axis denotes the regression coefficient (β) for the in-clade-vs.-out-of-clade contrast, and the y-axis lists the four estimate views (LMM main, bootstrap percentile, bootstrap BCa, leave-one-out range). The dashed vertical line marks $\beta = 0$. All four estimate views for CA are shown in red as the only bile acid reaching BH-FDR < 0.05 in the LMM main analysis.
