# Supplementary material for: Global gut microbiome atlas identifies epidemiologic-stage-specific signatures in inflammatory bowel disease
Source: Cell Rep Med. 2026 Aug 10;7(9):102974. doi: 10.1016/j.xcrm.2026.102974 (PMC13589510; doi:10.1016/j.xcrm.2026.102974)
Supplement: Supplementary file 2 [file mmc10.pdf]

Cell Reports
Medicine

Article

Global gut microbiome atlas identifies epidemiologic-stage-specific signatures in inflammatory bowel disease

Graphical abstract

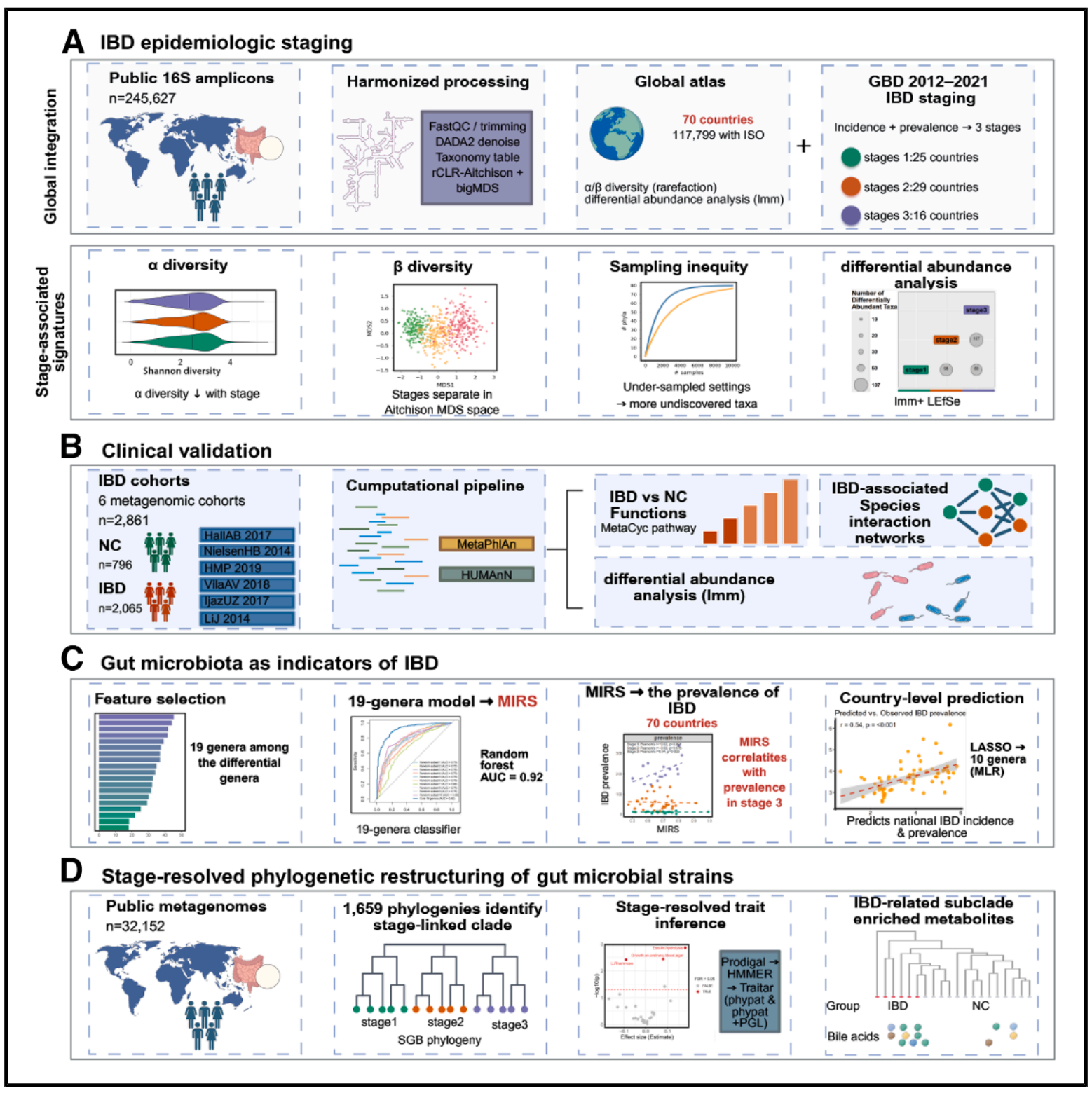

Highlights

- A 16S atlas of 117,799 samples across 70 countries spans three IBD stages
- *Blautia* rises with IBD across scales, while *Escherichia/ Shigella* reverses
- An IBD-enriched *Eisenbergiella* subclade correlates with fecal cholic acid
- A 19-genus MIRS reaches AUC 0.92 and tracks prevalence in stage 3 countries

Authors

Jinxia Zhai (翟锦霞), Yingjie Li (李迎杰), Jiameng Liu (刘佳梦), ..., Yuhan Sun (孙羽晗), Jingsheng Yu (于景盛), Cong Dai (戴聪)

Correspondence

cdai@cmu.edu.cn

In brief

By integrating 117,799 gut 16S profiles from 70 countries alongside shotgun and strain-level metagenomes, Zhai et al. build a global atlas linking IBD epidemiologic stages to gut microbial ecology. They define stage-resolved microbial signatures and a 19-genus risk score (MIRS), which tracks national IBD burden in high-prevalence regions.

Check for updates

Zhai et al., 2026, Cell Reports Medicine 7, 102974
September 15, 2026 © 2026 The Authors. Published by Elsevier Inc.
https://doi.org/10.1016/j.xcrm.2026.102974

CellPress

Cell Reports Medicine

CellPress
OPEN ACCESS

Article

Global gut microbiome atlas identifies epidemiologic-stage-specific signatures in inflammatory bowel disease

Jinxia Zhai (翟锦霞),[1] Yingjie Li (李迎杰),[2] Jiameng Liu (刘佳梦),[1] Xinyi Su (苏心怡),[1] Runze Cui (崔润泽),[1] Dianyu Zheng (郑殿宇),[1] Yuhan Sun (孙羽晗),[1] Jingsheng Yu (于景盛),[3] and Cong Dai (戴聪)[1,4,*]

[1]Department of Gastroenterology, First Hospital of China Medical University, Shenyang 110001, Liaoning, China

[2]Department of Gastroenterology, First Affiliated Hospital, Jinzhou Medical University, Jinzhou 121001, Liaoning, China

[3]Department of Rare Diseases, Peking Union Medical College Hospital, Peking Union Medical College and Chinese Academy of Medical Sciences, Beijing 100730, China

[4]Lead contact

*Correspondence: cdai@cmu.edu.cn

https://doi.org/10.1016/j.xcrm.2026.102974

SUMMARY

The global rise of inflammatory bowel disease (IBD) reflects environmental shifts, yet how these changes are embedded in the gut microbial ecology remains unclear. We construct a microbiome atlas comprising 245,627 profiles. By classifying countries into three epidemiologic stages, we establish a framework. As the IBD burden increases, the gut microbial alpha diversity declines, and community structures form distinct clusters. This transition is characterized by a gradient of core genera. Integrating six shotgun metagenomic cohorts, we identify the depletion of anabolic pathways in IBD patients. Strain-level analysis reveals that epidemiologic staging shapes genetic architecture within species, identifying an IBD-enriched subclade of *Eisenbergiella* associated with elevated fecal cholic acid. We develop a microbial inflammatory risk score (MIRS), based on 19 genera, that discriminates IBD from controls (area under the curve [AUC] = 0.92). MIRS correlates with IBD prevalence. Our study provides an atlas linking epidemiology to microbiome ecology and strain evolution, offering a foundation for population-level surveillance and interventions in IBD.

INTRODUCTION

The microbiome in inflammatory bowel disease (IBD) has been extensively investigated.[1–7] A hallmark of IBD-associated dysbiosis is a pronounced reduction in species diversity,[5] driven by a depletion of obligate anaerobes, including the Clostridia, accompanied by an expansion of facultative aerobes such as the Enterobacteriaceae.[8] A central challenge in microbiome research is to move beyond these associative observations toward elucidating the mechanisms that underlie disease initiation and progression.[9]

Accumulating evidence indicates that microbiome variation in individuals with IBD is shaped by multiple factors, including host genetics[10] and ethnic background,[11–13] many of which are closely linked to the geographical region.[14–18] Recently, an epidemiologic staging framework has been proposed that classifies countries into early-, intermediate-, and late-stage IBD regions based on national-level incidence and prevalence thresholds.[19] Across regions at different stages of IBD epidemiology, microbiome composition may establish distinct relationships with human health, highlighting the need for research strategies that integrate population-scale epidemiologic patterns with microbial ecological features.

However, advancing from correlation to mechanistic insight requires resolving heterogeneity within microbial species. Investigating intraspecies metagenomic variation[20] can, therefore, provide a deeper understanding of host-microbe interactions. Previous studies have revealed pronounced biogeographic variability[21–23] of the gut microbiota and have demonstrated that strain variation is associated with different life stages,[24] human lifestyle,[25] and phenotypic variability.[26] In recent years, the growing availability of thousands of metagenomes from healthy individuals and those with IBD[2–7,27–30] has enabled large-scale evolutionary analyses of microbial communities across regions at different stages of IBD epidemiology, creating opportunities to identify microbiome-based biomarkers associated with IBD.

Based on this framework, we integrated and constructed a dataset comprising 117,799 samples from 70 countries, in which all samples were assigned to distinct IBD stages. We demonstrate the value of this resource for evaluating microbiome compositional patterns across the three global IBD stages and across 70 countries, as well as for quantitatively assessing current gaps in knowledge. In parallel, we assembled an expanded metagenomic dataset encompassing six IBD cohorts, including 1,446 IBD samples and 558 healthy controls. This dataset allowed us to systematically investigate the differences

© 2026 The Authors. Published by Elsevier Inc.
This is an open access article under the CC BY license (http://creativecommons.org/licenses/by/4.0/).

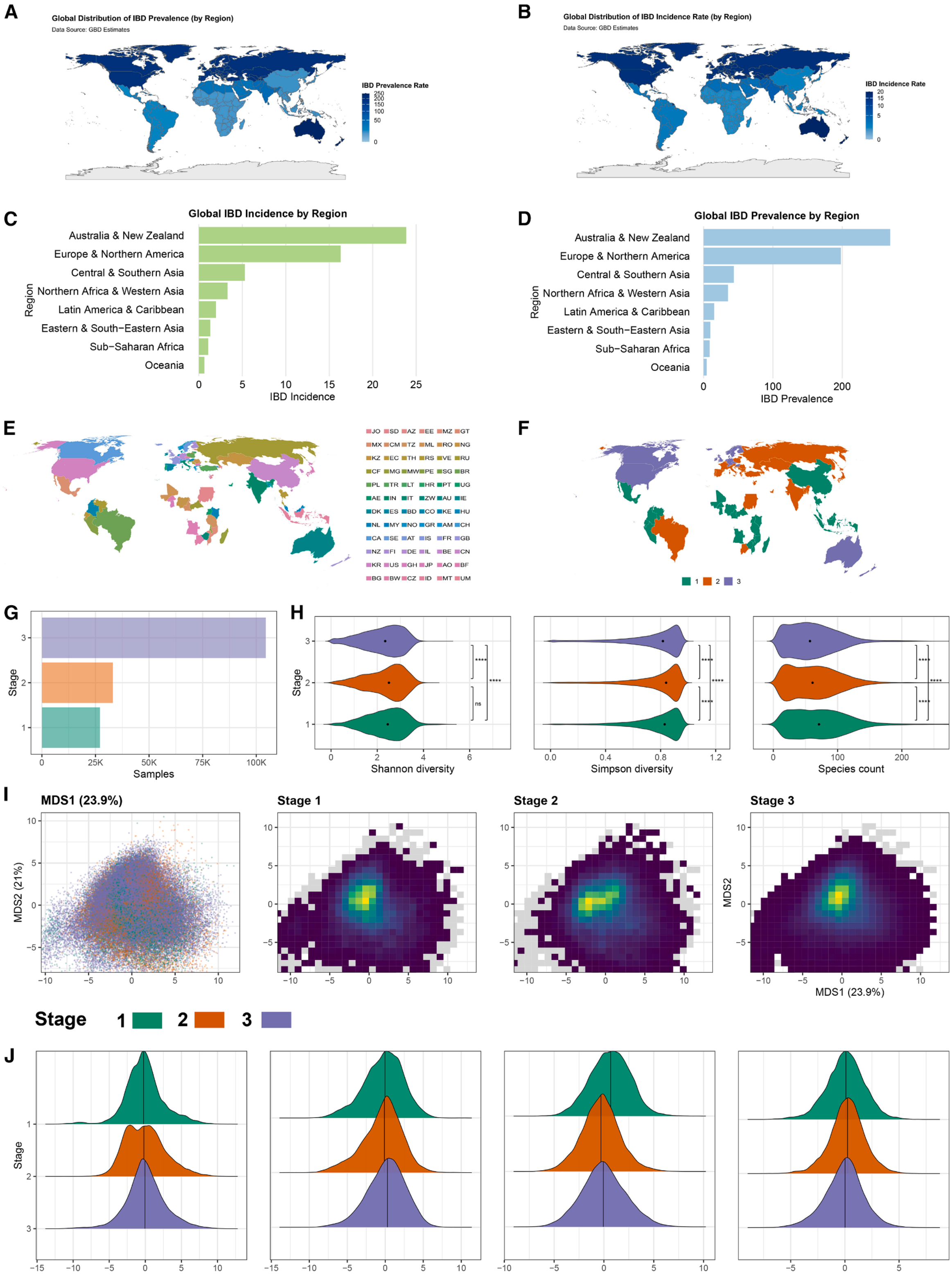

Figure 1. Regional structure

(A–D) (A and B) Global distribution of IBD prevalence and incidence rates. (C and D) Region-specific prevalence (D) and incidence (C); regions on the *y* axis and burden on the *x* axis.

(legend continued on next page)

in microbial composition and metabolic function between patients with IBD and healthy individuals, interaction networks of IBD-associated microbes in healthy populations, cross-cohort-consistent core microbial signatures of IBD, and the use of these core signatures in machine-learning models to predict IBD incidence and prevalence across 70 countries worldwide. Furthermore, leveraging an additional resource of 32,152 metagenomes with phylogenetic trees spanning 1,659 microbial species, we identified specific species clades enriched in IBD as well as strains exhibiting stage-specific associations with IBD epidemiology. Overall, by integrating global, multi-scale microbiome and epidemiologic data, we systematically characterized, at a global scale, the microbial community structures, core biomarkers, and evolutionary differentiation features corresponding to distinct IBD epidemiological stages, thereby providing a unified framework for understanding microbiome ecological shifts and their potential mechanisms during the global progression of IBD.

RESULTS

Regions representing three epidemiological stages of IBD and 70 countries exhibit distinct gut microbiome profiles

The IBD incidence and prevalence vary substantially across regions, with the highest in Australia and New Zealand (Figures 1A–1D). After excluding samples with unknown ISO codes, 117,804 samples remained, representing 72 countries, of which 70 were assigned to an IBD epidemiological stage (Figures S1 and S33). The United Arab Emirates exhibited the highest alpha diversity ($n = 1$; Shannon mean = 3.48, Simpson mean = 0.95, richness mean = 112, based on a single sample; Figures S2–S4; Table S1). At the epidemiological level, stage 1 had the highest average diversity ($n = 15,857$; Shannon mean = 2.36, Simpson mean = 0.77, richness mean = 82), followed by stage 2 ($n = 22,025$; Shannon mean = 2.34, Simpson mean = 0.77, richness mean = 64) and stage 3 ($n = 79,917$; Shannon mean = 2.22, Simpson mean = 0.75, richness mean = 62) (Figures 1E–1H; Table S2). The alpha diversity differed significantly across the 72 countries (Wilcoxon rank-sum test, false discovery rate [FDR] <0.05; Table S3). Similarly, the Shannon diversity differed significantly among the three stages (FDR <0.05): stage 1 vs. stage 3 and stage 2 vs. stage 3 showed highly significant differences (Wilcoxon rank-sum test, FDR $<2.2 \times 10^{-16}$; Table S4).

Given that the read depth varied substantially across countries, we re-evaluated this analysis. The lowest median read depth was observed in Botswana (11,767 reads per sample; Table S5). Median read depths were 43,925, 46,190, and 41,672 reads per sample for stages 1, 2, and 3, respectively (Table S6). To control for these differences, we conducted a rarefaction analysis (see STAR Methods, Figures S5 and S6). The mean Shannon diversity values remained essentially unchanged or slightly decreased, but the overall trends remained consistent (Tables S7 and S8). Collectively, our global analysis reveals pronounced cross-country and cross-stage differences in gut microbial diversity. Importantly, as the global IBD burden increased, gut microbiome diversity systematically declined, suggesting that microbial dysbiosis may parallel the global escalation of IBD prevalence. This decline in diversity is also in line with previous studies reporting that patients with IBD generally display reduced Shannon and Simpson indices compared with healthy individuals.[31] To verify that this stage-wise decline was not driven by participants with other diseases or unspecified health status, we re-evaluated this analysis on an IBD-only subset ($n = 4,171$; stage 1 = 286, stage 2 = 344, stage 3 = 3,541) using the same rarefaction procedure (see STAR Methods). The overall stage-wise trends remained consistent (Wilcoxon FDR $<5 \times 10^{-6}$ for every stage pair; Figures S34A–S3C; Tables S55 and S56). In this subset, stage 1 was represented by samples from a single country (China) and stage 2 by samples from seven countries, so the stage 1 vs. stage 2 ordering for Shannon (2.41 vs. 2.44) reflects the country composition of each stage rather than a biological difference; stage 3, nevertheless, retained the lowest Shannon (2.37), with richness in stages 2 and 3 (61.5 and 61.7) below that in stage 1 (72.5).

To explore the compositional differences among regions at different IBD stages, we performed principal coordinate analysis (PCoA) (Figure 1I; see STAR Methods). Even in two dimensions, systematic differences among the three stages were evident. This persisted beyond the first two axes: distinct separations among the three stages (Figure 1J) and 70 countries (Figure S7) appeared in the first four axes, and across all eight axes (Figures S8–S14), clusters were far more compact and distinct than expected by chance (Davies-Bouldin index = 13.26; $p < 4 \times 10^{-6}$; Figure S15). The same test on the IBD-only subset gave a comparable result (Davies-Bouldin index = 14.09; $p < 2 \times 10^{-5}$; Figure S34D); PERMANOVA on the robust Aitchison distance further showed that stage accounted for 0.51% of the total variation ($F = 10.94$, $p = 0.005$), sex 0.42%, and age group 1.85% (Table S57). To quantify stage-specific dispersion, we performed PERMDISP2 on the same eight-dimensional MDS embedding. The mean distance to the spatial median was the largest for stage 3 (5.19), intermediate for stage 1 (5.11), and the smallest for stage 2 (5.03), with $F(2, 5,997) = 3.17$ (parametric $p = 0.042$; permutation $p = 0.037$); Tukey's honestly significant difference (HSD) test identified stage 3 vs. stage 2 as the only significant pairwise contrast ($p = 0.032$; stage 1 vs. stage 2, $p = 0.452$; stage 1 vs. stage 3, $p = 0.387$; Table S52; Figure S35). This reflects a pronounced disruption of the gut

(E) Map of the 72 countries classified into world regions.
(F) Map of countries across the three stages categorized into world regions; colors match (H).
(G) Bar plot of the number of samples per stage (*x* axis, sample count; *y* axis, stage).
(H) Violin plots of observed Shannon index, Simpson index, and species count for samples from countries in the three stages.
(I) PCoA of samples from all stages. In the top-left scatterplot, each point is one sample; axes are the first two PCoA axes. The other three plots share these axes but show one stage each. Yellow, higher sample density; dark blue, few (but not zero) samples; gray shading, area occupied by all samples across all stages.
(J) Density plots of the first four axes of variation from the ordination in (I). Each panel is one factor (*x* axis, factor value; *y* axis, relative frequency of that value within a given IBD stage). See also Data S1.

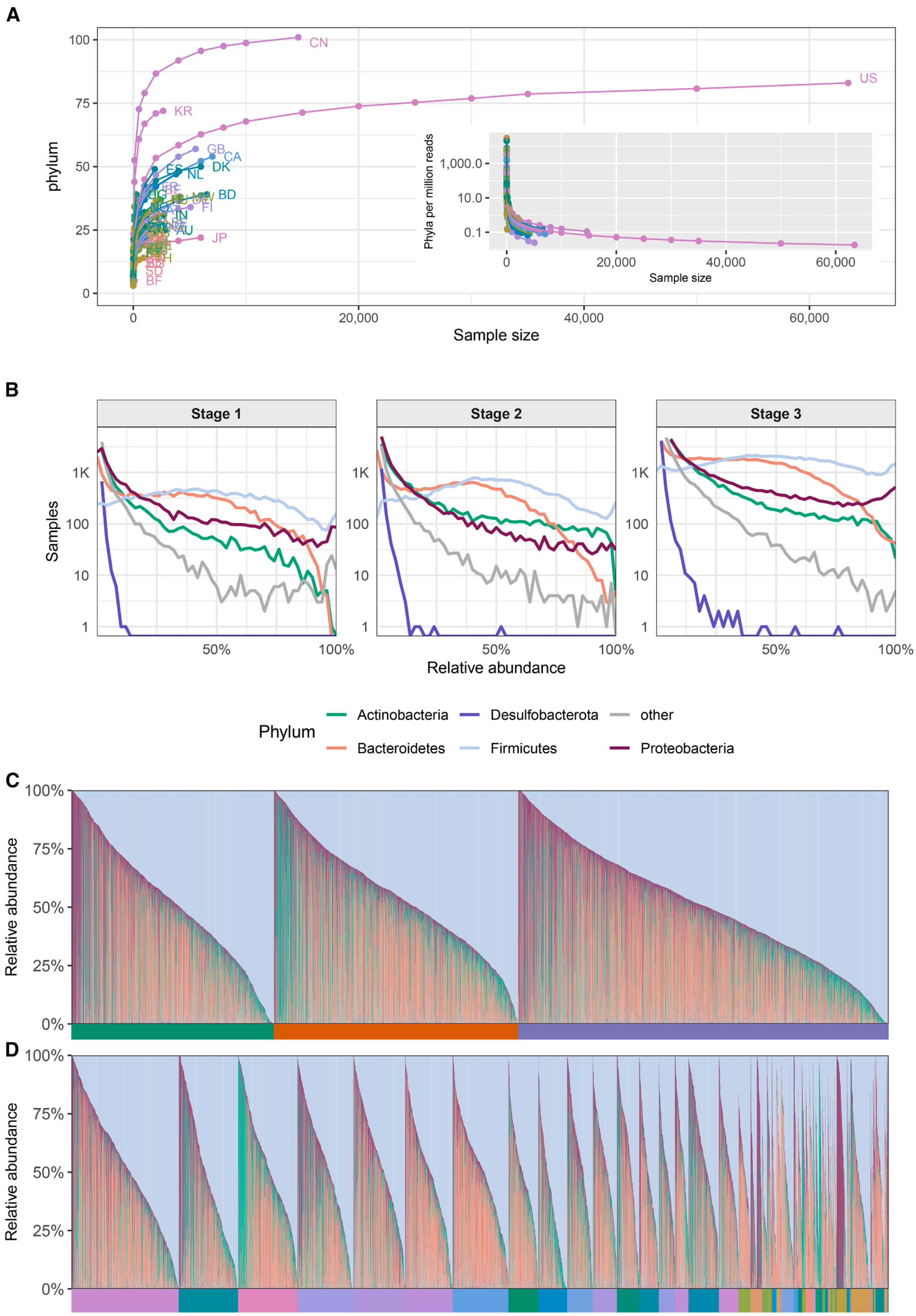

(legend on next page)

microbial homeostasis during disease progression, with greater heterogeneity and signatures of ecological drift.

Uneven microbiome sampling leaves taxa undiscovered

Previous studies have characterized the microbiome diversity across the eight major world regions (Sub-Saharan Africa, Northern Africa and Western Asia, Central and Southern Asia, Eastern and South Eastern Asia, Latin America and the Caribbean, Australia and New Zealand, Oceania, and Europe and Northern America). Here, to investigate the microbiome diversity at the level of individual countries, we repeatedly subsampled each country and quantified the number of unique microbial phyla present in the selected microbiome samples (Figure 2A; Figure S16). We included all 102 phyla identified in the initial DADA2 classification and all samples with a defined country assignment. Although China had approximately 49,000 fewer samples than the country with the largest dataset (the United States), the number of phyla discovered in samples from China far exceeded that of other countries. Among the 102 phyla detected, 101 were present in Chinese samples, compared with 83 in samples from the United States.

The rate of new phylum discovery declined after the first few thousand samples in every country. On average, eight more phyla were identified in 2,000 Chinese samples than in 1,000 samples, yet no additional phyla were found when increasing the sample size from 8,000 to 10,000, confirming the decline in discovery rate. Among the first 10,000 samples from the United States, 67 phyla were identified, whereas increasing the sample size to 50,000 yielded only 13 additional phyla, bringing the total to 80. When 10,000 samples were analyzed, the United States exhibited a discovery rate of 0.094 new phyla per million reads, which decreased to only 0.022 taxa per million reads when 50,000 samples were included, indicating an extremely low yield of new taxa despite increased sequencing effort. Most microbial phyla in the United States and China have already been comprehensively captured, and further sequencing contributes little to novel phylum discovery. In contrast, when all available samples from Australia were analyzed, the discovery rate remained at an average of 0.30 new phyla per million reads (Figure 2A, inset). The relatively high discovery rates outside China and the United States suggest that additional sampling in under-represented countries could uncover numerous unrecorded microbial phyla (Table S9).

We further analyzed the dominant phyla across 70 countries and the three stages of IBD (Figure 2B; Figure S17). At the stage level, the relative abundance of Firmicutes in stage 3 samples was approximately uniformly distributed, whereas in stage 2, the distribution of Firmicutes peaked around 50%, with higher abundances becoming progressively less common. The abundance of Desulfobacterota increased gradually from stage 1 to stage 3. Compared with stages 1 and 2, Proteobacteria became the most dominant phylum in stage 3, indicating a marked expansion of Proteobacteria in most patient samples (Table S10). Consistent with this shift, Actinobacteria abundance showed a pronounced reduction between stages 2 and 3 (Wilcoxon test, FDR $<2.2 \times 10^{-16}$; Figure 2C; Table S12). We also observed broad patterns of diversity within countries (Figure 2D). Although Firmicutes dominated across nearly all countries and IBD stages, samples from India, the Netherlands, and Sweden exhibited significantly higher relative abundances of Actinobacteria (Wilcoxon test, FDR <0.05) and lower abundances of Bacteroidetes (Wilcoxon test, FDR <0.05; Figure 2D; Table S11).

Systematic differences in microbiome composition across 70 countries and the three IBD-associated global stages

To quantify the differences in specific microbial taxa across 70 countries and among the three IBD-associated global stages, we performed differential abundance analysis under a five-method framework (see STAR Methods; Table 43; Figure S26). Pairwise comparisons between countries and between IBD stages revealed pronounced differences among these taxa (Table S13). When comparing the three IBD stages, we identified 98 differentially abundant taxa between stage 1 and stage 2, 89 between stage 1 and stage 3, and 107 between stage 2 and stage 3 (Figure 3A). At the country level, the largest number of differentially abundant taxa was observed between Romania and the United States (121 taxa) (Figure S18).

We next examined taxa differing between stage 3 and the other stages. Among these differential taxa, *Bifidobacterium* displayed consistently low adjusted p values in comparisons between stage 3 and stages 1 or 2, indicating significant inter-stage differences (Figure 3B). Across the three stages, *Blautia* exhibited a progressive increase in relative abundance (linear mixed-effects model [LMM] FDR $<2.2 \times 10^{-16}$). This pattern suggests that *Blautia* may follow a trajectory of gradual enrichment along disease progression or developmental transition, increasing from early to late stages. *Succinivibrio*, by contrast, displayed a markedly lower relative abundance in stage 3 than in stage 1 (LMM FDR $<2.2 \times 10^{-16}$) and stage 2 (LMM FDR = 5.4×10^{-8}), with stage 2 also significantly lower than stage 1 (LMM FDR $<2.2 \times 10^{-16}$). This decrease across stages implies a potential association between depleted *Succinivibrio*

Figure 2. Countries vary in microbiome composition

(A) Number of unique phyla detected in subsamples of varying size per country. Each point is the mean across 1,000 subsampling iterations (*x* axis, number of samples selected; *y* axis, unique phyla; color, country). The inset uses the same *x* axis and color but plots the average number of newly discovered phyla per million sequencing reads.

(B) Histograms of the relative abundance distribution of the most prevalent phyla. Each panel visualizes all samples from one of the three IBD stage regions (*x* axis, relative abundance; *y* axis, number of samples). Each line is one phylum by color.

(C) Stacked bar chart of microbial phylum relative abundance in 10% of randomly selected samples. Each bar is one sample; colored segments give the per-phylum relative abundance. Samples are ordered by IBD stage.

(D) Stacked bar chart of the five most prevalent phyla. Each column is one sample; colored segments give the per-phylum relative abundance. Samples are ordered by country (colors as in Figure 2A). See also Data S2.

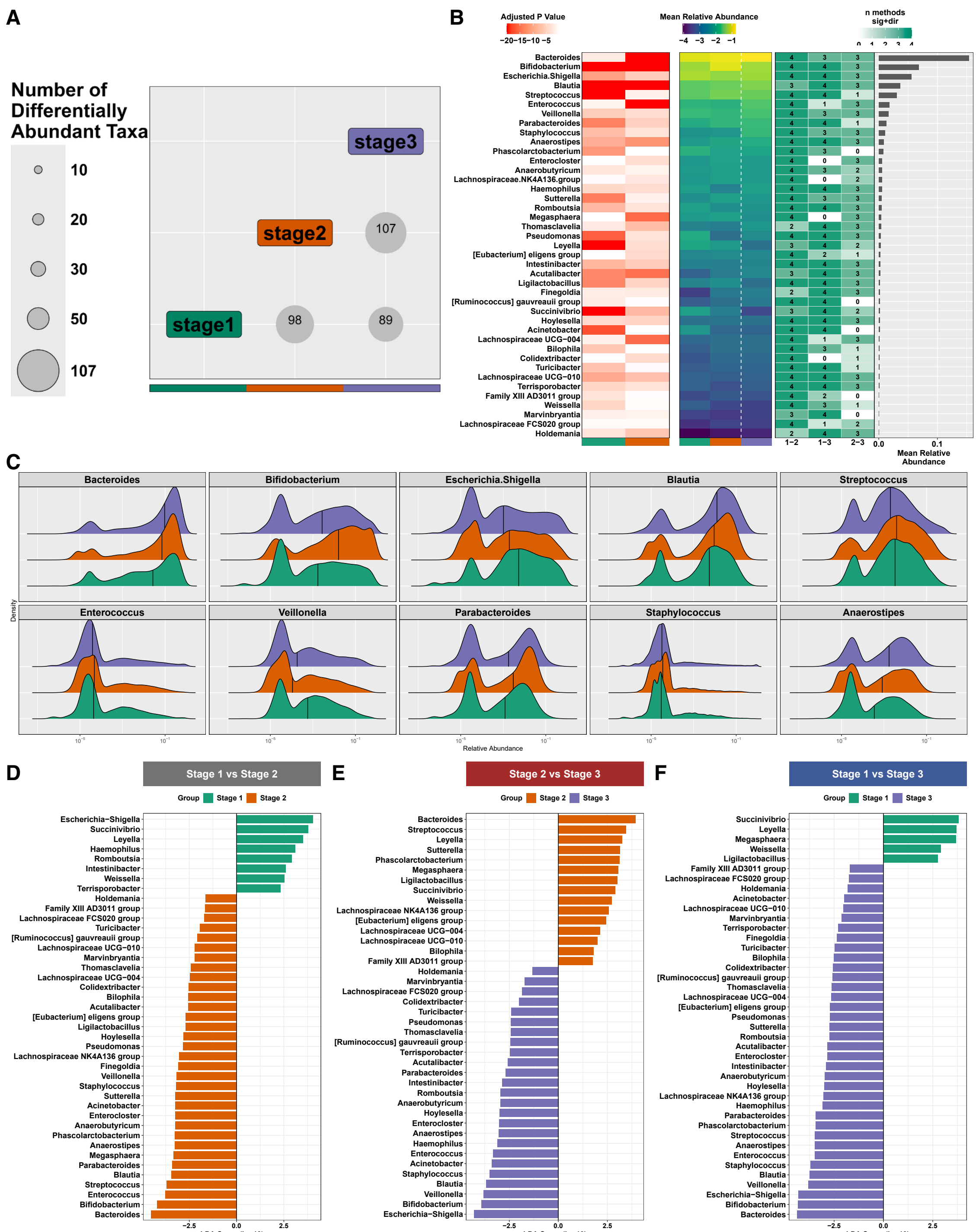

Figure 3. Taxa are differentially abundant between IBD stage-associated regions

(A) The *x* and *y* axes are colored by stage; at each intersection, circle size and the inside number give the number of taxa significantly different between the two stages.

(B) Red-white heatmap, adjusted *p* values for stage-wise differences when stages 1 and 2 are each compared with stage 3 (*y* axis, all evaluated taxa; *x* axis, stages 1 and 2); each cell is the strength of the differential abundance result. Blue-green heatmap, mean relative abundance of each taxon across the three stages (*x* axis). Green-white heatmap, number of methods flagging each taxon as significant. Bar chart, mean relative abundance averaged across all stages.

(legend continued on next page)

abundance and disease progression or host physiological shifts. At the country level, we found that *Succinivibrio* showed the lowest adjusted *p* values across the US-country comparisons, indicating strong cross-country differences. Specifically, the relative abundance of *Succinivibrio* was significantly higher in Malawi (LMM FDR< 2.2×10^{-16}), Cameroon (LMM FDR< 2.2×10^{-16}), and India (LMM FDR< 2.2×10^{-16}) than in the United States (Table S14; Figure S19).

A closer examination of the relative abundance distributions across the three IBD stages revealed more specific patterns. Nearly all stage regions contained numerous samples with high *Bacteroides* abundance, as evidenced by strong peaks near 1 (Figures 3C; Figure S20). Notably, stage 1 regions displayed fewer samples with such extreme *Bacteroides* enrichment, showing a flatter peak near 1 compared with stages 2 and 3. We further evaluated inter-country variation and observed marked heterogeneity in genus-level patterns. For example, in Sudan, Thailand, and Portugal, the peak of *Bacteroides'* relative abundance was near 0, while in most other countries, it approached 1 (Figure S21).

To evaluate the robustness of the stage-pairwise differences described above, we assessed the cross-method consensus across four sample-level differential abundance (DA) methods under both socio-demographic index (SDI)-naive and SDI-adjusted scenarios. For SDI-naive baseline, all three contrasts retained substantial four-method consensus (stage 1 vs. 2: 24.6%; stage 1 vs. 3: 27.6%; stage 2 vs. 3: 18.2%). The stage 1 vs. stage 2 (20.2%) and stage 1 vs. stage 3 (13.8%) contrasts retained substantial four-method consensus under SDI adjustment, indicating that these stage-pairwise taxonomic differences are primarily driven by stage assignment rather than by country-level industrialization heterogeneity (Figure S26).

When comparing the three IBD stages using a five-method consensus differential abundance framework (sig+dir at Benjamini-Hochberg-adjusted false discovery rate [BH-FDR] <0.05), 7 genus-level biomarkers (including *Bifidobacterium*, *Succinivibrio*, and *Anaerostipes*) were identified, all in the stage 1 vs. stage 3 contrast (Figures 3D–3F; Table S43). *Anaerostipes* was consistently identified as a potential biomarker by all five differential abundance methods (stage 1 vs. stage 3: LMM FDR = 3.35×10^{-9}; ALDEx2 FDR = 3.94×10^{-6}; ANCOM-BC2 FDR = 2.41×10^{-8}; MaAsLin2 FDR = 6.42×10^{-6}; LEfSe FDR = 1.01×10^{-3}) and the machine-learning-based methods (see below).

To further assess robustness to V-region primer-specific amplification bias, we additionally repeated the LMM differential-abundance analysis within the three largest V-region subsets (v4, v3–v4, and v1–v2; see STAR Methods). Direction concordance between the v4 and v3–v4 subsets across the 203 taxa was 73.9% (150/203) for stage 1 vs. stage 2 and 78.3% (159/203) for stage 1 vs. stage 3, and five genera—*Segatella*, *Mitsuokella*, *Leyella*, *Succinivibrio* and *Muribaculaceae incertae sedis*—were sig+dir concordant (BH-FDR <0.05) across all three stage pairs, including *Succinivibrio* whose depletion in highly industrialized stage 3 regions is described above (Figures S29 and S30; Tables 53 and S54).

To evaluate whether the stage-associated microbial shifts observed in the full-cohort differential-abundance analysis are robust within IBD patients themselves, we restricted the analysis to an IBD single-disease subset. Among the 11 taxon-stage-pair comparisons significant at BH-FDR <0.05 in both the full-cohort and IBD-only analyses, all changed in the same direction; across all 203 taxa, the directional concordance rates for the three stage pairs were 44.3%, 50.7%, and 64.0%. Taken together, these results indicate that the stage-associated microbial signal observed in the full-cohort analysis is preserved within the IBD single-disease subset (Tables S45 and S46; Figures S31 and S32).

Systematic differences in microbiome composition between IBD and healthy individuals

To quantify disease-associated variation in gut microbial composition at both the genus and species levels, we applied an LMM that incorporated project and median read length[32] random effects to account for experimental batch effects and technical artifacts, while treating age and country as fixed covariates to control for potential demographic influences on microbial abundances (STAR Methods). Pairwise comparisons between disease states revealed substantial differences in microbial relative abundances across taxa. At the genus level, we identified 23 differentially abundant genera between IBD and controls (FDR <0.05) (Table S18), 21 between normal controls (NC) and ulcerative colitis (UC) (FDR <0.05), 25 between NC and Crohn's disease (CD) (FDR <0.05), and 19 between UC and CD (FDR <0.05) (Figure 4A; Table S19). At the species level, we observed 21 differentially abundant species between IBD and NC (FDR <0.05; Table S20), 22 between NC and UC (FDR <0.05), 26 between NC and CD (FDR <0.05), and 20 between UC and CD (FDR <0.05) (Figure 4B; Table 21). At the genus level, *Blautia* showed markedly higher relative abundance in IBD compared with controls (Figure 4H; FDR <2.2×10^{-16}). Consistently, *Blautia* abundance was significantly elevated in both CD and UC relative to NC (Figure 4G; NC vs. CD: FDR <2.2×10^{-16}; NC vs. UC: FDR = 4.66×10^{-9}). Moreover, *Blautia* abundance was significantly higher in CD than in UC (FDR = 3.84×10^{-5}). Notably, in our previous analyses of the three global IBD epidemiological stages, *Blautia* exhibited a monotonic enrichment pattern, increasing progressively from stage 1 to stage 3 (Figure 3B; all pairwise FDR <0.05), suggesting that *Blautia* represents a consistently enriched IBD-associated genus whose abundance escalates along the trajectory of disease progression. *Anaerostipes* was also significantly enriched in IBD relative to NC (Figures 4G and 4H; FDR = 8.92×10^{-8}); our earlier-stage analyses revealed that *Anaerostipes* likewise increased progressively from stage 1 to stage 3 (Figure 3B; all pairwise FDR <0.05). Conversely, *Escherichia/Shigella* was also enriched in IBD (Figures 4G and 4H; FDR = 6.00×10^{-10}), yet our stage analyses revealed that its abundance decreased

(C) Each panel illustrates the relative abundance of one of the ten most abundant taxa (*x* axis, relative abundance of the indicated genus; *y* axis, relative frequency within the specified stage region).
(D–F) LEfSe identifies stage-associated microbial biomarkers in pairwise comparisons between the three IBD stages. The *x* axis indicates the LDA score, and the *y* axis lists the corresponding taxa. See also Data S3.

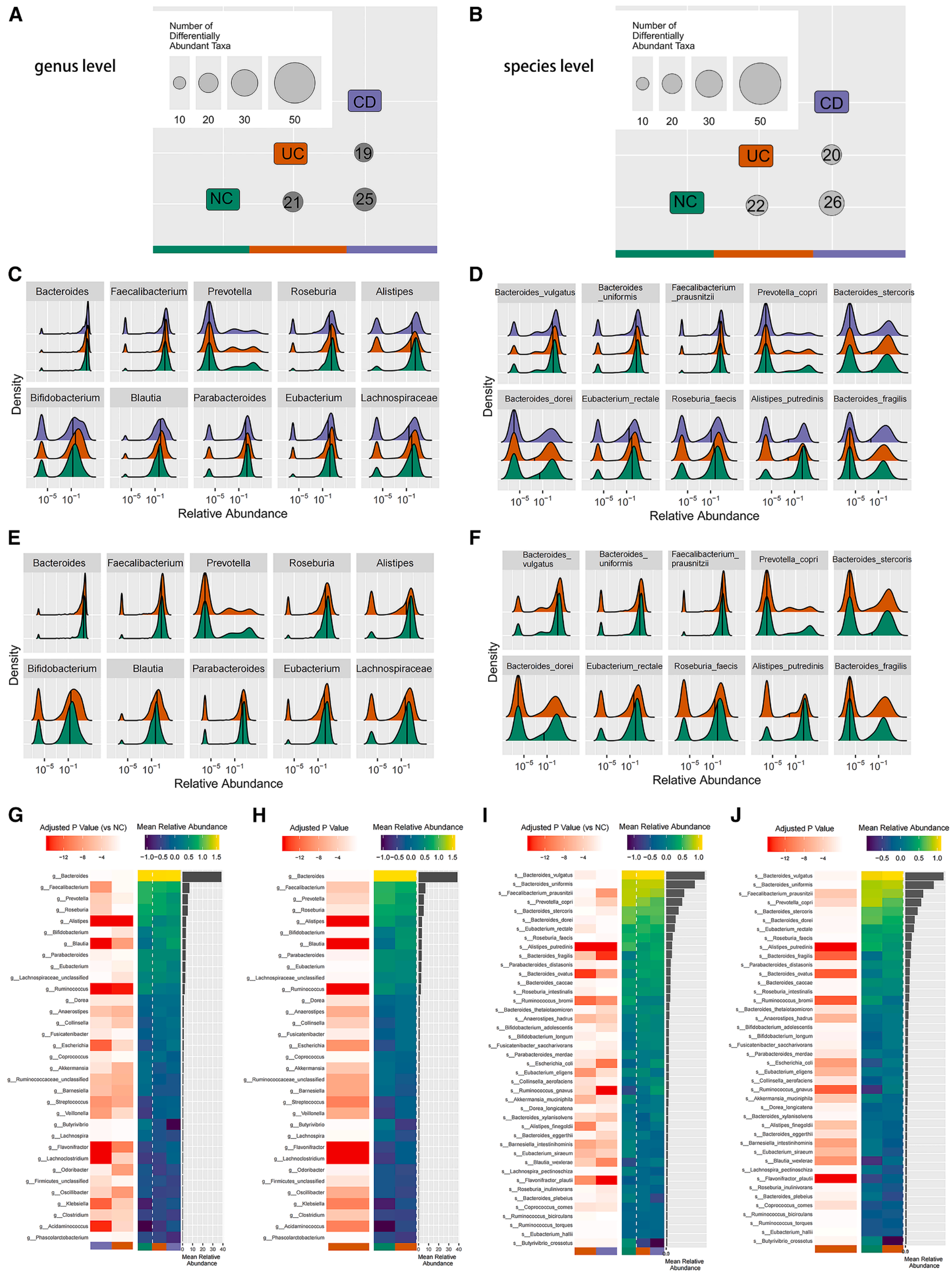

Figure 4. Systematic differences in microbiome composition between IBD and healthy individuals

(A) Differential abundance testing of genera among NC, UC, and CD groups.

(B) Differential abundance testing of species among NC, UC, and CD groups.

(C–F) Each panel illustrates the relative abundance of one of the ten most abundant genera (C and E) or species (D and F). In (C) and (D), each colored area shows the distribution of NC, UC, and CD samples, using the same color scheme as in (A). In (E) and (F), each colored region depicts the distribution of samples from IBD (yellow) and NC (green). The *x* axis represents the relative abundance, and the *y* axis shows the relative frequency.

(legend continued on next page)

CellPress
OPEN ACCESS

progressively from stage 1 to stage 3 (Figure 3B; all pairwise FDR <0.05). At the species level, *Blautia wexlerae* exhibited markedly enriched abundance in IBD relative to NC (Figure 4J; FDR = 1.16×10^{-9}). *Blautia wexlerae* abundance was also significantly elevated in both CD and UC relative to NC (Figure 4I; NC vs. CD: FDR = 9.17×10^{-11}; NC vs. UC: FDR = 4.31×10^{-5}), and CD displayed significantly higher abundance than UC (FDR = 2.58×10^{-2}). Similarly, *Anaerostipes hadrus* and *Escherichia coli* were also significantly enriched in IBD relative to NC (Figure 4J; FDR = 4.95×10^{-7} and FDR = 4.80×10^{-9}, respectively), with consistent enrichment in both CD and UC relative to NC (Figure 4I; all FDR <0.05). Together, these results indicate that *Blautia* and *Anaerostipes* exhibit individual-level enrichment across all IBD diagnostic categories (IBD, CD, and UC), while also showing population-level gradients aligned with the global disease burden (highest in stage 3), mirroring epidemiological trends. Further inspection of relative abundance distributions yielded additional insights. Among the top 10 most abundant genera and species, *Blautia*—previously identified as significantly enriched in IBD (NC vs. CD: FDR <2.2×10^{-16}; NC vs. UC: FDR = 4.66×10^{-9})—showed only 2.01% of NC samples exceeding 10% abundance, compared with 13.11% in CD and 6.28% in UC (Figure 4C). For *Blautia*, 2.01% of NC samples exceeded 10% relative abundance, compared with 10.27% of IBD samples (Figure 4E). Collectively, these case-control findings partially recapitulate well-established IBD-associated shifts: *Escherichia/Shigella* enrichment in our six metagenomic cohorts (n = 2,861), while *Blautia* and *Anaerostipes* enrichment in IBD contrasts with earlier reports that more commonly described its depletion in IBD and warrants further mechanistic investigation. Notably, combined with our country-level epidemiological stage gradient, these genera display directional concordance vs. reversal. *Blautia* and *Anaerostipes* were enriched in IBD and progressively increased from stage 1 to stage 3, whereas *Escherichia/Shigella* was enriched in IBD yet progressively decreased across the same gradient.

Functional profiling and pathway-level alterations of the gut microbiome in IBD

Beyond taxonomic and abundance-based analyses, shotgun metagenomic sequencing enabled a direct comparison of microbial functional potential between IBD and NC. Across all fecal samples, we identified 508 MetaCyc metabolic pathways, corresponding to 87 higher-level metabolic functions (Table S22). Of these, 16 differed significantly between IBD and NC (blocked Wilcoxon test, FDR <0.05; Figures 5A and 5B; Table S23). Functional composition was highly overlapping between NC and IBD, as well as across NC, CD, and UC, with a few categories accounting for most relative abundance. In both groups, "Others" was the largest fraction (IBD: 0.3863; NC: 0.3718), followed by proteinogenic amino acid biosynthesis (IBD: 0.1421; NC: 0.1451) and purine nucleotide biosynthesis (IBD: 0.1272; NC: 0.1327). Compared with NC, IBD samples showed higher mean relative abundance of super-pathways (0.01154 vs. 0.00921) and fatty acid biosynthesis (0.03308 vs. 0.03040). In contrast, multiple functions related to anabolic metabolism and genetic information processing were reduced in IBD, including purine nucleotide biosynthesis (0.12724 vs. 0.13275) and pyrimidine nucleotide biosynthesis (0.03395 vs. 0.03701).

To resolve these shifts at finer granularity, we performed pathway-level analyses for CD vs. NC and UC vs. NC. Among the 508 pathways, 73 differed significantly between IBD and NC, including 62 pathways in CD vs. NC and 58 pathways in UC vs. NC (blocked Wilcoxon test, FDR <0.05; Figure 5C; Table S24). A dominant and shared pattern across CD and UC was a coordinated reduction in anabolic capacity, mainly in amino acid biosynthesis and nucleotide metabolism. A smaller subset was enriched in IBD, including peptidoglycan maturation and adenosine/guanosine deoxyribonucleotide *de novo* biosynthesis II, suggesting enhanced microbial potential related to cell wall remodeling and deoxyribonucleotide supply in disease.

IBD-discriminatory species form a coherent interaction network

Building on the directionality-defined health-associated and IBD-associated taxa, we constructed an NC-only interaction network to test whether IBD-discriminatory species show an interpretable co-occurrence/mutual-exclusion architecture at a healthy baseline. We applied a stringent Spearman-Pearson concordance filter for robustness (STAR Methods; Table 25). Across 30 species nodes, 73 high-confidence edges passed all criteria, predominantly positive (63/73, 86.3%) with fewer negative associations (10/73, 13.7%), indicating that the taxa most informative for discriminating IBD (UC/CD) from controls are embedded in a largely co-varying backbone even in healthy controls. The structure was dominated by a few high-degree hubs, most notably *Coprococcus comes* (degree = 11). The strongest positive associations (by rho) defined a high-confidence core, including *Anaerostipes hadrus-Blautia wexlerae* (rho = 0.582; Spearman FDR <2.2×10^{-16}), with dense co-variation among *Firmicutes*-related taxa (e.g., *Roseburia intestinalis-Roseburia inulinivorans*, rho = 0.435; Spearman FDR <2.2×10^{-16}). Negative edges were fewer, with the strongest mutual exclusions largely involving *Flavonifractor plautii*, including *Coprococcus comes-Flavonifractor plautii* (rho = −0.350; Spearman FDR <2.2×10^{-16}). Thus, IBD-discriminatory species are not merely independent markers but form a coherent, predominantly cooperative covariance structure

(G and I) The red-white heatmaps display adjusted p values for genus-level (G) and species-level (I) differential abundance when CD and UC samples are compared with NC samples. The y axis lists all evaluated genera or species, and the x axis lists CD and UC (using the same color scale as in A). Each cell represents the strength of the differential abundance result for that taxon. The accompanying blue-green heatmaps depict the mean relative abundance (log10) of each taxon across NC, UC, and CD, as indicated on the x axis. Bar charts show the mean relative abundance.
(H and J) The red-white heat maps display adjusted p values for genus-level (H) and species-level (J) differential abundance when IBD samples are compared with NC samples. The y axis lists all evaluated taxa, and the x axis lists IBD (yellow). Each cell represents the strength of the differential abundance result for that taxon. The blue-green heatmaps show the mean relative abundance of each taxon across NC and IBD, as indicated on the x axis. Bar charts display the mean relative abundance. See also Data S4.

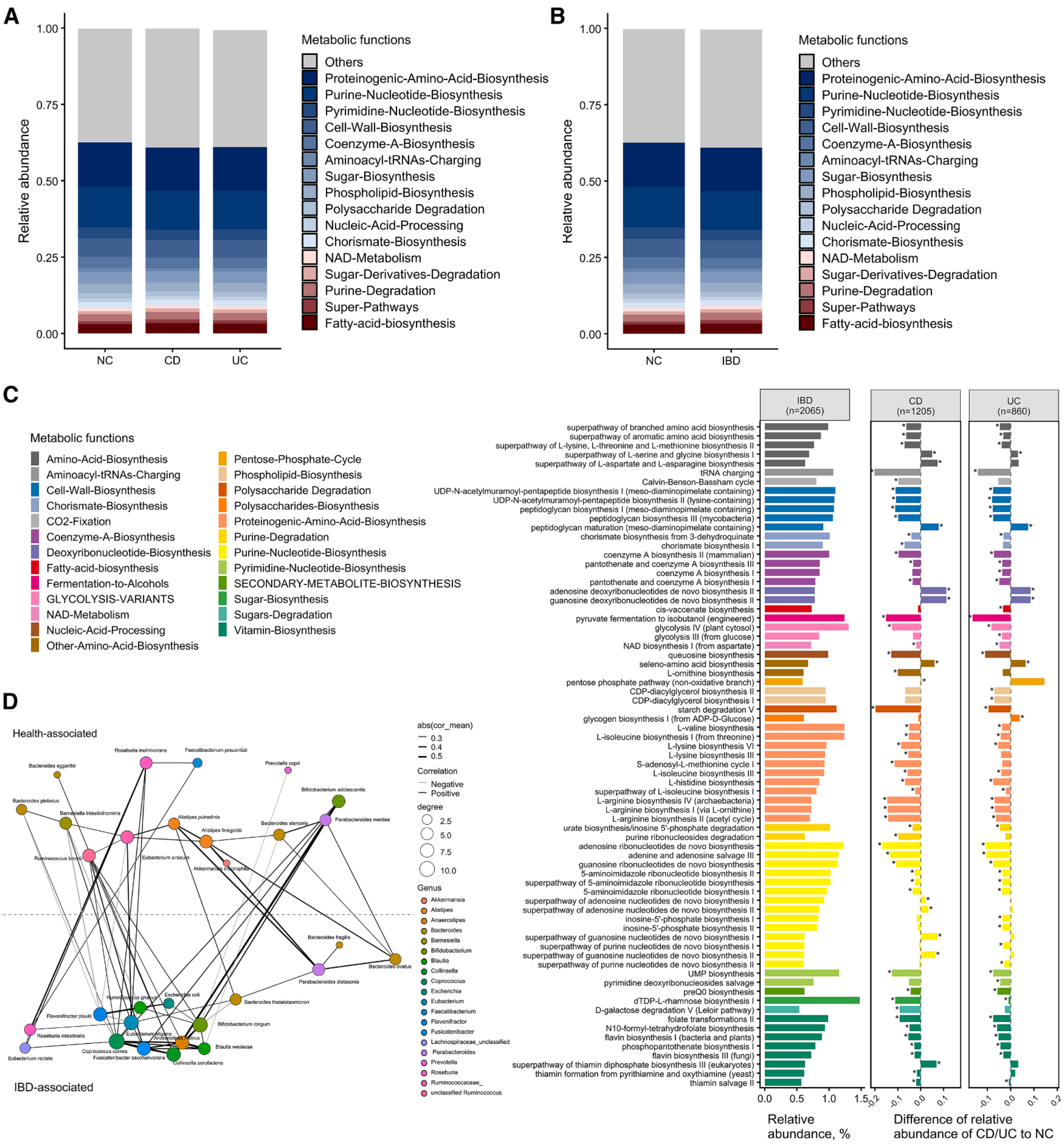

Figure 5. Gut microbial functional remodeling in IBD at metabolic function and pathway resolution

(A) Stacked bar plots showing mean relative abundance of MetaCyc-derived metabolic functions across NC, CD, and UC.

(B) Same as (A), with CD and UC pooled as IBD for an overall comparison against NC.

(C) Differential MetaCyc pathways between CD vs. NC and UC vs. NC. The left panel shows mean pathway relative abundance (%) in IBD (n = 2,065). The middle and right panels show the difference of relative abundance (case-NC) for CD (n = 1,205) and UC (n = 860).

(D) Species-level interaction network of IBD-discriminatory taxa inferred from healthy controls. Nodes represent species classified as health-associated or IBD-associated based on UC/CD vs. NC differential abundance. See also Data S5 and Data S8.

punctuated by fewer directional mutual exclusions, consistent with IBD-relevant taxa organizing into ecological modules (microbiome "ecotypes") whose coordinated dynamics could amplify disease-associated community-level effects (Figure 5D).

Gut microbiota as indicators of IBD

To identify microbiome-derived biomarkers associated with IBD, we examined the correlations between microbial genus-level abundances and disease status using a linear mixed model adjusted for age, country, sequencing batch, and median read length. In total, we identified 21 IBD-associated genera (excluding two unclassified genera) with an FDR below 0.05 (Figures 4H; Table S18). Among them, eight genera were enriched in controls, whereas thirteen were enriched in IBD patients. Next, we applied a random forest machine-learning algorithm to evaluate whether these genera could serve as a

CellPress
OPEN ACCESS

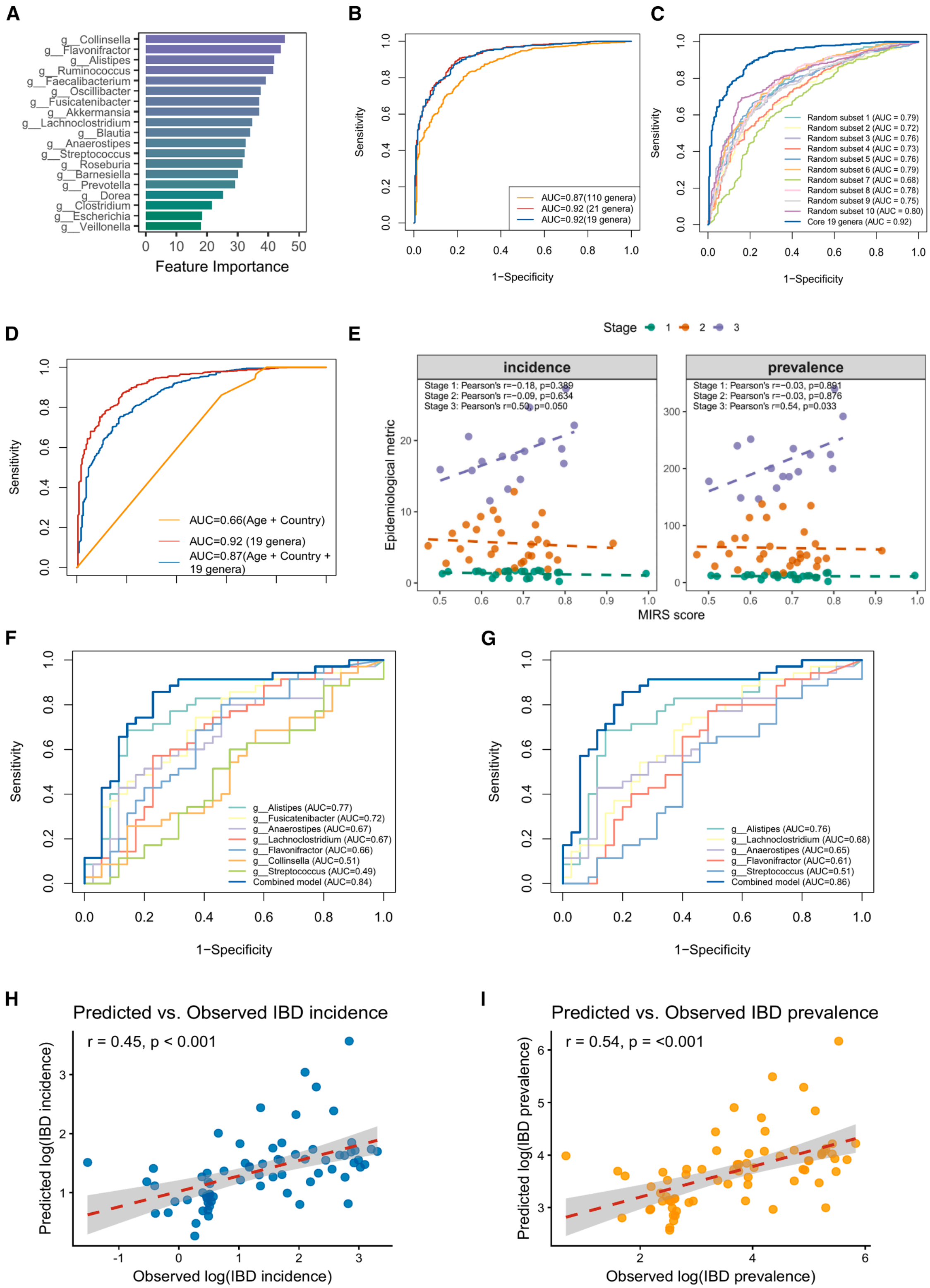

Figure 6. Global IBD risk prediction models based on core gut microbial features

(A) Feature importance analysis of the 19 key genera identified in the random forest model by 10-fold cross-validation.

(B) Receiver operating characteristic (ROC) curves of random forest models incorporating 110, 21, and 19 genera in distinguishing IBD from control samples in the validation dataset (n = 857).

(legend continued on next page)

microbiome-based classifier distinguishing IBD from healthy states. Of 2,861 total samples, 2,004 were assigned to the training dataset and 857 to the validation dataset. The random forest model incorporating all 21 genera achieved an area under the curve (AUC) of 0.92, outperforming the model using all 110 genera (AUC = 0.87). Through 10-fold cross-validation, we identified a refined model composed of 19 core genera (Figure 6A). The simplified 19-genera model exhibited equivalent discriminative accuracy (AUC = 0.92) to the full 21-genera model (Figure 6B), indicating that these core genera captured the essential microbial signals underlying IBD. This 19-genus classifier underpins all downstream analyses in the present study. At genus-level resolution, it matches the discriminative accuracy of the recent species-level UC (10-feature) and CD (9-feature) classifiers of Zheng et al.[33] (discovery AUC >0.90, validation AUC = 0.81, 95% confidence interval: 0.80–0.82). Genus-level features were chosen to permit cross-platform integration of 16S and shotgun cohorts across 70 countries, the scale required for the country-level epidemiological analyses developed below. Moreover, the 19-genera model significantly outperformed 10 random 19-genera subsets selected from the total genus pool (all $p < 0.05$; Figure 6C). Given that demographic variables such as age and country may also contribute to disease risk, we further compared the predictive performance of models integrating intrinsic factors (age, country) with microbial features. The model, including age and country alone, achieved an AUC of 0.66, whereas the model incorporating the 19 core genera achieved an AUC of 0.92. A full model combining both intrinsic and microbial factors reached an AUC of 0.87 (Figure 6D). However, this slightly lower value was likely influenced by sample imbalance across countries, where some regions contained only IBD or only control samples; such cohort characteristics are known to reduce the cross-study predictability of metagenomic random-forest classifiers.[34,35] Despite this imbalance, the 19-genera model retained robust performance under leave-one-country-out and leave-one-BioProject-out validation, with mean AUCs of 0.73 and 0.73, respectively (Figures S27 and S28; Tables 47 and 48). Together, these results indicate that the discriminative power of the 19 genera is largely independent of age and geographical factors, underscoring their independent predictive value in disease classification.

Based on the 19-genera random forest model, we develop a microbial inflammatory risk score (MIRS) to evaluate individual susceptibility to IBD. Across the global dataset, MIRS showed no significant association with overall IBD incidence or prevalence. However, in countries at the third epidemiological stage, where disease burden was highest, MIRS correlated positively with IBD prevalence ($r = 0.54$, $p = 0.03$). A log-linear regression analysis further revealed that a 1-SD increase in standardized MIRS was associated with an ~13% increase in IBD prevalence ($\beta = 0.12$, $p = 0.03$). These findings suggest that microbial structural features more strongly reflect disease risk in regions with stable epidemiological patterns and high disease burden. This cross-regional validation of MIRS provides a methodological framework for microbiome-based risk prediction and global IBD surveillance (Figures 6E; Table S26).

To further assess whether the relative abundances of the 19 core genera could predict national IBD incidence and prevalence, we performed LASSO regression analysis using microbiome data from 117,799 samples across 70 countries. The most predictive features identified by LASSO were subsequently used to construct multiple linear regression models for continuous outcomes (IBD incidence and prevalence). The final models, consisting of five core genera for incidence and seven core genera for prevalence (Table S27), effectively predicted both IBD incidence (Figure S22) and prevalence (Figure S23), with Pearson correlation coefficients between predicted and observed values of $r = 0.45$ ($p < 0.001$) and $r = 0.54$ ($p < 0.001$), respectively (Figures 6H and 6I; Table S28). Additionally, to evaluate the classification capacity of these microbial features in distinguishing countries with differing IBD risk levels, we dichotomized countries into "high-risk" and "low-risk" groups according to the median values of IBD incidence or prevalence. Logistic regression models based on LASSO-selected genera were then constructed to assess discriminatory performance. The AUC values of individual genera ranged from 0.49 to 0.77, while the combined multi-genera models achieved AUCs of 0.84 (prevalence-based classification) and 0.86 (incidence-based classification) (Figures 6F and 6G). These results demonstrate that the integrated microbial signature more accurately distinguishes between high- and low-IBD-risk countries, confirming the robustness and biological relevance of the core microbiome in explaining global epidemiological heterogeneity in IBD. Unlike earlier microbiome-based IBD diagnostic models, such as the species-level classifiers of Zheng et al. that predict an individual's disease status, the present analysis predicts the epidemiological burden of IBD at the country level.

(C) Comparison of the optimal 19-genera random forest model with ten random models constructed from randomly selected 19-genera subsets out of the total pool of 110 genera. Model performance was evaluated by AUC.

(D) Predictive performance of random forest models using intrinsic factors (age and country), the 19 core microbial genera, and their combination in differentiating IBD from healthy samples, with corresponding AUC values indicated.

(E) Correlations between the MIRS and IBD incidence and prevalence across countries representing different epidemiological stages.

(F) ROC curves of logistic regression models constructed using single genera or a combination of seven LASSO-selected genera to classify countries with high vs. low IBD prevalence.

(G) ROC curves of single- and multi-genera logistic regression models based on five LASSO-selected genera in classifying countries with high vs. low IBD incidence.

(H) Correlation between predicted and observed IBD incidence values derived from the multiple linear regression model based on five core genera identified by LASSO. Pearson's r and p values are indicated.

(I) Correlation between predicted and observed IBD prevalence values derived from the multiple linear regression model based on seven core genera identified by LASSO, with Pearson's r and p values indicated. See also Data S6.

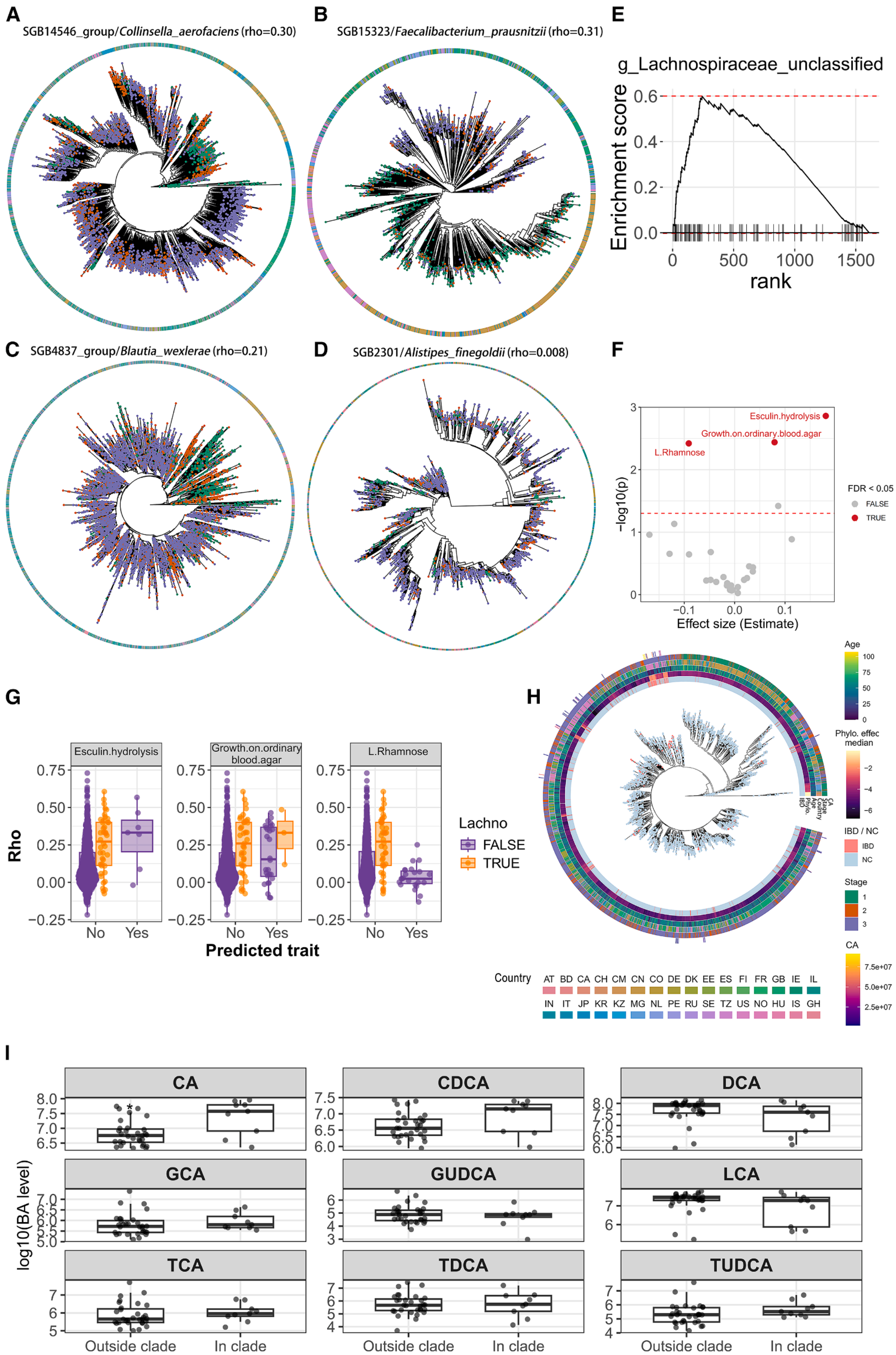

Figure 7. IBD stage-linked microbial clades exhibit distinct phylogenetic signals and bile acid profiles

(A–D) Representative SGB phylogenies selected based on their stage-specific patterns in IBD and differences in SGB abundance across stages, with annotations indicating the IBD stage from which samples were obtained.

(legend continued on next page)

Stage-resolved phylogenetic restructuring of gut microbial strains in IBD reveals an *Eisenbergiella* subclade linked to CA metabolism

We examined whether 21 species also exhibited IBD stage-specific intraspecies variability, aiming to determine whether epidemiological stage differences between samples correlated with greater phylogenetic distances among their respective microbial strains. We evaluated 32,152 gut metagenomes from 24,829 individuals (IBD: $n = 1,779$). Sixteen of the twenty-one species displayed significant associations (Tables S29 and S30; Mantel test, Pearson's correlation between stage distance and phylogenetic distance, FDR <0.05). Notably, four species-level genome bins (SGBs) showed clear IBD stage-specific clade structures: samples belonging to stage 1 and stage 3 formed multiple locally enriched subclades in the phylogenetic tree, suggesting the presence of relatively distinct strain groups detectable in both early and late disease stages. In contrast, stage 2 samples were more broadly scattered, without aggregation into specific clades. Stage 2 strain composition may represent a transitional state, containing sublineages resembling those from both stage 1 and stage 3. Among these taxa, SGB14546_group/*Collinsella aerofaciens*—previously reported to exhibit signatures of human co-adaptation[22] and strong geographical stratification[36]—together with SGB15323/*Faecalibacterium prausnitzii* displayed moderate Mantel-Pearson correlations (rho = 0.30 and 0.31, respectively; FDR <0.05), indicating stable associations between their phylogenetic differentiation and IBD stage. SGB4837_group/*Blautia wexlerae* (rho = 0.21, FDR <0.05) likewise showed significant stage dependence, suggesting stage-specific replacement or diversification of strains during IBD progression (Figures 7A–7C). Collectively, these findings indicate that IBD epidemiological staging stratifies not only microbial abundance and functional features but also deeper layers of intraspecies genetic variation. Conversely, several species, such as SGB2301/*Alistipes finegoldii*, showed little stage stratification (Figure 7D; rho = 0.008, FDR = 0.09). We next identified the taxonomic groups exhibiting the strongest IBD stage effects. A rank-based SGB enrichment analysis (SEA; STAR Methods) revealed that SGBs belonging to the *unclassified Lachnospiraceae group* tended to display elevated IBD stage effects (normalized enrichment score = 1.49, $p = 3.17 \times 10^{-5}$, FDR = 0.03) (Figure 7E; Table 31).

We then asked whether SGBs showing IBD stage structuring shared specific phenotypic traits (as predicted using Traitar[37]) or ecological properties. We identified three microbial phenotypes significantly associated with IBD stage stratification: esculin hydrolysis, growth on ordinary blood agar, and L-rhamnose utilization (Figure 7F; Table S32). Growth on ordinary blood agar was the only trait whose association with IBD stage was independent of membership in the unclassified Lachnospiraceae group; the increase in Rho among strains capable of growth on blood agar (estimate = 0.07, $p < 0.05$) suggests that this phenotype is robustly associated with stage stratification regardless of the strain's taxonomic background (Figure 7G; Table S33).

To further dissect whether IBD is associated with subspecies-level genetic variation, we evaluated 1,659 SGB-specific phylogenies using anpan[38] to fit phylogenetic generalized linear mixed models (PGLMM). Using age and country as covariates, and constructing IBD stage-stratified models, we identified two SGBs associated with IBD: *s__Firmicutes_bacterium_AF16_15* (elpd_diff = −4.1) and *s__Faecalibacterium_SGB15346* (elpd_diff = −4.3). Notably, both associations were detected exclusively in stage 3 (Table 34). Focusing on SGB4993 (*s__Firmicutes_bacterium_AF16_15*), GTDB-style phylogenetic annotation places this SGB within an *Eisenbergiella*-related clade (*s__Eisenbergiella* sp*900066775*). One subclade, enriched for IBD cases, was particularly abundant among younger US individuals (Figure 7H). The *AF16-15/sp900066775* genome (GCA_003461565.1) encodes multiple group II intron-like reverse transcriptases (RVT-GII/GIIM) and at least one diversity-generating retroelement-associated RT (RVT-DGR), indicating a genome rich in retrotransposition-associated mobile genetic modules and thus possessing high genetic plasticity. Among 40 HMP2 IBDMDB samples within the *Eisenbergiella* sp*900066775* phylogeny (9 within the IBD-enriched subclade and 31 outside), cholic acid (CA) was the only bile acid significantly elevated in the IBD-enriched subclade (BH-FDR = 0.030; linear mixed model; Figure 7I; Table S35). The CA association was robust to outlier influence: the 95% percentile and BCa bootstrap intervals were (1.88×10^6, 4.99×10^7) and (2.94×10^6, 5.09×10^7), respectively, both above zero. Leave-one-out refitting retained $p < 0.05$ in 40 of 40 iterations (Figure S37). Genome annotation of SGB4993 (GCA_003461565.1) recovered bile salt hydrolase (BSH) paralogs but no bai-operon core 7α-dehydroxylases; BSH was significantly lower in the IBD-enriched subclade ($p = 0.0091$; Figure S36; Table S58). Collectively, these data implicate the IBD-enriched *Eisenbergiella sp900066775* subclade in CA metabolism, supported by metabolomic, genomic, and gene quantification evidence.

DISCUSSION

Here, we integrated over 100,000 publicly available 16S rRNA gene amplicon sequencing samples from 482 BioProjects to

(E) Gene set enrichment analysis of the genus *Lachnospiraceae_unclassified*. The *x* axis indicates the rank of the IBD stage effect. The position of *Lachnospiraceae_unclassified* is highlighted in black.
(F) Volcano plot showing associations between Traitar-predicted microbial phenotypes and IBD stage effects. The *x* axis represents the estimated effect from the linear model; the *y* axis shows the corresponding *p* value after log10 transformation.
(G) Boxplot comparing the distribution of rho values across SGBs predicted to exhibit several features. Colors differentiate whether an SGB belongs to the genus *Lachnospiraceae_unclassified*.
(H) The phylogeny of SGB4993 (*Firmicutes bacterium AF16-15*) shows a clade predominantly composed of IBD individuals (tips in red) and another clade composed mainly of healthy individuals (tips in gray). Outer rings annotate the phylogenetic signal (phylo effect median) obtained from the anpan model, age, country, IBD stage, and the relative abundance of CA.
(I) In the clade enriched for IBD patients highlighted in (H), samples showed higher levels of CA compared with those outside the clade (samples derived from participants in the HMP_IBDMDB_2019 cohort). See also Data S7.

link country-level gut microbial ecologies across 70 countries with their corresponding IBD burden, thereby constructing a stage-based microbial framework for IBD to evaluate global variation of the human gut microbiome across different stages of the disease.

We observed a progressive decline in microbial α-diversity from stage 1 to stage 3, supporting the notion that IBD progression is accompanied by intestinal dysbiosis. At the level of β-diversity, samples from stages 1, 2, and 3 occupied clearly separable regions in ordination space. Gut microbial communities segregate into distinct niches in β-diversity ordination space.[32] Samples from North America and parts of Europe were predominantly assigned to IBD stage 3, whereas non-Western countries were more frequently classified as stage 1 or stage 2. This suggests that the proposed staging framework partially captures lifestyle-associated structuring of gut microbial communities at the population level.

The principal conceptual contribution of this work, beyond confirming established IBD case-control patterns, is the cross-scale framework that integrates individual-level disease signal with country-level epidemiologic stage gradient and explicitly resolves the directional concordance vs. reversal of taxon-level signals across the two scales. For example, *Escherichia/Shigella* was enriched in IBD relative to controls, yet decreased progressively from stage 1 to stage 3 along the country-level epidemiological gradient. A plausible interpretation is that the IBD enrichment reflects the inflammation-driven expansion of *facultative anaerobes* such as *Escherichia/Shigella* within the dysbiotic gut,[5,39] whereas the country-level decline from stage 1 to stage 3 likely reflects lower environmental exposure to *Escherichia/Shigella* in higher-income settings with improved sanitation infrastructure.[40] Conversely, *Blautia* and *Anaerostipes* exhibited concordant enrichment in IBD individuals and progressive increase from stage 1 to stage 3, suggesting that these taxa share common host and ecological drivers across individual and population scales. Notably, our observation of *Blautia*[41,42] and *Anaerostipes*[43] enrichment in IBD diverges from earlier reports of their depletion in IBD; this enrichment was observed in our integrated six-cohort dataset (n = 2,861) after mixed-effects adjustment for age, country, project, and median read length and requires confirmation in independent cohorts and additional geographical settings. Functional profiling further supported the ecological distinction between IBD and healthy states. We observed a systematic depletion of microbial biosynthetic capacity in IBD, including pathways related to amino acid biosynthesis and nucleotide metabolism, alongside enrichment of fatty acid biosynthesis and sugar-derivative degradation. Building on these compositional signatures, we using a random forest machine-learning model, developed the MIRS to distinguish healthy and IBD participants and accurately predict national-level IBD prevalence in stage 3 countries. This pattern suggests that once countries enter a high-burden, relatively stable epidemiological stage, population-level microbiome configurations become more tightly aligned with disease burden. These findings highlight the necessity of stage-aware calibration strategies when attempting to infer epidemiological patterns from microbiome summary metrics, rather than applying a single global mapping.

Strain-level phylogenetic analyses provided an evolutionary perspective consistent with the stage structure. The varying degrees of phylogeny-geography correlation observed across different microbial species suggest the influence of both environmental selection and isolation by distance, with their relative effects varying depending on the microbial species. Our results further demonstrate that microbial strains exhibit pronounced stage-associated variation, with phylogenetic distances among conspecific strains correlating with host IBD stage distances. These patterns may arise from isolation by distance and subsequent genetic drift,[44,45] environmental selection, or their combined effects. Within this context, we identified an *Eisenbergiella* subclade associated with CA levels that was prevalent in IBD patients but rare in healthy individuals. The identification of this subclade links bile acid metabolism to IBD-associated strain-level phylogenetic structure, thereby extending the current understanding of microbial evolutionary patterns in IBD.

This study constructed a staged microbial framework for IBD on a global scale using large-scale public data. It reveals the diversity patterns of gut microbiota across different stages of IBD in various regions worldwide and demonstrates how microbiome profiles can predict IBD prevalence in high-burden regions. It also emphasizes the complex relationship between IBD and within-species genetic variation of the gut microbiota at different stages of the disease.

Limitations of the study

First, individual-level lifestyle indicators such as BMI, diet, medication history, and antibiotic exposure are inconsistently reported across public 16S cohorts. Despite adjustment for sex, age group, disease and SDI, residual confounding between epidemiologic stage and Westernized-lifestyle exposures cannot be ruled out. The stage-associated microbial shifts should, therefore, be interpreted as a joint signal of IBD burden and broader lifestyle gradient. In parallel, reference databases under-represent species from non-European and non-American populations, which may bias stage-associated taxonomic features toward those observable in Western-derived genomes.

RESOURCE AVAILABILITY

Lead contact

Requests for further information and resources should be directed to and will be fulfilled by the lead contact, Cong Dai (cdai@cmu.edu.cn).

Materials availability

This study did not generate new unique reagents.

Data and code availability

- This paper analyzes existing, publicly available data, accessible at NCBI BioProject and curatedMetagenomicData. Accession numbers and cohort identifiers are listed in the key resources table.
- All original code has been deposited at Zenodo (https://doi.org/10.5281/zenodo.20390342) and GitHub (https://github.com/zhai199887/Inflammatory-Bowel-Disease) and is publicly available as of the date of publication.
- Any additional information required to reanalyze the data reported in this paper is available from the lead contact upon request.

ACKNOWLEDGMENTS

The authors would like to thank all the researchers for generously sharing their sequencing data included in this study. We acknowledge funding from the National Natural Science Foundation of China (grant Zendo numbers 82270571 and 82570632 to C.D.). The graphical abstract was created using BioRender (https://biorender.com).

AUTHOR CONTRIBUTIONS

C.D. conceived and designed the study. J.Z. wrote the first draft. Y.L., J.L., X.S., R.C., D.Z., J.Y., and Y.S. critically revised the manuscript. All authors reviewed and approved the final version before submission.

DECLARATION OF INTERESTS

The authors declare no competing interests.

STAR★METHODS

Detailed methods are provided in the online version of this paper and include the following:

- KEY RESOURCES TABLE
- EXPERIMENTAL MODEL AND STUDY PARTICIPANT DETAILS
  - 16S rRNA amplicon compendium
  - Shotgun metagenomic cohorts
  - MetaPhlAn4 SGB resource
- METHOD DETAILS
  - IBD three-stage classification
  - Sample selection and preprocessing
  - Calculation of Shannon diversity
  - Rarefaction analysis and phylum discovery rate
  - Between-country and between-stage alpha-diversity comparison
  - Rarefaction-based alpha-diversity across countries and IBD stages
  - Principal coordinates analysis
  - Cluster evaluation of the three IBD stages
  - PERMDISP analysis of stage dispersion
  - Diversity and clustering analyses on an IBD-only subset
  - Differential abundance analysis
- COUNTRY-LEVEL PHYLUM DISCOVERY RATE
  - IBD stage region signature determination
  - Aggregation to country-level taxonomic profiles
  - Public cohorts of patients with IBD and normal controls
  - Functional profiling and pathway-level differential analysis
  - Strain-strain interaction network
  - Random forest model for the MIRS
  - LASSO-based feature selection and multiple linear regression modeling
  - IBD stage association with microbial phylogeny
  - Microbial characteristics and IBD stage effect
  - Phylogenetic generalized mixed models in anpan
  - Enrichment of taxonomic levels
  - Bile-acid association and robustness analyses in the SGB4993 phylogeny
  - Functional validation of bile-acid metabolism in SGB4993
- QUANTIFICATION AND STATISTICAL ANALYSIS

SUPPLEMENTAL INFORMATION

Supplemental information can be found online at https://doi.org/10.1016/j.xcrm.2026.102974.

Received: January 6, 2026
Revised: June 1, 2026
Accepted: July 10, 2026
Published: August 10, 2026

REFERENCES

1. Morgan, X.C., Tickle, T.L., Sokol, H., Gevers, D., Devaney, K.L., Ward, D.V., Reyes, J.A., Shah, S.A., LeLeiko, N., Snapper, S.B., et al. (2012). Dysfunction of the intestinal microbiome in inflammatory bowel disease and treatment. Genome Biol. *13*, R79. https://doi.org/10.1186/gb-2012-13-9-r79.
2. Franzosa, E.A., Sirota-Madi, A., Avila-Pacheco, J., Fornelos, N., Haiser, H.J., Reinker, S., Vatanen, T., Hall, A.B., Mallick, H., McIver, L.J., et al. (2018). Gut microbiome structure and metabolic activity in inflammatory bowel disease. Nat. Microbiol. *4*, 293–305. https://doi.org/10.1038/s41564-018-0306-4.
3. Lewis, J.D., Chen, E.Z., Baldassano, R.N., Otley, A.R., Griffiths, A.M., Lee, D., Bittinger, K., Bailey, A., Friedman, E.S., Hoffmann, C., et al. (2015). Inflammation, Antibiotics, and Diet as Environmental Stressors of the Gut Microbiome in Pediatric Crohn's Disease. Cell Host Microbe *18*, 489–500. https://doi.org/10.1016/j.chom.2015.09.008.
4. Gevers, D., Kugathasan, S., Denson, L.A., Vázquez-Baeza, Y., Van Treuren, W., Ren, B., Schwager, E., Knights, D., Song, S.J., Yassour, M., et al. (2014). The treatment-naive microbiome in new-onset Crohn's disease. Cell Host Microbe *15*, 382–392. https://doi.org/10.1016/j.chom.2014.02.005.
5. Lloyd-Price, J., Arze, C., Ananthakrishnan, A.N., Schirmer, M., Avila-Pacheco, J., Poon, T.W., Andrews, E., Ajami, N.J., Bonham, K.S., Brislawn, C.J., et al. (2019). Multi-omics of the gut microbial ecosystem in inflammatory bowel diseases. Nature *569*, 655–662. https://doi.org/10.1038/s41586-019-1237-9.
6. Nielsen, H.B., Almeida, M., Juncker, A.S., Rasmussen, S., Li, J., Sunagawa, S., Plichta, D.R., Gautier, L., Pedersen, A.G., Le Chatelier, E., et al. (2014). Identification and assembly of genomes and genetic elements in complex metagenomic samples without using reference genomes. Nat. Biotechnol. *32*, 822–828. https://doi.org/10.1038/nbt.2939.
7. He, Q., Gao, Y., Jie, Z., Yu, X., Laursen, J.M., Xiao, L., Li, Y., Li, L., Zhang, F., Feng, Q., et al. (2017). Two distinct metacommunities characterize the gut microbiota in Crohn's disease patients. GigaScience *6*, gix050. https://doi.org/10.1093/gigascience/gix050.
8. Schirmer, M., Garner, A., Vlamakis, H., and Xavier, R.J. (2019). Microbial genes and pathways in inflammatory bowel disease. Nat. Rev. Microbiol. *17*, 497–511. https://doi.org/10.1038/s41579-019-0213-6.
9. Fischbach, M.A. (2018). Microbiome: Focus on Causation and Mechanism. Cell *174*, 785–790. https://doi.org/10.1016/j.cell.2018.07.038.
10. Goodrich, J.K., Waters, J.L., Poole, A.C., Sutter, J.L., Koren, O., Blekhman, R., Beaumont, M., Van Treuren, W., Knight, R., Bell, J.T., et al. (2014). Human genetics shape the gut microbiome. Cell *159*, 789–799. https://doi.org/10.1016/j.cell.2014.09.053.
11. Brooks, A.W., Priya, S., Blekhman, R., and Bordenstein, S.R. (2018). Gut microbiota diversity across ethnicities in the United States. PLoS Biol. *16*, e2006842. https://doi.org/10.1371/journal.pbio.2006842.
12. Deschasaux, M., Bouter, K.E., Prodan, A., Levin, E., Groen, A.K., Herrema, H., Tremaroli, V., Bakker, G.J., Attaye, I., Pinto-Sietsma, S.J., et al. (2018). Depicting the composition of gut microbiota in a population with varied ethnic origins but shared geography. Nat. Med. *24*, 1526–1531. https://doi.org/10.1038/s41591-018-0160-1.
13. Mallott, E.K., Sitarik, A.R., Leve, L.D., Cioffi, C., Camargo, C.A., Jr., Hasegawa, K., and Bordenstein, S.R. (2023). Human microbiome variation associated with race and ethnicity emerges as early as 3 months of age. PLoS Biol. *21*, e3002230. https://doi.org/10.1371/journal.pbio.3002230.
14. De Filippo, C., Cavalieri, D., Di Paola, M., Ramazzotti, M., Poullet, J.B., Massart, S., Collini, S., Pieraccini, G., and Lionetti, P. (2010). Impact of diet in shaping gut microbiota revealed by a comparative study in children from Europe and rural Africa. Proc. Natl. Acad. Sci. USA *107*, 14691–14696. https://doi.org/10.1073/pnas.1005963107.
15. Langdon, A., Crook, N., and Dantas, G. (2016). The effects of antibiotics on the microbiome throughout development and alternative approaches for

therapeutic modulation. Genome Med. *8*, 39. https://doi.org/10.1186/s13073-016-0294-z.

16. Vangay, P., Johnson, A.J., Ward, T.L., Al-Ghalith, G.A., Shields-Cutler, R.R., Hillmann, B.M., Lucas, S.K., Beura, L.K., Thompson, E.A., Till, L.M., et al. (2018). US Immigration Westernizes the Human Gut Microbiome. Cell *175*, 962–972.e10. https://doi.org/10.1016/j.cell.2018.10.029.
17. Le Bastard, Q., Vangay, P., Batard, E., Knights, D., and Montassier, E. (2020). US Immigration Is Associated With Rapid and Persistent Acquisition of Antibiotic Resistance Genes in the Gut. Clin. Infect. Dis. *71*, 419–421. https://doi.org/10.1093/cid/ciz1087.
18. Peters, B.A., Yi, S.S., Beasley, J.M., Cobbs, E.N., Choi, H.S., Beggs, D.B., Hayes, R.B., and Ahn, J. (2020). US nativity and dietary acculturation impact the gut microbiome in a diverse US population. ISME J. *14*, 1639–1650. https://doi.org/10.1038/s41396-020-0630-6.
19. Hracs, L., Windsor, J.W., Gorospe, J., Cummings, M., Coward, S., Buie, M.J., Quan, J., Goddard, Q., Caplan, L., Markovinović, A., et al. (2025). Global evolution of inflammatory bowel disease across epidemiologic stages. Nature *642*, 458–466. https://doi.org/10.1038/s41586-025-08940-0.
20. Yan, Y., Nguyen, L.H., Franzosa, E.A., and Huttenhower, C. (2020). Strain-level epidemiology of microbial communities and the human microbiome. Genome Med. *12*, 71. https://doi.org/10.1186/s13073-020-00765-y.
21. Blanco-Míguez, A., Beghini, F., Cumbo, F., McIver, L.J., Thompson, K.N., Zolfo, M., Manghi, P., Dubois, L., Huang, K.D., Thomas, A.M., et al. (2023). Extending and improving metagenomic taxonomic profiling with uncharacterized species using MetaPhlAn 4. Nat. Biotechnol. *41*, 1633–1644. https://doi.org/10.1038/s41587-023-01688-w.
22. Suzuki, T.A., Fitzstevens, J.L., Schmidt, V.T., Enav, H., Huus, K.E., Mbong Ngwese, M., Grießhammer, A., Pfleiderer, A., Adegbite, B.R., Zinsou, J.F., et al. (2022). Codiversification of gut microbiota with humans. Science *377*, 1328–1332. https://doi.org/10.1126/science.abm7759.
23. Karcher, N., Pasolli, E., Asnicar, F., Huang, K.D., Tett, A., Manara, S., Armanini, F., Bain, D., Duncan, S.H., Louis, P., et al. (2020). Analysis of 1321 Eubacterium rectale genomes from metagenomes uncovers complex phylogeographic population structure and subspecies functional adaptations. Genome Biol. *21*, 138. https://doi.org/10.1186/s13059-020-02042-y.
24. Vatanen, T., Ang, Q.Y., Siegwald, L., Sarker, S.A., Le Roy, C.I., Duboux, S., Delannoy-Bruno, O., Ngom-Bru, C., Boulangé, C.L., Stražar, M., et al. (2022). A distinct clade of Bifidobacterium longum in the gut of Bangladeshi children thrives during weaning. Cell *185*, 4280–4297.e12. https://doi.org/10.1016/j.cell.2022.10.011.
25. Shridhar, S.V., Beghini, F., Alexander, M., Singh, A., Juárez, R.M., Brito, I.L., and Christakis, N.A. (2024). Environmental, socioeconomic, and health factors associated with gut microbiome species and strains in isolated Honduras villages. Cell Rep. *43*, 114442. https://doi.org/10.1016/j.celrep.2024.114442.
26. Zahavi, L., Lavon, A., Reicher, L., Shoer, S., Godneva, A., Leviatan, S., Rein, M., Weissbrod, O., Weinberger, A., and Segal, E. (2023). Bacterial SNPs in the human gut microbiome associate with host BMI. Nat. Med. *29*, 2785–2792. https://doi.org/10.1038/s41591-023-02599-8.
27. Hall, A.B., Yassour, M., Sauk, J., Garner, A., Jiang, X., Arthur, T., Lagoudas, G.K., Vatanen, T., Fornelos, N., Wilson, R., et al. (2017). A novel Ruminococcus gnavus clade enriched in inflammatory bowel disease patients. Genome Med. *9*, 103. https://doi.org/10.1186/s13073-017-0490-5.
28. Lee, J.W.J., Plichta, D., Hogstrom, L., Borren, N.Z., Lau, H., Gregory, S.M., Tan, W., Khalili, H., Clish, C., Vlamakis, H., et al. (2021). Multi-omics reveal microbial determinants impacting responses to biologic therapies in inflammatory bowel disease. Cell Host Microbe *29*, 1294–1304.e4. https://doi.org/10.1016/j.chom.2021.06.019.
29. Shaw, K.A., Bertha, M., Hofmekler, T., Chopra, P., Vatanen, T., Srivatsa, A., Prince, J., Kumar, A., Sauer, C., Zwick, M.E., et al. (2016). Dysbiosis, inflammation, and response to treatment: a longitudinal study of pediatric subjects with newly diagnosed inflammatory bowel disease. Genome Med. *8*, 75. https://doi.org/10.1186/s13073-016-0331-y.
30. Zeevi, D., Korem, T., Zmora, N., Israeli, D., Rothschild, D., Weinberger, A., Ben-Yacov, O., Lador, D., Avnit-Sagi, T., Lotan-Pompan, M., et al. (2015). Personalized Nutrition by Prediction of Glycemic Responses. Cell *163*, 1079–1094. https://doi.org/10.1016/j.cell.2015.11.001.
31. Ning, L., Zhou, Y.L., Sun, H., Zhang, Y., Shen, C., Wang, Z., Xuan, B., Zhao, Y., Ma, Y., Yan, Y., et al. (2023). Microbiome and metabolome features in inflammatory bowel disease via multi-omics integration analyses across cohorts. Nat. Commun. *14*, 7135. https://doi.org/10.1038/s41467-023-42788-0.
32. Abdill, R.J., Graham, S.P., Rubinetti, V., Ahmadian, M., Hicks, P., Chetty, A., McDonald, D., Ferretti, P., Gibbons, E., Rossi, M., et al. (2025). Integration of 168,000 samples reveals global patterns of the human gut microbiome. Cell *188*, 1100–1118.e17. https://doi.org/10.1016/j.cell.2024.12.017.
33. Zheng, J., Sun, Q., Zhang, M., Liu, C., Su, Q., Zhang, L., Xu, Z., Lu, W., Ching, J., Tang, W., et al. (2024). Noninvasive, microbiome-based diagnosis of inflammatory bowel disease. Nat. Med. *30*, 3555–3567. https://doi.org/10.1038/s41591-024-03280-4.
34. Pasolli, E., Truong, D.T., Malik, F., Waldron, L., and Segata, N. (2016). Machine Learning Meta-analysis of Large Metagenomic Datasets: Tools and Biological Insights. PLoS Comput. Biol. *12*, e1004977. https://doi.org/10.1371/journal.pcbi.1004977.
35. Thomas, A.M., Manghi, P., Asnicar, F., Pasolli, E., Armanini, F., Zolfo, M., Beghini, F., Manara, S., Karcher, N., Pozzi, C., et al. (2019). Metagenomic analysis of colorectal cancer datasets identifies cross-cohort microbial diagnostic signatures and a link with choline degradation. Nat. Med. *25*, 667–678. https://doi.org/10.1038/s41591-019-0405-7.
36. Andreu-Sánchez, S., Blanco-Míguez, A., Wang, D., Golzato, D., Manghi, P., Heidrich, V., Fackelmann, G., Zhernakova, D.V., Kurilshikov, A., Valles-Colomer, M., et al. (2025). Global genetic diversity of human gut microbiome species is related to geographic location and host health. Cell *188*, 3942–3959.e9. https://doi.org/10.1016/j.cell.2025.04.014.
37. Weimann, A., Mooren, K., Frank, J., Pope, P.B., Bremges, A., and McHardy, A.C. (2016). From Genomes to Phenotypes: Traitar, the Microbial Trait Analyzer. mSystems *1*, e00101-16. https://doi.org/10.1128/mSystems.00101-16.
38. Ghazi, A.R., Thompson, K.N., Bhosle, A., Mei, Z., Yan, Y., Wang, F., Wang, K., Franzosa, E.A., and Huttenhower, C. (2025). Quantifying Metagenomic Strain Associations from Microbiomes with Anpan. Preprint at bioRxiv. https://doi.org/10.1101/2025.01.06.631550.
39. Winter, S.E., Winter, M.G., Xavier, M.N., Thiennimitr, P., Poon, V., Keestra, A.M., Laughlin, R.C., Gomez, G., Wu, J., Lawhon, S.D., et al. (2013). Host-derived nitrate boosts growth of E. coli in the inflamed gut. Science *339*, 708–711. https://doi.org/10.1126/science.1232467.
40. Sonnenburg, E.D., and Sonnenburg, J.L. (2019). The ancestral and industrialized gut microbiota and implications for human health. Nat. Rev. Microbiol. *17*, 383–390. https://doi.org/10.1038/s41579-019-0191-8.
41. Schirmer, M., Denson, L., Vlamakis, H., Franzosa, E.A., Thomas, S., Gotman, N.M., Rufo, P., Baker, S.S., Sauer, C., Markowitz, J., et al. (2018). Compositional and Temporal Changes in the Gut Microbiome of Pediatric Ulcerative Colitis Patients Are Linked to Disease Course. Cell Host Microbe *24*, 600–610.e4. https://doi.org/10.1016/j.chom.2018.09.009.
42. Gonzalez, C.G., Mills, R.H., Zhu, Q., Sauceda, C., Knight, R., Dulai, P.S., and Gonzalez, D.J. (2022). Location-specific signatures of Crohn's disease at a multi-omics scale. Microbiome *10*, 133. https://doi.org/10.1186/s40168-022-01331-x.
43. Pascal, V., Pozuelo, M., Borruel, N., Casellas, F., Campos, D., Santiago, A., Martinez, X., Varela, E., Sarrabayrouse, G., Machiels, K., et al. (2017). A microbial signature for Crohn's disease. Gut *66*, 813–822. https://doi.org/10.1136/gutjnl-2016-313235.
44. Martiny, J.B.H., Bohannan, B.J.M., Brown, J.H., Colwell, R.K., Fuhrman, J.A., Green, J.L., Horner-Devine, M.C., Kane, M., Krumins, J.A., Kuske, C.R., et al. (2006). Microbial biogeography: putting microorganisms on the map. Nat. Rev. Microbiol. *4*, 102–112. https://doi.org/10.1038/nrmicro1341.

45. Moeller, A.H., Suzuki, T.A., Lin, D., Lacey, E.A., Wasser, S.K., and Nachman, M.W. (2017). Dispersal limitation promotes the diversification of the mammalian gut microbiota. Proc. Natl. Acad. Sci. USA *114*, 13768–13773. https://doi.org/10.1073/pnas.1700122114.

46. GBD 2021 Diseases and Injuries Collaborators (2024). Global incidence, prevalence, years lived with disability (YLDs), disability-adjusted life-years (DALYs), and healthy life expectancy (HALE) for 371 diseases and injuries in 204 countries and territories and 811 subnational locations, 1990-2021: a systematic analysis for the Global Burden of Disease Study 2021. Lancet *403*, 2133–2161. https://doi.org/10.1016/s0140-6736(24)00757-8.

47. Vich Vila, A., Imhann, F., Collij, V., Jankipersadsing, S.A., Gurry, T., Mujagic, Z., Kurilshikov, A., Bonder, M.J., Jiang, X., Tigchelaar, E.F., et al. (2018). Gut microbiota composition and functional changes in inflammatory bowel disease and irritable bowel syndrome. Sci. Transl. Med. *10*, eaap8914. https://doi.org/10.1126/scitranslmed.aap8914.

48. Ijaz, U.Z., Quince, C., Hanske, L., Loman, N., Calus, S.T., Bertz, M., Edwards, C.A., Gaya, D.R., Hansen, R., McGrogan, P., et al. (2017). The distinct features of microbial 'dysbiosis' of Crohn's disease do not occur to the same extent in their unaffected, genetically-linked kindred. PLoS One *12*, e0172605. https://doi.org/10.1371/journal.pone.0172605.

49. Li, J., Jia, H., Cai, X., Zhong, H., Feng, Q., Sunagawa, S., Arumugam, M., Kultima, J.R., Prifti, E., Nielsen, T., et al. (2014). An integrated catalog of reference genes in the human gut microbiome. Nat. Biotechnol. *32*, 834–841. https://doi.org/10.1038/nbt.2942.

50. Muller, E., Algavi, Y.M., and Borenstein, E. (2022). The gut microbiome-metabolome dataset collection: a curated resource for integrative meta-analysis. NPJ Biofilms Microbiomes *8*, 79. https://doi.org/10.1038/s41522-022-00345-5.

51. Mistry, J., Chuguransky, S., Williams, L., Qureshi, M., Salazar, G.A., Sonnhammer, E.L.L., Tosatto, S.C.E., Paladin, L., Raj, S., Richardson, L.J., et al. (2021). Pfam: The protein families database in 2021. Nucleic Acids Res. *49*, D412–D419. https://doi.org/10.1093/nar/gkaa913.

52. Caspi, R., Billington, R., Keseler, I.M., Kothari, A., Krummenacker, M., Midford, P.E., Ong, W.K., Paley, S., Subhraveti, P., and Karp, P.D. (2020). The MetaCyc database of metabolic pathways and enzymes - a 2019 update. Nucleic Acids Res. *48*, D445–D453. https://doi.org/10.1093/nar/gkz862.

53. Oksanen, J. (2011). vegan: Community Ecology Package-R package version 1.17-8. http://CRAN.R-project.org/package=vegan.

54. Kassambara, A. (2019). Rstatix: Pipe-Friendly Framework for Basic Statistical Tests (CRAN).

55. Bates, D., Mächler, M., Bolker, B., and Walker, S. (2015). Fitting Linear Mixed-Effects Models Using lme4. J. Stat. Softw. *67*, 1–48. https://doi.org/10.18637/jss.v067.i01.

56. Kuznetsova, A., Brockhoff, P.B., and Christensen, R.H.B. (2017). lmerTest Package: Tests in Linear Mixed Effects Models. J. Stat. Softw. *82*, 1–26. https://doi.org/10.18637/jss.v082.i13.

57. Delicado, P., and Pachón-García, C. (2025). Multidimensional scaling for big data. Adv. Data. Anal. Classif. *19*, 649–670. https://doi.org/10.1007/s11634-024-00591-9.

58. Dudek, A., and Walesiak, M. (2020). The choice of variable normalization method in cluster analysis. In Proceeding of the 35th International Business Information Management Association Conference (IBIMA), pp. 325–340.

59. Hothorn, T., Hornik, K., Wiel, M.A.v.d., and Zeileis, A. (2008). Implementing a class of permutation tests: the coin package. J. Stat. Softw. *28*, 1–23. https://doi.org/10.18637/jss.v028.i08.

60. Liaw, A., and Wiener, M. (2002). Classification and regression by randomForest. R. News 2, 18–22.

61. Kuhn, M. (2008). Building Predictive Models in R Using the caret Package. J. Stat. Softw. *28*, 1–26. https://doi.org/10.18637/jss.v028.i05.

62. Robin, X., Turck, N., Hainard, A., Tiberti, N., Lisacek, F., Sanchez, J.C., and Müller, M. (2011). pROC: an open-source package for R and S+ to analyze and compare ROC curves. BMC Bioinf. *12*, 77. https://doi.org/10.1186/1471-2105-12-77.

63. Paradis, E., and Schliep, K. (2019). ape 5.0: an environment for modern phylogenetics and evolutionary analyses in R. Bioinformatics *35*, 526–528. https://doi.org/10.1093/bioinformatics/bty633.

64. Korotkevich, G., Sukhov, V., Budin, N., Shpak, B., Artyomov, M.N., and Sergushichev, A. (2016). Fast gene set enrichment analysis. Preprint at bioRxiv. https://doi.org/10.1101/060012.

65. Harrell J., F.E., and Dupont, C. (2020). Hmisc: harrell miscellaneous. R package version.

66. Pedersen, T.L. (2024). Ggraph: An Implementation of Grammar of Graphics for Graphs and Networks (Comprehensive R Archive Network). (CRAN)).

67. Callahan, B.J., McMurdie, P.J., Rosen, M.J., Han, A.W., Johnson, A.J.A., and Holmes, S.P. (2016). DADA2: High-resolution sample inference from Illumina amplicon data. Nat. Methods *13*, 581–583. https://doi.org/10.1038/nmeth.3869.

68. Hyatt, D., Chen, G.-L., LoCascio, P.F., Land, M.L., Larimer, F.W., and Hauser, L.J. (2010). Prodigal: prokaryotic gene recognition and translation initiation site identification. BMC Bioinf. *11*, 119. https://doi.org/10.1186/1471-2105-11-119.

69. Eddy, S.R. (2011). Accelerated profile HMM searches. PLoS Comput. Biol. *7*, e1002195. https://doi.org/10.1371/journal.pcbi.1002195.

70. Pasolli, E., Schiffer, L., Manghi, P., Renson, A., Obenchain, V., Truong, D.T., Beghini, F., Malik, F., Ramos, M., Dowd, J.B., et al. (2017). Accessible, curated metagenomic data through ExperimentHub. Nat. Methods *14*, 1023–1024. https://doi.org/10.1038/nmeth.4468.

71. Schloss, P.D. (2024). Rarefaction is currently the best approach to control for uneven sequencing effort in amplicon sequence analyses. mSphere *9*, e00354-23. https://doi.org/10.1128/msphere.00354-23.

72. Martino, C., Morton, J.T., Marotz, C.A., Thompson, L.R., Tripathi, A., Knight, R., and Zengler, K. (2019). A Novel Sparse Compositional Technique Reveals Microbial Perturbations. mSystems *4*. https://doi.org/10.1128/mSystems.00016-19.

73. Borg, I., and Groenen, P.J. (2005). Modern Multidimensional Scaling: Theory and Applications (Springer).

74. Anderson, M.J. (2006). Distance-based tests for homogeneity of multivariate dispersions. Biometrics *62*, 245–253. https://doi.org/10.1111/j.1541-0420.2005.00440.x.

75. Fernandes, A.D., Reid, J.N., Macklaim, J.M., McMurrough, T.A., Edgell, D.R., and Gloor, G.B. (2014). Unifying the analysis of high-throughput sequencing datasets: characterizing RNA-seq, 16S rRNA gene sequencing and selective growth experiments by compositional data analysis. Microbiome *2*, 15. https://doi.org/10.1186/2049-2618-2-15.

76. Lin, H., and Peddada, S.D. (2024). Multigroup analysis of compositions of microbiomes with covariate adjustments and repeated measures. Nat. Methods *21*, 83–91. https://doi.org/10.1038/s41592-023-02092-7.

77. Mallick, H., Rahnavard, A., McIver, L.J., Ma, S., Zhang, Y., Nguyen, L.H., Tickle, T.L., Weingart, G., Ren, B., Schwager, E.H., et al. (2021). Multivariable association discovery in population-scale meta-omics studies. PLoS Comput. Biol. *17*, e1009442. https://doi.org/10.1371/journal.pcbi.1009442.

78. Kumbhari, A., Cheng, T.N.H., Ananthakrishnan, A.N., Kochar, B., Burke, K.E., Shannon, K., Lau, H., Xavier, R.J., and Smillie, C.S. (2024). Discovery of disease-adapted bacterial lineages in inflammatory bowel diseases. Cell Host Microbe *32*, 1147–1162.e12. https://doi.org/10.1016/j.chom.2024.05.022.

79. Wang, S., Zeng, S., Egan, M., Cherry, P., Strain, C., Morais, E., Boyaval, P., Ryan, C.A., Dempsey, E., Ross, R.P., and Stanton, C. (2021). Metagenomic analysis of mother-infant gut microbiome reveals global distinct and shared microbial signatures. Gut Microbes *13*, 1911571. https://doi.org/10.1080/19490976.2021.1911571.

80. Hothorn, T., Hornik, K., Wiel, M.A.v.d., and Zeileis, A. (2008). Implementing a Class of Permutation Tests: The coin Package. J. Stat. Softw. *28*, 1–23. https://doi.org/10.18637/jss.v028.i08.

81. Akiyama, S., Nishijima, S., Kojima, Y., Kimura, M., Ohsugi, M., Ueki, K., Mizokami, M., Hattori, M., Tsuchiya, K., Uemura, N., et al. (2024). Multibiome analysis identifies distinct gut microbial signatures and their crosstalk in ulcerative colitis and Crohn's disease. Nat. Commun. *15*, 10291. https://doi.org/10.1038/s41467-024-54797-8.

82. Paradis, E., Claude, J., and Strimmer, K. (2004). APE: Analyses of Phylogenetics and Evolution in R language. Bioinformatics *20*, 289–290. https://doi.org/10.1093/bioinformatics/btg412.

83. DiCiccio, T.J., and Efron, B. (1996). Bootstrap confidence intervals. Statist. Sci. *11*, 189. https://doi.org/10.1214/ss/1032280214.

84. Efron, B. (1987). Better Bootstrap Confidence Intervals. J. Am. Stat. Assoc. *82*, 171–185. https://doi.org/10.1080/01621459.1987.10478410.

85. Efron, B. (1992). Bootstrap methods: another look at the jackknife. In Breakthroughs in statistics: Methodology and distribution (Springer), pp. 569–593.

86. Buchfink, B., Xie, C., and Huson, D.H. (2015). Fast and sensitive protein alignment using DIAMOND. Nat. Methods *12*, 59–60. https://doi.org/10.1038/nmeth.3176.

87. Ridlon, J.M., Kang, D.J., and Hylemon, P.B. (2006). Bile salt biotransformations by human intestinal bacteria. J. Lipid Res. *47*, 241–259. https://doi.org/10.1194/jlr.R500013-JLR200.

88. Funabashi, M., Grove, T.L., Wang, M., Varma, Y., McFadden, M.E., Brown, L.C., Guo, C., Higginbottom, S., Almo, S.C., and Fischbach, M.A. (2020). A metabolic pathway for bile acid dehydroxylation by the gut microbiome. Nature *582*, 566–570. https://doi.org/10.1038/s41586-020-2396-4.

89. Begley, M., Gahan, C.G.M., and Hill, C. (2005). The interaction between bacteria and bile. FEMS Microbiol. Rev. *29*, 625–651. https://doi.org/10.1016/j.femsre.2004.09.003.

90. Almeida, A., Nayfach, S., Boland, M., Strozzi, F., Beracochea, M., Shi, Z.J., Pollard, K.S., Sakharova, E., Parks, D.H., Hugenholtz, P., et al. (2021). A unified catalog of 204,938 reference genomes from the human gut microbiome. Nat. Biotechnol. *39*, 105–114. https://doi.org/10.1038/s41587-020-0603-3.

91. Cantalapiedra, C.P., Hernández-Plaza, A., Letunic, I., Bork, P., and Huerta-Cepas, J. (2021). eggNOG-mapper v2: Functional Annotation, Orthology Assignments, and Domain Prediction at the Metagenomic Scale. Mol. Biol. Evol. *38*, 5825–5829. https://doi.org/10.1093/molbev/msab293.

92. Wirbel, J., Pyl, P.T., Kartal, E., Zych, K., Kashani, A., Milanese, A., Fleck, J.S., Voigt, A.Y., Palleja, A., Ponnudurai, R., et al. (2019). Meta-analysis of fecal metagenomes reveals global microbial signatures that are specific for colorectal cancer. Nat. Med. *25*, 679–689. https://doi.org/10.1038/s41591-019-0406-6.

STAR★METHODS

KEY RESOURCES TABLE

REAGENT or RESOURCE	SOURCE	IDENTIFIER
**Deposited data**		
Global Burden of Disease (GBD) 2012–2021	GBD 2021 Collaborators[46]	https://www.healthdata.org/gbd
16S compendium	Abdill et al.[32]	https://doi.org/10.5281/zenodo.10452633
HallAB 2017 cohort	Hall et al.[27]	NCBI BioProject: PRJNA385949
NielsenHB 2014 cohort	Nielsen et al.[6]	ENA: PRJEB1220
HMP IBDMDB 2019 cohort	Lloyd-Price et al.[5]	NCBI BioProject: PRJNA398089
VilaAV 2018 cohort	Vich Vila et al.[47]	EGA: EGAS00001001704, EGAD00001004194
IjazUZ 2017 cohort	Ijaz et al.[48]	ENA: PRJEB18780
LiJ 2014 cohort	Li et al.[49]	ENA: PRJEB5224
Dominant strain gut microbiome phylogenies	Andreu-Sánchez et al.[36]	https://doi.org/10.5281/zenodo.14651412
HMP IBDMDB 2019 metabolomics (bile acids)	Lloyd-Price et al.[5]; Muller et al.[50]	https://github.com/borenstein-lab/microbiome-metabolome-curated-data
MetaPhlAn4 genomic database	Blanco-Miguez et al.[21]	http://cmprod1.cibio.unitn.it/biobakery4/metaphlan_databases/
Pfam-A HMM database	Mistry et al.[51]	https://www.ebi.ac.uk/interpro/entry/pfam/
MetaCyc pathway database	Caspi et al.[52]	https://metacyc.org
Source code of this study	This paper	https://doi.org/10.5281/zenodo.20390342; https://github.com/zhai199887/Inflammatory-Bowel-Disease
**Software and algorithms**		
vegan R package, version 2.7.2	Oksanen[53]	https://github.com/vegandevs/vegan
rstatix R package, version 0.7.3	Kassambara[54]	https://github.com/kassambara/rstatix
lme4 R package, version 1.1.37	Bates et al.[55]	https://github.com/lme4/lme4
lmerTest R package, version 3.1.3	Kuznetsova et al.[56]	https://github.com/runehaubo/lmerTestR
bigmds R package, version 3.0.0	Delicado and Pachón-García[57]	https://github.com/pachoning/bigmds
clusterSim R package, version 0.51.5	Dudek and Walesiak[58]	https://doi.org/10.32614/CRAN.package.clusterSim
coin R package, version 1.4.3	Hothorn et al.[59]	https://CRAN.R-project.org/package=coin
randomForest R package, version 4.7.1.2	Liaw and Wiener[60]	https://CRAN.R-project.org/package=randomForest
caret R package, version 7.0.1	Kuhn[61]	https://topepo.github.io/caret/
pROC R package, version 1.19.0.1	Robin et al.[62]	https://github.com/xrobin/pROC
ape R package, version 5.8.1	Paradis and Schliep[63]	https://CRAN.R-project.org/package=ape
fgsea R package, version 1.34.2	Korotkevich et al.[64]	https://github.com/ctlab/fgsea
Hmisc R package, version 5.2.4	Harrell Jr and Dupont[65]	https://github.com/harrelfe/Hmisc
ggraph R package, version 2.2.2	Pedersen[66]	https://github.com/thomasp85/ggraph
DADA2, version 1.14.0	Callahan et al.[67]	https://benjjneb.github.io/dada2/index.html
MetaPhlAn 4	Blanco-Miguez et al.[21]	https://huttenhower.sph.harvard.edu/metaphlan
Prodigal, version 2.6.3	Hyatt et al.[68]	https://github.com/hyattpd/Prodigal
HMMER, version 3.3	Eddy[69]	http://hmmer.org
Traitar3	Weimann et al.[37]	https://github.com/nick-youngblut/traitar3
anpan R package	Ghazi et al.[38]	https://github.com/biobakery/anpan
curatedMetagenomicData	Pasolli et al.[70]	https://bioconductor.org/packages/curatedMetagenomicData

EXPERIMENTAL MODEL AND STUDY PARTICIPANT DETAILS

16S rRNA amplicon compendium

A 16S rRNA amplicon compendium of 168,464 samples from 482 BioProjects (Table S37) was used for the country- and stage-level analyses in Figures 1, 2, and 3 (α-diversity, β-diversity, phylum-level composition and discovery, genus-level differential abundance) and for the cross-country external validation of the 19-genera classifier (MIRS, LASSO regression, and logistic-regression classification) across 70 countries in Figures 6E–6I.

Shotgun metagenomic cohorts

Six metagenomic cohorts obtained from curatedMetagenomicData (HallAB 2017, NCBI BioProject: PRJNA385949; NielsenHB 2014, ENA: PRJEB1220; HMP IBDMDB 2019, NCBI BioProject: PRJNA398089; VilaAV 2018, EGA: EGAS00001001704; IjazUZ 2017, ENA: PRJEB18780; LiJ 2014, ENA: PRJEB5224; 2,861 samples; 2,065 IBD +796 healthy controls; Table S41) were used for the genus- and species-level case-control differential abundance analyses in Figure 4, the MetaCyc functional analysis in Figure 5, and the training and internal evaluation of the 19-genera random-forest classifier (n = 2,004 training; n = 857 internal validation) in Figures 6A–6D.

MetaPhlAn4 SGB resource

The MetaPhlAn4 SGB resource (32,152 metagenomes from 24,829 individuals; IBD n = 1,779; 1,659 SGBs) was used for the strain-level phylogenetic analysis in Figure 7.

Human gut microbiome 16S rRNA amplicon sequencing data were retrieved from NCBI (245,627 samples from 1,437 BioProjects), and shotgun metagenomic data were accessed via curatedMetagenomicData. All primary data collection and ethical approvals were conducted in the original studies.

METHOD DETAILS

IBD three-stage classification

Based on previous work, we adopted published critical ranges of IBD incidence (CR-I) and prevalence (CR-P) (CR classification).[19] Stage 1 was defined as CR-I = 0.1–1.2 and CR-P = 1.2–10.5; Stage 2 as CR-I = 3.3–10.6 and CR-P = 31.2–100.5; and Stage 3 as CR-I = 18.1–34.1 and CR-P = 362.9–660.1. Our >160,000 microbiome samples were derived from datasets spanning 2012–2021. To obtain stage assignments that are consistent with these samples, we extracted data for 70 countries from the Global Burden of Disease (GBD) dataset (https://vizhub.healthdata.org/gbd-results/) for 2012–2021 and calculated the mean IBD incidence and prevalence for each country over 2012–2021. GBD was used as a single global epidemiological source to avoid the heterogeneity in statistical methods, diagnostic definitions, and cohort representativeness inherent to multi-source primary studies; the 2012–2021 calendar window was chosen to align with the years over which the 16S compendium samples were deposited at NCBI; and the per-country arithmetic mean was applied to the CR thresholds directly, without an additional machine-learning classifier, keeping the classification step transparent and reproducible. Applying the above thresholds, each of the 70 countries was assigned to one of three IBD stages: 25 countries were categorized as Stage 1, 29 as Stage 2, and 16 as Stage 3 (Table S36). The resulting distribution across stages was broadly concordant with prior reports. For a quantitative comparison, we mapped our 70-country stage assignments to the per-country stage assignments reported by Hracs et al. for the 2010-2019 decade (Table S59): 30 countries are concordantly classified, 7 are discordantly classified (CN, CO, FR, IE, IL, IT, ES), and the remaining 33 have no population-based observation within 2010–2019 in the GIVES-21 records that underlie Hracs et al.'s classifier.

Sample selection and preprocessing

Following the search strategy described in a 2025 Cell study,[32] we retrieved metadata for all biological samples from the NCBI "human gut metagenome" category. We retained samples whose library source was annotated as "genomic" or "metagenomic" and whose library strategy was annotated as "amplicon", yielding 245,627 samples from 1,437 BioProjects. We then excluded BioProjects with fewer than 50 samples (10,752 samples in total), resulting in a working set of 234,875 samples from 811 BioProjects. After amplicon processing, 31,887 samples (13.6%) were identified as containing non-applicable data—such as BioProjects targeting fungi or archaea, or BioProjects incorrectly annotated as using shotgun or nanopore sequencing rather than 16S amplicon sequencing—and were removed. An additional 31,509 samples (13.4%) were excluded because, although valid results might be obtainable, they would require manual intervention and detailed knowledge of study-specific sequencing strategies. Specifically, we removed BioProjects in which at least 5 of the first 10 processed samples contained >25% chimeric reads. Several additional BioProjects were excluded because they contained samples associated with multiple sequencing runs or because associated samples could not be downloaded. This is a conservative exclusion: aggregating multiple SRA runs per biological sample without explicit per-run metadata would either double-count reads from a single specimen or introduce within-sample technical-replicate variance that the BioProject random effect cannot absorb, both of which would bias all downstream linear mixed-effects estimates. At the sample level, we removed 807 samples whose reads were completely discarded during quality filtering. For the remaining BioProjects, we aggregated BioProject-level taxonomic count tables into a single large matrix with 168,464 samples (from 482 BioProjects) as rows and 4,018 unique taxonomic identifiers as columns, forming a global human gut microbiome

compendium. We then applied additional quality-control steps. First, we removed 16,781 samples with fewer than 10,000 sequencing reads (10% of all samples). Next, we removed 2,485 taxonomic entries whose total read count across all remaining samples was <1,000, as well as 681 taxa detected in fewer than 100 samples. After removing these taxa, we re-evaluated read counts and discarded an additional 21 samples whose total read counts remained <10,000. We then assessed, for each sample, the proportion of reads that could not be classified to at least the phylum level. We removed 30,061 samples in which >10% of reads remained unclassified at the phylum level. The final filtered compendium comprised 121,601 samples and 1,514 taxa. This filtered dataset was used for almost all downstream analyses. Detailed sample metadata are provided in Table S37. Per-step sample retention by stage and country, the corresponding stratified bias analyses, and the BioProject-level retention summary for the 482 BioProjects of the final compendium are provided in Figure S33 and Tables S49, S50 and S51.

Calculation of Shannon diversity

To quantify within-sample microbial diversity, we computed three α-diversity metrics: Shannon diversity, Simpson diversity, and species richness. Specifically, the Shannon index was calculated using the diversity function in the R package vegan (v2.7.2), with natural logarithms (base e) and including all taxonomic features in the filtered dataset. Prior to calculation, we aggregated amplicon sequence variants (ASVs) only when their taxonomic annotations were identical across all ranks, ensuring that counts were strictly aggregated at the level of complete taxonomic annotations rather than at any single rank (e.g., phylum, genus). Similarly, the Simpson index and species richness were computed using the diversity and specnumber functions in vegan (v2.7.2), respectively, on the same filtered and annotation-consistent feature table.

Rarefaction analysis and phylum discovery rate

To evaluate the relationship between sample size and the number of detected phyla across countries, we performed a sample-size-based rarefaction analysis. Using the unfiltered working set (168,464 × 4,018), we first aggregated ASVs at the phylum level, again retaining only features with identical taxonomic annotations. Rarefaction was then performed in parallel at the country level (ISO code). For each country, we constructed simulated datasets of varying sizes by randomly subsampling n samples without replacement, with n ranging from 1 to 150,000. For each sample size, we performed 50 independent subsampling iterations and recorded the number of unique phyla observed in each iteration. The mean number of observed phyla across iterations was reported as the final estimate for that sample size. To account for differences in sequencing depth, we also calculated the mean number of newly detected phyla per million reads (“phyla per million reads”) and used this as the phylum discovery rate. Results were visualized as discovery curves with sample size on the x axis and mean observed phylum count on the y axis (Figure 2A). An inset showed the trend in phylum discovery rate (phyla per million reads) as a function of sample size. This analysis indicated that the rate of new phylum discovery decreases as sample size increases, suggesting that the current global sampling effort captures most readily detectable microbial diversity, while under-sampled countries still harbor incompletely characterized microbial communities.

Between-country and between-stage alpha-diversity comparison

Prior to comparative analyses, we removed samples lacking valid ISO country codes and retained only samples from the 70 countries included in the main analysis panel (total n = 117,799). For these samples, α-diversity was quantified using the Shannon, Simpson, and richness indices computed as described above. Pairwise comparisons of α-diversity between countries and between IBD stages were performed using the pairwise_wilcox_test function from the R package rstatix (v0.7.3), implementing the Wilcoxon rank-sum test. Multiple testing correction was applied using the Benjamini-Hochberg false discovery rate (FDR) procedure (p.adjust.method = “fdr”), and FDR-adjusted q values were reported.

Rarefaction-based alpha-diversity across countries and IBD stages

To further assess differences in α-diversity across countries and across the three IBD stages, we performed a depth-standardized rarefaction analysis. For each of the 70 countries and each IBD stage, we randomly selected 1,000 samples (restricted to groups with ≥1,000 samples to allow sampling without replacement) from the filtered dataset. Each selected sample was rarefied to 10,000 sequencing reads by random subsampling of reads. Using these rarefied samples, we computed the Shannon index for each sample and then calculated the mean Shannon diversity for each country and each IBD stage. The entire rarefaction and averaging procedure was repeated 1,000 times to obtain stable estimates and uncertainty intervals.[71]

Principal coordinates analysis

We performed principal coordinates analysis (PCoA) on a robust centered log-ratio (rCLR)-transformed abundance matrix. For each sample, the count of each taxon was divided by the geometric mean of all taxa in that sample, and the natural logarithm of this ratio was taken to mitigate compositional effects and normalize for differences in sequencing depth.[72] Using the rCLR-transformed data, we computed pairwise Aitchison distances between all samples to obtain a distance matrix. To perform dimensionality reduction on this large sample set, we applied the divide-and-conquer multidimensional scaling (MDS) algorithm proposed by Delicado and Pachón-García, as implemented in the R package bigmds (v3.0.0) via the divide_conquer_mds function (parameters: block size l = 10,000; number of overlapping points c_points = 16; target dimensionality r = 8).[57,73] This algorithm partitions the sample matrix into overlapping blocks and stitches the results at shared points, efficiently approximating the global MDS structure and enabling

CellPress
OPEN ACCESS

analysis of large-scale datasets. We extracted the first eight principal coordinates (mds1-mds8) and used their coordinate matrix to visualize the spatial distribution of samples (e.g., heatmaps) and to generate kernel density plots of samples from different IBD stages in the MDS space.

Cluster evaluation of the three IBD stages

To evaluate the clustering quality of the three IBD stages in the MDS space, we computed the Davies-Bouldin index (DBI), which quantifies the ratio of between-cluster separation to within-cluster compactness. Using the eight-dimensional MDS embedding (mds1-mds8) derived from the rCLR-based Aitchison distance, we applied the index. DB function from the R package clusterSim (v0.51.5), treating the three IBD stages as cluster labels (centrotypes = "centroids"). To assess whether the observed clustering was better than expected by chance, we performed a bootstrap permutation test. We generated 250,000 random labelings of samples by permuting the original stage labels without replacement and computed the DBI for each permuted labeling, thereby obtaining an empirical null distribution of DBI values. The observed DBI for the true IBD stage labels (13.26) was markedly lower than the range of DBI values under random labeling, corresponding to an empirical p value $<4 \times 10^{-6}$. These results indicate that samples from the three IBD stages form clusters in the eight-dimensional MDS space that are significantly more compact and better separated than expected by chance.

PERMDISP analysis of stage dispersion

We applied PERMDISP2[74] to the same eight-dimensional MDS embedding used for the Davies-Bouldin analysis above using the betadisper function from the R package vegan (v2.7.3) with the default type = "median" (spatial median). We drew a stratified subsample of 2,000 samples per stage. The overall F test was obtained from the anova function on the fitted betadisper object and from the permutest function (999 permutations) in the same package, and pairwise contrasts were obtained from the Tukey HSD function at the default 95% family-wise confidence level. Per-stage means, the F test, and pairwise Tukey HSD contrasts are reported in Table S52, with a boxplot in Figure S35.

Diversity and clustering analyses on an IBD-only subset

To assess whether the patterns observed across the three stages were driven by the inclusion of non-IBD disease samples, we repeated the alpha-diversity, PCoA, PERMANOVA, and Davies-Bouldin analyses on an IBD-only subset (Table S37 IBD_label = "IBD"; $n = 4{,}171$; Stage 1 $n = 286$, Stage 2 $n = 344$, Stage 3 $n = 3{,}541$). The depth-standardized rarefaction protocol described above was applied to mean Shannon, Simpson, and species richness per stage across 1,000 iterations, with pairwise stage differences assessed by the Wilcoxon rank-sum test and Benjamini-Hochberg FDR correction. PCoA was performed on the same robust Aitchison distance as the full cohort, with the first four principal coordinates extracted by cmdscale. The contribution of stage to this PCoA space was tested by PERMANOVA using the adonis2 function from the R package vegan (v2.7.2), with the formula d $\sim$ stage + sex + age_group and 199 permutations; SDI was omitted because Stage 1 contained samples from a single country. The same Davies-Bouldin permutation framework described above was applied to the IBD-only PCoA, with 50,000 stage-label permutations.

Differential abundance analysis

For differential abundance analysis across countries and IBD stages in the global dataset, we further filtered the taxa and samples. We retained only taxa with a mean relative abundance $\geq$0.01% and a prevalence (proportion of non-zero samples) $\geq$5% across the overall sample pool; within those selected taxa we further restricted analyses to samples with $\geq$1,000 sequencing reads, yielding 117,671 samples and 203 taxa for modeling. To test for differences in abundance between countries or between IBD stages, while simultaneously assessing robustness of the stage main-effect findings against V-region, platform, and pipeline heterogeneity inherent to the 16S compendium, we adopted a five-method $\times$ three-pairwise cross-method consensus framework with national-level industrialisation (SDI) covariate adjustment. The five methods rest on different statistical foundations: linear mixed-effects models (LMM, the main analysis); ALDEx2[75]; ANCOM-BC2[76]; MaAsLin2[77]—these four sample-level methods constituting the cross-method panel—and LEfSe, retained as the primary baseline (without covariates by design). All four sample-level cross-method analyses (LMM, ALDEx2, ANCOM-BC2, MaAsLin2) shared the same fixed-effect covariate set (sex_factor + age_group + disease + sdi) and used Benjamini-Hochberg FDR <0.05 as the significance threshold; Project, amplicon, and mean ASV length entered as random intercepts in LMM (fitted with the R packages lme4 and lmerTest[55,56]), ANCOM-BC2, and MaAsLin2 to account for potential technical heterogeneity,[32] and as fixed effects in ALDEx2 (which does not natively support random effects). The IHME GBD 2012–2021 mean Socio-Demographic Index (SDI) was added as a national-level industrialisation fixed-effect covariate to disentangle the country-level industrialisation gradient from the IBD-stage gradient. Individual-level lifestyle metadata (BMI, diet, medication history, antibiotic exposure) are inconsistently reported across the public 16S cohorts and were therefore not modeled as direct covariates in the present analysis. For each taxon $\times$ stage-pair cell, we computed the four-method directional concordance (sign agreement with the LMM main analysis) and the four-method significance overlap (BH-FDR <0.05 in all four methods).

$$\text{taxon abundance} \sim \text{stage} + \text{sex} + \text{age}_{\text{group}} + \text{disease} + \text{sdi} + (1|\text{project}\backslash) + (1|\text{amplicon}) + (1|\text{length})$$

$$\text{taxon abundance} \sim \text{country} + \text{sex} + \text{age}_{\text{group}} + \text{diease} + (1|\text{project}) + (1|\text{amplican}) + (1|\text{length})$$

$$c_{ij} = \beta_{0,i} + \beta_{1,i}\, stage_j + \beta_{2,i}\, sex_j + \beta_{3,i}\, age_{group_j} + \beta_{4,i}\, disease_j +$$

$$\beta_{5,i} sdi_j + \beta_{6,i}\, project_j + \beta_{\Gamma,i}\, amplicon_j + \beta_{8,i}\, length_j + \varepsilon_{i,j}$$

$$y_{ij} = d_j + \beta_{0,j} + \beta_{1,i}\, stage_j + \beta_{2,i}\, sex_j + \beta_{3,i}\, age_{group_j} + \beta_{4,i}\, diease_j +$$

$$\beta_{5,i}\, sdi_j + u_i^{(project)} j + u_i^{(amplicon)} j + u_i^{(length)} j + \varepsilon_{ij}$$

$$z_{ij} = \beta_{0,i} + \beta_{1,i} \cdot stage_j + \beta_{2,i} \cdot sex_j + \beta_{3,i} \cdot age_{group_j} + \beta_{4,i} \cdot disease_j + \beta_{5,i} \cdot sdj_j + u_i^{(project)} j + u_i^{(amplicon)} j + u_i^{(length)} j + \varepsilon_{ij}$$

These model formulations followed previously described approaches. For each model, we iteratively changed the reference level of the categorical predictor (country or stage) so that all pairwise contrasts between regions or stages could be obtained. To account for the compositional nature of microbiome data, a pseudocount of 1 was added to zero values in the relative abundance matrix prior to centered log-ratio (CLR) transformation. Taxa-level abundances were then transformed using the CLR transformation. The resulting CLR values were standardized column-wise to mean 0 and standard deviation 1 before fitting the mixed-effects models. All *p*-values from the mixed-effects models were adjusted for multiple testing using the Benjamini-Hochberg procedure to obtain FDR-controlled q-values. *p* values smaller than 2.2×10^{-16} were truncated and reported as 2.2×10^{-16}. Complete model results for these analyses are provided in Tables S13 and S14. In contrast, ALDEx2 used Monte Carlo sampling from the Dirichlet distribution with each instance center log-ratio transformed; ANCOM-BC2 used sampling-fraction bias correction with s0_perc = 0.05; MaAsLin2 used a linear model with TSS normalization and log transformation as its default; the resulting four-method significance counts per stage-pair are visualised in Figure 3B. Cross-method concordance summaries (5-method × 3-pairwise; 609 records) are provided in Table S43, per-method records for ALDEx2, ANCOM-BC2, MaAsLin2 and LEfSe (2,098 records) in Table S44, and the per-method sig+dir/dir-only bars together with the four-method significance UpSet are visualised in Figure S26. We applied an analogous pipeline to the IBD case-control dataset, resulting in 2,861 samples and 42 species for species-level analyses and 33 genera for genuslevel analyses. For these models, the formulas were.[78]

$$\text{taxon abundace} \sim \text{disease}_{\text{subtype}} + \text{age} + \text{country} + (1|\text{project}) + (1|\text{median\_read\_length})$$

$$\text{taxon abundance} \sim \text{disease} + \text{age} + \text{country} + (1|\text{project}) + (1|\text{median\_read\_length})$$

Again, following the previously described model structures. Model fitting, transformation, and multiple-testing correction were performed as above (Tables S18, S20 and S21).

To assess the influence of non-IBD disease samples on the stage signal, we additionally repeated the stage 1/2/3 LMM differential-abundance analysis on the IBD-only subset defined above (*n* = 4,171). The IBD-only LMM uses the same fixed-effect covariates as the full-cohort analysis except disease and the same project/amplicon/sequencing-length random intercepts. Direction concordance between the IBD-only and full-cohort LMMs across the 203 evaluable taxa × 3 stage pairs is reported in three significance tiers (taxa significant in both cohorts, taxa significant in either cohort, all 203 taxa irrespective of significance) in Table S46; the per-taxon IBD-only LMM emmeans output is provided as Table S45, the cross-cohort heatmap of the four-method consensus genera (*n* = 41) in Figure S31, and the cross-cohort UpSet of BH-FDR <0.05 records in Figure S32. To assess V-region primer-specific bias for specific taxa as a potential source of artifactual stage signal, we additionally repeated the stage 1/2/3 LMM differential-abundance analysis stratified by the three largest V-region subsets of ST37 (AMPLICON: "v4", *N* = 86,346; "v3-v4", *N* = 29,077; "v1-v2", *N* = 8,147; of which 71,218, 24,513 and 7,381 respectively entered the LMM after the same STAR Methods filter as the full cohort). Each subset LMM uses the same fixed-effect covariates as the full-cohort analysis except sdi (which is collinear with stage within each V-region subset and is therefore dropped) and the same project/sequencing-length random intercepts; v1-v2 stage 1 carried only 60 samples with sex/age_group/disease all single-valued within the cell and was likewise dropped. Per-taxon BH-FDR significance of the four-method consensus genera (*n* = 41, the same row set as Figure 3B) across the three V-region subsets and three stage pairs is reported in Figure S29, and the V-region-intersection UpSet of BH-FDR <0.05 records (stacked by stage pairwise contrast) in Figure S30; the per-taxon three-subset LMM emmeans output (203 evaluable taxa × 3 stage pairs) is provided as Table S53, and the cross-subset direction concordance, Top-K Jaccard, Spearman ρ, robust and direction-flipped genera lists as Table 54.

COUNTRY-LEVEL PHYLUM DISCOVERY RATE

To generate the country-level phylum discovery curves shown in Figure 2A, we carried out the following analysis. For each country, we randomly selected n microbiome samples from that country (without replacement) and recorded the number of unique phyla present across the selected samples. This subsampling procedure was repeated 50 times for each sample size n, and the mean number of observed phyla was reported. The procedure was repeated across all specified sample sizes for every country. The resulting data are provided in Table S9. To test whether discovery curves differed significantly between countries, we performed pairwise Wilcoxon

rank-sum tests for every possible pair of countries at each sample size, comparing the number of observed phyla between countries. Results of these pairwise tests are summarized in Table S38. For Figure 2A, 95% confidence intervals were obtained by bootstrapping with 1,000 repetitions, and the corresponding intervals are shown in Figure S24.

IBD stage region signature determination

We used principal component analysis (PCA) to identify taxa most strongly associated with overall microbiome variation within each IBD stage region and to define a heuristic "variance score" for each taxon. This score ranges from 0 (no association) to 1 (perfect correspondence) and reflects how closely each taxon aligns with the dominant axes of variation in its region. For each region, taxonomic read counts were transformed using the rCLR approach.[72] PCA was then performed independently for each region, retaining a sufficient number of principal components (PCs) to explain at least 50% of the total variance. Using the eigenvectors of the selected PCs, we computed, for each taxon in each region, the proportion of its variance explained by these PCs; this proportion was defined as the variance score. Thus, each taxon received a variance score between 0 and 1. The key assumption is that if a subset of PCs captures most of the variance in the dataset, and a given taxon's variation is largely explained by the same PCs, then that taxon contributes substantially to the overall structure of microbiome variation in that region. Conversely, taxa whose variance is poorly captured by the selected PCs are considered more weakly associated with the regional microbiome structure (Tables S15, S16 and S17).

Aggregation to country-level taxonomic profiles

We assigned all samples with valid metadata to one of 70 countries, yielding a total of 117,799 samples. For each country, we computed the mean relative abundance of each of the 1,514 taxa, resulting in a 70 $\times$ 1,514 country-by-taxon abundance matrix. This matrix was used as the basis for multiple downstream microbiome analyses at the country level.

Public cohorts of patients with IBD and normal controls

For the six metagenomic cohorts HallAB 2017[27] (NCBI BioProject: PRJNA385949), NielsenHB 2014[6] (ENA: PRJEB1220), HMP 2019[5] IBDMDB (NCBI BioProject: PRJNA398089), VilaAV 2018[47] (EGA: EGAS00001001704, EGAD00001004194), IjazUZ 2017[48] (ENA: PRJEB18780), and LiJ 2014[49] (ENA: PRJEB5224), data were accessed via curatedMetagenomicData. These cohorts collectively cover a wide range of demographic and clinical characteristics, including disease status (healthy, CD, UC), age, country, project identifier, and read length. We retained only normal control (NC) and IBD samples and removed samples labeled as undetermined colitis. After filtering, we obtained 796 controls and 2,065 IBD samples; within the IBD group, 1,205 samples were classified as CD and 860 as UC. Detailed sample counts, taxonomic abundances, and metadata are provided in Tables S39, S40 and S41. After harmonization across cohorts, we retained 276 consistent taxa across a total of 2,861 samples for downstream integrative analyses.

Functional profiling and pathway-level differential analysis

Metagenomic functional profiles for the 6 cohorts described above were obtained from curatedMetagenomicData, using pathway-level annotations summarized at the MetaCyc pathway level. Functional abundance matrices were generated from HUMAnN outputs, in which the categories "UNMAPPED" and "UNINTEGRATED" represent special aggregate entries rather than biologically interpretable pathways; these entries were therefore removed prior to downstream analyses. Following their removal, pathway abundances were re-normalized within each sample to ensure that relative abundances summed to one. Functional categorization of pathways was performed based on previously published literature.[79] Specifically, pathway identifiers (pathway_id) were extracted from the MetaCyc pathway strings and matched to a curated mapping table linking pathway_id to higher-level metabolic functions. To ensure a one-to-one correspondence, only the first occurrence of each pathway_id was retained. Pathways that could not be assigned to any functional category were initially grouped under "Others". To further reduce the proportion of unclassified pathways, a second round of manual curation was performed, in which remaining pathways were reassigned based on biochemical annotations and pathway descriptions (Table S22). To derive metabolic function-level abundances, relative abundances of all pathways belonging to the same functional category were summed within each sample, yielding the total relative abundance of each metabolic function per sample. Differential abundance analyses were conducted using blocked (stratified) Wilcoxon rank-sum tests, with project included as a blocking factor to control for cohort-specific effects. In this framework, comparisons were performed within each project and then combined across projects to obtain a global test statistic and *p* value. Only projects containing samples from both comparison groups were included in the statistical testing. All blocked Wilcoxon tests were implemented using the coin R package[80] (version 1.4.3), and multiple testing correction was applied using the BH procedure to control the FDR (Table S23). At the pathway level, blocked Wilcoxon tests were performed separately for CD vs. NC and UC vs. NC, yielding effect directions and FDR-adjusted *p* values for each pathway (Table S24). For metabolic function–level analyses, functions were retained if they satisfied all of the following criteria: FDR <0.05, overall mean relative abundance >0.01, and prevalence $\geq$5%. Functions not meeting these criteria were collapsed into the "Others" category. For pathway-level visualization, pathways were retained if at least one of the two comparisons (CD vs. NC or UC vs. NC) reached statistical significance (FDR <0.05), and if the mean relative abundance in the disease group exceeded 0.005 with a prevalence $\geq$5%. These filtered pathways were used for subsequent visualization and interpretation.

Strain-strain interaction network

To define a set of microbial taxa exhibiting consistent directional changes in IBD relative to healthy controls, we parsed the linear mixed-effects model output table (Table S21) for all pairwise contrasts involving UC/CD versus NC and harmonized effect directions to a unified disease - NC scale. For each taxon, those showing at least one significant positive disease - NC effect (Benjamini-Hochberg-adjusted FDR <0.05) in any UC or CD contrast and no significant negative effects were classified as IBD-associated, whereas taxa showing at least one significant negative effect and no significant positive effects were classified as Health-associated; taxa displaying both significant positive and negative effects ("Mixed") or no significant effects were excluded from network inference. This procedure yielded a final set of 30 taxa that were carried forward as network nodes. To characterize microbial crosstalk under a healthy baseline, interaction inference was restricted to NC samples only. Relative abundance profiles of the 30 selected taxa were extracted from NC samples to form a sample-by-taxon matrix. Pairwise associations across NC samples were assessed using Spearman and Pearson correlation coefficients, calculated with the rcorr function from the Hmisc package (version 5.2.4), and *p* values from each method were independently adjusted for multiple testing using the Benjamini-Hochberg procedure. Taxon pairs were retained as network edges only if they satisfied a stringent robustness criterion: BH-FDR <0.05 for both Spearman and Pearson correlations, absolute correlation coefficients ≥ 0.20[78,81] for both methods, and concordant correlation direction. For each retained edge, an aggregate association strength was defined as the mean of Spearman and Pearson coefficients (cor mean = $(\rho + r)/2$), and edges were labeled as positive (cor mean >0) or negative accordingly; all retained interactions are reported in Table S25. Network visualization was performed using ggraph (version 2.2.2), with nodes colored by genus and sized proportionally to degree, edges colored by correlation sign (black for positive, gray for negative) and scaled in width according to |cor mean|. For visualization purposes only, force-directed layouts were computed separately within Health-associated and IBD-associated taxa and vertically offset to enhance readability.

Random forest model for the MIRS

We employed a random forest model (implemented in the randomForest R package, v4.7.1.2) to identify microbiome-derived features capable of distinguishing IBD from healthy samples at baseline. To ensure sample independence within the random forest model, we used the createDataPartition function from the caret package (v7.0.1) to randomly divide 2,861 samples into a training dataset (n = 2,004; 1,446 IBD samples and 558 healthy controls) for model training and a validation dataset (n = 857; 619 IBD samples and 238 healthy controls) for model testing. We initially trained two random forest models: one included all 110 genera shared between the training and validation datasets as input features, while the other included 21 genera. Based on the model incorporating the 21 genera, we performed 10-fold cross-validation and ranked the features by mean decrease in accuracy, thereby identifying the top 19 most important genera (Figure S25) and establishing a more concise model. To validate the robustness of this refined 19-genera model, we compared its performance against ten simulated models, each constructed with a random subset of 19 genera drawn from the total pool of 110 genera. We performed leave-one-country-out and leave-one-BioProject-out validations on the training dataset, each holding out one country or one BioProject and retraining the 19-genera model with the same parameters. Additionally, we trained two supplementary random forest models to evaluate whether the predictive value of the microbial features could be attributed to age and country. One model included only age and country, while the other incorporated these intrinsic factors along with the 19 genera. The predictive performance of all random forest models was evaluated by the AUC, and model performance differences were assessed using the DeLong test. Using the random forest model constructed with the 19 key genera, we developed the Microbial Inflammatory Risk Score (MIRS), representing the probability that a participant is classified as "IBD" by the model. The probability (MIRS) was estimated through tree-voting aggregation, calculated as the proportion of trees voting for the "IBD" class:

where X denotes the feature matrix of the 19 genera for a given participant, T represents the total number of trees in the random forest (set to 1,000 in this study), and $h_t(X, IBD)$ indicates the prediction of the tth tree (t = 1, 2, 3, …, T) regarding whether the sample belongs to the IBD class. Subsequently, we applied the trained random forest model to a dataset comprising 117,799 samples from 70 countries to compute the mean relative abundances of the 19 core genera for each country (70×19 matrix; Table S42). Using these country-level microbial profiles, MIRS scores were calculated for each country, and their Pearson correlations with national IBD incidence and prevalence were analyzed (Table 26).

LASSO-based feature selection and multiple linear regression modeling

To investigate whether gut microbial genera could predict the incidence and prevalence of IBD across 70 countries, we applied a LASSO (least absolute shrinkage and selection operator) regression model using genus-level relative abundance profiles as predictors and log-transformed IBD incidence or prevalence as continuous response variables. The optimal penalty parameter (λ) was determined by 10-fold cross-validation to avoid overfitting. Genera with non-zero coefficients in the LASSO model were considered as key microbial predictors. Subsequently, the selected genera were included in a multiple linear regression (MLR) model to evaluate their collective predictive performance for IBD incidence and prevalence. Model performance was assessed by Pearson correlation between predicted and observed values of log-transformed disease metrics. The final genera subsets showed significant correlations with observed IBD incidence (r = 0.45, $p < 0.001$) and prevalence (r = 0.54, $p < 0.001$). In addition, to further validate the classification performance of the model, we dichotomized the log-transformed IBD incidence and prevalence into "high" and "low" groups according to their median values, corresponding to "IBD" and "NC" states, respectively. Based on the LASSO-selected genera, logistic regression models were constructed to estimate the predicted probability for each sample, followed by the

generation of receiver operating characteristic (ROC) curves using the pROC package (v1.19.0.1). The predictive performance of both single-genera and multi-genera combined models was evaluated by the area under the AUC.

IBD stage association with microbial phylogeny

Phylogenetic distances were extracted from the phylogenies using ape v5.8.1 via cophenetic.phylo.[82] For disease staging, samples belonging to the same IBD stage were assigned a distance of 0, whereas pairs of samples from different stages were assigned stage distances proportional to their separation (e.g., stage 3 versus stage 1 was encoded as 2). We then computed the correlation between phylogenetic distances and these stage distances using a Mantel test (vegan, v2.7.2) with both Pearson and Spearman coefficients and 2,000 permutations, and subsequently estimated BH-FDRs. A comparison of correlation estimates obtained from the two coefficients showed high concordance. Given this robustness, we proceeded with the Pearson-based estimates for downstream analyses.

Microbial characteristics and IBD stage effect

To generate standardized phenotype profiles for all 1659 SGBs, we retrieved their nucleotide FASTA sequences (.fna) from the MetaPhlAn4 genomic database and performed *de novo* gene prediction prior to phenotype inference. Because SGBs are constructed from metagenome-assembled genomes and draft isolates, their assemblies are typically fragmented and composed of contigs of heterogeneous length; thus, direct use of precomputed protein files would introduce unpredictable biases arising from inconsistent gene-calling pipelines. To avoid this issue and to ensure uniform ORF detection across all SGBs, we applied Prodigal (v2.6.3) in metagenomic mode (-p meta), which does not require the $\geq$20 kb training region needed for the single-genome model and is specifically optimized for fragmented metagenomic assemblies by identifying ORFs independently on each contig without assuming chromosomal continuity. For every SGB, Prodigal (meta) generated both predicted amino acid sequences (.faa) and gene coordinate files (.gff), which were subsequently annotated against the Pfam-A HMM database using hmmsearch (HMMER v3.3). Domain-level annotations were summarized into sample-specific Pfam matrices and provided the feature space for microbial phenotype inference. Phenotype prediction was then performed using Traitar3[37] in "from_genes" mode, applying both the phyletic pattern classifier (phypat) and the phylogeny-aware classifier (phypat+PGL). Only phenotype assignments that were consistently predicted by both classifiers were retained to ensure high-confidence trait calls. To generate a binary trait matrix (1 = presence; 0 = absence) for each SGB, we converted the high-confidence phenotype assignments into a standardized per-trait presence/absence profile, which served as the input for downstream association analyses. Annotations were then associated with IBD stage effects (Rho) using a linear model, and BH-adjusted FDRs were estimated to control for multiple testing. For traits with FDR <0.05, we further incorporated the genus identity of each SGB—specifically, whether it belonged to the unclassified Lachnospiraceae group—as an additional covariate in a subsequent model. This adjustment was necessary because the unclassified Lachnospiraceae group was markedly enriched among SGBs exhibiting strong IBD stage effects and also showed increased abundance in UC patients, suggesting that this family could simultaneously influence both disease state and strain-level stratification. Including this taxonomic background as a covariate, therefore, allowed us to isolate the independent contribution of each microbial phenotype while minimizing confounding from lineage-specific ecological or evolutionary structure.

Phylogenetic generalized mixed models in anpan

We conducted analyses separately within each stage and matched the binary IBD phenotype (case/control) to the leaves (samples) of each reconstructed species phylogeny. When longitudinal sampling or repeated measurements were available for a given participant, we randomly retained a single sample per individual to ensure independence at the subject level. We then fitted phylogenetic generalized linear mixed models (PGLMMs) using anpan[38] to assess whether IBD exhibited a detectable phylogenetic signal on each phylogeny. Because IBD is a binary outcome, we used a binomial model with IBD as the dependent variable, including age and country as covariates, and a phylogenetic random effect. The covariance structure of the phylogenetic random effect was specified by the sample-to-sample phylogenetic relationship matrix derived from the tree under a Brownian motion assumption, with variance parameter denoted as σ_phylo. Model fitting was performed in a Bayesian framework. To test whether the phylogenetic random effect improved the explanation and prediction of IBD, we compared two models: (i) a phylogenetic model including the phylogenetic random effect; and (ii) a corresponding null model that removed the phylogenetic random effect while retaining the same covariates. Predictive performance was evaluated using Pareto-smoothed importance sampling leave-one-out cross-validation (PSIS-LOO) to obtain expected log predictive density (ELPD). We defined elpd_diff = ELPD_null-ELPD_phylo; thus, smaller (more negative) elpd_diff values indicate improved prediction when including the phylogenetic random effect. Uncertainty in elpd_diff was summarized as elpd_diff ± 2SE. Evidence for a phylogenetic effect was defined as a confidence interval excluding 0 and elpd_diff < -4, indicating that the phylogenetic model predicted substantially better than the null. To ensure the reliability of the loo approximation, we excluded results where the diagnostic Pareto K value was $\geq$0.7 in at least 1% of samples. In such cases, the loo may be driven by a small number of influential observations, and we additionally used the posterior distribution of σ_phylo as a complementary metric to assess whether the phylogenetic effect differed from 0.

Enrichment of taxonomic levels

To test whether IBD stage-associated effects were enriched in specific taxonomic levels, we performed gene set enrichment analysis (GSEA) using the R package fgsea[64] (v1.34.2). Each SGB was assigned to all taxonomic levels to which it belonged; because taxonomic levels are nested, the same SGB could contribute to multiple categories. We ran fgsea with standard parameters, using the ρ values as the ranking statistic. Overall, *p*-values were adjusted once across all tested taxonomic categories using the BH-FDR procedure (Table 31).

Bile-acid association and robustness analyses in the SGB4993 phylogeny

To assess whether the IBD-enriched subclade was associated with specific bile acids, we analyzed $n = 40$ HMP2 IBDMDB samples falling within the Eisenbergiella sp900066775 phylogeny (9 in the subclade, 31 outside). For each of the nine measured bile acids, the subclade effect (Group) was tested with a linear mixed-effects model fitted using the R packages lme4 and lmerTest (v1.1.37 and v3.1.3, respectively) at REML = FALSE.

$$\text{bile acide} \sim \text{Group} + \text{age} \ (1|\text{study})$$

BH-adjusted FDRs were computed across the nine bile acids. Two robustness checks were performed alongside the main model: (i) a non-parametric bootstrap with 10,000 resamples using the R package boot, from which percentile and BCa 95% confidence intervals[83–85] were derived; and (ii) a leave-one-out sensitivity analysis that iteratively removed one sample, refitted the model, and recorded the per-iteration regression coefficient, raw P, and BH-FDR. Table S35 provides the full per-iteration outputs; Figure S37 shows the forest plot of the four estimate views.

Functional validation of bile-acid metabolism in SGB4993

The SGB4993 representative genome (GCA_003461565.1) was queried with DIAMOND BLASTp (v2.1.24)[86] against 19 *Clostridium scindens* bai-operon proteins[87,88] and 7 BSH proteins[89] from SwissProt at an e-value of 1×10^{-10}, with orthogonal UHGG eggNOG-mapper annotation[90,91] for KEGG K01442 and K15868-K15874. The bai-operon analytical module[92] was then applied to HUMAnN3-stratified Uniref. 90 abundances on the $n = 40$ HMP IBDMDB samples (9 in-clade, 31 out-of-clade). Per-query outputs are provided in Table S58 and Figure S36.

QUANTIFICATION AND STATISTICAL ANALYSIS

All statistical analyses were performed in R using the R packages listed in the Key Resources Table. Statistical details for each analysis, including the statistical test, exact sample size (n), the unit represented by n, and the adjustment method, are provided in the corresponding Results section, Methods section, and supplemental data tables. The center line indicates the median, and boxes indicate the interquartile range. Pairwise Wilcoxon rank-sum tests were employed to assess α-diversity differences between countries and IBD stages utilizing the rstatix R package (v0.7.3), with Benjamini-Hochberg FDR correction. To assess differential abundance across the three IBD stages on a 117,671-sample $\times$ 203-taxon modeling subset, we performed linear mixed-effects models, ALDEx2, ANCOM-BC2, MaAsLin2, and LEfSe. Blocked Wilcoxon rank-sum tests were employed to assess pathway-level differential abundance utilizing the coin R package (v1.4.3), with cohort project as the blocking variable. A random forest classifier was employed to discriminate IBD from healthy samples utilizing the randomForest R package (v4.7.1.2), with AUC evaluation and 10-fold cross-validation for feature selection.

Cell Reports Medicine, Volume *7*

Supplemental information

Global gut microbiome atlas identifies epidemiologic-stage-specific signatures in inflammatory bowel disease

Jinxia Zhai (翟锦霞), Yingjie Li (李迎杰), Jiameng Liu (刘佳梦), Xinyi Su (苏心怡), Runze Cui (崔润泽), Dianyu Zheng (郑殿宇), Yuhan Sun (孙羽晗), Jingsheng Yu (于景盛), and Cong Dai (戴聪)

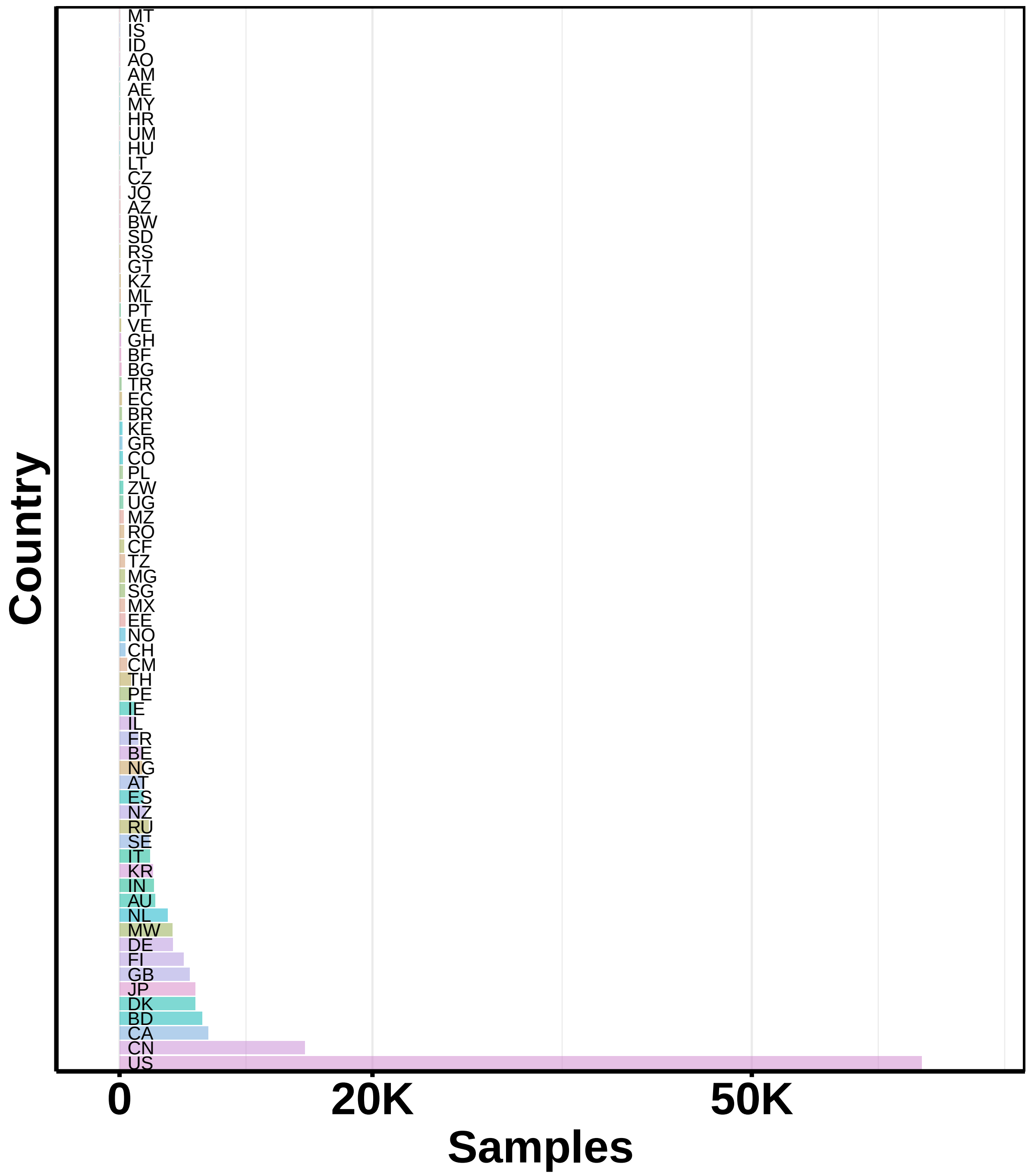

Figure S1. Regional structure. Related to Figure 1

A bar plot illustrating the number of samples contributed by each of the 70 countries analyzed. The x-axis indicates the total number of samples, and the y-axis lists all countries evaluated.

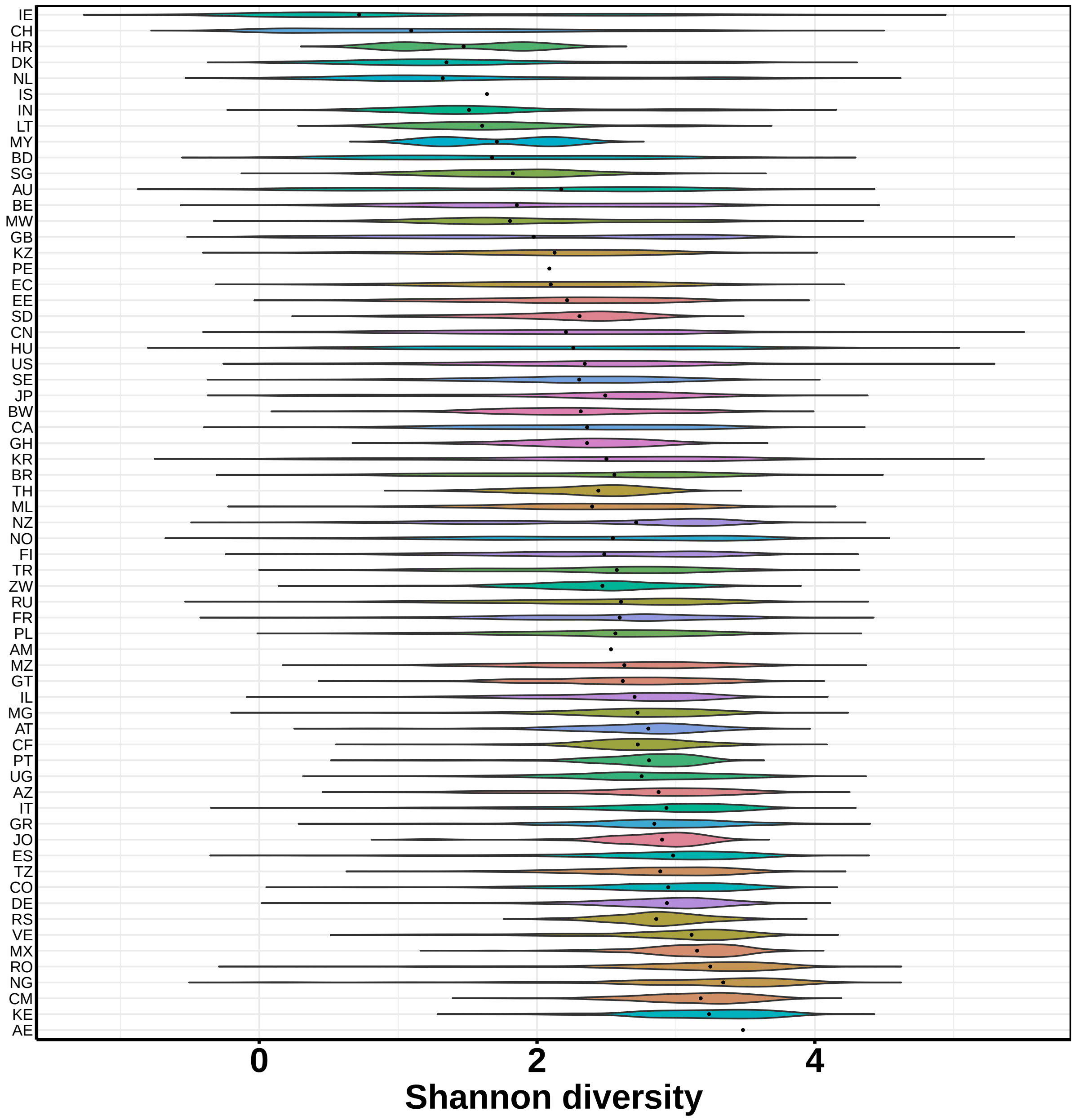

Figure S2. Regional structure. Related to Figure 1

Violin plots showing the distributions of observed Shannon index across the 70 countries. Vertical lines within each violin indicate the median values.

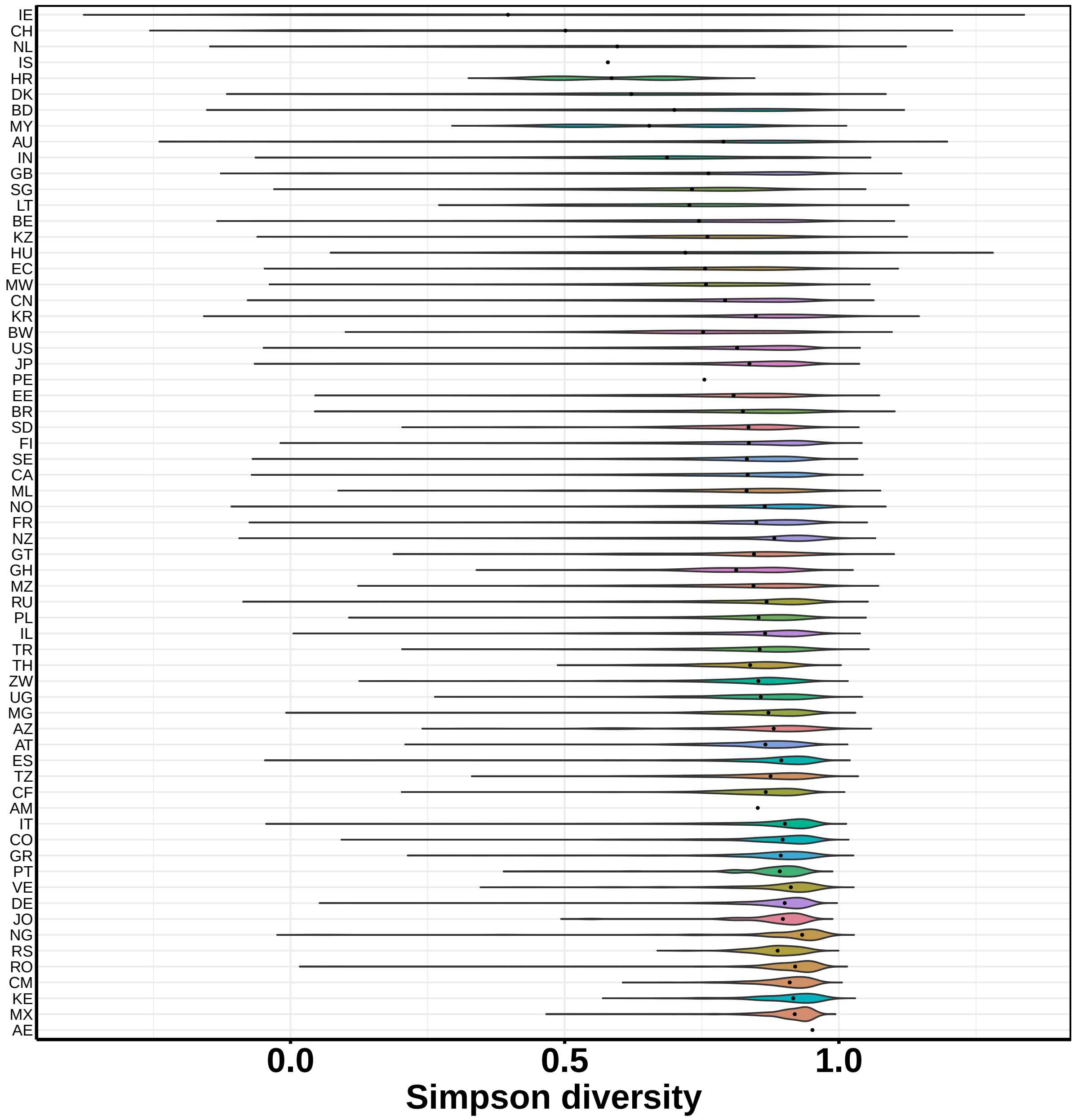

Figure S3. Regional structure. Related to Figure 1

Violin plots showing the distributions of observed Simpson index across the 70 countries. Vertical lines within each violin indicate the median values.

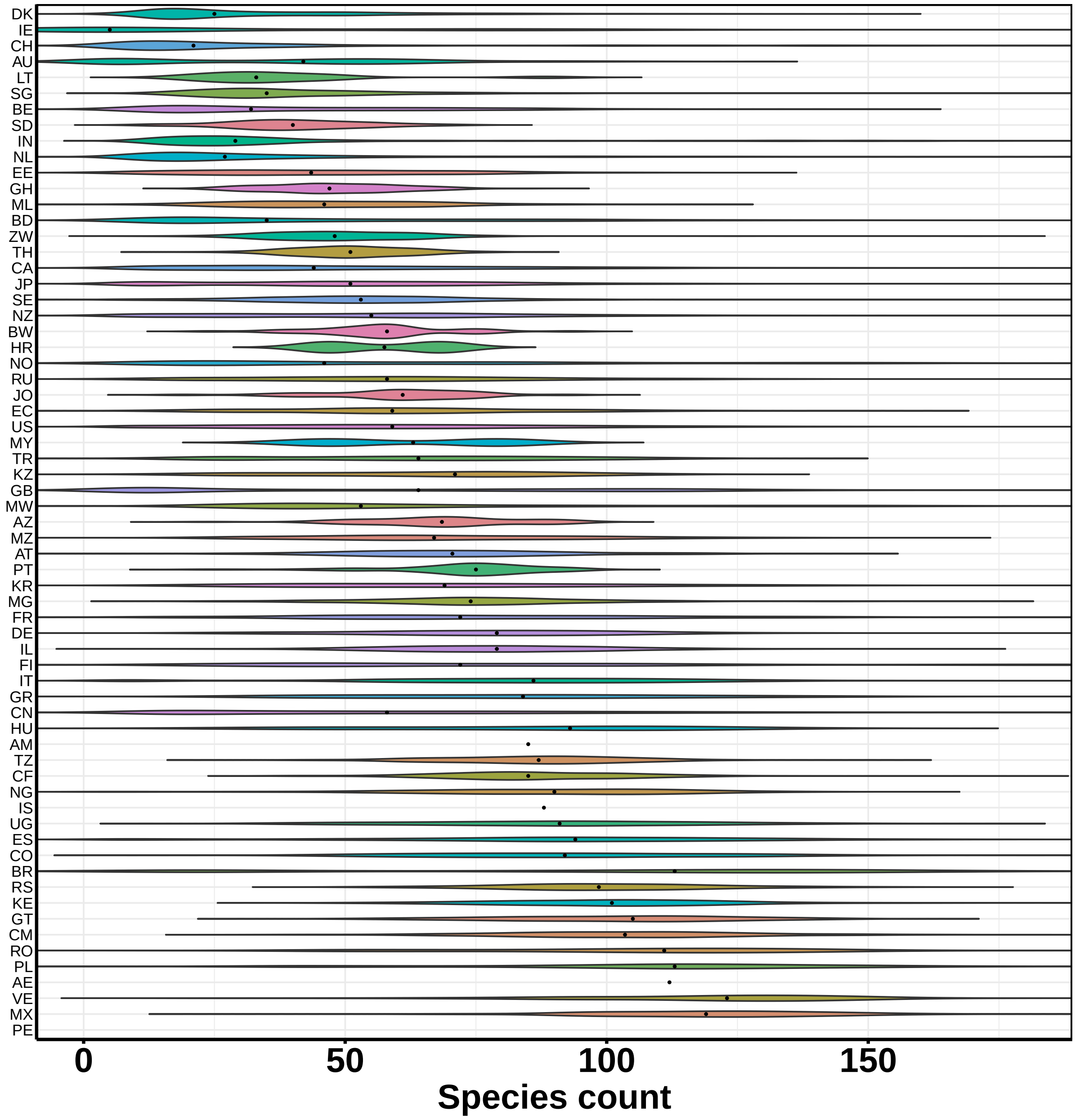

Figure S4. Regional structure. Related to Figure 1

Violinplots showing the distributions of observed species count values across the 70 countries . Vertical lines within each violin indicate the median values.

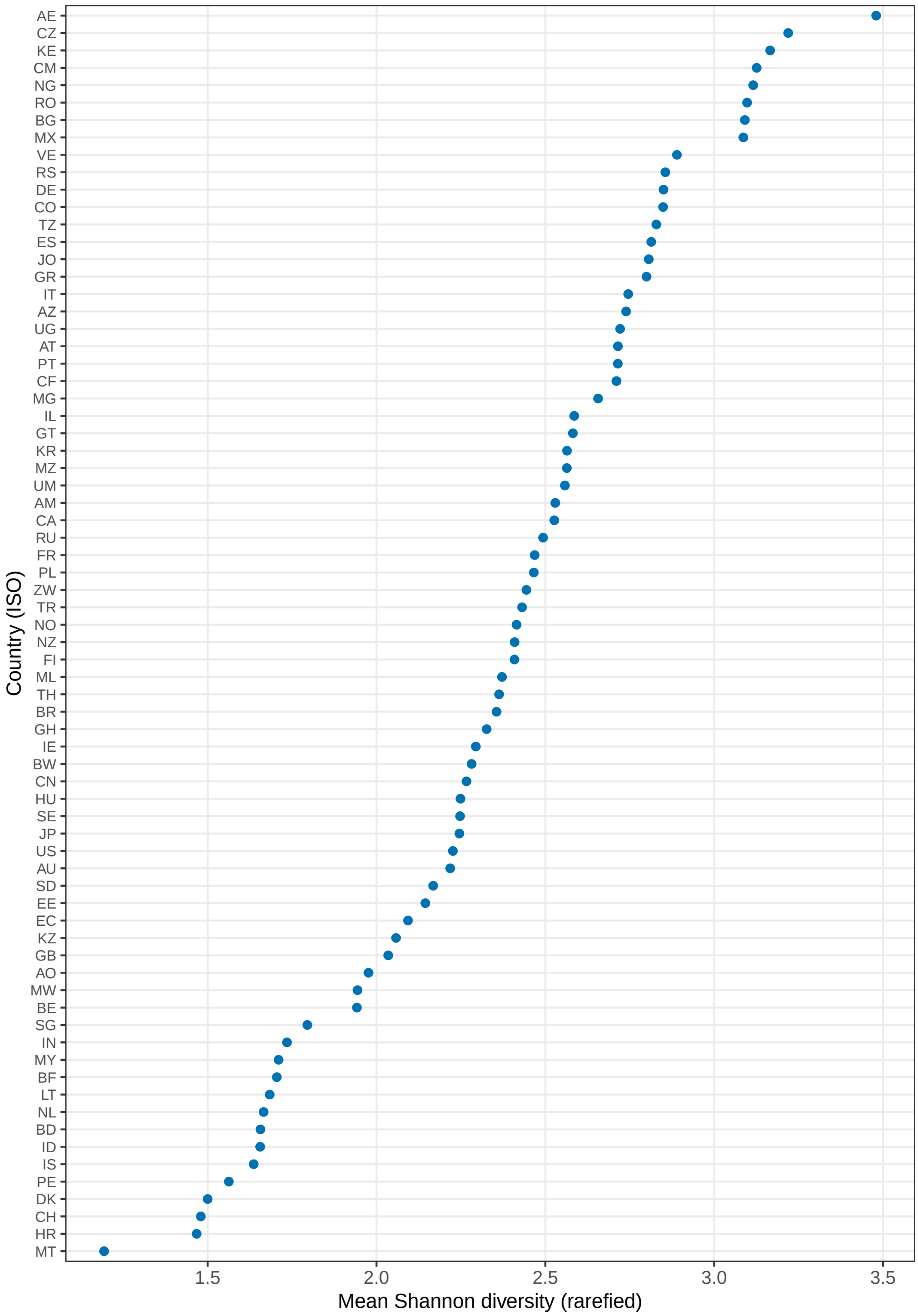

Figure S5. Rarefaction diversity estimates. Related to Figure 1

The x-axis denotes Shannon diversity, and the y-axis lists all countries evaluated in the rarefaction analysis. Each point represents the mean α diversity calculated across 1,000 iterations (see Methods). Statistical estimates were derived using the R t.test function.

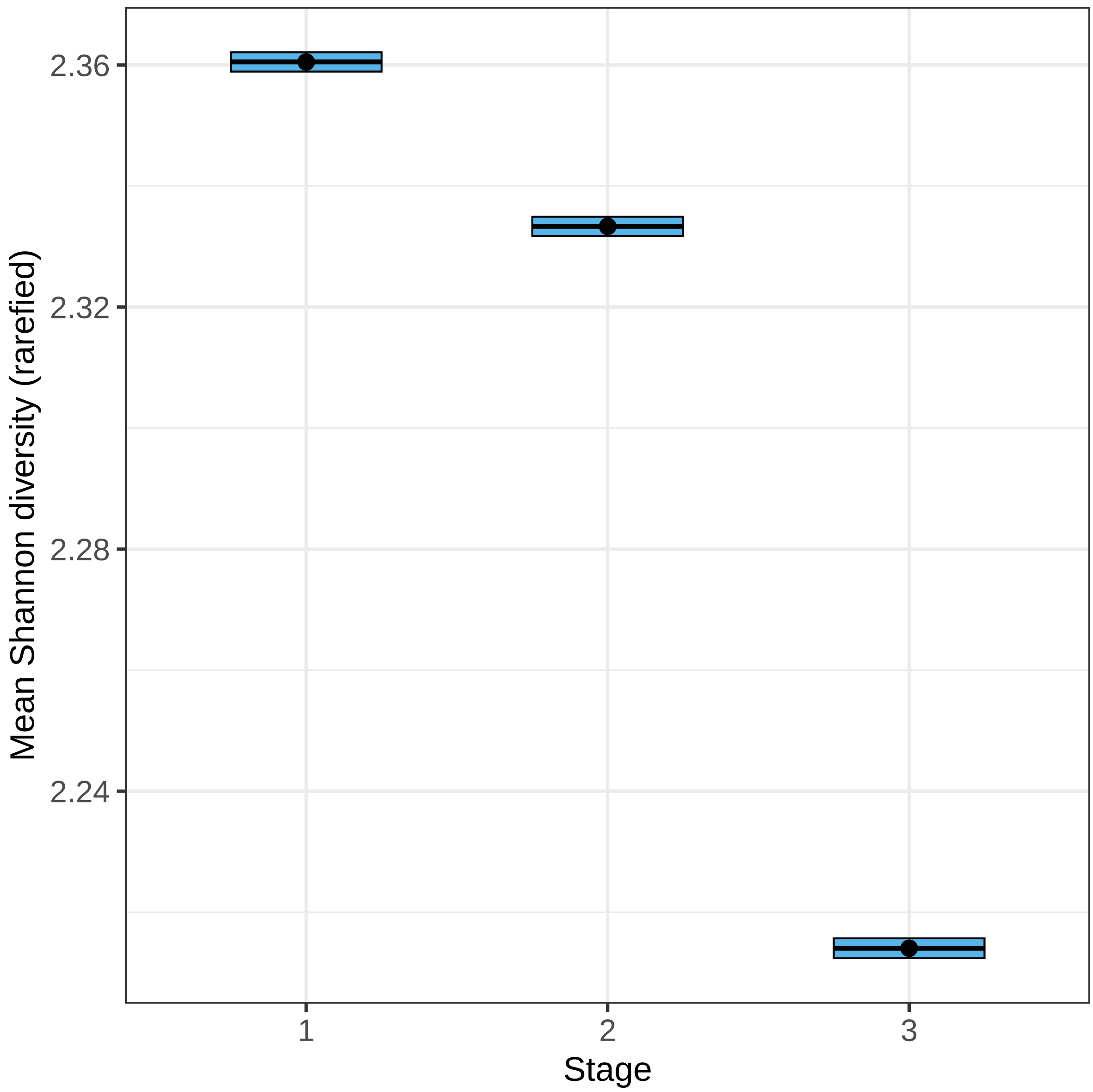

Figure S6. Rarefaction diversity estimates. Related to Figure 1

The y-axis denotes Shannon diversity, and the x-axis lists regions stratified by the three IBD epidemiologic stages evaluated in the rarefaction analysis. Each point represents the mean α diversity calculated across 1,000 iterations (see Methods). Statistical estimates were computed using the R t.test function.

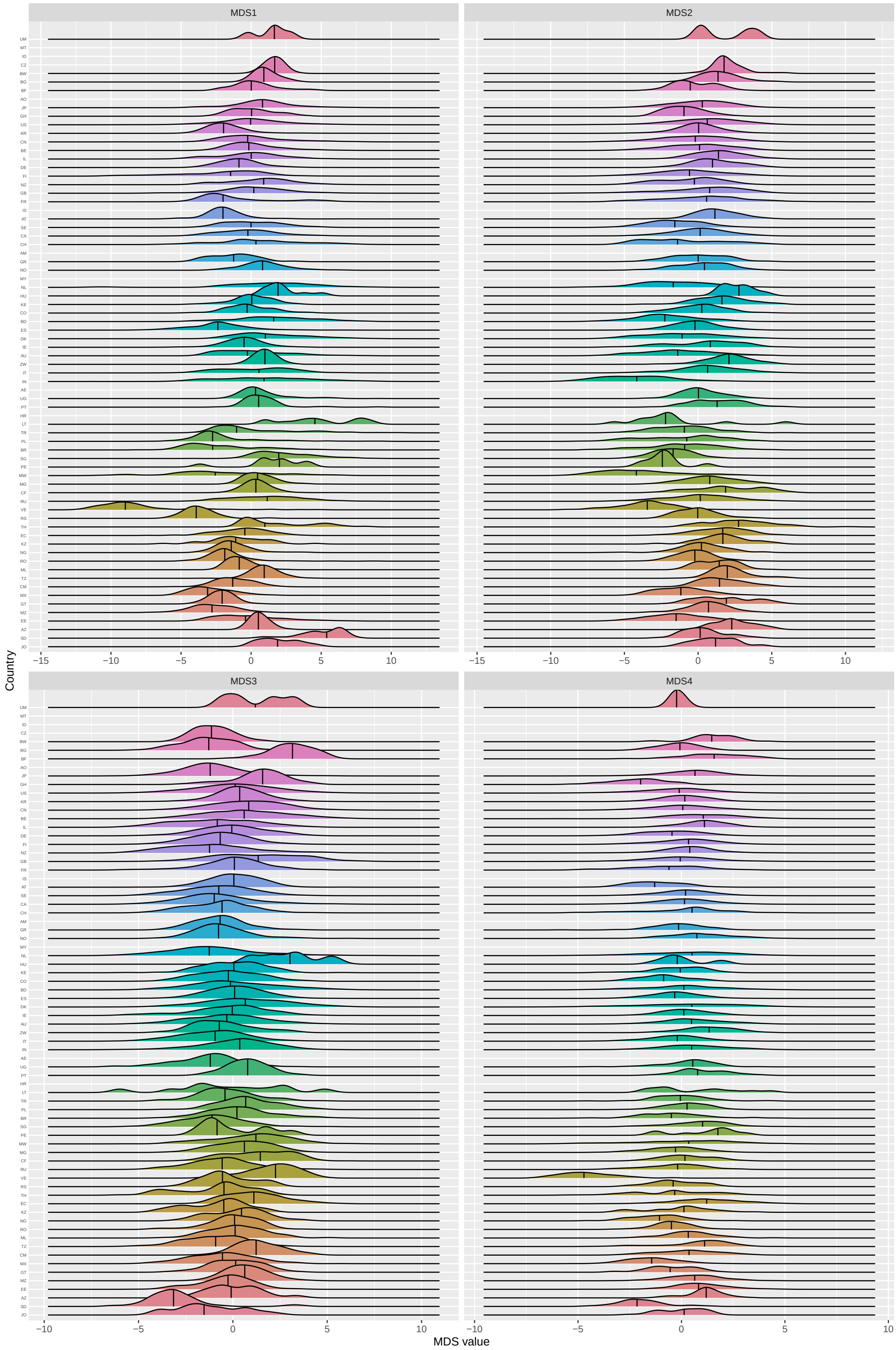

Figure S7. Ordination results. Related to Figure 1

This figure comprises a series of panels illustrating the results of a principal coordinates analysis (PCoA) of samples from 70 countries. Factors MDS1 through MDS4 are shown. Each panel depicts a single factor; the x-axis indicates the value of that factor, and the y-axis represents the relative frequency of that value within each country.

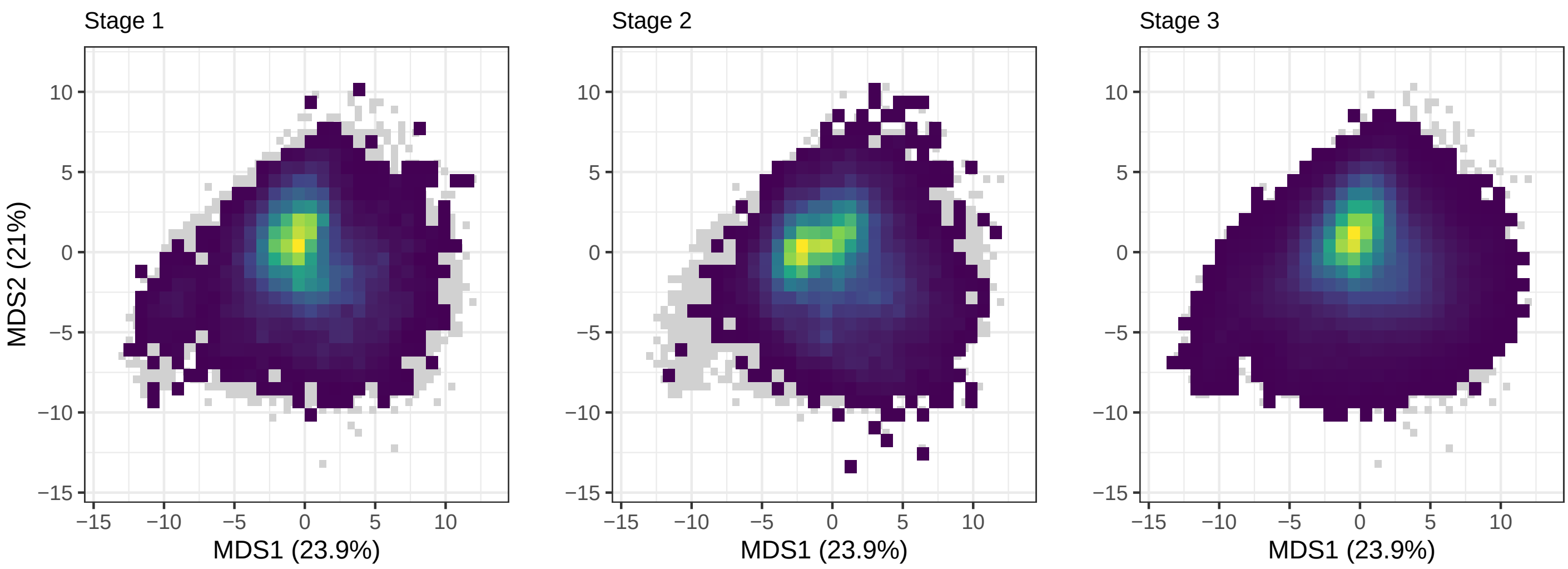

Figure S8. Alternate ordination plot. Related to Figure 1

This figure presents the results of a principal coordinates analysis of samples stratified by the three IBD epidemiologic stages. The layout is analogous to that of Figure 1I but is rendered at higher resolution (a 50-by-50 grid rather than a 30-by-30 grid). Patterns along the first and second MDS axes are shown. Each point represents an individual sample, and each panel includes only samples from a single stage. To facilitate evaluation of regions with substantial point overlap, a heatmap representation is used instead of a scatter plot: yellow areas indicate regions of higher sample density, whereas deep purple areas denote regions with fewer (but non-zero) samples. The gray shading overlaid on each panel indicates the area occupied by all samples across all stages.

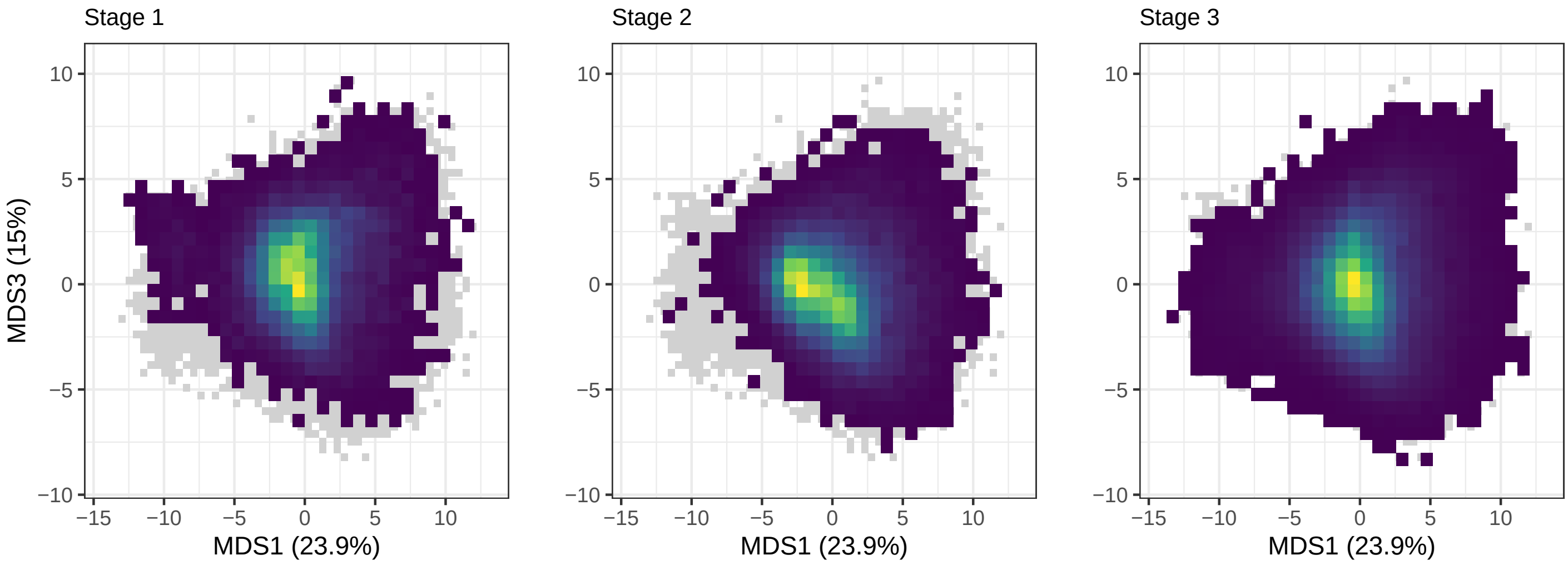

Figure S9. Alternate ordination plot. Related to Figure 1

Similar to Supplementary Figure 8,but showing axes MDS 1vs MDS3.

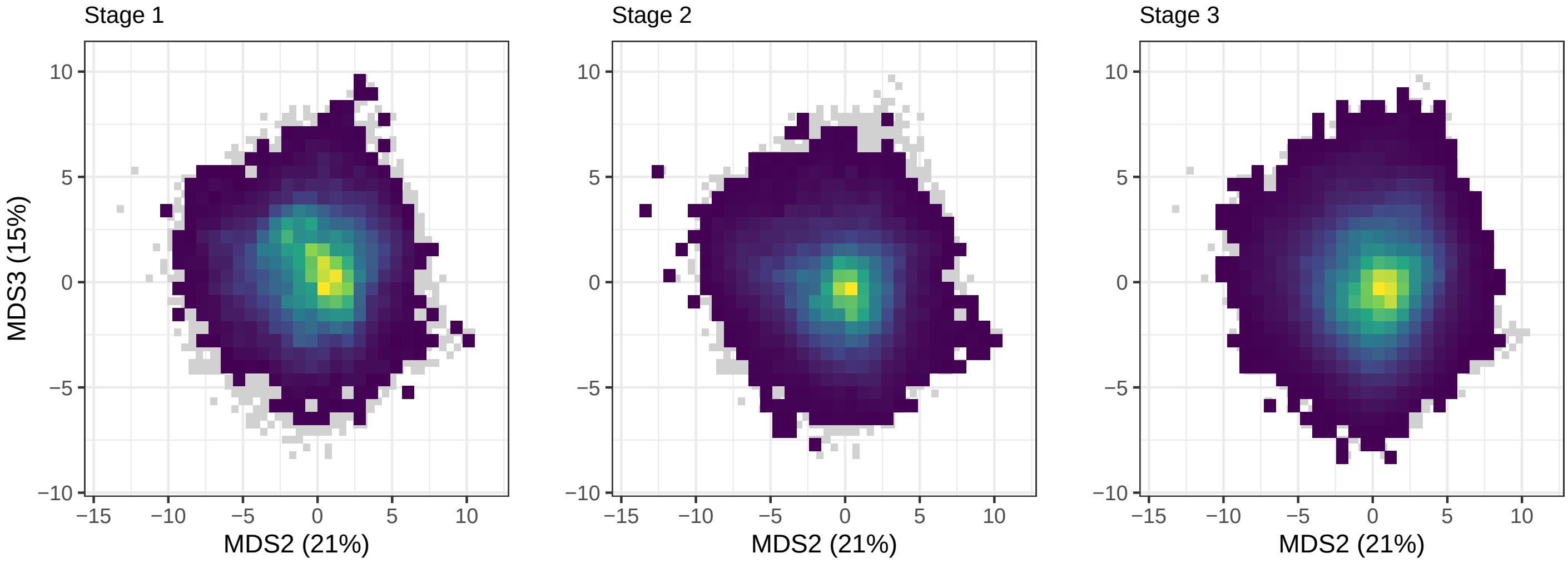

Figure S10. Alternate ordination plot. Related to Figure 1

Similar to Supplementary Figure 8,but showing axes MDS 2vs MDS3.

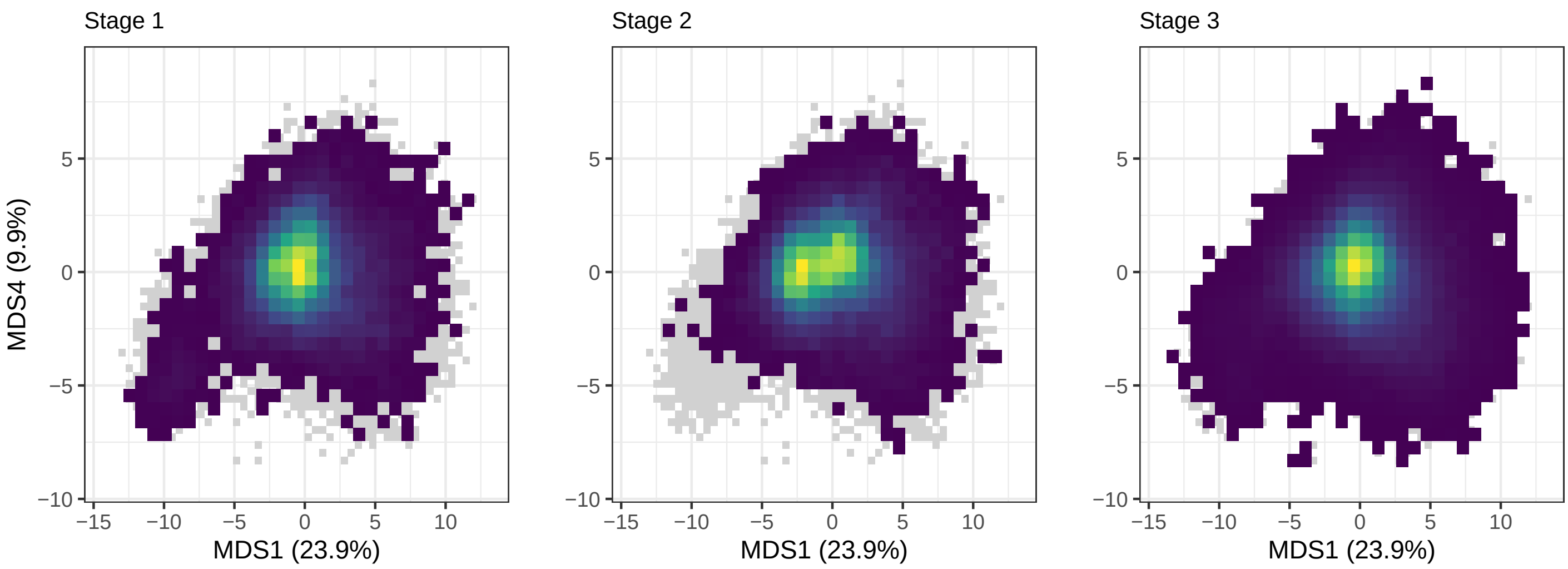

Figure S11. Alternate ordination plot. Related to Figure 1

Similar to Supplementary Figure 8,but showing axes MDS 1vs MDS4.

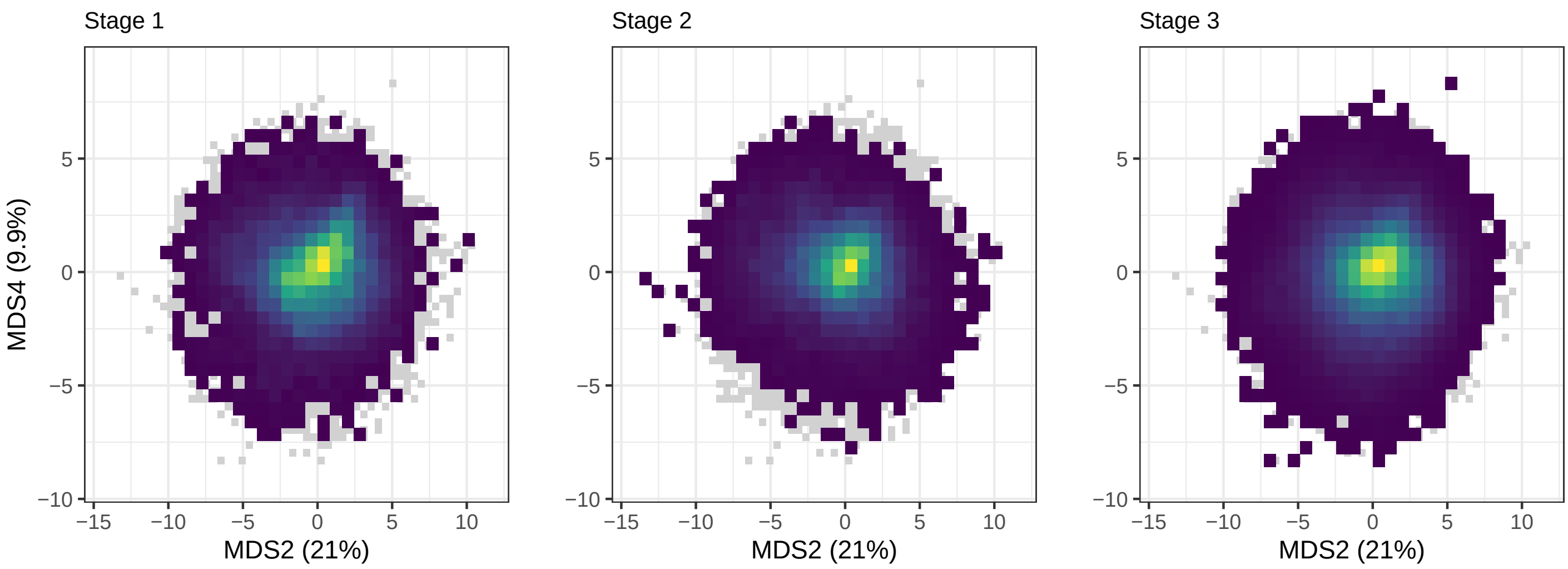

Figure S12. Alternate ordination plot. Related to Figure 1

Similar to Supplementary Figure 8,but showing axes MDS 2vs MDS4.

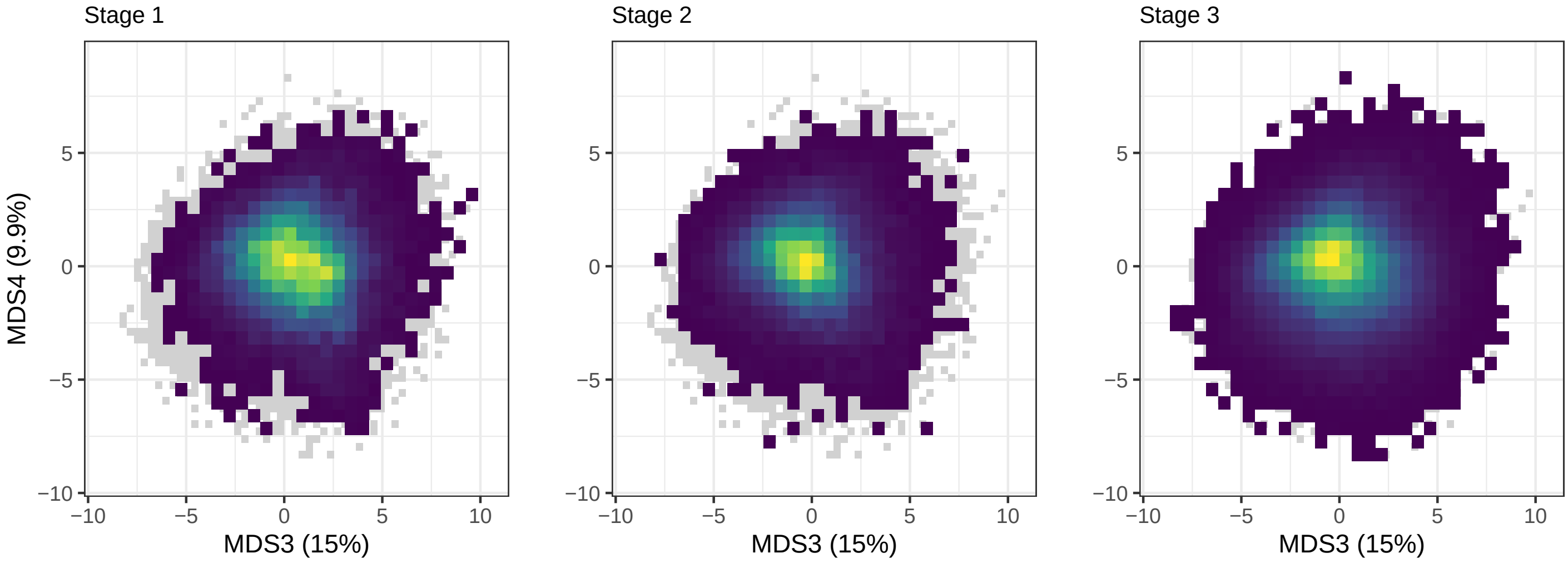

Figure S13. Alternate ordination plot. Related to Figure 1

Similar to Supplementary Figure 8,but showing axes MDS 3vs MDS4.

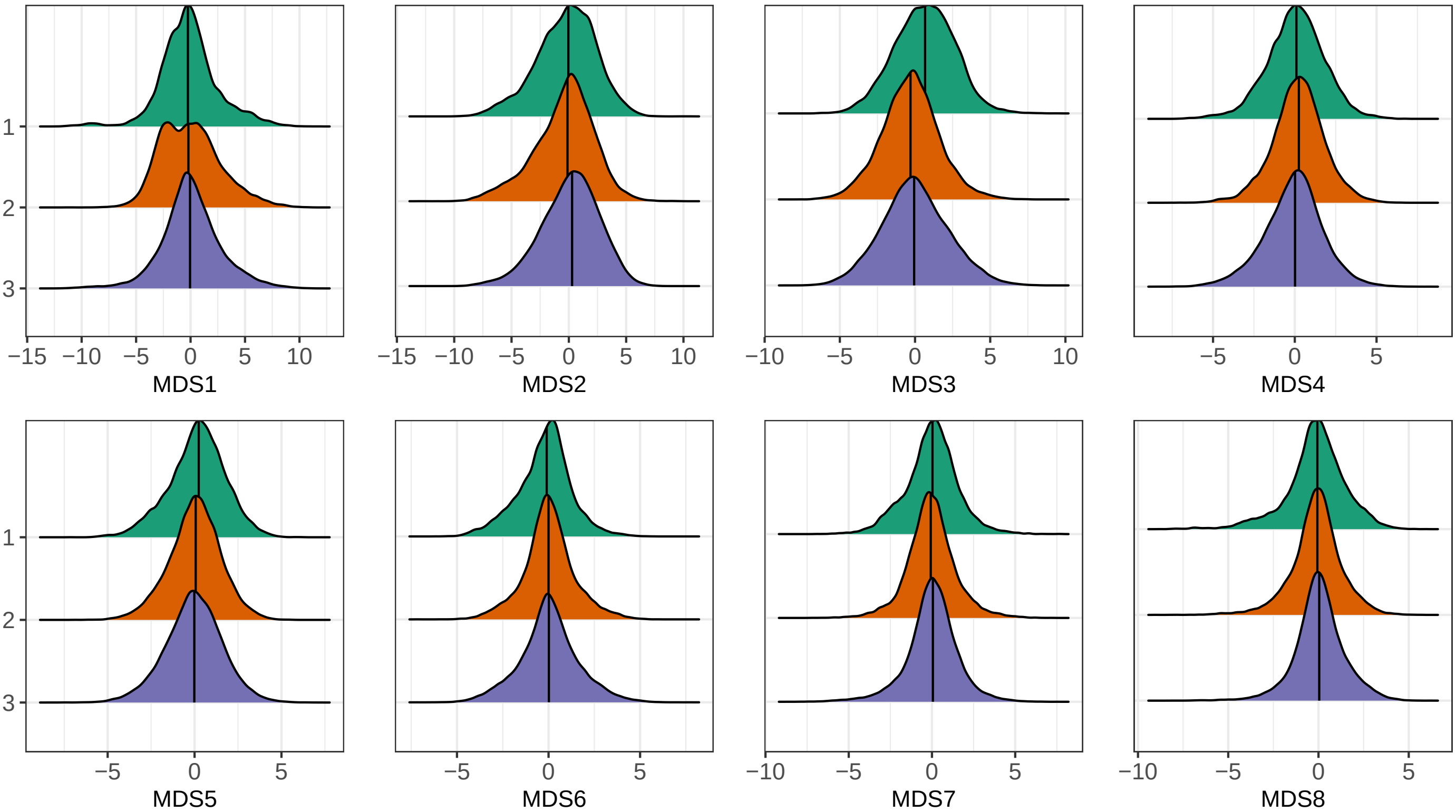

Figure S14. Ordination results. Related to Figure 1

This figure comprises a series of panels illustrating the results of a principal coordinates analysis of samples stratified by the three IBD epidemiologic stages. This analysis extends the data shown in Figure 1J by displaying factors MDS1 through MDS8. Each panel depicts a single factor; the x-axis indicates the value of that factor, and the y-axis represents the relative frequency of that value within the corresponding region.

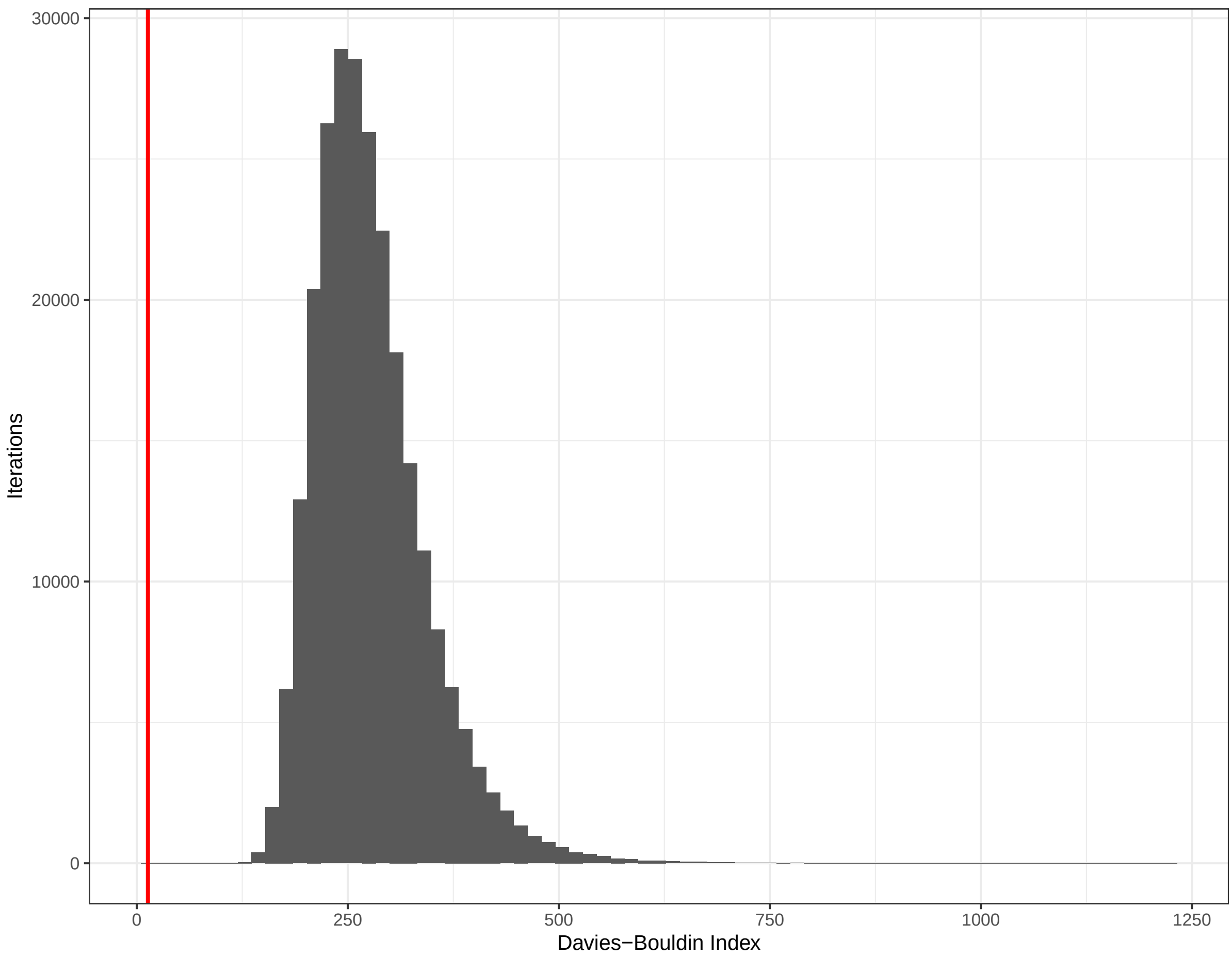

Figure S15. Cluster strength analysis. Related to Figure 1

This histogram presents the results of a bootstrap analysis assessing the strength of clustering by the three IBD epidemiologic stages in ordination space. In each iteration (250,000 in total), the regional labels corresponding to stages 1, 2, and 3 were randomly permuted across all samples, and a single score (the DaviesCBouldin index) was calculated for that iteration. The x-axis indicates the score, and the y-axis denotes the number of iterations falling within each bin. The red vertical line indicates the clustering score (13.26) obtained using the true stage assignments.

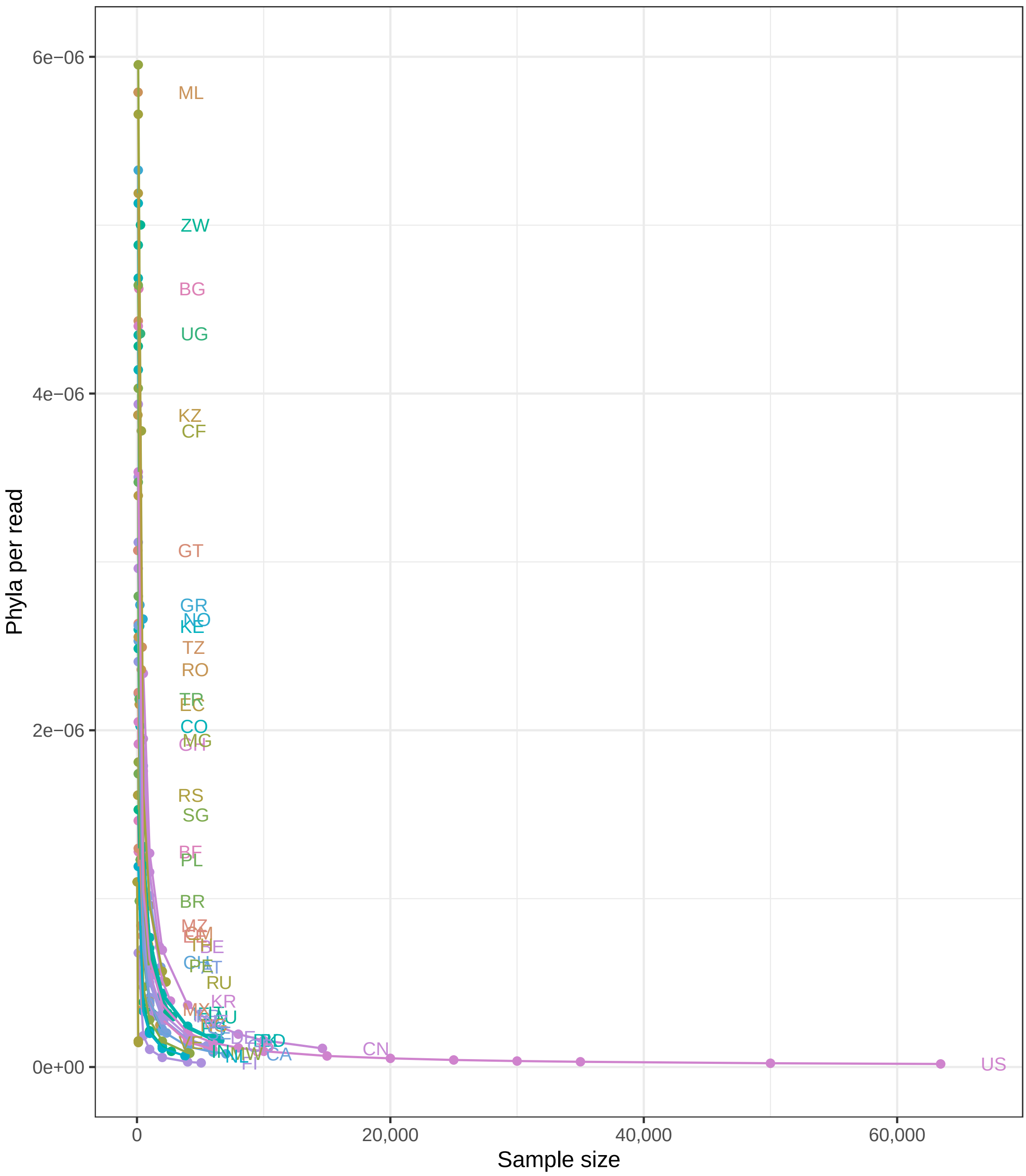

Figure S16. Discovery curves at the country level across 70 countries. Related to Figure 2

The x-axis indicates the number of samples, and the y-axis denotes the number of phyla per read (phylum per read). Similar to the inset in Figure 2A, increasing numbers of microbiome samples were progressively subsampled from each country. For each sample size (shown on the x-axis), we calculated the number of unique phyla observed and the number of sequencing reads per sample. We then computed the mean phyla per read (y-axis) by dividing the mean number of unique phyla by the mean read count for each sample size.

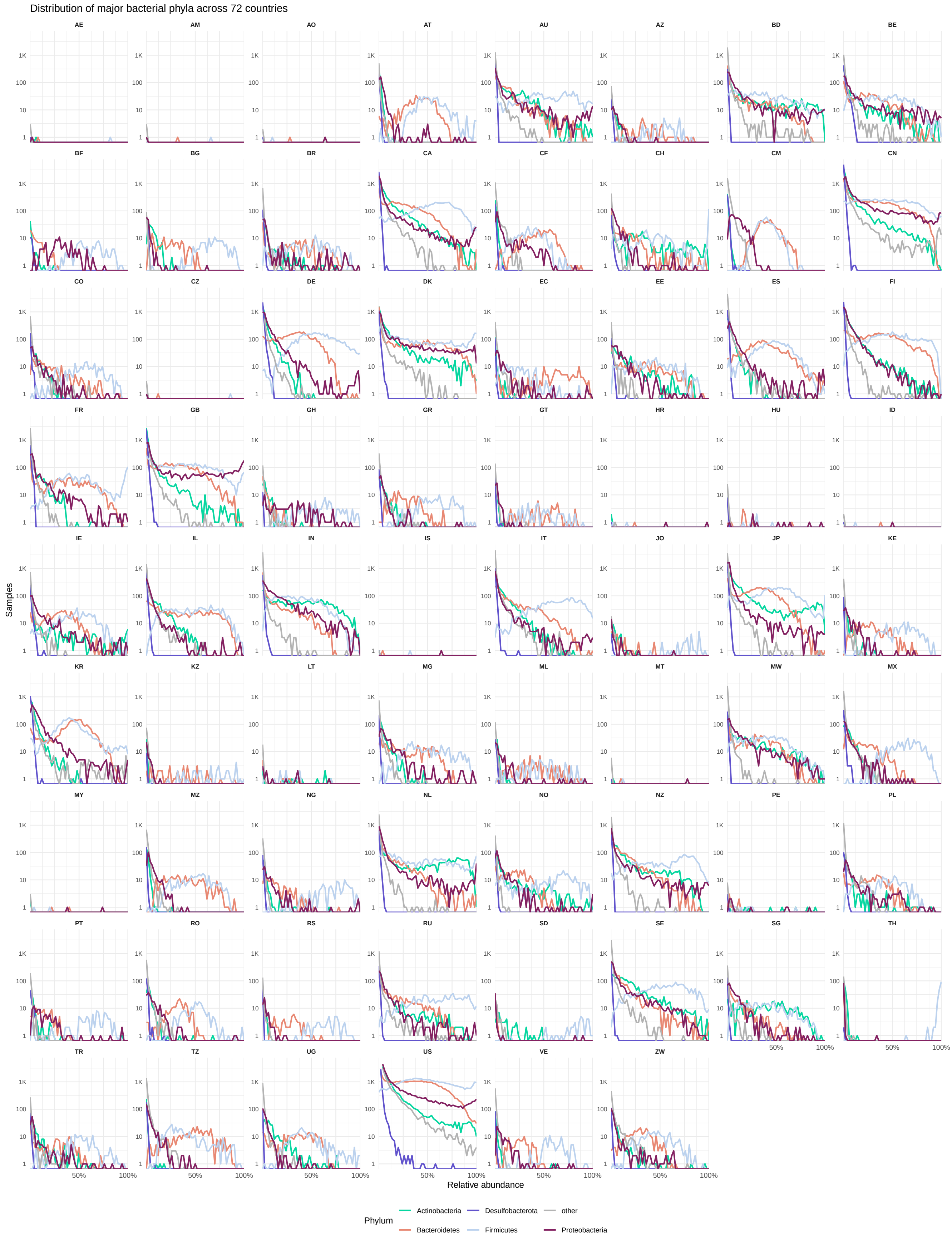

Figure S17. Relative abundance of dominant phyla. Related to Figure 2

This figure illustrates the distribution of relative abundances of the most prevalent phyla in the compendium. Each panel visualizes all samples from a single country. The x-axis indicates the relative abundance of each phylum, and the y-axis denotes the number of samples exhibiting the corresponding relative abundance. Each line represents a single phylum and is distinguished by color.

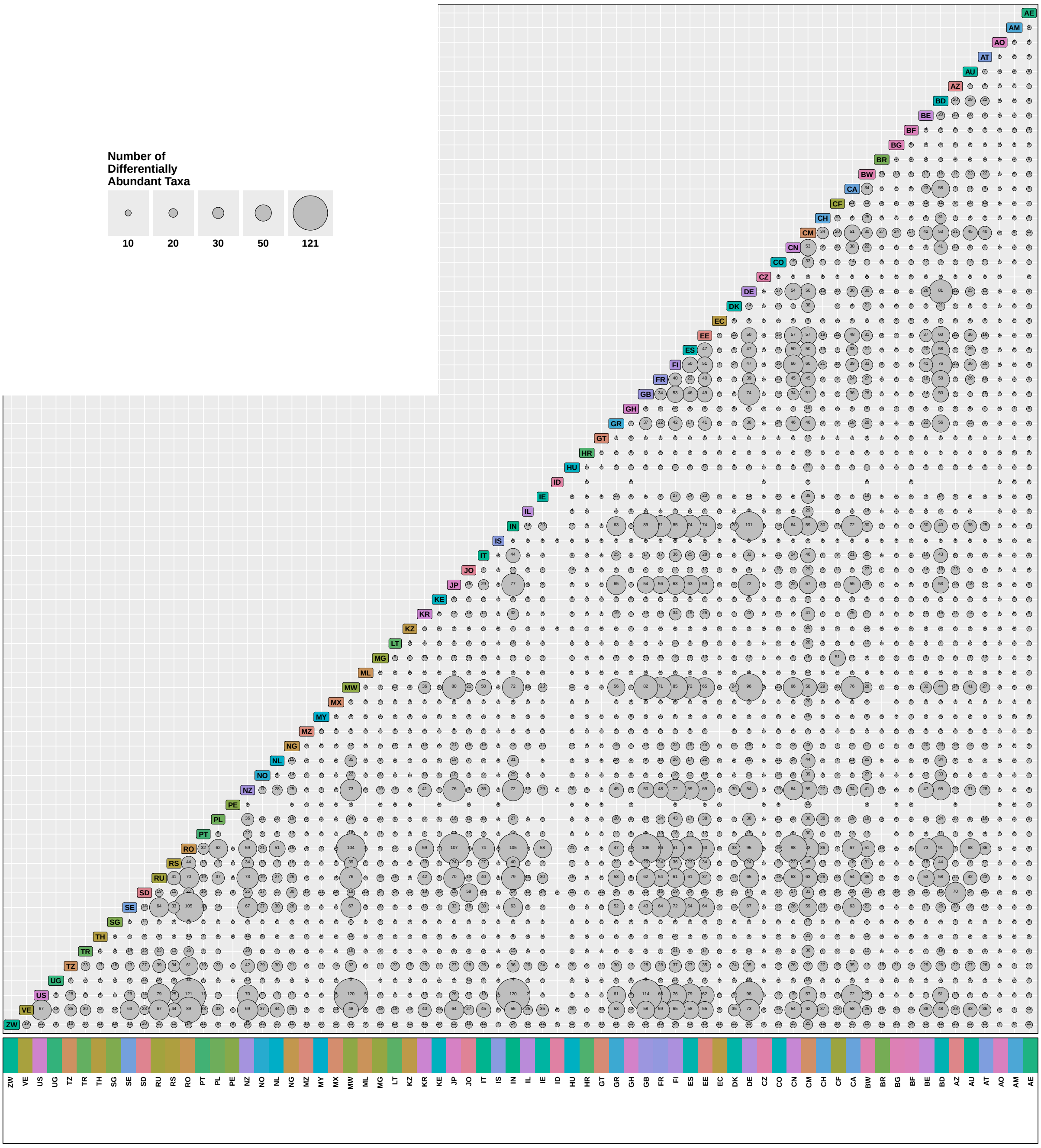

Figure S18. Taxa are differentially abundant between countries. Related to Figure 3

203 taxa were selected to test for differential abundance between countries. The x-and y- axes are each colored by country; at each intersection, the size of the circle and the number below it indicate the number of taxa that were significantly different between the two countries.

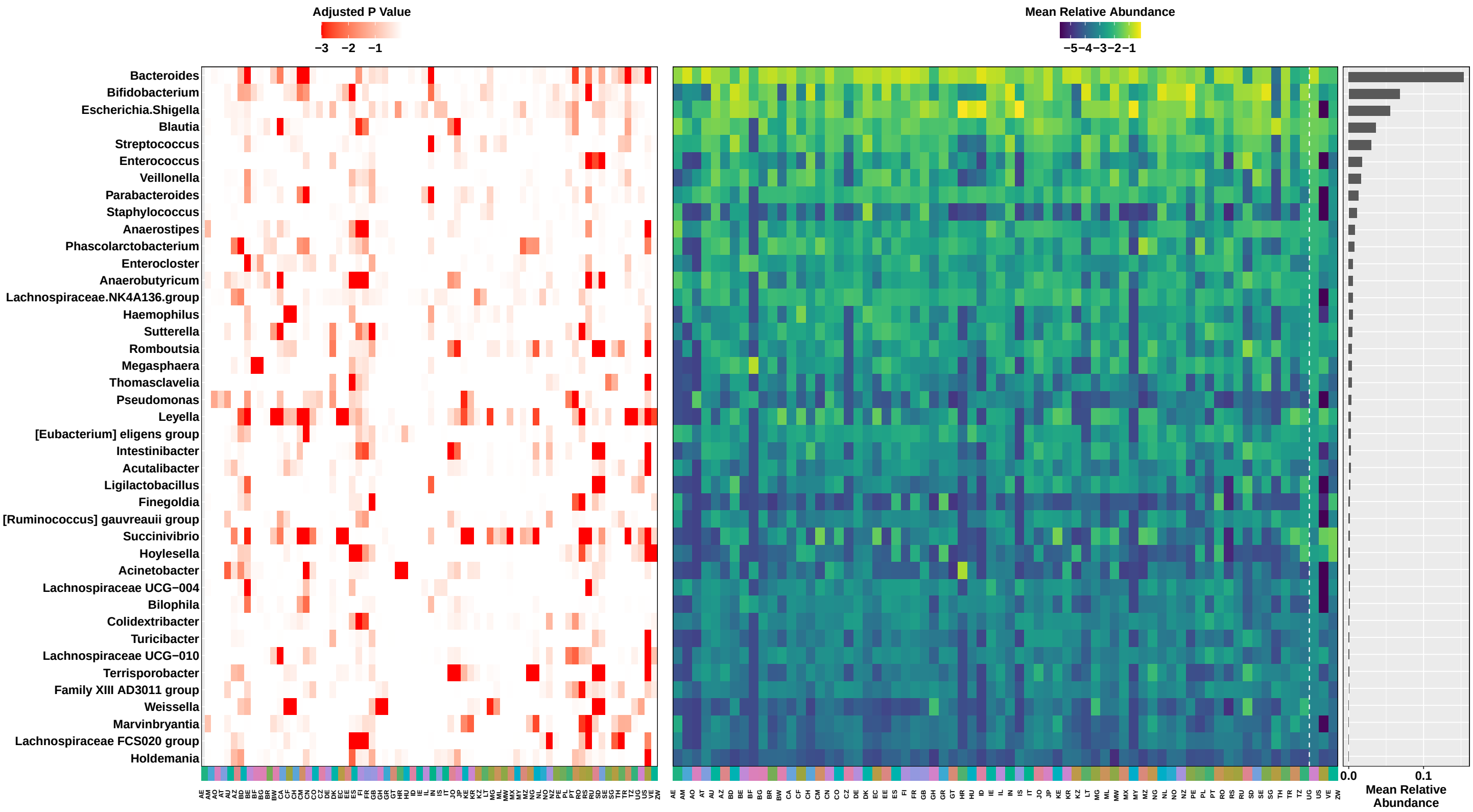

Figure S19. Taxa are differentially abundant between countries. Related to Figure 3

The red-white heat map illustrates adjusted p-values for country-wise differences when each country is compared to the United States. The y-axis lists all evaluated genera, and the x-axis lists all countries (using the same color scale as in Supplementary Figure 18). Each cell represents the strength of the differential abundance result for that taxon. The blue-green heat map depicts the mean relative abundance (log10) of each taxon in each country, as indicated on the x-axis. The accompanying bar chart shows the mean relative abundance of each taxon averaged across all countries.

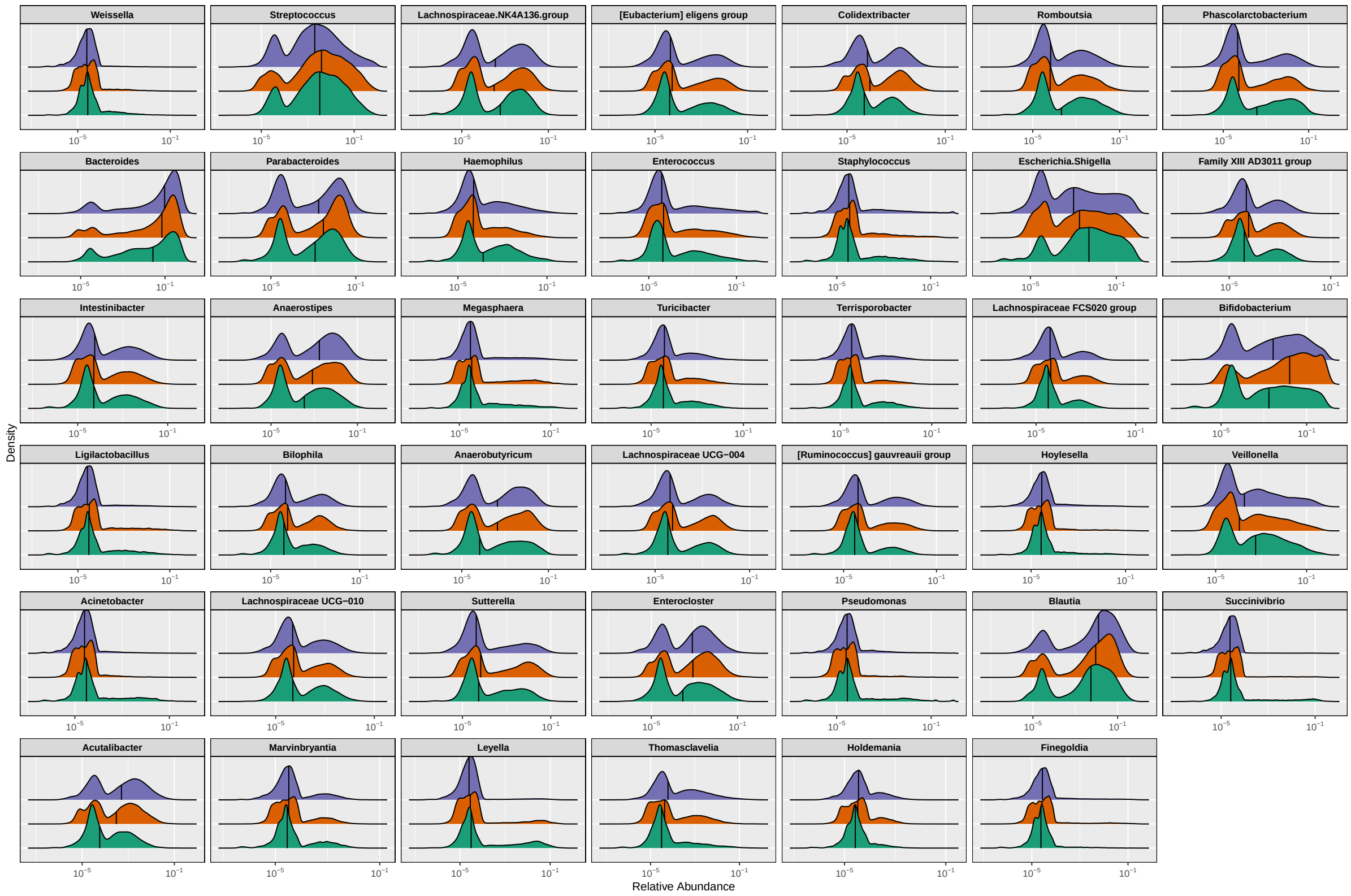

Figure S20. Relative abundance of differentially abundant taxa across the three IBD stages.Related to Figure 3

Similar to Figure 3C, each panel illustrates the relative abundance of a single taxon. Shown here are all taxa ranke 1-41 by average relative abundance. Within each panel, colored areas represent the distributions of samples from a single IBD stage. Thex-axis indicates the relative abundance of the specified taxon (log10-transformed), and the y-axis denotes the relative frequency with which that abundance is observed within the corresponding stage. Black vertical lines indicate the median.

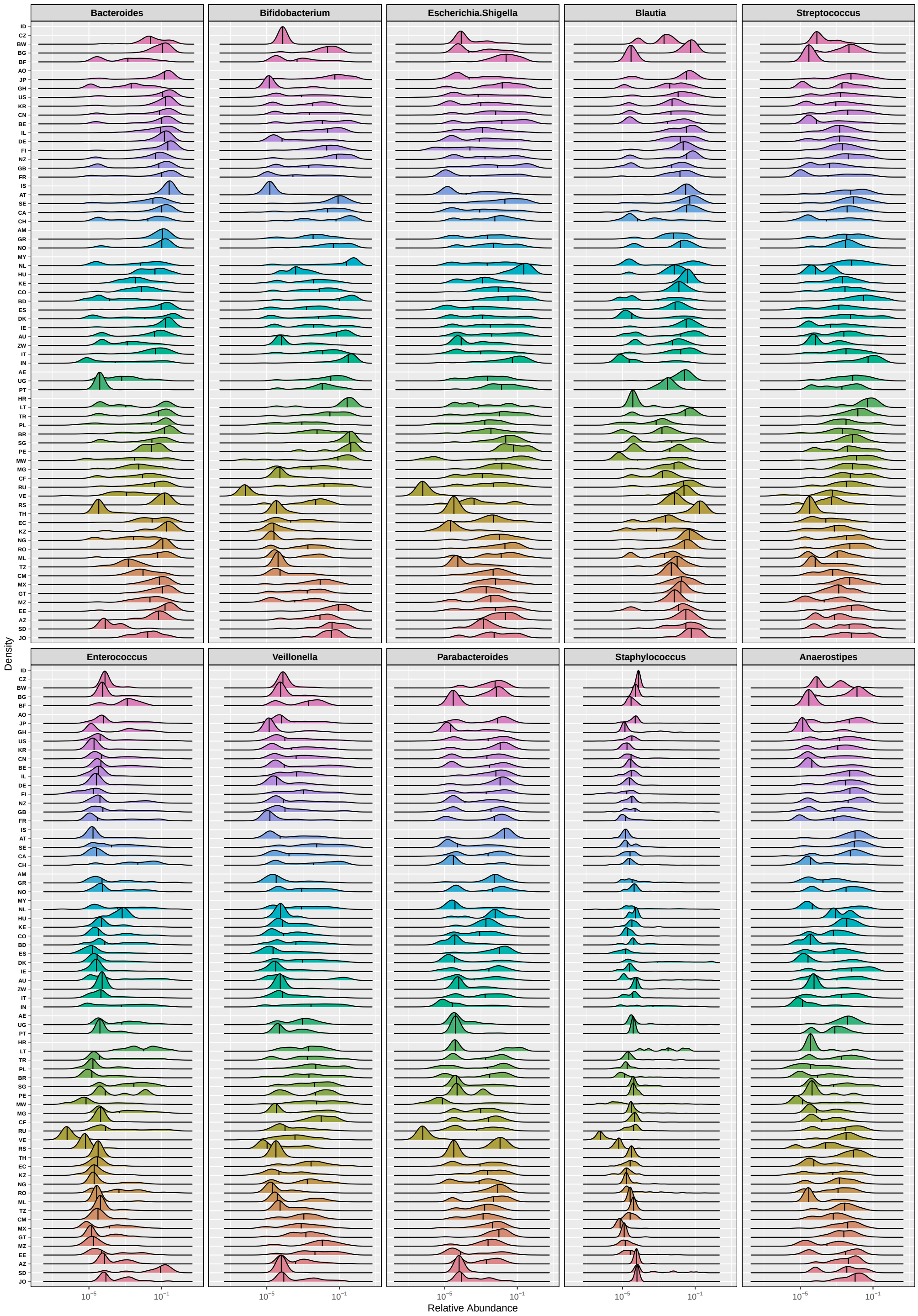

Figure S21. Taxa are differentially abundant between countries. Related to Figure 3

Each panel illustrates the relative abundance (log10) of one of the ten most abundant taxa. Each colored area indicates the distribution of samples from an individual country, using the same color scheme as SF18. The x-axis represents the (log10) relative abundance of the specified genus, and the y-axis shows the relative frequency with which that abundance is observed in the specified country. Black vertical lines indicate the median values.

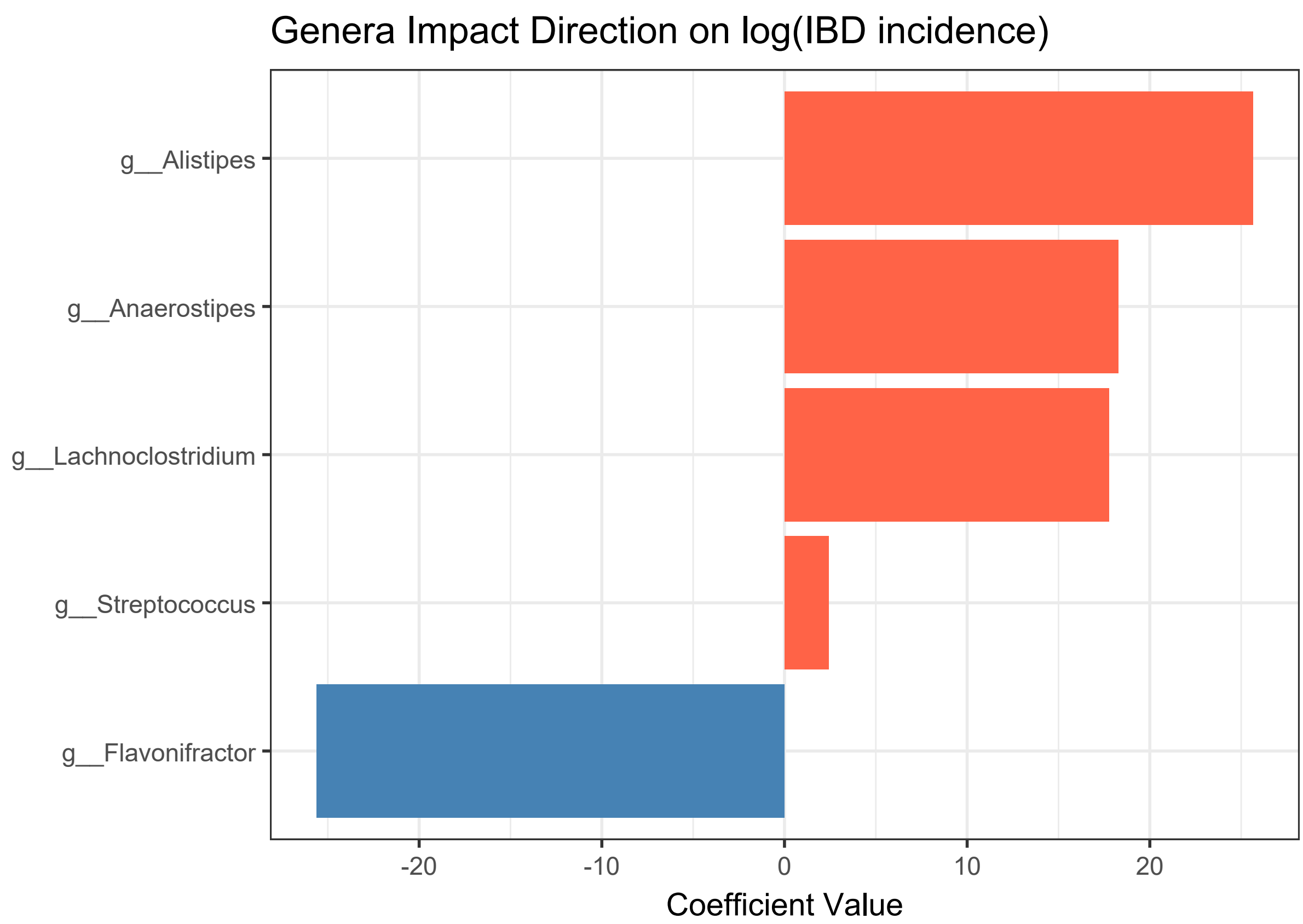

Figure S22. Direction and magnitude of genus-level effects on IBD incidence. Related to Figure 6

This figure shows the direction and magnitude of effects of core microbial genera on national IBD incidence, as estimated by a multivariable linear regression (MLR) model following LASSO-based feature selection. The analysis was performed using country-level mean relative abundances of genera from 70 countries, with the outcome modeled as log-transformed IBD incidence. The x-axis indicates the regression coefficient value, and the y-axis lists the genera retained in the final model. Positive coefficients (red bars) indicate genera whose higher relative abundance is associated with increased IBD incidence, whereas negative coefficients (blue bars) indicate genera associated with lower incidence. The absolute value of each coefficient reflects the strength of its contribution to the model.

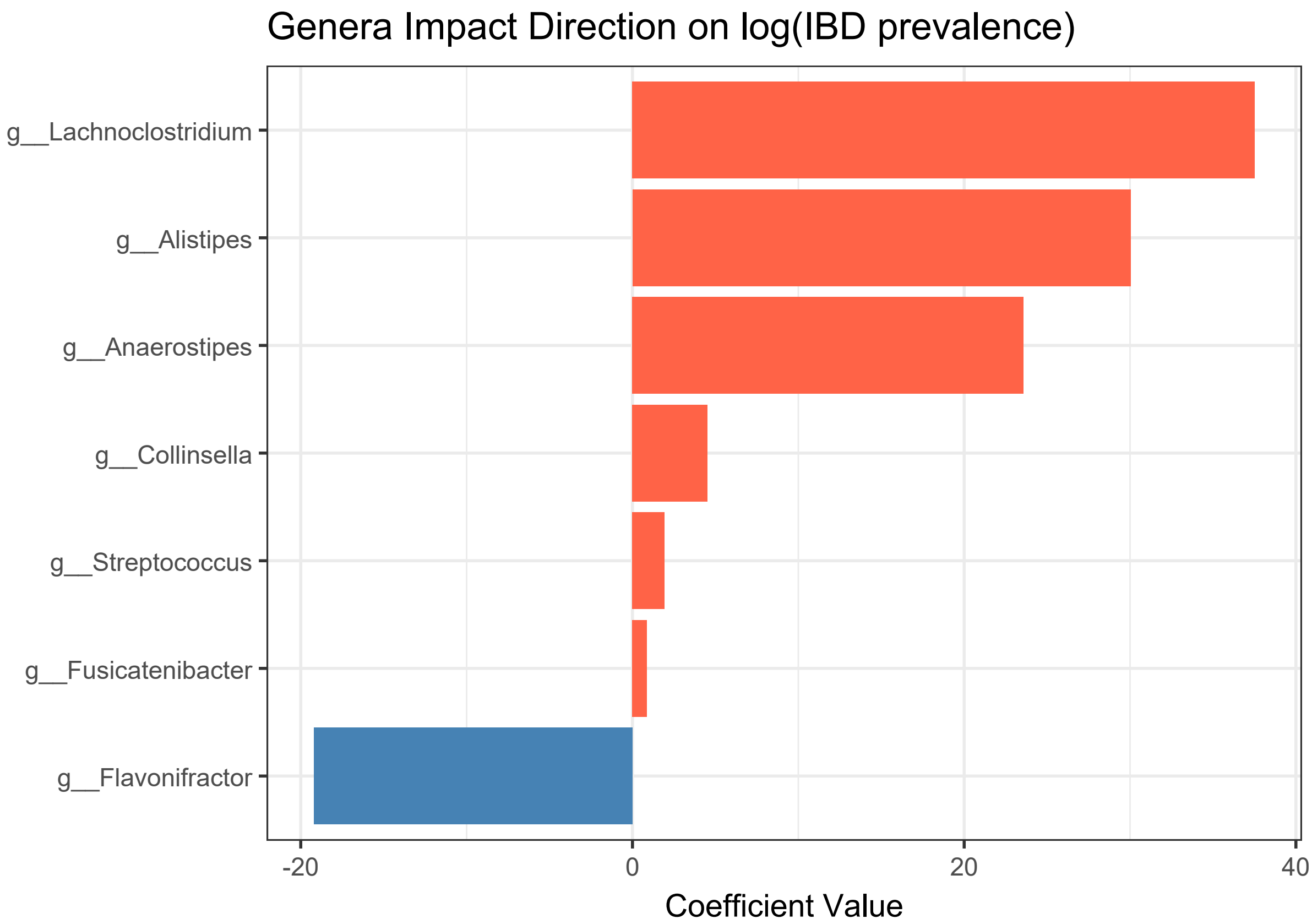

Figure S23. Direction and magnitude of genus-level effects on IBD prevalence. Related to Figure 6

Using the same LASSO-MLR framework as in Supplementary Figure 22, this figure illustrates the effects of selected microbial genera on national IBD prevalence (log-transformed). Country-level mean relative abundances were used as predictors. The x-axis denotes regression coefficient values, and the y-axis lists genera included in the final model. Positive coefficients (red bars) represent genera positively associated with higher IBD prevalence, while negative coefficients (blue bars) represent genera inversely associated with prevalence. Larger absolute coefficient values indicate stronger predictive contributions to the prevalence model.

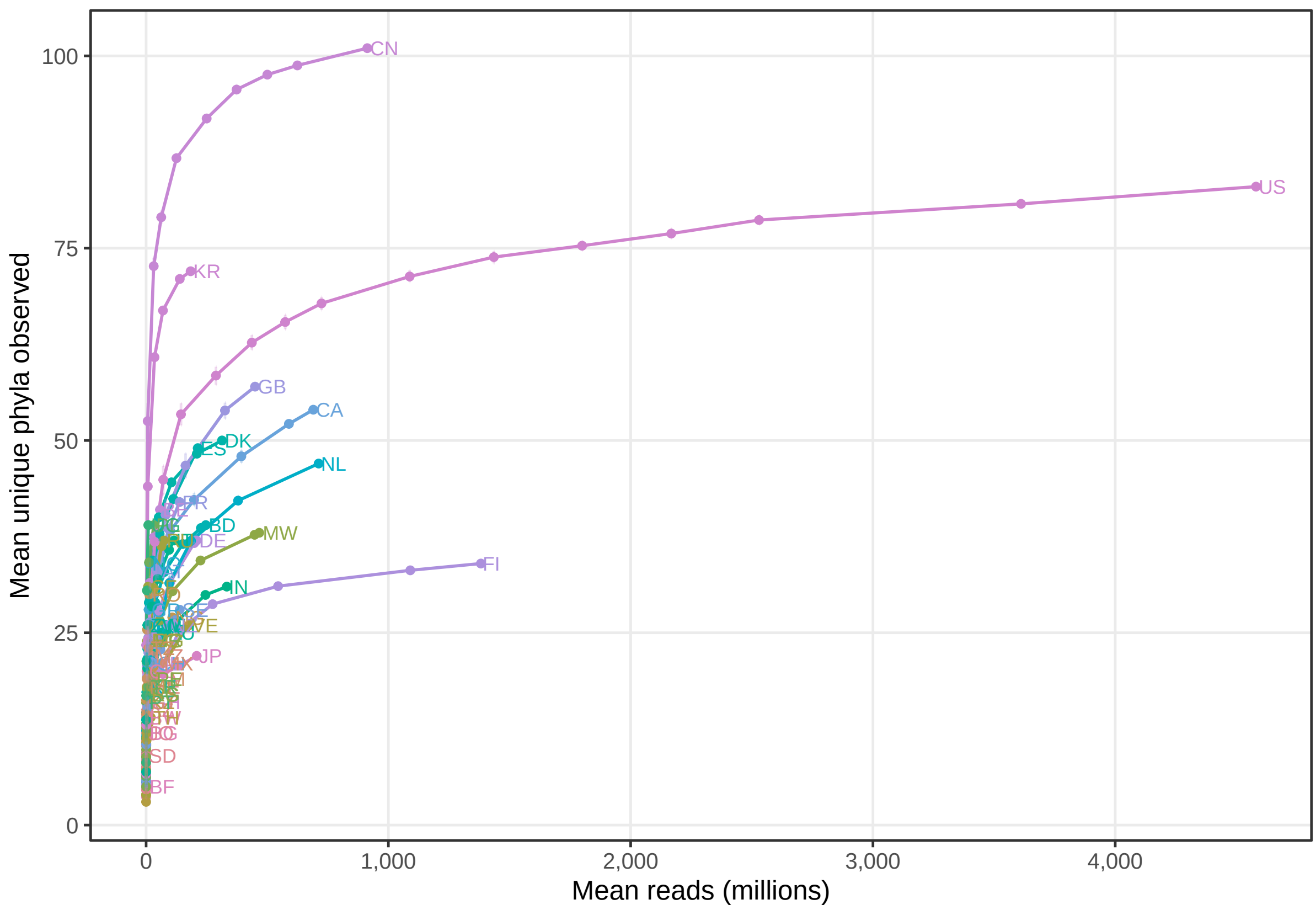

Figure S24. Country-level discovery curves. Related to Figure 2

This figure illustrates the number of phyla discovered as the number of sequencing reads increases. Using the same sampling scheme as in Figure 2A, increasing numbers of microbiome samples were progressively subsampled from each country. For each sample size, we calculated the mean number of sequencing reads in the selected samples (x-axis) and the mean number of unique phyla observed in the selected samples (y-axis).

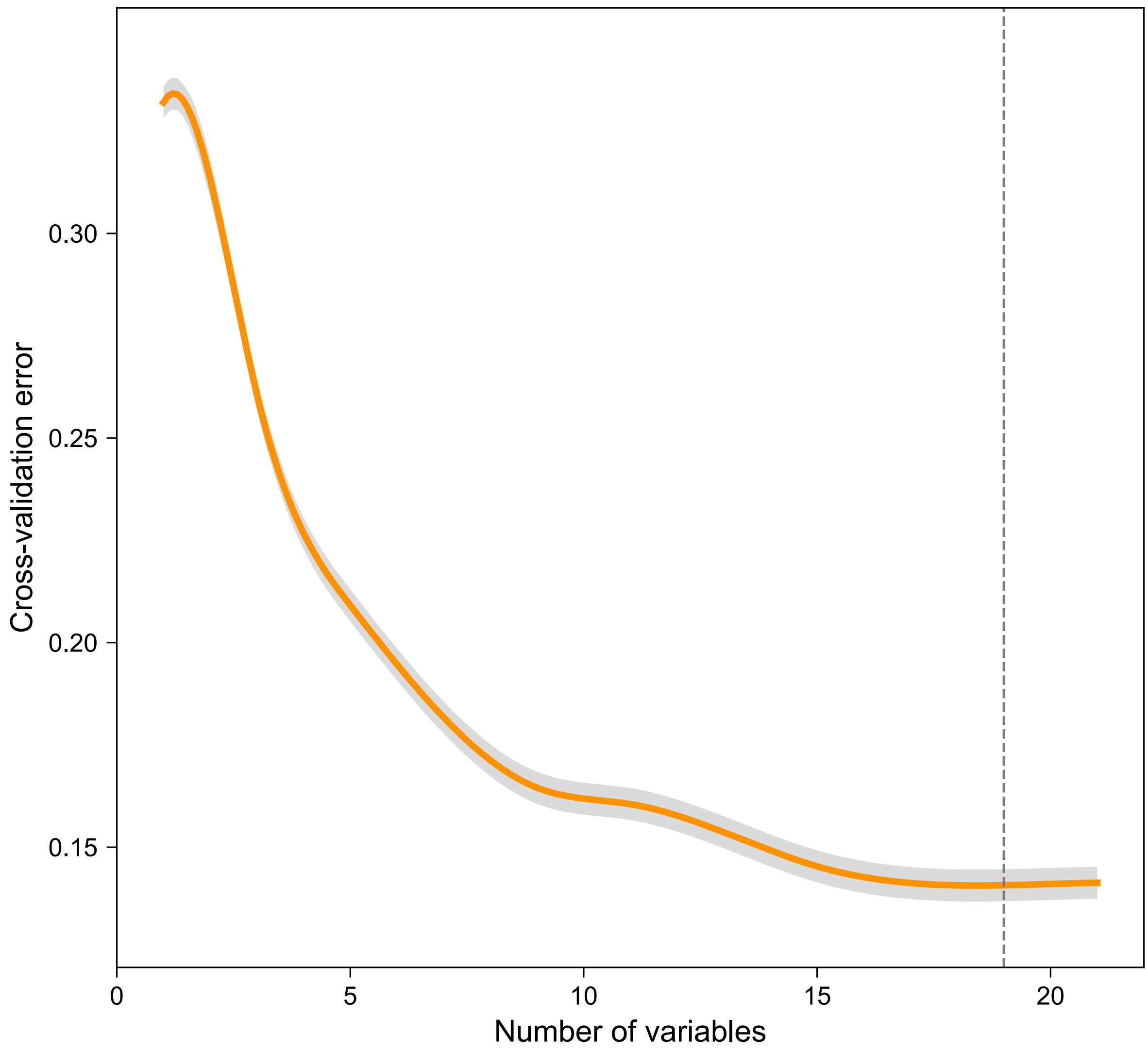

Figure S25. Feature selection and cross-validation of the random forest model for MIRS construction. Related to Figure 6

This plot shows the relationship between the number of microbial genera included in the random forest model and the corresponding cross-validation error. The x-axis indicates the number of variables (genera) ranked by importance based on mean decrease in accuracy, and the y-axis represents the tenfold cross-validation error. The orange line denotes the mean cross-validation error across folds, with the shaded area indicating the associated variability. The dashed vertical line marks the selected model incorporating the top 19 genera, which achieved a near-minimal cross-validation error and was subsequently used to construct the Microbial Inflammatory Risk Score (MIRS).

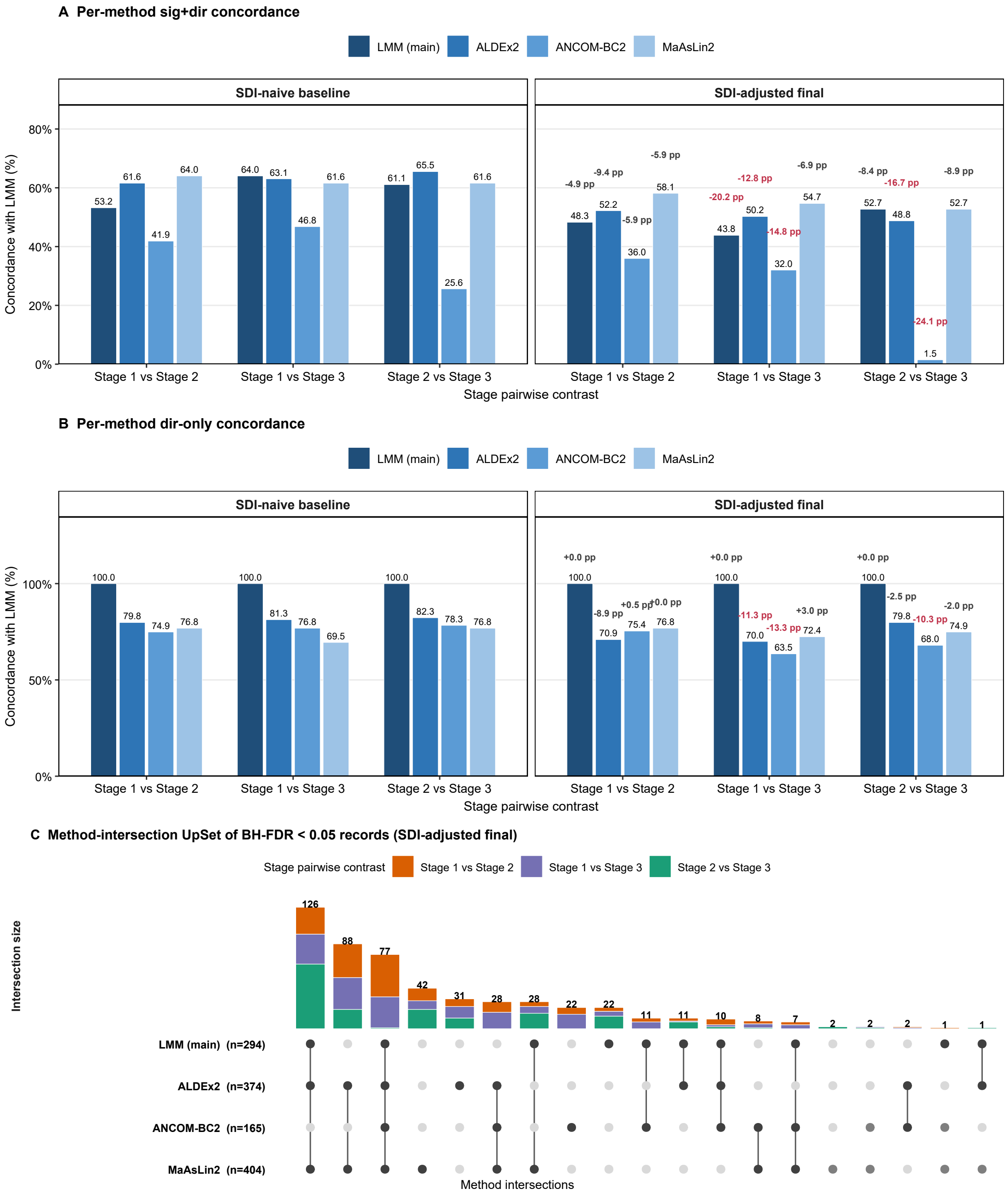

Figure S26.Sig+dir and dir-only concordance comparisons using 16S amplicon data. Related to Figure 3

The sig+dir % and dir-only % of four differential abundance (DA) analyses (two-sided) are shown in A and B, respectively. The reference direction is set to be the LMM main analysis. The Y-axis denotes patterns of percentage of LMM-concordant taxa. Numbers above bars are per-cell percentage values. By default, all four DA methods implement the BH procedure to adjust for multiple comparisons. Color and the name of the corresponding DA method are shown at the top within the graph. Two cohort-confounding scenarios are considered: SDI-naive baseline (no SDI covariate), as well as SDI-adjusted final (SDI as continuous covariate); number of modelled taxa = 203. Panel C shows the four-method UpSet of BH-FDR < 0.05 records under SDI-adjusted, stacked by stage pairwise contrast; the largest combination is LMM + ALDEx2 + MaAsLin2 (126 records) and the four- method full consensus is 77 records.

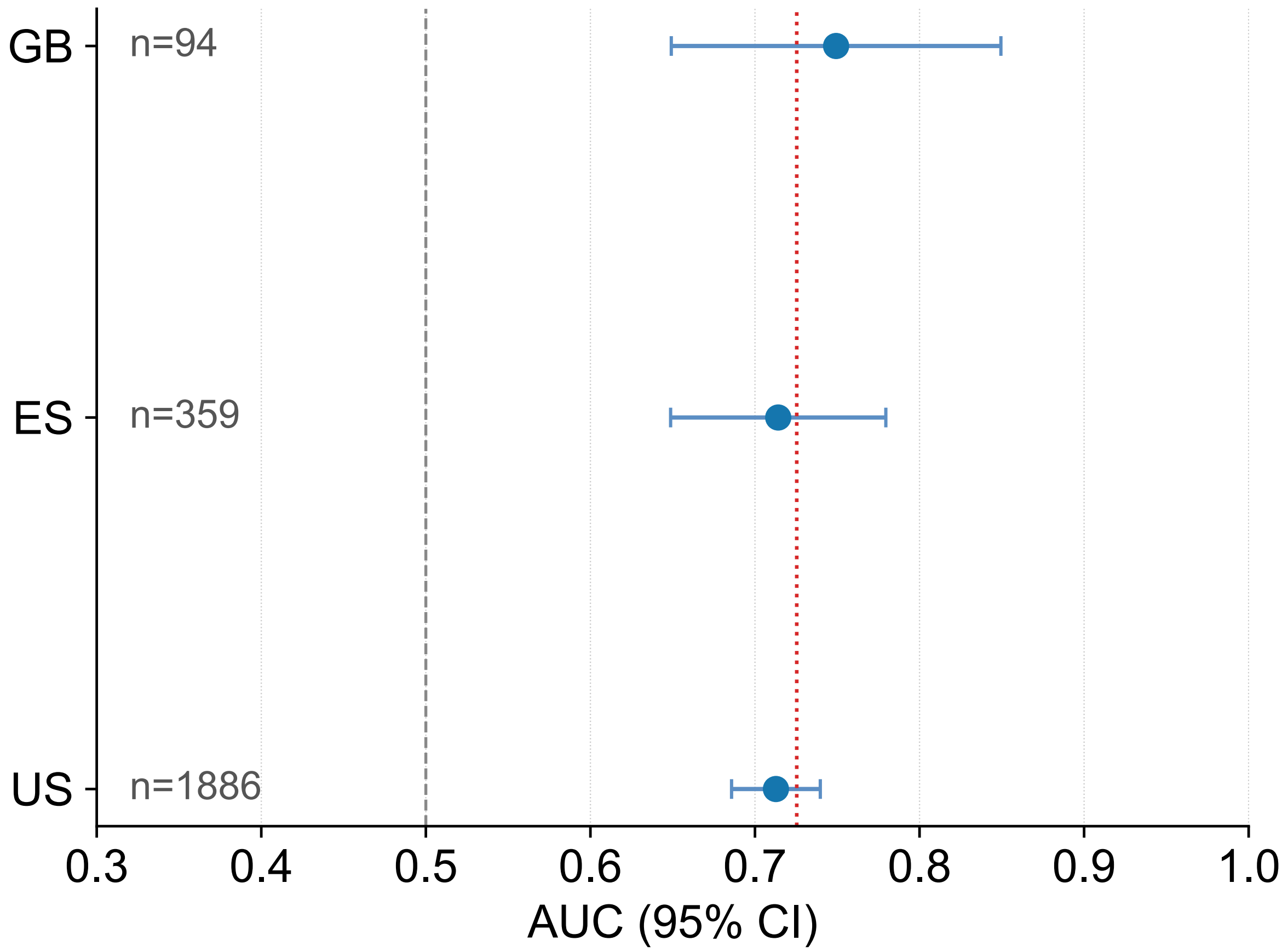

Figure S27. Leave-one-country-out cross-cohort generalisation of the IBD random forest classifier. Related to Figure 6

Forest plot showing per-country AUC of the 19-genera random forest classifier when one country at a time is held out from the metagenomic training pool. The x-axis denotes AUC with 95% confidence intervals, and the y-axis lists evaluable held-out countries by ISO alpha-2 code. The dashed vertical line marks AUC = 0.5; the dotted vertical line marks the mean AUC across evaluable countries.

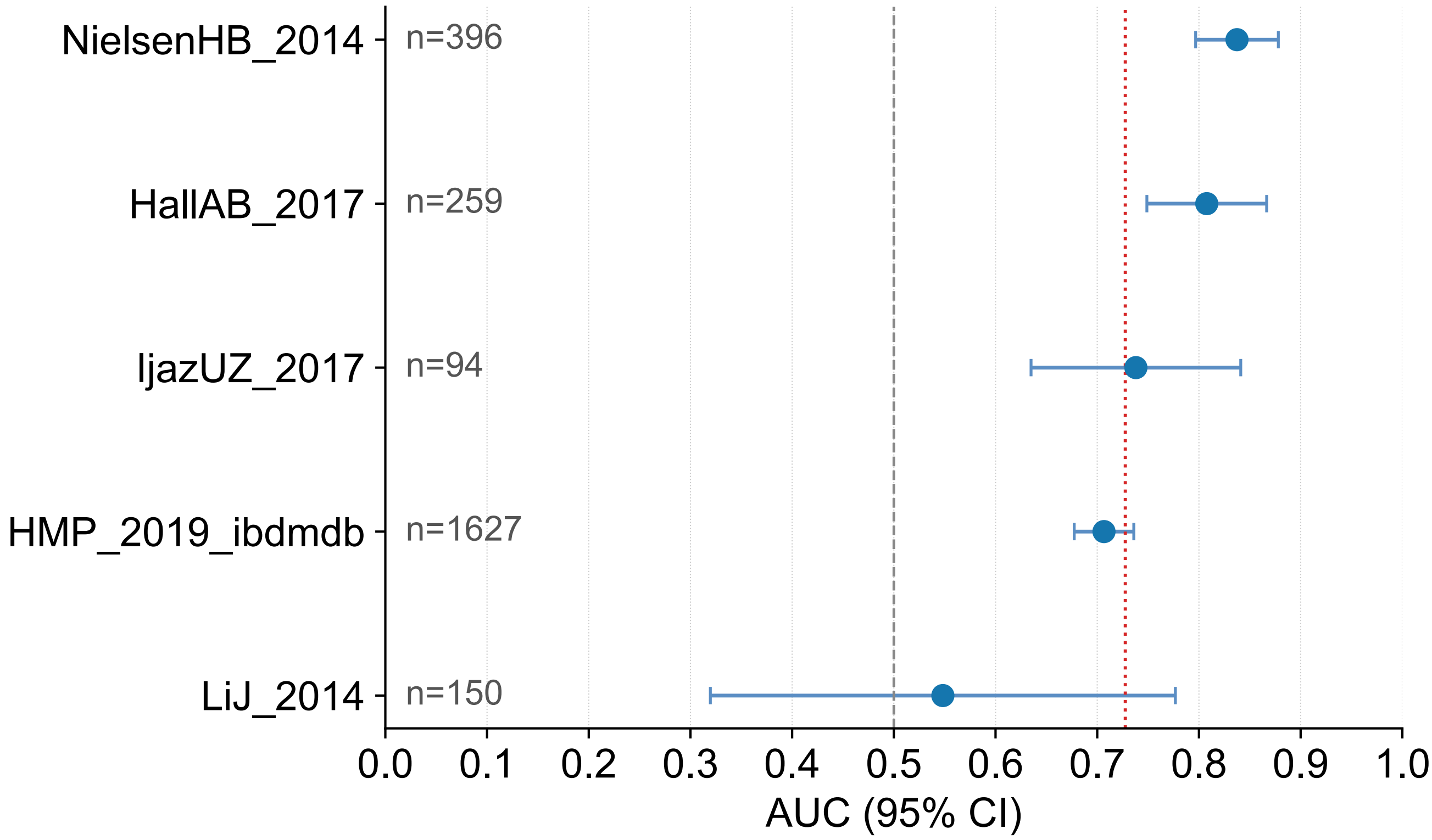

Figure S28. Leave-one-BioProject-out cross-cohort generalisation of the IBD random forest classifier. Related to Figure 6

Forest plot showing per-BioProject AUC of the 19-genera random forest classifier when one BioProject at a time is held out from the metagenomic training pool. The x-axis denotes AUC with 95% confidence intervals, and the y-axis lists evaluable held-out BioProjects. The dashed vertical line marks AUC = 0.5; the dotted vertical line marks the mean AUC across evaluable BioProjects.

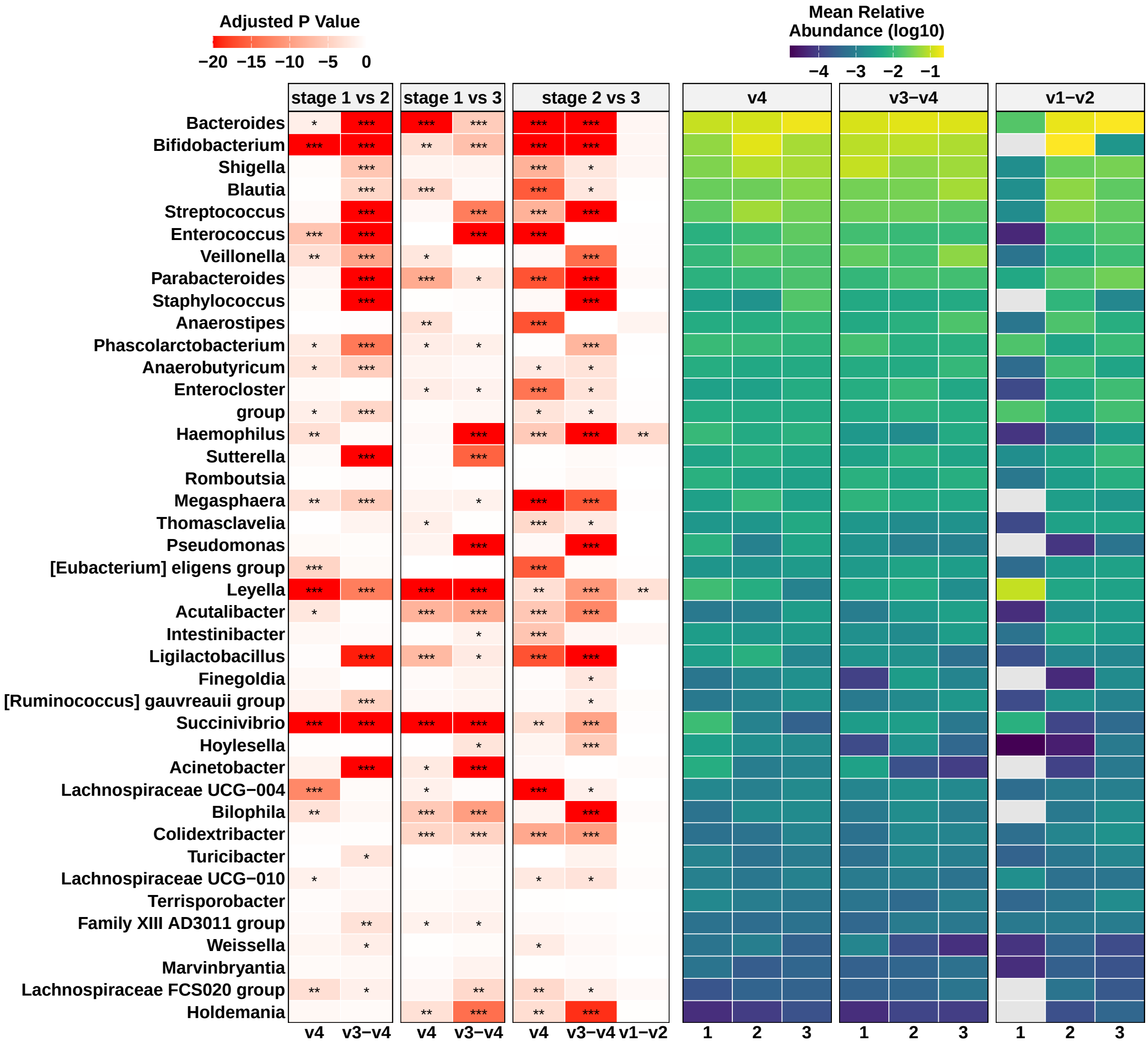

Figure S29. Taxa are differentially abundant between IBD stage-associated regions across V-region subsets. Related to Figure 3

The red-white heat map illustrates adjusted p-values for stage-wise differences within three V-region subsets. The y-axis lists the 41 taxa flagged by all four methods (LMM, ALDEx2, ANCOM-BC2, MaAsLin2; 0-4) as significant (BH-FDR < 0.05) and direction-concordant with LMM, and the x-axis lists V-region subsets within three stage-pairwise- contrast panels (Stage 1 vs. Stage 2, Stage 1 vs. Stage 3, Stage 2 vs. Stage 3). Each cell represents the strength of the differential abundance result for that taxon, with stars (*, **, ***) overlaying cells at BH-FDR < 0.05, 0.001, and 10^{-4}. The blue-green heatmap depicts the mean relative abundance (log10) of each taxon across the three V-region subsets, each stratified by stage (1, 2, 3) on the x-axis.

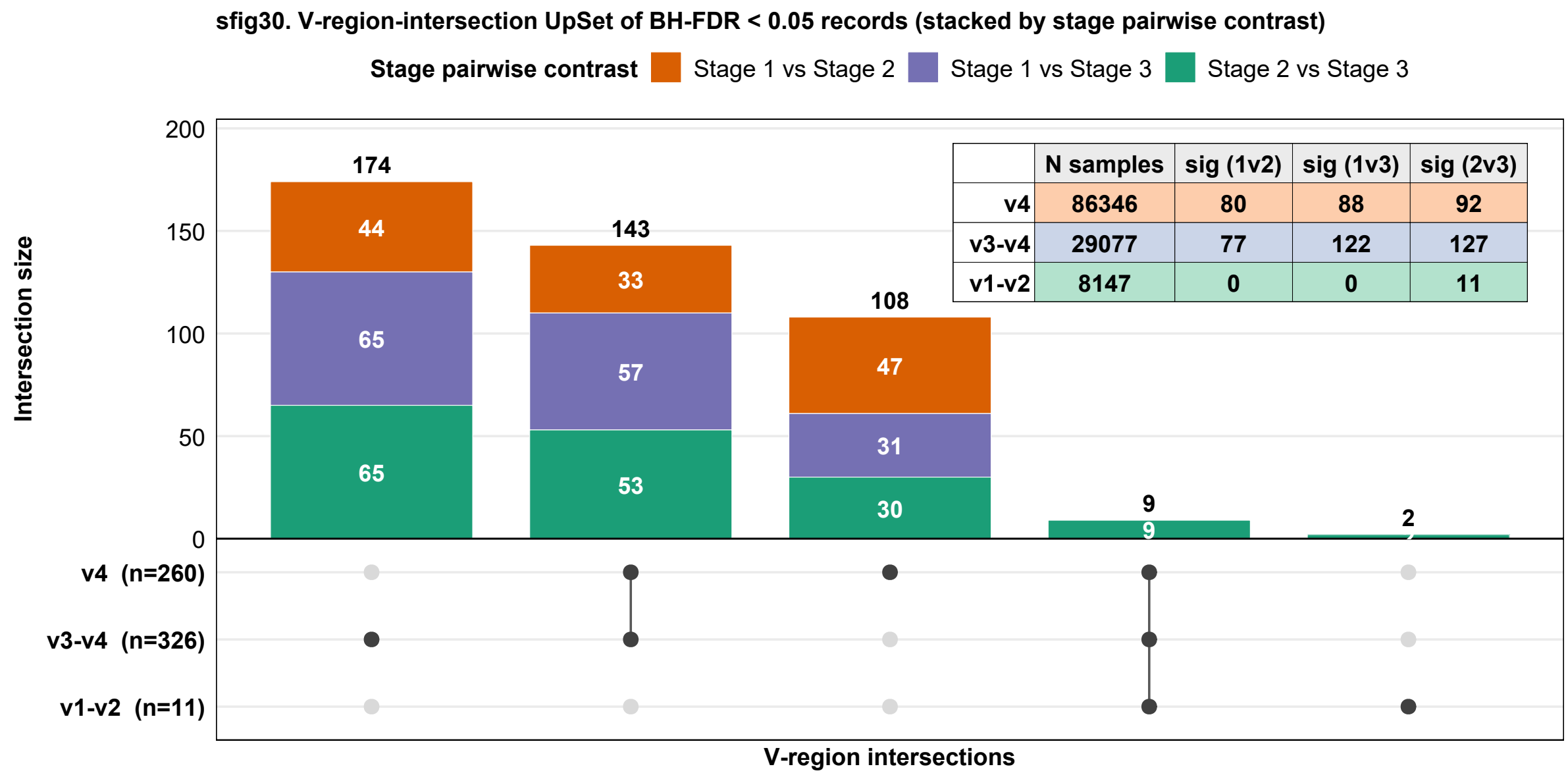

Figure S30. Cross-V-region consistency of BH-FDR- significant taxa across stage pairwise contrasts. Related to Figure 3

Overview of 436 BH-FDR < 0.05 records detected across three V-region subsets and three stage pairwise contrasts (Stage 1 vs. Stage 2, Stage 1 vs. Stage 3, Stage 2 vs. Stage 3), based on the per-subset LMM. Records show substantial cross-V-region consistency, with 58% (152/260) of v4 records also detected in v3-v4 and 82% (9/11) of v1-v2 records confirmed in both v4 and v3-v4.

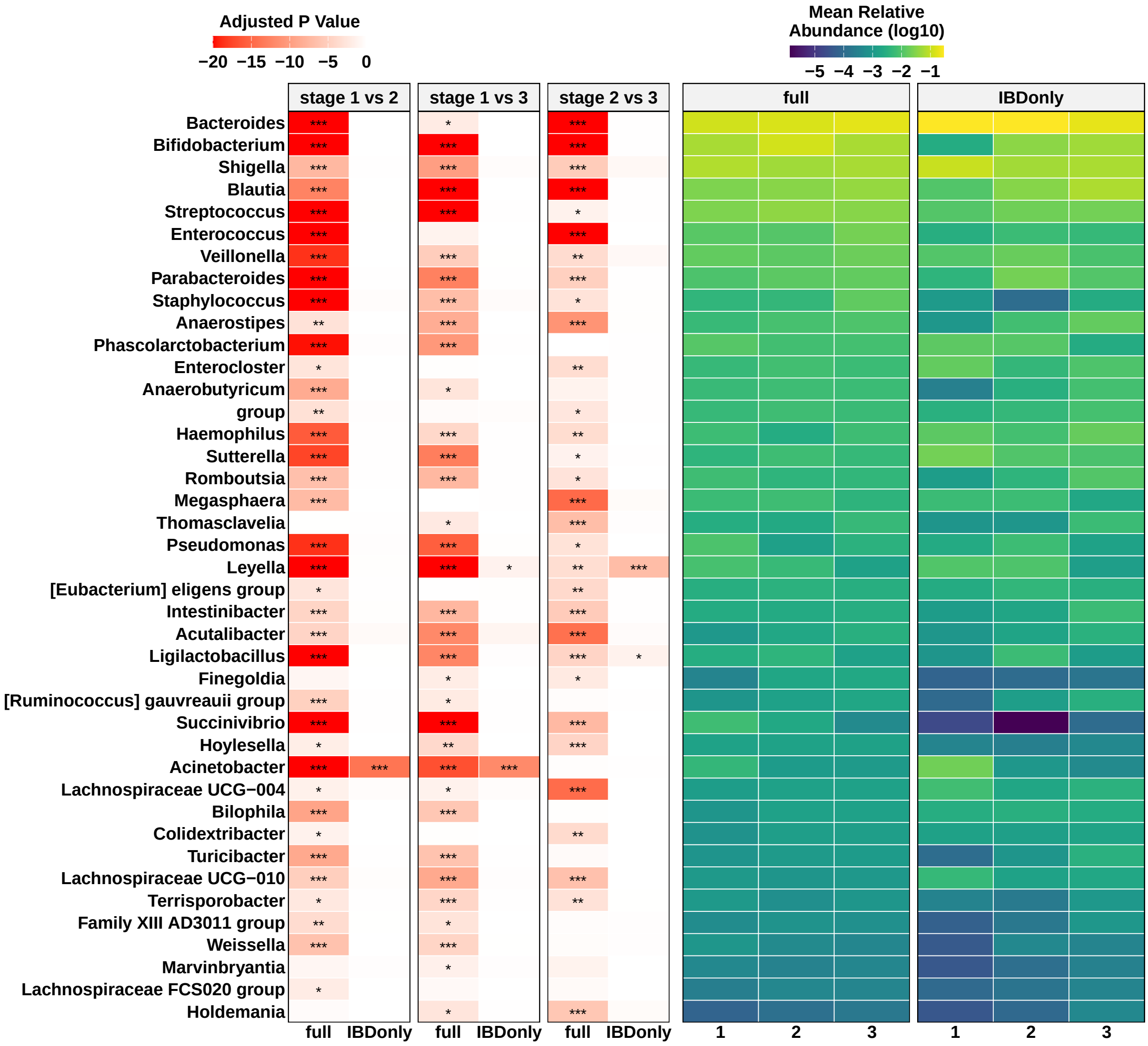

Figure S31. Taxa are differentially abundant between IBD stage-associated regions across both the full cohort and the single-disease IBD-only subcohort. Related to Figure 3

The red-white heat map illustrates adjusted p-values for stage-wise differences within two cohorts. The y-axis lists the 41 taxa flagged by all four methods (LMM, ALDEx2, ANCOM-BC2, MaAsLin2; 0-4) as significant (BH-FDR < 0.05) and direction-concordant with LMM, and the x-axis lists cohorts (full, n = 117,671; IBD-only, n = 4,171) within three stage-pairwise-contrast panels (Stage 1 vs. Stage 2, Stage 1 vs. Stage 3, Stage 2 vs. Stage 3). Each cell represents the strength of the differential abundance result for that taxon, with stars (*, **, ***) overlaying cells at BH-FDR < 0.05, 0.001, and 10^{-4}. The blue- green heatmap depicts the mean relative abundance (log10) of each taxon across the two cohorts, each stratified by stage (1, 2, 3) on the x-axis.

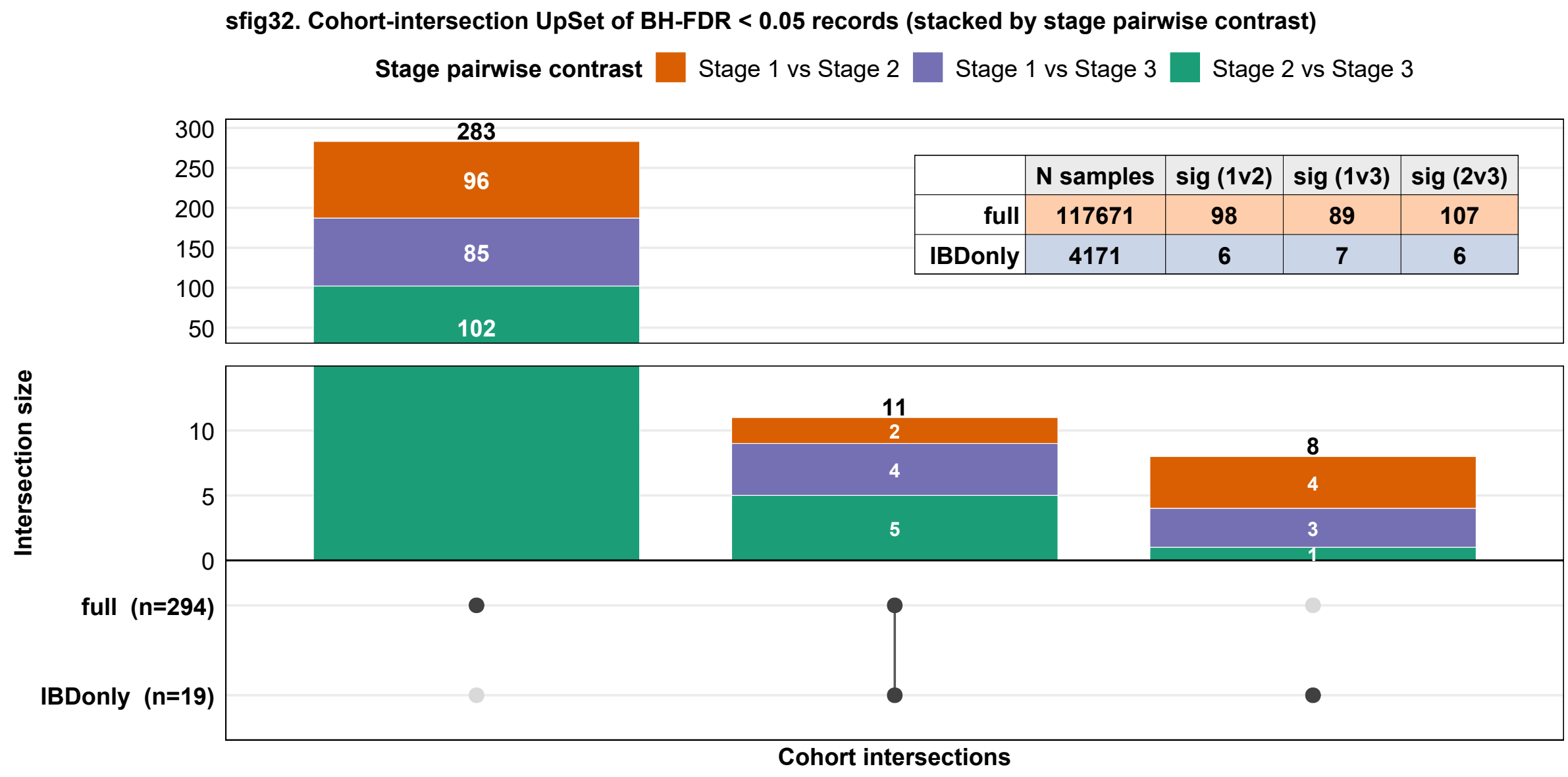

Figure S32. Cross-cohort consistency of BH-FDR- significant taxa between the full cohort and the IBD-only subcohort across stage pairwise contrasts. Related to Figure 3

The UpSet plot summarises the 302 unique taxon × stage-pair records reaching BH-FDR < 0.05 in at least one cohort, distributed across three stage pairwise contrasts (Stage 1 vs. Stage 2, Stage 1 vs. Stage 3, Stage 2 vs. Stage 3), based on the per-cohort LMM. Records show substantial cross-cohort consistency, with 58% (11/19) of IBD-only records also detected in the full cohort and 100% (11/11) of these doubly-significant records being direction-concordant between cohorts.

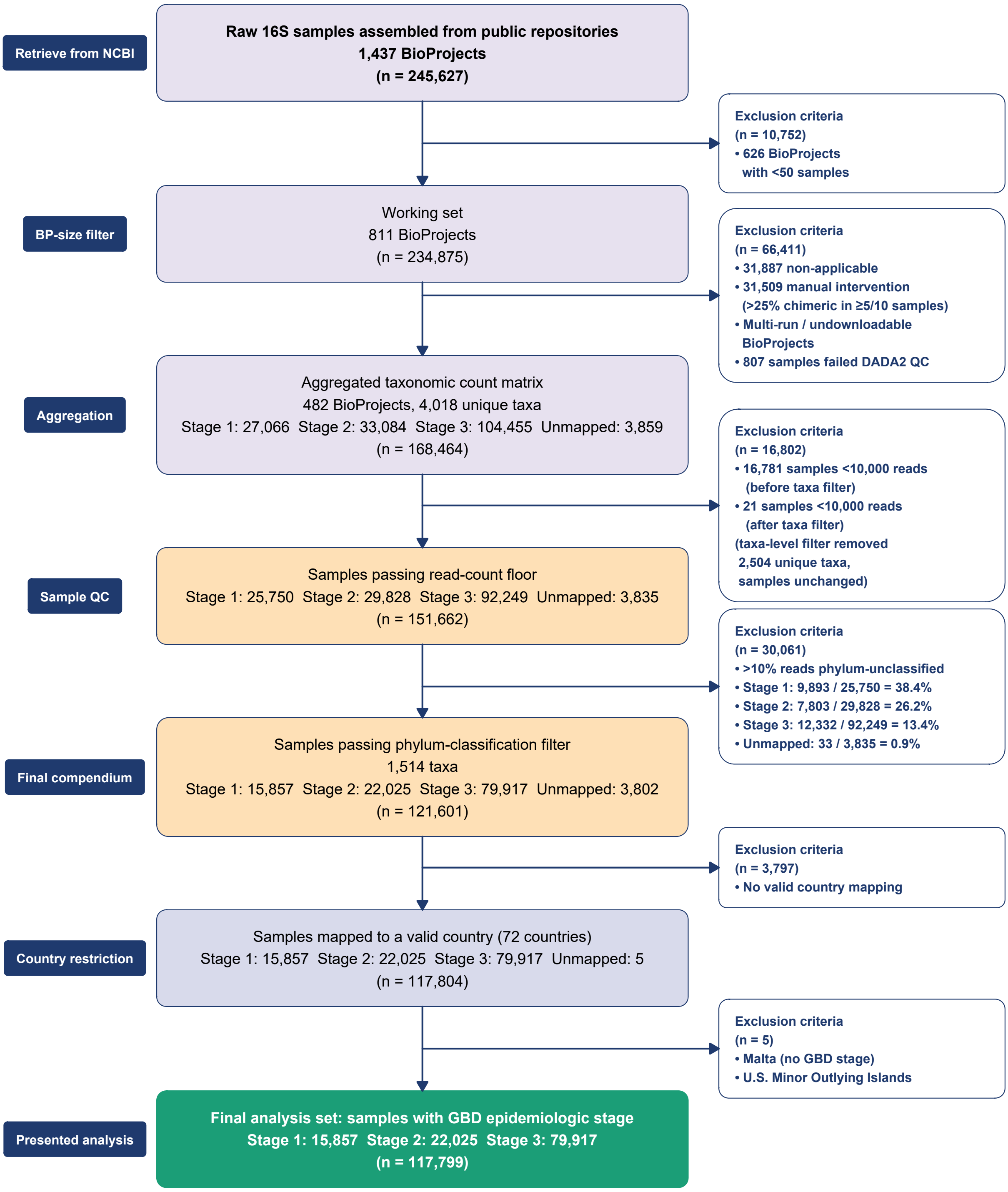

Figure S33. Overview of the sample-retention flow for the 16S compendium. Related to Figure 1

A list of the general steps in the data pipeline and how many samples completed each step. See Methods for more details about each process. From the aggregation step onwards, the number of samples in each GBD epidemiologic stage (Stage 1, Stage 2, Stage 3, and Unmapped) is shown within each step.

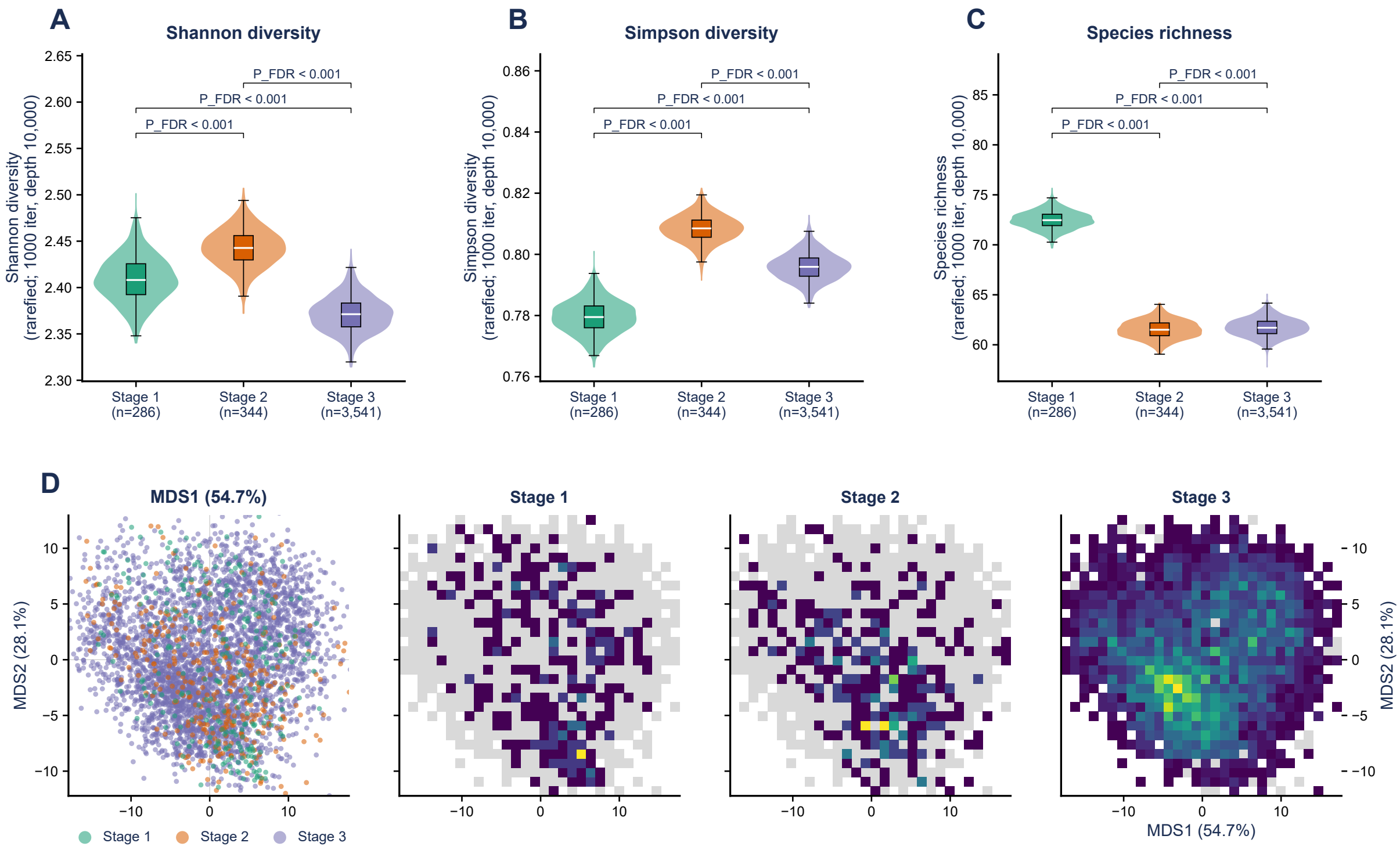

Figure S34. Alpha-diversity and PCoA analyses on an IBD-only subset. Related to Figure 1

(A–C) Violin plots showing the distributions of rarefied Shannon index, Simpson index, and species richness values for samples from the IBD-only subset assigned to the three IBD stages. (D) A series of plots illustrating the results of a PCoA of samples from all IBD stages. In the leftmost scatter plot, each point represents a single sample; the x-axis corresponds to the first PCoA axis, which explains the greatest proportion of variation in the dataset, and the y-axis corresponds to the second PCoA axis, which explains the second-greatest proportion of variation. The other three plots use the same axes but include only samples from a single IBD stage. These plots employ a heatmap design rather than a scatter plot to better visualize areas with high point overlap— yellow areas indicate regions of the ordination space with a higher density of samples, while dark blue areas represent regions with few (but not zero) samples. The gray shading indicates the area occupied by all samples across all stages.

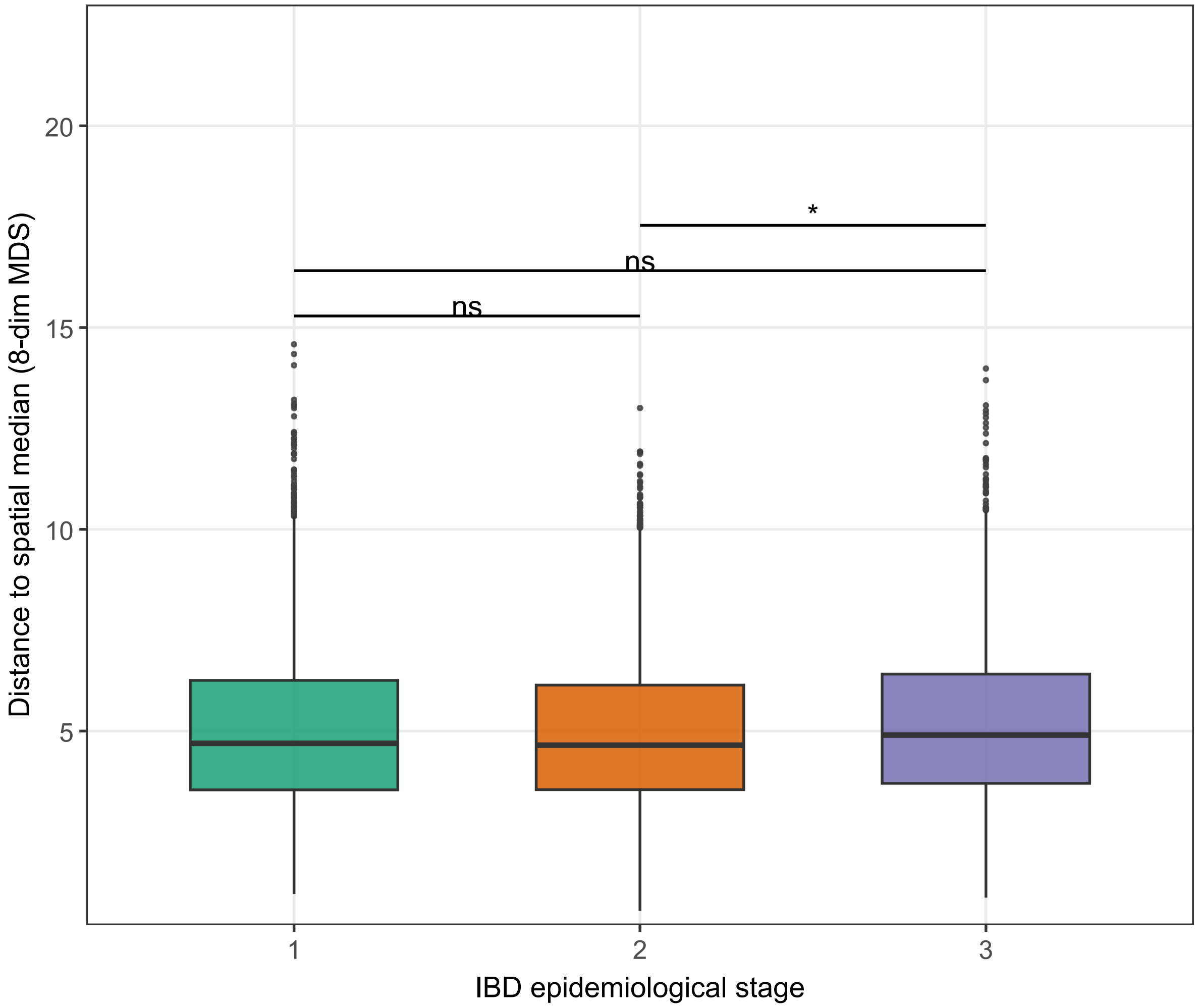

Figure S35. Multivariate dispersion across the three IBD epidemiological stages. Related to Figure 1

Boxplots showing the per-sample distance to the spatial median in the eight-dimensional MDS embedding for samples from countries assigned to the three IBD stages. The colors used here match those shown in Figure 1H. Statistical comparisons were performed using PERMDISP2.

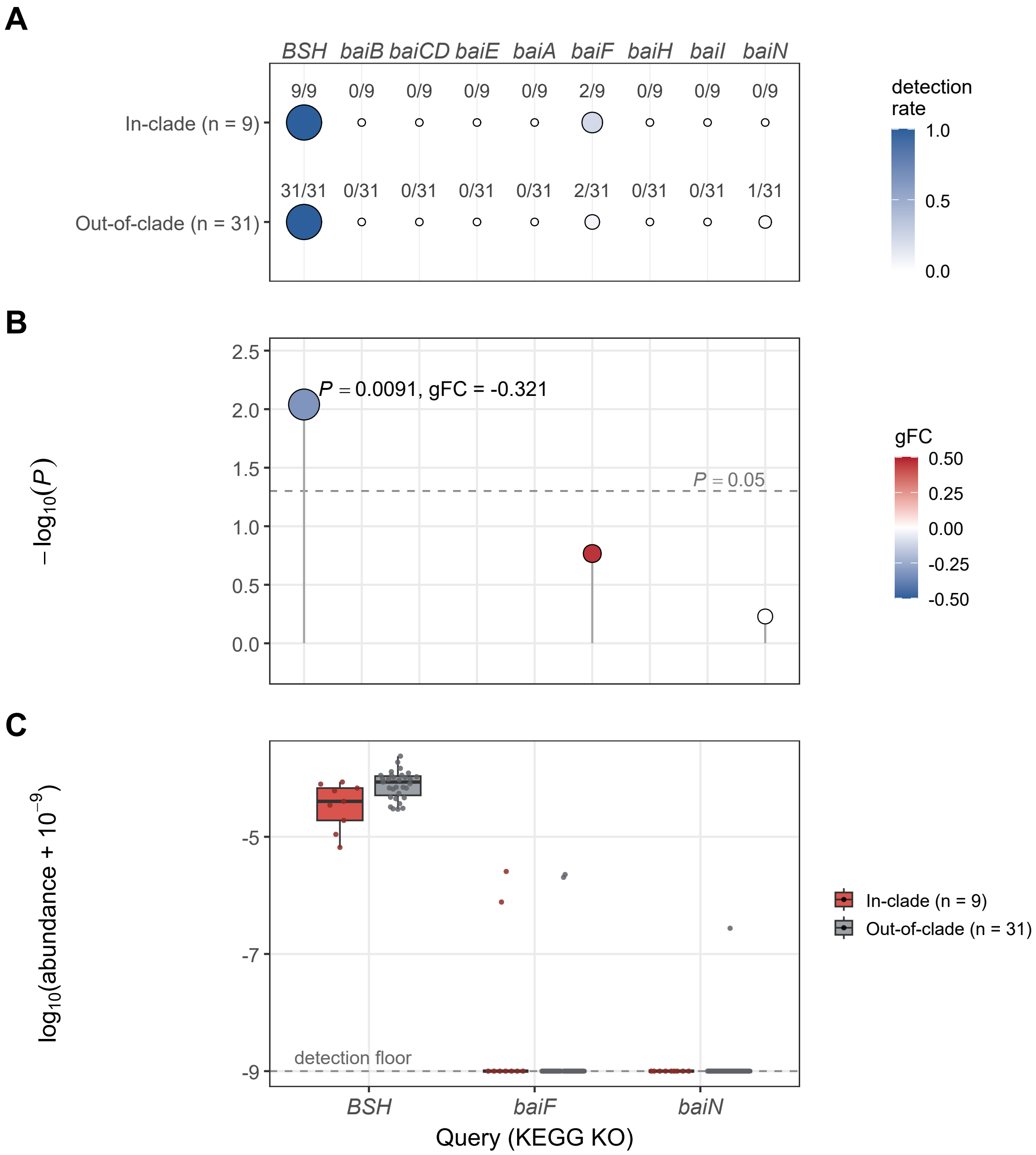

Figure S36. Subclade-level functional validation of bile acid metabolism genes in the IBD-enriched SGB4993 clade. Related to Figure 7

(A) Dot plot showing per-group detection rate of each query. (B) Lollipop plot showing $-\log_{10}$(blocked Wilcoxon *P*) per query; point fill encodes the generalized fold change (gFC), and the dashed line marks $P = 0.05$. (C) Boxplot showing per-sample $\log_{10}$ UniRef90 abundance for the three testable queries (BSH, baiF, baiN); the dashed line marks the detection floor.

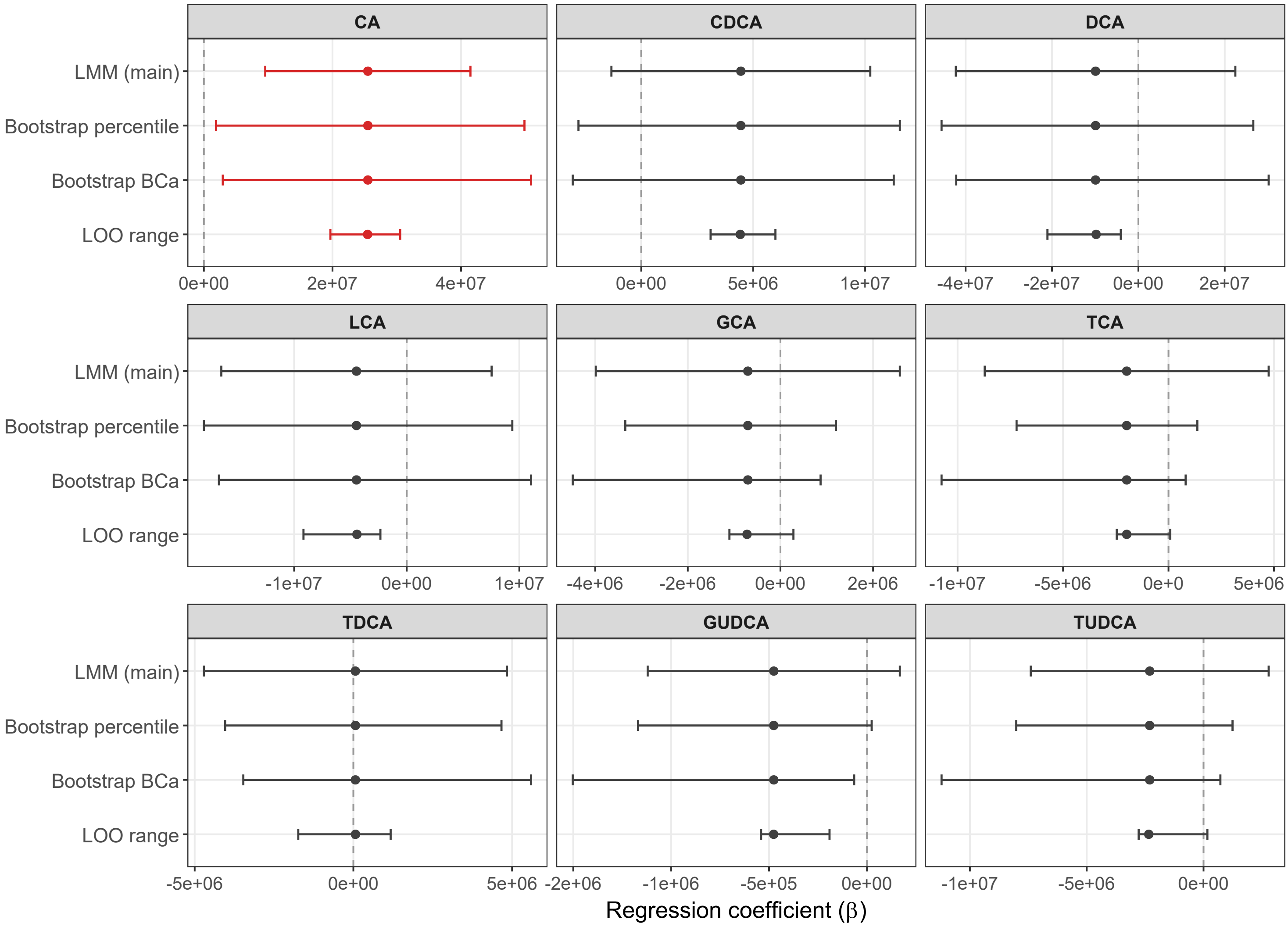

Figure S37. Robustness of the SGB4993 × bile-acid association across four estimate views per bile acid. Related to Figure 7

Forest plot showing the regression coefficient (β) (in-clade vs. out-of-clade) for each of the nine measured bile acids on the HMP2 IBDMDB n = 40 cohort (9 in-clade vs. 31 out-of-clade). The x-axis denotes the regression coefficient (β) for the in-clade-vs.-out-of-clade contrast, and the y-axis lists the four estimate views (LMM main, bootstrap percentile, bootstrap BCa, leave-one-out range). The dashed vertical line marks $\beta = 0$. All four estimate views for CA are shown in red as the only bile acid reaching BH-FDR < 0.05 in the LMM main analysis.
